# A Critical Evaluation of Vibrational Stark Effect (VSE) Probes with the Local Vibrational Mode Theory

**DOI:** 10.3390/s20082358

**Published:** 2020-04-21

**Authors:** Niraj Verma, Yunwen Tao, Wenli Zou, Xia Chen, Xin Chen, Marek Freindorf, Elfi Kraka

**Affiliations:** 1 Department of Chemistry, Southern Methodist University, 3215 Daniel Avenue, Dallas, TX 75275-0314, USA; nirajverma288@gmail.com (N.V.); ywtao.smu@gmail.com (Y.T.); mfreindorf@gmail.com (M.F.); 2 Institute of Modern Physics, Northwest University, Xi’an 710127, China; qcband@gmail.com; 3 Hubei Key Laboratory of Natural Medicinal Chemistry and Resource Evaluation, School of Pharmacy, Tongji Medical College, Huazhong University of Science and Technology, Wuhan 430030, China; d201881314@hust.edu.cn; 4 Laboratory of Theoretical and Computational Chemistry, Institute of Theoretical Chemistry, Jilin University, Changchun 130023, China; chenxin1211@mails.jlu.edu.cn

**Keywords:** Stark spectroscopy, vibrational Stark effect, VSE, local vibrational mode theory, normal mode decomposition, vibrational Stark effect probes, infrared spectroscopy, electric field, carbonyl, nitrile

## Abstract

Over the past two decades, the vibrational Stark effect has become an important tool to measure and analyze the in situ electric field strength in various chemical environments with infrared spectroscopy. The underlying assumption of this effect is that the normal stretching mode of a target bond such as CO or CN of a reporter molecule (termed vibrational Stark effect probe) is localized and free from mass-coupling from other internal coordinates, so that its frequency shift directly reflects the influence of the vicinal electric field. However, the validity of this essential assumption has never been assessed. Given the fact that normal modes are generally delocalized because of mass-coupling, this analysis was overdue. Therefore, we carried out a comprehensive evaluation of 68 vibrational Stark effect probes and candidates to quantify the degree to which their target normal vibration of probe bond stretching is decoupled from local vibrations driven by other internal coordinates. The unique tool we used is the local mode analysis originally introduced by Konkoli and Cremer, in particular the decomposition of normal modes into local mode contributions. Based on our results, we recommend 31 polyatomic molecules with localized target bonds as ideal vibrational Stark effect probe candidates.

## 1. Introduction

The Stark effect refers to the response of a spectroscopic transition to an applied electric field. It was discovered by Stark in 1913 observing that an applied electric field causes a splitting in the absorption lines of hydrogen [1]. In 1995, Chattopadhyay and Boxer reported that the infrared absorbance of the CN stretching mode in the anisonitrile molecule changed proportionally with the strength of an electric field imposed on the molecule [2]. Since then, the vibrational Stark spectroscopy (VSS) has become an important analytical method, which has been summarized over the past decade in a series of review articles [3,4,5,6,7,8,9,10,11]. The vibrational Stark effect (VSE) describes the perturbation of a vibrational frequency by an electric field [2,3,5,12] according to Equation (1):

(1) $$\nu =\nu_{0}-\left(\Delta \vec{\mu }\cdot \vec{F}+\frac{1}{2}\vec{F}\cdot \Delta \alpha \cdot \vec{F}\right)$$

where ν and $\nu_{0}$ are the vibrational frequencies of a specific molecular vibrational mode (i.e., the target bond stretching mode in most cases) with (ν) and without ($\nu_{0}$) an external electric field $\vec{F}$, respectively; $\Delta \vec{\mu }$ is the difference dipole moment (also known as *Stark tuning rate*) and Δα is the difference polarizability in a VSE experiment. The electric field strength is in general below 100 MV/cm; therefore, the quadratic term with regard to Δα in Equation (1) can be neglected, so that the change in the vibrational frequency $\Delta \nu =\nu -\nu_{0}$ directly correlates with the change in the strength of the electric field $\vec{F}$ [5].

This linear relationship between vibrational frequency and electric field has formed the basis for the vibrational Stark spectroscopy. Given a simplified electrostatic description of non-covalent interactions between the vibrational probe and surrounding molecules, the strength of these intermolecular interactions can be assessed by the electric field a target chemical bond feels, as revealed by the VSE [5,13]. The VSE has been extensively applied to study the non-covalent interactions in different types of chemical systems and environments including proteins/enzymes [6,7,8,10,11,14,15,16,17,18,19,20,21,22,23,24,25,26,27,28,29,30,31,32,33], nucleic acids [34,35], ionic liquids [36,37], biological membranes [38], electrochemical interfaces/surfaces [12,39,40,41,42,43], and polymers [3,44,45]. Recently, the range of applications has been extended to the investigation of water clusters [46,47] and molecular solids [48].

These applications have been based on the following four underlying assumptions [2,5,26,29,49,50,51]:

1. The normal stretching vibration of a probe bond (e.g., the C=O bond in formaldehyde) is considered to be largely decoupled from rest of the molecule, i.e., its associated normal mode is ideally localized, which is generally not the case [52,53,54,55,56];
2. The vibrational frequency shift Δν arising from changes in the vicinal environment of the probe molecule can be fully attributed to the external electric field. This is the basic foundation for using the VSE as a tool to characterize non-covalent interactions;
3. The difference dipole moment $\Delta \vec{\mu }$ in Equation (1) is unaffected by the external electric field $\vec{F}$, so that the vibrational frequency shift Δν responds to $\vec{F}$ in a linear fashion;
4. The linear relationship between vibrational frequency and the electric field, observed for a relatively weak electric field strength (in the order of 1 MV/cm) will also hold for the binding pocket of proteins, where the effective electric field caused by the enzyme environment could be a hundred times stronger.

The first assumption is the most important as the vibrational Stark effect is based on a simplified model assuming that the probe bond stretching vibration encodes all information about the surrounding electric field. However, to the best of our knowledge, no systematic study on the extent to which those commonly applied and/or potential vibrational Stark effect probes can meet this requirement, has been reported so far. To fill this gap, we used in this work as powerful tool the *characterization of normal mode* (CNM) procedure, which is an important part of the local vibrational mode analysis originally developed by Konkoli and Cremer [57,58,59,60]. CNM determines quantitatively to what extent the local stretching vibrational mode of the probe bond is decoupled from the other local vibrational modes of the probe, and therefore provides a unique measure to assess the qualification of a probe molecule.

This paper is structured in the following way: First, the local vibrational mode theory including the CNM method is summarized and it is discussed how CNM can be applied to evaluate a vibrational Stark effect probe in the Methodology part. Then, the Computational Details are given. In the Results and Discussion part, 68 VSE probes and candidates with C=O, C≡N, S=O and other types of probe bonds are analyzed and scored with the CNM approach. A complete set of 107 VSE probes is given in the Supplementary Information. Furthermore, the sensitivity of the calculated scores with regard to density functional is checked. Lastly, 31 probe molecules with high scores are recommended for experimental verification and application.

## 2. Methodology

The VSE and its related spectroscopy require that the normal vibrational mode of the probe bond stretching is decoupled from the local vibrational modes led by other internal coordinates within the probe molecule [5,51], which does not comply with the fact that normal vibrational modes are generally delocalized over several part of a molecule or even the whole molecule because of the mass coupling [52,53,54,55,56,61].

A prominent example is the popular Tolman electron parameter (TEP) which assesses the metal-ligand (ML) bond strength in nickel-tricarbonyls [Ni(CO)_3_L] indirectly using the $A_{1}$-symmetrical CO stretching mode as probe. The TEP rests upon the assumption that the $A_{1}$-symmetrical CO stretching mode is fully localized and does not couple with other local modes [62,63,64]. However, our local mode analysis clearly revealed that this assumption is generally not true [65,66,67]. This indicates that, also in a polyatomic VSE probe, the normal vibrational mode of the probe bond stretching may not be ideally localized, which will impact its qualification for characterizing VSE. Therefore, a method is needed to quantify the local character of the probe bond vibration and it can be easily applied to existing VSE probes and potential probe candidates. In the following, we will review the CNM method, developed by Konkoli, Larsson and Cremer in 1998 [59,60,68,69] within the framework of the local vibrational mode theory [57], which is the perfect tool to determine in a quantitative way to what extent the normal vibration of the probe bond stretching is consisting of pure stretching character.

The harmonic normal vibrational modes and corresponding frequencies for a polyatomic molecular system with *N* atoms in its equilibrium geometry can be obtained by solving the Wilson equation of vibrational spectroscopy [52,70]:

(2) $$f^{x}L=\mathrm{ML}\Lambda$$

where $f^{x}$ is the Hessian matrix in terms of 3N Cartesian coordinates with the dimension of 3N×3N. Matrix M is the diagonal mass matrix accounting for all *N* atoms in *x*, *y*, and *z* directions. The diagonal matrix Λ in the ($N_{vib}\times N_{vib}$) dimension contains $N_{vib}$ vibrational eigenvalues $\lambda_{\mu }$ ($\mu =1,\ldots ,N_{vib}$ with $N_{vib}=3N-K$) and *K* equals six or five for nonlinear or linear molecules, respectively. The ($3N\times N_{vib}$) dimensional matrix L has $N_{vib}$ vibrational eigenvectors $l_{\mu }$ as column vectors which are orthonormal to each other. The vibrational frequencies $\omega_{\mu }$ can be connected with eigenvalue $\lambda_{\mu }$ according to $\lambda_{\mu }=4\pi^{2}c^{2}\omega_{\mu }^{2}$ where *c* is the speed of light.

Equation (2) can be rewritten in terms of normal coordinates Q [52,70] as

(3) $$f^{Q}=K=L^{T}f^{x}L$$

where K is the Hessian matrix expressed in normal coordinates Q with dimension ($N_{vib}\times N_{vib}$) and $L^{T}$ is the transpose of L.

Konkoli and Cremer defined a local vibrational mode via the *leading parameter principle* [57], which states that a local vibration is initiated by an associated internal coordinate $q_{n}$ via its infinitesimal change. Only the masses of the atoms involved in this internal coordinate $q_{n}$ are kept, the masses of all other atoms are assigned a zero value. As a consequence, the other atoms of the molecule can effortlessly follow the local vibration led by $q_{n}$ as a collection of massless points. The internal coordinate $q_{n}$ can be associated with the Cartesian coordinates x of the molecule via the Wilson B-matrix [52] which collects the partial derivatives of $q_{n}$ with regard to Cartesian coordinates x

(4) $$b_{n}=\frac{\partial q_{n}}{\partial x}$$

The local mode vector $a_{n}$ associated with internal coordinate $q_{n}$ is then defined by

(5) $$a_{n}=\frac{K^{-1}d_{n}^{T}}{d_{n}K^{-1}d_{n}^{T}}$$

with $d_{n}=b_{n}L$ [70]. The local mode vector $a_{n}$, a column vector of length $N_{vib}$ can be transformed into Cartesian coordinates via Equation (6) [57,61]

(6) $$a_{n}^{x}=La_{n}$$

To each local mode $a_{n}$, local mode properties can be assigned, such as a local mode force constant, frequency and mass [57]. The *local mode force constant* $k_{n}^{a}$ of internal coordinate $q_{n}$ is obtained with

(7) $$k_{n}^{a}=a_{n}^{T}Ka_{n}={\left(d_{n}K^{-1}d_{n}^{T}\right)}^{-1}$$

The local mode force constant $k_{n}^{a}$ was also named *adiabatic force constant*, where *a* (adiabatic) as the superscript means “relaxed” and *n* as the subscript corresponds to the internal coordinate $q_{n}$ leading this local vibration [57].

The *local mode mass* $m_{n}^{a}$ of mode *n* is given by

(8) $$m_{n}^{a}=1/G_{n,n}={\left(b_{n}M^{-1}b_{n}^{T}\right)}^{-1}$$

where $G_{n,n}$ is the *n*-th diagonal element of the Wilson **G** matrix [52,70].

Local mode force constant and mass are needed to determine the *local mode frequency* $\omega_{n}^{a}$

(9) $${\left(\omega_{n}^{a}\right)}^{2}=\frac{1}{4\pi^{2}c^{2}}k_{n}^{a}G_{n,n}$$

Zou and co-workers demonstrated that, for a complete non-redundant set of $N_{vib}$ local modes, there exists a one-to-one relationship between local and normal vibrational modes that can be verified by an adiabatic connection scheme (ACS), providing the physical fundament for the local vibrational modes [61]. The reason why a complete set of non-redundant parameter set is required in such relation is because this set of local vibrational modes can span the same internal vibration space spanned by $N_{vib}$ normal modes [71,72,73]. In addition, this one-to-one correspondence between the local and normal vibrational modes forms the basis for the CNM method leading to a detailed analysis of a vibrational spectrum and in this way decoding a wealth of information embedded in the spectrum [65,74]. It is also important to note that this analysis can be applied to both calculated and experimentally determined fundamental vibrational frequencies [75].

According to the CNM method [59], any normal vibrational mode $l_{\mu }$ can be decomposed into local mode contributions from a non-redundant set of $N_{vib}$ local vibrational modes by calculating the overlap between each local mode vector $a_{n}^{x}$ with this normal mode vector $l_{\mu }$ as $S_{n\mu }$ according to Equation (10)

(10) $$S_{n\mu }=\frac{{\left(a_{n}^{x},l_{\mu }\right)}^{2}}{\left(a_{n}^{x},a_{n}^{x}\right)\left(l_{\mu },l_{\mu }\right)}$$

where (a,b) is a short notation for the scalar product of two vectors of a and b

(11) $$\left(a,b\right)=\sum_{i,j}a_{i}O_{ij}b_{j}$$

$O_{ij}$ is element within the metric matrix O. In this work, we used the force constant matrix $f^{x}$ as the metric (i.e., $O=f^{x}$) to include the influence from the electronic structure.

The calculation of $S_{n\mu }$ in Equation (10) was simplified via the following steps. If we consider the complete set of $N_{vib}$ normal modes collected in L and the non-redundant set of $N_{vib}$ local modes collected in $A^{x}$, Equation (10) can be written as

(12) $$S=\frac{{\left(A^{x},L\right)}^{2}}{\left(A^{x},A^{x}\right)\left(L,L\right)}$$

where

(13) $$\left(A^{x},L\right)={A^{x}}^{T}f^{x}L$$

(14) $$\left(A^{x},L\right)={\left(LA\right)}^{T}f^{x}L$$

(15) $$\left(A^{x},L\right)=\frac{DK^{-1}L^{T}}{DK^{-1}D^{T}}f^{x}L$$

where D=BL collects $d_{n}$ as row vectors, then

(16) $$\left(A^{x},L\right)=\frac{DK^{-1}K}{DK^{-1}D^{T}}$$

(17) $$\left(A^{x},L\right)=\frac{D}{DK^{-1}D^{T}}$$

and

(18) $$\left(A^{x},A^{x}\right)={A^{x}}^{T}f^{x}A^{x}$$

(19) $$\left(A^{x},A^{x}\right)=\frac{DK^{-1}L^{T}}{DK^{-1}D^{T}}f^{x}\frac{LK^{-1}D^{T}}{DK^{-1}D^{T}}$$

(20) $$\left(A^{x},A^{x}\right)=\frac{DK^{-1}}{DK^{-1}D^{T}}K\frac{K^{-1}D^{T}}{DK^{-1}D^{T}}$$

(21) $$\left(A^{x},A^{x}\right)=\frac{DK^{-1}}{DK^{-1}D^{T}}\frac{ID^{T}}{DK^{-1}D^{T}}$$

(22) $$\left(A^{x},A^{x}\right)=\frac{I}{DK^{-1}D^{T}}$$

with

(23) $$\left(L,L\right)=L^{T}f^{x}L=K$$

Then, Equation (10) can be re-written as

(24) $$S_{n\mu }=\frac{{\left(A^{x},L\right)}_{n\mu }^{2}}{{\left(A^{x},A^{x}\right)}_{n}{\left(L,L\right)}_{\mu }}$$

(25) $$S_{n\mu }=\frac{\frac{D_{n\mu }^{2}}{{\left[DK^{-1}D^{T}\right]}_{nn}^{2}}}{\frac{1}{{\left[DK^{-1}D^{T}\right]}_{nn}}K_{\mu \mu }}$$

(26) $$S_{n\mu }=\frac{D_{n\mu }^{2}k_{n}^{a}}{K_{\mu \mu }}$$

where $K_{\mu \mu }$ is the μ-th diagonal element of matrix K. As long as the molecular system is at a local minimum on the potential energy surface, both $k_{n}^{a}$ and $K_{\mu \mu }$ are positive thus leading to a positive value of $S_{n\mu }$. It can be easily proven that $S_{n\mu }$ is independent of the prefactor inside an internal coordinate as a linear combination of a few basic internal coordinates (e.g., $S_{n\mu }$ stays the same whatever nonzero value *p* takes if the internal coordinate $q_{n}$ is defined by $q_{n}=p\cdot \left(q_{a}\pm q_{b}\right)$).

Therefore, the contribution of local mode $a_{n}$ to the normal mode $l_{\mu }$ can be calculated by

(27) $$C_{n\mu }=\frac{S_{n\mu }}{\sum_{m}^{N_{vib}}S_{m\mu }}$$

In order to evaluate the degree to which a normal vibrational mode $l_{\mu }$ involving the stretching of the probe chemical bond is decoupled from all other internal coordinates, a complete non-redundant set of internal coordinates including the one for the probe bond (denoted as $q_{1}$) has to be constructed and the corresponding local mode vectors $a_{n}$ need to be determined as well as their overlap with the target normal vibrational mode $l_{\mu }$. In this way, one can quantify the extent to which the normal mode has predominantly probe bond local stretching character and determine the actual percentage via

(28) $$C_{1\mu }=\frac{S_{1\mu }}{\sum_{m}^{N_{vib}}S_{m\mu }}$$

$C_{1\mu }$ is a number ranging from 0 to 1. A large $C_{1\mu }$ value indicates that normal vibration mode $l_{\mu }$ has predominantly the local vibration led by internal coordinate $q_{1}$ and a value of 1 would identify mode $l_{\mu }$ as a 100% local probe bond stretching vibration. For simplicity, we coined the term ”performance score” being defined as the $C_{1\mu }$ percentage value to evaluate and compare different vibrational Stark effect probes in the remainder of this work.

**Figure 1:**
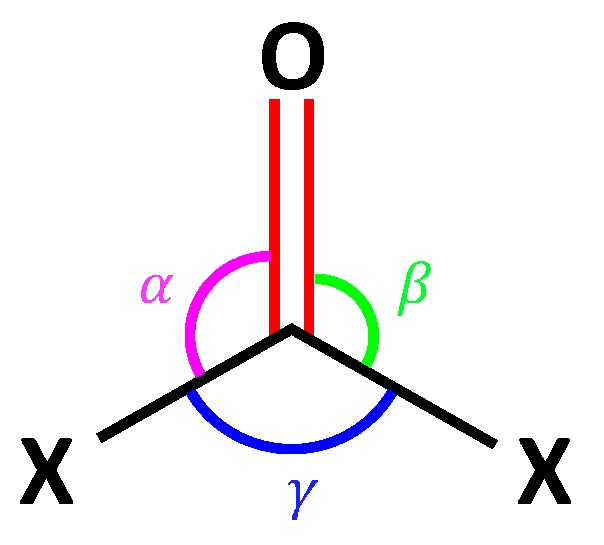
The three angles α, β and γ for a molecule with topology X_2_CO. A balanced choice of bond angles for the non-redundant parameter set consists of (i) angle γ and (ii) angle δ, which is an antisymmetric combination of angles α and β (i.e., δ=α−β).

Theoretically speaking, any complete and non-redundant internal coordinate parameter set (including the probe bond) could be used for normal mode decomposition; however, in order to better accommodate the rocking (asymmetric bending) vibration in formaldehyde-like topology (see Figure 1), we employed an antisymmetric combination (denoted as δ) of two angles containing the probe bond (e.g., C=O) when analyzing these probe molecules.

**Figure 2:**
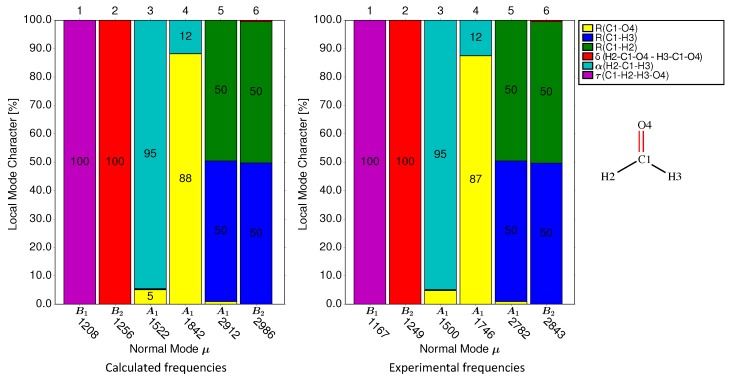
Decomposition of each normal vibrational mode into the contributions from six local vibrational modes for formaldehyde molecule. The labels under the *x*-axis are the irreducible representations and the vibrational frequencies (in cm${}^{-1}$) of normal modes. In the right panel, the decomposition result have been calibrated using experimentally measured vibrational frequencies of formaldehyde in the gas phase [76]. The uncalibrated calculated vibrational frequencies and decomposition result are shown in the left panel. The geometry optimization and Hessian calculation were carried out at ωB97XD/aug-cc-pVDZ level of theory.

In the following, the procedure of calculating the performance score for a VSE probe is demonstrated for formaldehyde (**1-1**) as an example. This molecule contains four atoms and has as such six normal vibrational modes. We selected as non-redundant set of six internal coordinates the three bond lengths, two bond angles (including δ), and one dihedral angle (τ) shown in Figure 2, which also shows the decomposition of each of the six normal vibrational modes into local mode components in the form of a bar diagram for both experimental and calculated vibrational frequencies. The two H−C−O angles were anti-symmetrically combined to angle δ. The probe bond vibration is colored in yellow.

The normal mode decomposition of formaldehyde (see Figure 2) clearly identifies normal mode 4 as probe bond stretching with the largest local C=O stretching contribution, i.e., $C_{n\mu }$ value of 0.8816 and a contribution of 12% from the local H−C−H angle bending mode. As such, the performance score of formaldehyde as the VSE probe is 88.

The potential energy distribution (PED) method [77,78,79,80,81,82] widely used in vibrational spectroscopy has been applied in some scattered investigation to analyze vibrational Stark effect probes [83]. PED takes a step back and it is based on the idea that the potential energy can be expressed as a power series in terms of normal coordinates Q, which can be further decomposed into internal coordinates $q_{n}$. In contrast, the CNM method as part of the Konkoli–Cremer local vibrational mode theory is directly based on vibrational spectroscopy and local modes can be smoothly transitioned to normal modes via adiabatic connection scheme [67]. In this sense, CNM analysis is superior than PED analysis as the former has better physical fundament in terms of vibrational spectroscopy [58,59,60,84].

**Figure 3:**
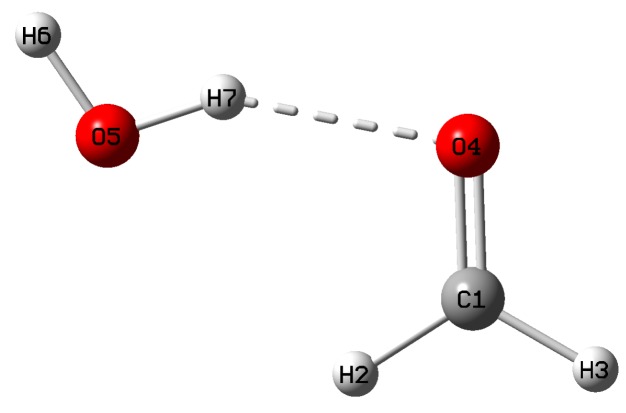
Formaldehyde molecule connected with one water molecule via a hydrogen bond. The geometry was optimized at ωB97XD/aug-cc-pVDZ level of theory.

One may raise concern on the suitability of calculating the performance score based on an isolated probe molecule model as in real application scenarios a probe molecule may have non-covalent interactions with surrounding molecules. Therefore, we constructed a model system of formaldehyde which is hydrogen bonded with a water molecule as shown in Figure 3.

The target normal vibration of C=O stretching consists of the local vibrations of 87.2% R(C1=O4) and 12.6% α(H2−C1−H3). As such, in the hydrogen bonded complex, the performance score for the formaldehyde molecule is 87.2, only somewhat smaller than the score of 88.2 for the single molecule shown in Figure 2 by 1.0 unit. Such minor difference in performance score can be safely ignored. Therefore, in the remainder of this work, we stick to the single molecule approach for the calculation of the performance score of the probe molecules.

## 3. Computational Details

Geometry optimizations and Hessian evaluations for all probe molecules investigated in this work was carried out in the gas phase using the Gaussian 16 package [85]. All molecules were optimized using the ωB97XD density functional [86] with Dunning’s aug-cc-pVDZ basis set [87,88,89] except molecules containing a porphyrin group, which were calculated at the M06L/def2TZVP level [90,91,92].

The decomposition of the normal vibrational modes into local mode contributions was carried out with the COLOGNE2019 package [93]. In order to test the sensitivity of the decomposition results with regard to the density functional employed in this work, all calculations were repeated at the M06-2X/aug-cc-pVDZ level [94] except for molecules containing a porphyrin group.

## 4. Results and Discussion

In this section, (i) we evaluate the performance of commonly used VSE probes in comparison with a number of potential vibrational probe candidates, using the CNM method. These polyatomic probe molecules contain C=O, C≡N, S=O or other chemical bonds, whose stretching vibrations are considered as decoupled from other local vibrational modes. (ii) We analyze how the atomic masses influence the performance of selected VSE probes and discuss the feasibility of improving a vibrational probe by isotopic substitution. (iii) In addition, we analyze how the performance score of a VSE probe depends on the density functional used for the calculation. (iv) Practical suggestions on the ideal VSE probes to experimentalists are made with related physicochemical properties (e.g., solubility and reactivity).

### 4.1. Group 1: C=O/C≡O Probes

**Figure 4:**
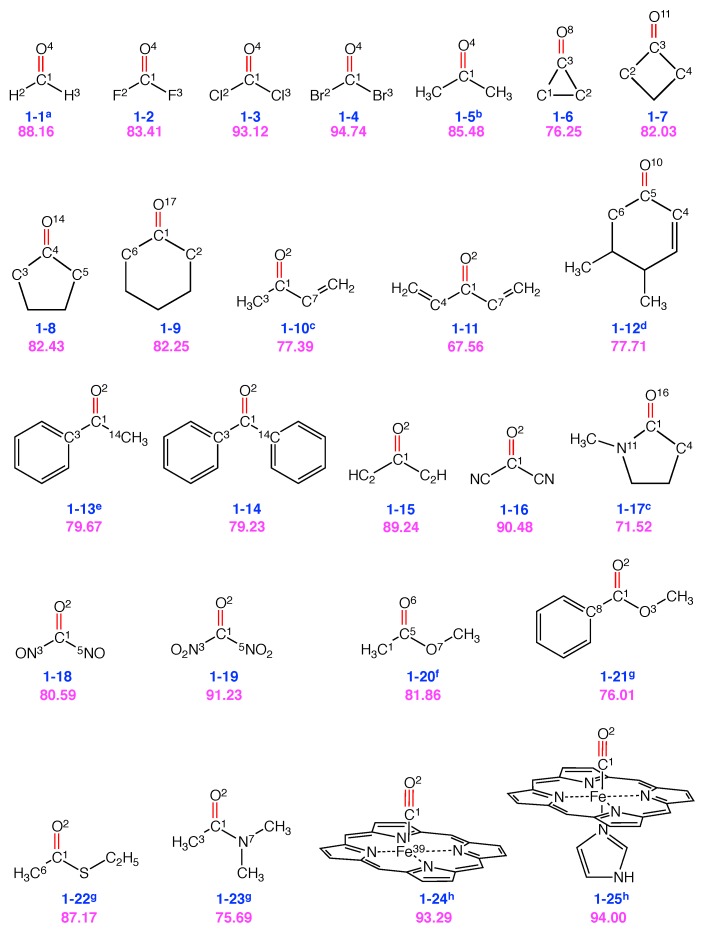
Group 1: 25 VSE probes with C=O or C≡O probe bonds in red. Below each structure, a label of **n-m** is given in blue; **n** denotes the group number (reflecting the type of VSE probe bond and **m** is the number of the molecule within this group. Vibrational probe labels with superscripts are taken from the literature (a [95], b [5,96], c [96], d [5,7,25,28], e [5,13,97], f [13], g [13,26], h [14,15,98]). The corresponding performance score as VSE probe is given in purple. The number as superscript in the 2D structure refers to atom index in the molecule. The superscripts are shown only for the atoms whose associated local modes participate in the C=O or C≡O normal mode with more than 5% contribution.

Figure 4 shows a list of 25 typical VSE probes with C=O or C≡O VSE probe bonds and their performance scores. A complete list of all probes tested in this work and their decomposition of normal modes into local modes are provided in the Supporting Information.

**Figure 5:**
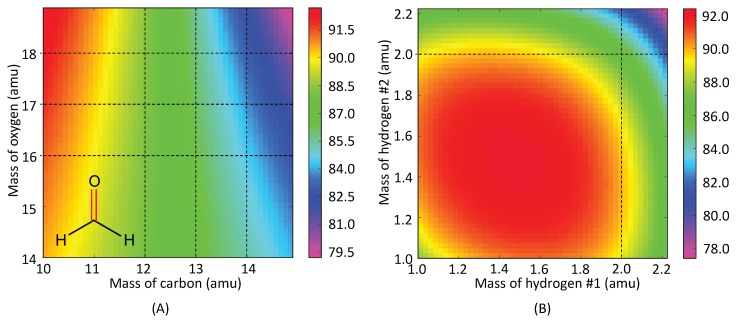
Performance score of formaldehyde as a function of atomic masses in (**A**) carbon and oxygen atoms and (**B**) two hydrogen atoms, respectively. Red is for higher score while purple is for the lower score. The dashed lines indicate the atomic mass values for isotopes.

Table 1 shows the decomposition of target normal mode of probe bond stretching into corresponding local modes where all contributions greater than 5% are shown. Formaldehyde **(1-1)** (described in more detail in the Methodology Section) has relatively simple structure and its performance score is 88. As revealed by Equation (26), the overlap $S_{n\mu }$ is composed of different terms, $k_{n}^{a}$ characterizes the pure electronic effect, whereas $D_{n\mu }$ and $K_{\mu \mu }$ depend on the normal modes, which involve the atomic mass, geometry, and electronic effect. This implies that the performance score is a result of multiple factors. As McKean has shown in his work that the isotopic substitution could result in isolated (i.e., local) modes of CH bond stretching in -CD${}_{2}$H groups [99], it would be interesting to see if similar strategy could lead to better performed VSE probes. In order to do so, we calculated the performance score landscape for **1-1** as a function of the atomic masses as shown in Figure 5.

Changing the atomic masses of carbon and oxygen atoms (the two atoms of the VSE probe bond) from the most abundant isotopes (${}^{12}$C and ${}^{16}$O) to higher hypothetical isotopes results only in a trivial change (ca. –3.0) of the performance score. By replacing the hydrogen atoms with deuterium, the performance score drops down to 86.07. The isotope effect seems to play a complex role on the performance score and is not directly intuitive for us to decide which atomic mass to change.

Carbonyl fluoride (**1-2**), carbonyl chloride (**1-3**) and carbonyl bromide (**1-4**) have a similar structure as **1-1** except that the hydrogen atoms are replaced with more electronegative halogen atoms X. The performance score of 83 for **1-2** is the lowest in this series, gradually increasing for the higher homologues, i.e., 93 for **1-3** and 95 for **1-4**. With the increase in the atomic number in the series F, Cl and Br, the electronegativity of X decreases, the atomic mass increases, and the C−X bond becomes longer and weaker, which should lead to less coupling with the C=O stretching. This is obviously in line with the calculated performance scores. However, it is oversimplified to conclude that the difference in the performance score for these three molecules can be completely attributed to electronic effects unless the mass effect can be eliminated. Therefore, we performed model calculations using the same atomic mass in order to extract the electronic effect, as shown in Table 2.

Table 2 shows how the performance scores of these four molecules are affected by the atomic mass of X. When the atomic mass of X is kept the same for all of the four molecules, the performance scores show a consistent pattern: **1-4** > **1-3** > **1-1** > **1-2**. As expected, the lowest performance score is found for **1-2**, (i.e., shortest and strongest C−X bonds leading to a large mode-mode coupling with the C=O target bond). The highest score is found for **1-4** as its longest and weakest C−X bonds leading to only moderate coupling with the C=O target bond. The electronegative F atoms in **1-2** attract electron and lead to stronger C−X bonds. The joint contribution from the local stretchings of two C-F bonds to the target normal vibration is 10.4%. The less electronegative Br atom in **1-2** is less suited to attract electrons, which is linked to its larger atomic radius leading to longer C−Br bonds, whose local vibration contribution to the target normal vibration is less than 5% in total. Overall, this result reveals that the performance score differences are mainly caused by electronic structure differences and not by a significant mass effect.

Compared to substituent effects, isotopic effects are a less effective choice in tweaking the performance score. One also has to consider that isotopic substitution is often experimentally demanding and time consuming. Therefore, in the remainder of this work, we will focus on substituent effects. The carbonyl group can be incorporated into a ring structure. When the C=O bond is attached to cycloalkyl groups (**1-6**, **1-7**, **1-8** and **1-9**), the performance score is relatively low (⩽83) due to the large contamination from the local vibrations of the adjacent C−C single bonds. For example, in cyclopropanone (**1-6**), two local C−C vibrations adjacent to the C=O bond contribute jointly 19% to the target normal vibration mode dominated by the local C=O stretching. Meanwhile, the performance score remains almost the same with increasing ring size. However, the second largest contribution from adjascent C−C−C bond angle to the C=O stretching normal mode in **1-8** (8%) and **1-9** (5%) decreases, which can be related to decreasing ring strain. In 3,4-Dimethyl-2-cyclohexen-1-one (**1-12**) the C=O bond is attached to a six-membered ring. **1-12** is a simplified motif of an inhibitor which has been frequently used to measure the electric field inside the binding pocket of ketosteroid isomerase [5,22,25]. Its relatively low performance score of 78 compares to the scores of cyclopentanone (**1-8**) and cyclohexanone (**1-9**) with similar ring structures. A slight difference in the performance score can be attributed to the extended electron density (i.e., π-conjugation) in **1-12**. The CNM analysis shows that the local C−C−C angle bending contributes to 7%.

In *N*-methylpyrrolidione (**1-17**), the C=O bond is connected to a five-membered ring, but it has even lower performance score than **1-8**. This is caused by the conjugation between the lone pair electrons of nitrogen atom with the π electrons of the double bond, and this conjugation results in a large contribution from the local vibration of the C−N bond stretching (8%) and N−C−C angle bending (11%) to the target normal mode. A similar situation occurs also in dimethylacetamide (**1-23**), which has a performance score of 76.

Acetone (**1-5**) has moderate performance score of 85 as VSE probe. The contamination of the target normal vibration results from the local vibrations of the two C−C bonds and the bending of C−C−C adjacent to the C=O bond. If the methyl group in **1-5** is replaced by a vinyl group as in but-3-en-2-one (**1-10**) and penta-1,4-dien-3-one (**1-11**), the performance score drops to 77 and 68, respectively. This can be explained by the π conjugation between the C=C and C=O bonds leading to kinematic coupling [61]. The local C−C−C angle bending contributes 5% in **1-10** and 7% in **1-11** to the target normal vibration.

Acetophenone (**1-13**) has been used to measure the electric field inside various solvents [13,97]. Its performance score is 80, which is slightly lower than that of acetone. While most contamination of the target normal mode of **1-13** is caused by the adjacent C−C−C angle bending (6%), the CC π bonds of the phenyl ring conjugated with the C=O bond contribute 3% to the target normal vibration collectively. Replacement of the methyl group in **1-13** with another phenyl group leading to benzophenone (**1-14**) does not impact the performance score significantly. However, replacing the methyl group of **1-13** with a methoxy group leading to methyl benzoate (**1-21**) decreases the performance score to 76. The CNM analysis identifies for **1-21** the adjacent C−C−O angle bending and C−C local bond stretching vibrations as important contributors to the target normal vibration with 7% and 6%, respectively. Similar contribution patterns to the target normal mode are found for methyl acetate (**1-20**) with a performance score of 82. In ethyl thioacetate (**1-22**), the performance score is raised to 87. The major contamination arises from the adjacent C−C bond. While the local C−C bond stretching contribution is 5% and the C−S bond contribution is less than 5%.

In 1,4-pentadiyn-3-one (**1-15**) and oxo-malononitrile (**1-16**), triple bonds are connected to the carbonyl group increasing the performance scores to 89 and 90, respectively. It could be expected that the π conjugation between the triple bond and the C=O bond might lower the performance score as in **1-10** and **1-11**. However, due to the linear C−C≡C/N bond angle arising from *sp*-hybridized carbon, the target normal vibration is then dominated by the local vibration of the C=O bond stretching. In addition, the local C−C single bond stretching and C−C−C angle bending vibrations contribute with less than 5% to the target normal mode.

In the case of 1,3-dioxourea (**1-18**), local C−N bond stretching vibrations jointly contribute 12.8% to the target normal mode, leading to a relatively low performance score of 81. If the nitroso groups are replaced with nitro groups as in nitro ketone (**1-19**), the performance score raises to 91. This large difference in performance score is caused by the delocalization of π electrons. In **1-18**, all atoms are in the same plane leading to extended delocalization of π electrons. However, in the case of **1-19**, not all atoms are in the same plane and therefore it leads to weaker delocalization and higher performance score.

Carbon monoxide can serve as a diatomic VSE probe [100]. When coordinated to the iron atom in iron porphyrin (**1-24**), the C≡O ligand can be used to characterize the electric field in enzymes [7,10]. According to our analysis, **1-24** has a high performance score of 93. The local Fe−C bond contributes with 7% to the target normal vibration. When an additional imidazole group is coordinated to the iron atom as in **1-25**, the performance score increases marginally to 94 as the contribution from the local vibration of C−Fe bond decreases to 5%.

We have observed that the electronic effects play a critical role in determining the performance score compared to the mass effects. To be more specific, increasing ring strain by reducing the ring size and enhancing the π electron delocalization can lower the score.

### 4.2. Groups 2 and 3: C≡N and S=O Probes

**Figure 6:**
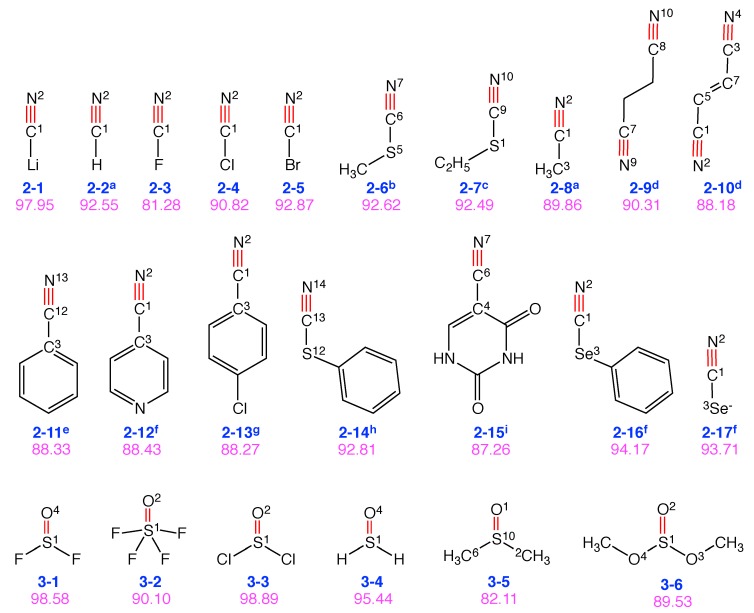
Group 2: 17 VSE probes with C≡N probe bonds in red. Group 3: 6 VSE probes with S=O VSE probe bonds in red. Below each structure, a label as **n-m** is given in blue, **n** denotes the group number (reflecting the type of VSE probe bond), and **m** is the number of this molecule in this group. Vibrational probe labels with superscripts are taken from the literature (a [4,10,50,51], b [10,18,24,27], c [24], d [18,50], e [5,10,18,26,27,50], f [18], g [26,50,51], h [102], i [20]). The corresponding performance score as VSE probe is given in purple. The number in superscript refers to atom index in the molecule. The superscripts are shown only for the atoms whose associated local modes participate in the S=O or C≡N normal mode with more than 5% contribution.

In the following, we will discuss the performance scores for 17 molecules with a C≡N probe bond and 6 molecules with a S=O probe bond shown in Figure 6. Nitriles are one of the most commonly used VSE probes [98,101]. According to the CNM analysis, some S=O probes seem to perform even better.

Lithium cyanide (**2-1**) has a high performance score of 98 with a minor contamination from the C−Li bond stretching. By changing **2-1** to hydrogen cyanide (**2-2**), cyanogen fluoride (**2-3**) and its higher homologues cyanogen chloride (**2-4**) or cyanogen bromide (**2-5**), we observe changes in the performance score ranging from 81 to 93. Cyanogen fluoride is highly contaminated by the C−F bond stretching (19%). The bond stretching contamination of the C≡N probe bond vibration is reduced for the higher halogen homologues. **2-5** and **2-2** have almost the same contamination (7%) from the adjacent C−X bond. This shows that this is predominantly an electronic effect and not a mass effect, given the fact that the masses of H and Br are substantially different, whereas their electronegativities are not too far apart (H: 2.20 and Br: 2.74, Allred Rochow scale). Methyl thiocyanate (**2-6**), ethyl thiocyanate (**2-7**), and phenyl thiocyanate (**2-14**) have quite a comparable performance score of 93. For the phenyl selenocyanate (**2-16**) and the selenocyanate anion (**2-17**), increased performance scores of 94 are found due to Se−C contamination of 6%.

Methylisocyanide (**2-8**) and succinonitrile (**2-9**) have large contamination (10%) from adjacent C−C bond(s) in the target normal mode. The hybridization state of the adjacent carbon atom plays an important role in the performance score. When the adjacent carbon is *sp${}^{2}$* hybridized as in 2-butenedinitrile (**2-10**), there is the involvement of π conjugation which increases the contamination of adjacent C-C bonds to 11%. Similar situation is found in benzonitrile (**2-11**), 4-cyanopyridine (**2-12**), 4-chlorobenzonitrile (**2-13**) and 5-cyanouracil (**2-15**).

Thionyl difluoride (**3-1**) has a highly localized S=O bond with almost negligible contamination from other local modes. The same applies to thionyl dichloride (**3-3**) and thioaldehyde (**3-4**). In dimethyl sulfoxide (**3-5**) and dimethyl sulfite (**3-6**), one important contamination comes from the pyramidalization mode, i.e., movement of S atom in the normal direction of the plane formed by adjacent C−O−C plane (5%) and O−O−O plane (6%), respectively. The two equatorial S−F bonds in thionyl tetrafluoride (**3-2**) lead to significant contamination in the normal mode of S=O bond stretching.

We observe that the C≡N and S=O bonds give rise to many potentially good candidate for VSE probes with high performance scores.

### 4.3. Group 4: Vibrational Probes with Miscellaneous Bonds

**Figure 7:**
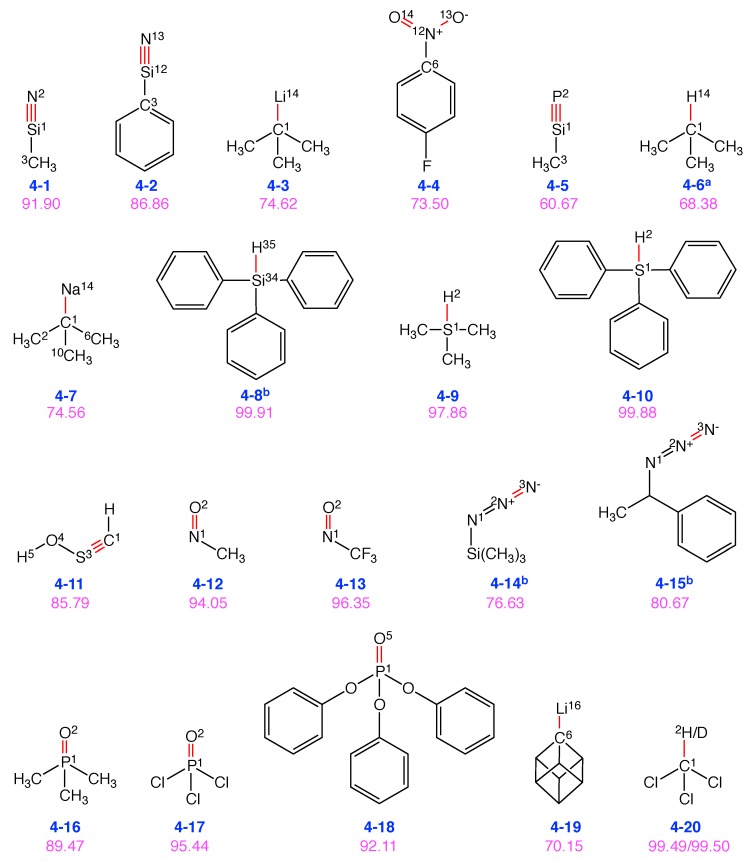
Group 4: 20 VSE probes with miscellaneous probe bonds in red. Below each structure, the label **n-m** is given in blue, **n** denotes the group number (reflecting the type of VSE probe bond), and **m** is the number of this molecule in this group. Vibrational probe labels with superscripts are taken from the literature (a [18]). The corresponding performance score as VSE probe is given in purple. The number in superscript refers to the atom index in the molecule. The superscripts are shown only for the atoms whose associated local modes participate in the normal mode of probe bond stretching with more than 5% contribution.

Besides the C=O, C≡N and S=O probes, other types of chemical bonds have been applied [18] or could be used for VSS as shown in Figure 7.

As demonstrated above, the nitrile group (-C≡N) leads to VSE probes with high performance scores. Therefore, we tested a series of potential VSE candidates with triple bonds. The Si≡N probe bond of methylnitrilosilane (**4-1**) has a satisfactory performance score of 92. The local Si−C stretching vibration contaminates the target Si≡N normal vibration with 7%. The related phenylnitrilosilane (**4-2**) shows a lower performance score of 87 due to larger contamination from the Si−C bond (9%). However, when the nitrogen atom in **4-1** is replaced with phosphorus (**4-5**), the performance score drops to 61. The major contamination comes from the adjascent C−Si bond of 39%. The C≡S triple bond (**4-11**) performs well with a score of 86, with a contamination from the local H−O−S angle bending mode (11%).

Another direction to obtain highly localized normal vibrations is to create a light-heavy situation, i.e., one light atom bonded with a relatively heavy atom [18]. Based on this rationale, we found a few promising probe candidates including trimethyl-$\lambda^{4}$-sulfane (**4-9**), triphenyl-$\lambda^{4}$-sulfane (**4-10**), and triphenylsilane (**4-8**) with performance scores above 98. Similarly, the C−H/C−D bond in chloroform and chloroform-*d* (**4-20**) leads to another high-performance vibrational probe.

We also tested C−Li as a probe bond, tert-butyllithium (**4-3**), and ((2*r*,3*R*,4*s*,5*S*)-cuban-1-yl)lithium (**4-19**). The best performance score of 75 in this series was found for **4-3**. Each of the three adjacent C−C single bonds contaminates the target normal mode. In **4-19**, the lithium atom is connected to a cubane motif. The performance score is only 70 with contamination of the C−Li normal mode by the three adjacent C−C bonds.

If the lithium atom in 4-3 is replaced with sodium leading to tert-butylsodium (**4-7**), the performance score remains the same with contaminations from the three local C−C−C angle bending vibrations. However, if the lithium atom in 4-3 is replaced with hydrogen to isobutane (**4-6**), the performance score decreases.

The nitroso group (-N=O) in nitrosomethane (**4-12**) and trifluoro(nitroso)methane (**4-13**) turns out to be another promising probe bond which renders performance scores above 94. However, the azide functional group (-N=N=N) leads to low performance scores as shown for azidotrimethylsilane (**4-14**) and 1-phenethyl azide (**4-15**) disqualifying N=N as a VSE probe bond. In both molecules, the terminal N=N stretching normal mode is highly contaminated (23% and 19% for **4-14** and **4-15**, respectively) with the adjacent local N=N bond stretching mode.

Given the superior performance scores of N=O probes, we used the P=O bond in a tetrahedral topology to design new probe candidates. In phosphoryl trichloride (**4-17**), the local P=O bond stretching vibration dominates the target normal vibration with a high performance score of 95. When the chlorine atoms in **4-17** are replaced with methyl groups leading to trimethylphosphine oxide (**4-16**), the performance score decreases to 90. The triphenylphosphine oxide (**4-18**) has a similar performance score of 92 although the other three local P−O bond vibrations contaminate the target normal mode.

We observe that the Si≡N bond shows similar characteristics as C≡N bond in terms of the performance score. However, replacing N to P significantly lowers the performance score. Including a combination of low and high atomic mass of atoms for a bond is another way to increase the performance score.

### 4.4. Sensitivity of Performance Score to Density Functional

As the performance score for the probe molecules based on the Characterization of Normal Mode (CNM) method is highly dependent on the quality of the Hessian matrix, and it has been tested that the vibrational frequencies calculated for the same molecule by different density functionals [103] can have differences up to 100 cm${}^{-1}$, one might argue that the performance scores calculated with CNM could be dependent on the employed density functional.

**Figure 8:**
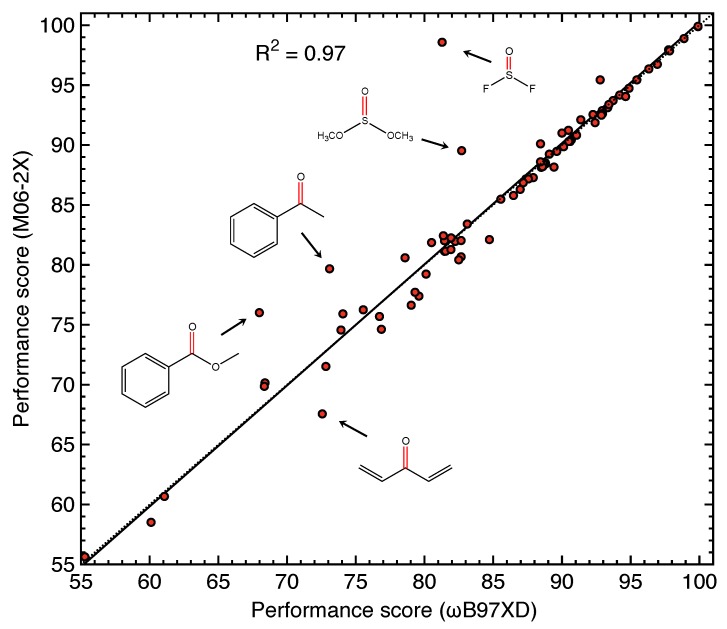
Correlation in the performance scores calculated with M06-2X and ωB97XD for probe molecules studied in this work. The solid line shows a linear correlation with R${}^{2}$ = 0.97, and the dashed line shows the line of y=x.

In order to test the sensitivity of performance score to the selected density functional, we repeated all the calculations including geometry optimization and Hessian evaluation with Truhlar’s Minnesota functional M06-2X, which is believed to perform well for organic compounds [94]. Then, the performance scores calculated by ωB97XD and M06-2X are compared in Figure 8.

Figure 8 shows that the performance scores calculated by M06-2X and ωB97XD are quite consistent, with an average difference of 1.2 for each probe. Probe molecules with relatively high performance scores above 85 are mostly on the line of y=x, with an average difference of 0.5 excluding the outlier thionyl difluoride. Probes with performance scores below 85 have an average difference of 1.7. The large deviations in thionyl difluoride is due to its different geometry when optimized with M06-2X (non-planar) compared to ωB97XD (planar). The other four outliers (dimethyl sulfite, acetophenone, methyl benzoate, and penta-1,4-dien-3-one) can be attributed to the difference in describing the π conjugation in these four molecules with ωB97XD and M06-2X. This result tells us that the performance score obtained in this work is insensitive to the selected density functional employed in calculation. The performance score is especially reliable for the probes when it is relatively high.

### 4.5. Suggestion on Ideal Vibrational Probes

From a systematic evaluation of the polyatomic vibrational Stark effect probes with our CNM method shown above, we pick out the molecules with scores higher than 85 as ideal probes to be recommended to vibrational Stark spectroscopy practitioners. Note that we do not deny that probes with lower performance score can be used as VSE probes; however, choosing well-performed probes will significantly reduce the chances of errors caused by the contamination of other local modes.

In Table 3, we have listed the performance score and calculated frequency for the target normal vibration for each of the ideal probes. As in a real “wet lab” when the vibrational probes are to be put into use, we have to consider the solubility of the probes into a specific solvent and its reactivity; therefore, we try to collect all related information for these probes as a reference for experimentalists.

## 5. Conclusions

In this work, we have employed the characterization of normal mode (CNM) method from Konkoli–Cremer local vibrational mode theory to evaluate more than 68 different vibrational Stark effect probes by quantifying to what extent the probing normal vibrational mode is indeed localized to the local stretching of the probe bond. The quantification was realized by using a performance score in the range of 0∼100 for each vibrational probe for comparison. The probe bonds investigated in this work include C=O/C≡O, C≡N, S=O, Si≡N, C−Li, Si≡P, C−Na, S≡C, N=N^+^=N^-^, S−H, N=O, P=O and C−H. In general, we found probe molecules with double or triple bond tend to score higher than those with single probe bond. However, in several cases of probe molecules with single probe bond (e.g., S−H in a tetrahedral geometry) the performance score can also be high.

We have shown that isotopic substitution for a vibrational probe is a much less efficient approach for obtaining better performed probe candidates compared with tweaking the structure via chemical modification. The validation of the calculated performance score results with different density functionals consolidates the reliability of the CNM method employed in this work.

It is important to note that we did not attempt to explore exhaustively the chemical space by trying all possible combinations of different elements and functional groups for a vibrational probe molecule. However, we have included those representative probes as a guidance for vibrational Stark spectroscopy practitioners. As it has been tested in this work, we can expect that the performance score of a probe molecule will only have a slight change if the chemical modification is distant from the probe bond. Furthermore, we provide an online service (https://vse-server.github.io/) for interested readers to evaluate their vibrational probe candidate molecules with our CNM method.

## Figures and Tables

**Table 1:** Decomposition of target normal mode of probe bond stretching into local modes.

Group 1: C=O and C≡O Probes	
Mol. ${}^{a}$	Local mode contributions ${}^{b}$
**1-1**	88.2% C1-O4, 11.8% H2-C1-H3
**1-2**	83.4% C1-O4, 10.4% (C1-F2, C1-F3), 6.2% F2-C1-F3
**1-3**	93.1% C1-O4
**1-4**	94.7% C1-O4
**1-5**	85.5% C1-O4
**1-6**	76.3% C3-O8, 18.7% (C3-C1, C3-C2)
**1-7**	82.0% C3-O11, 6.9% C2-C3-C4
**1-8**	82.4% C4-O14, 8.3% C3-C4-C5
**1-9**	82.3% C1-O17, 5.2% C6-C1-C2
**1-10**	77.4% C1-O2, 5.3% C3-C1-C7
**1-11**	67.6% C1-O2, 7.0% C4-C1-C7, 5.6% C4-C1, 5.6% C7-C1
**1-12**	77.7% C5-O10, 7.3% C4-C5-C6
**1-13**	79.7% C1-O2, 6.2% C14-C1-C3
**1-14**	79.2% C1-O2, 6.2% C3-C1-C14
**1-15**	89.2% C1-O2
**1-16**	90.5% C1-O2
**1-17**	71.5% C1-O16, 10.7% N11-C1-C4, 7.5% N11-C1
**1-18**	80.6% C1-O2, 6.4% N3-C1, 6.4% N5-C1
**1-19**	91.2% C1-O2
**1-20**	81.9% C5-O6, 6.1% C5-C1, 5.5% O7-C5-C1
**1-21**	76.0% C1-O2, 7.1% C8-C1-O3, 5.7% C8-C1
**1-22**	87.2% C1-O2, 5.3% C1-C6
**1-23**	75.7% C1-O2, 6.9% N7-C1, 6.8% N7-C1-C3
**1-24**	93.3% C1-O2, 6.7% C1-Fe39
**1-25**	94.0% C1-O2
**Group 2: C≡N probes**	
Mol. ${}^{a}$	Local mode contributions ${}^{b}$
**2-1**	98.0% C1-N2
**2-2**	92.6% C1-N2, 7.4% H3-C1
**2-3**	81.3% C1-N2, 18.7% F3-C1
**2-4**	90.8% C1-N2, 9.2% Cl3-C1
**2-5**	92.9% C1-N2, 7.1% Br3-C1
**2-6**	92.6% N7-C6, 7.4% S5-C6
**2-7**	92.5% C9-N10, 7.5% C9-S1
**2-8**	89.9% C1-N2, 10.1% C3-C1
**2-9**	45.1% N9-C7, 45.1% C8-N10
**2-10**	44.1% C1-N2, 44.1% C3-N4, 11.6% (C3-C7, C5-C1)
**2-11**	88.3% C12-N13, 11.3% C12-C3
**2-12**	88.4% N2-C1, 11.3% C3-C1
**2-13**	88.3% C1-N2, 11.5% C1-C3
**2-14**	92.8% C13-N14, 7.2% C13-S12
**2-15**	87.3% N7-C6, 12.5% C4-C6
**2-16**	94.2% N2-C1, 5.8% Se3-C1
**2-17**	93.7% C1-N2, 6.3% C1-Se3
**Group 3: S=O probes**	
Mol. ${}^{a}$	Local mode contributions ${}^{b}$
**3-1**	98.6% S1-O4
**3-2**	90.1% S1-O2
**3-3**	98.9% S1-O2
**3-4**	95.4% S1-O4
**3-5**	82.1% O1-S10, 5.2% pyra (S10-C6-O1-C2)${}^{*}$
**3-6**	89.5% S1-O2, 5.5% pyra (S1-O4-O2-O3)${}^{*}$
**Group 4: Other probes**	
Mol. ${}^{a}$	Local mode contributions ${}^{b}$
**4-1**	91.9% Si1-N2, 6.8% C3-Si1
**4-2**	86.9% Si12-N13, 9.2% Si12-C3
**4-3**	74.6% Li14-C1
**4-4**	36.8% O14-N12, 36.7% O13-N12, 17.5% N12-C6
**4-5**	60.7% Si1-P2, 39.2% Si1-C3
**4-6**	68.4% C1-H14
**4-7**	74.6% C1-Na14, 11.5% (C2-C1-C10, C6-C1-C10), 5.8% C2-C1-C6
**4-8**	99.9% H35-Si34
**4-9**	97.9% H2-S1
**4-10**	99.9% H2-S1
**4-11**	85.8% C1-S3, 10.6% S3-O4-H5
**4-12**	94.0% N1-O2
**4-13**	96.4% N1-O2
**4-14**	76.6% N3-N2, 23.2% N2-N1
**4-15**	80.7% N3-N2, 19.2% N2-N1
**4-16**	89.5% P1-O2
**4-17**	95.4% P1-O2
**4-18**	92.1% P1-O5
**4-19**	70.2% C6-Li16
**4-20**	99.5% C1-H2

*^a^* Column Mol. refers to the molecule label as shown in Figure 4, Figure 6 and Figure 7; *^b^* The decomposition of target normal mode into local modes showing all contributions greater than 5%; * pyra refers to pyramidilization angle. The first atom in the parentheses moves orthogonal to the plane formed by the other three atoms.

**Table 2:** Comparison of the performance scores of molecules X_2_CO, **1-1**–**1-4** using the atomic masses X = H, F, Cl, and Br. Each column represents the same electronic structure with different masses and a row represents same atomic mass with different electronic structures.

Mass of X (amu)	H${}_{2}$CO (1-1)	F${}_{2}$CO (1-2)	Cl${}_{2}$CO (1-3)	Br${}_{2}$CO (1-4)
1.008 [H]	**88.16**	73.35	92.89	94.06
18.998 [F]	88.49	**83.41**	93.06	94.73
35.453 [Cl]	89.04	84.38	**93.12**	94.73
79.904 [Br]	89.36	84.91	93.15	**94.74**

**Table 3:** Summary of ideal vibrational Stark effect probes with their physicochemical properties.

Probe Bond	Label ${}^{a}$	Score	Freq. ${}^{c}$	Solubility/Miscibility ${}^{b}$	Known Limitation ${}^{b}$
C=O	1-1	88.2	1770	water, ethanol, chloroform, ether, acetone, benzene	-
	**1-3**	93.1	1826	benzene, toluene, glacial acetic acid, most liquid hydrocarbons, water	-
	**1-4**	94.7	1829	-	reacts with water
	1-5	85.5	1754	water, benzene, alcohol, dimethylformamide, ether	-
	**1-15**	89.2	1706	organic solvents	-
	**1-16**	90.5	1759	water, acetone, benzene, ethanol, ether	-
	**1-19**	91.2	1919	chloroform	insoluble in water
	**1-22**	87.2	1723	water, alcohol, ether, carbon tetrachloride	-
	1-24	93.3	2145	-	binds to specific proteins
	1-25	94.0	1960	-	binds to specific proteins
C≡N	**2-1**	98.0	2169	water, DMF, THF	-
	2-2	92.5	2136	water, alcohol	-
	**2-5**	92.9	2250	acetonitrile, dicholoromethane, ethanol, ether, benzene, chloroform	reacts slowly with water
	2-6	92.6	2220
	2-9	90.2	2301	acetone, chloroform, dioxane, ehanol, benzene, ether, carbon sulfide	-
	2-14	92.8	2219
	2-16	94.2	2217	THF, dichloromethane, acetonitrile	-
S=O	**3-1**	98.6	1248	-	-
	**3-3**	98.9	1185	-	-
	**3-4**	95.4	988	-	-
	**3-6**	89.5	1158	-	-
Si≡N	**4-1**	91.9	1244	-	-
Si-H	4-8	99.9	2111	methanol	reacts with water
S-H	**4-9**	97.9	1452	-	-
	**4-10**	99.9	2350	-	-
S≡C	**4-11**	85.8	1137	-	-
N=O	**4-12**	94.0	1676	water	-
	**4-13**	96.3	1722	water	-
P=O	**4-16**	89.5	1139	polar organic solvents	-
	**4-17**	95.4	1226	-	reacts with water
	**4-18**	92.1	1250	-	-

${}^{a}$ The bold label refers to novel molecular probes introduced in this work. ${}^{b}$ Physiochemical properties are taken from PubChem database [104] and literature [105]. ${}^{c}$ The vibrational frequencies are computed at M06-2X/aug-cc-pVDZ level of theory and then scaled by an empirical factor of 0.9500 [106].

## Supplementary Materials

The following are available online at https://www.mdpi.com/1424-8220/20/8/2358/s1. The complete list of 107 vibrational Stark effect probe molecules investigated in this work with their performance scores and detailed normal mode decompositions.

## Abbreviations

The following abbreviations are used in this manuscript:

VSE	Vibrational Stark Effect
VSS	Vibrational Stark Spectroscopy
CNM	Characterization of Normal Mode
PED	Potential Energy Distribution
DFT	Density Functional Theory

## References

[1] Stark J. (1913). Observation of the Separation of Spectral Lines by an Electric Field. Nature.

[2] Chattopadhyay A., Boxer S.G. (1995). Vibrational Stark Effect Spectroscopy. J. Am. Chem. Soc..

[3] Bublitz G.U., Boxer S.G. (1997). Stark Spectroscopy: Applications in Chemistry, Biology, and Materials Science. Annu. Rev. Phys. Chem..

[4] Boxer S.G. (2009). Stark Realities. J. Phys. Chem. B.

[5] Fried S.D., Boxer S.G. (2015). Measuring Electric Fields and Noncovalent Interactions Using the Vibrational Stark Effect. Acc. Chem. Res..

[6] Ma J., Pazos I.M., Zhang W., Culik R.M., Gai F. (2015). Site-Specific Infrared Probes of Proteins. Annu. Rev. Phys. Chem..

[7] Fried S.D., Boxer S.G. (2017). Electric Fields and Enzyme Catalysis. Annu. Rev. Biochem..

[8] Adhikary R., Zimmermann J., Romesberg F.E. (2017). Transparent Window Vibrational Probes for the Characterization of Proteins With High Structural and Temporal Resolution. Chem. Rev..

[9] Błasiak B., Londergan C.H., Webb L.J., Cho M. (2017). Vibrational Probes: From Small Molecule Solvatochromism Theory and Experiments to Applications in Complex Systems. Acc. Chem. Res..

[10] Slocum J.D., Webb L.J. (2018). Measuring Electric Fields in Biological Matter Using the Vibrational Stark Effect of Nitrile Probes. Annu. Rev. Phys. Chem..

[11] Welborn V.V., Head-Gordon T. (2018). Computational Design of Synthetic Enzymes. Chem. Rev..

[12] Staffa J.K., Lorenz L., Stolarski M., Murgida D.H., Zebger I., Utesch T., Kozuch J., Hildebrandt P. (2017). Determination of The Local Electric Field at Au/SAM Interfaces Using the Vibrational Stark Effect. J. Phys. Chem. C.

[13] Fried S.D., Bagchi S., Boxer S.G. (2013). Measuring Electrostatic Fields in Both Hydrogen-Bonding and Non-Hydrogen-Bonding Environments Using Carbonyl Vibrational Probes. J. Am. Chem. Soc..

[14] Laberge M., Sharp K.A., Vanderkooi J.M. (1997). Protein Electric Field Effects on the CO Stretch Frequency of Carbonmonoxycytochromes c as a Function of Carbonyl Tilting and Bending Investigated with a Continuum Electrostatic Approach. J. Phys. Chem. B.

[15] Phillips G.N.J., Teodoro M., Li T., Smith B., Gilson M.M., Olson J.S. (1999). Bound CO is A Molecular Probe of Electrostatic Potential in the Distal Pocket of Myoglobin. J. Phys. Chem. B.

[16] Park E.S., Andrews S.S., Hu R.B., Boxer S.G. (1999). Vibrational Stark Spectroscopy in Proteins: A Probe and Calibration for Electrostatic Fields. J. Phys. Chem. B.

[17] Park E.S., Thomas M.R., Boxer S.G. (2000). Vibrational Stark Spectroscopy of NO Bound to Heme: Effects of Protein Electrostatic Fields on the NO Stretch Frequency. J. Am. Chem. Soc..

[18] Suydam I.T., Boxer S.G. (2003). Vibrational Stark Effects Calibrate the Sensitivity of Vibrational Probes for Electric Fields in Proteins. Biochemistry.

[19] Webb L.J., Boxer S.G. (2008). Electrostatic Fields Near the Active Site of Human Aldose Reductase: 1. New Inhibitors and Vibrational Stark Effect Measurements. Biochemistry.

[20] Lindquist B.A., Furse K.E., Corcelli S.A. (2009). Nitrile Groups as Vibrational Probes of Biomolecular Structure and Dynamics: An Overview. Phys. Chem. Chem. Phys..

[21] Walker D.M., Wang R., Webb L.J. (2014). Conserved Electrostatic Fields at the Ras-effector Interface Measured through Vibrational Stark Effect Spectroscopy Explain the Difference in Tilt Angle in the Ras Binding Domains of Raf and RalGDS. Phys. Chem. Chem. Phys..

[22] Fried S.D., Bagchi S., Boxer S.G. (2014). Extreme Electric Fields Power Catalysis in the Active Site of Ketosteroid Isomerase. Science.

[23] Ritchie A.W., Webb L.J. (2015). Understanding and Manipulating Electrostatic Fields at the Protein-Protein Interface Using Vibrational Spectroscopy and Continuum Electrostatics Calculations. J. Phys. Chem. B.

[24] Mohrmann H., Kube I., Lórenz-Fonfría V.A., Engelhard M., Heberle J. (2016). Transient Conformational Changes of Sensory Rhodopsin II Investigated by Vibrational Stark Effect Probes. J. Phys. Chem. B.

[25] Wu Y., Boxer S.G. (2016). A Critical Test of the Electrostatic Contribution to Catalysis with Noncanonical Amino Acids in Ketosteroid Isomerase. J. Am. Chem. Soc..

[26] Schneider S.H., Boxer S.G. (2016). Vibrational Stark Effects of Carbonyl Probes Applied to Reinterpret IR and Raman Data for Enzyme Inhibitors in Terms of Electric Fields at the Active Site. J. Phys. Chem. B.

[27] Deb P., Haldar T., Kashid S.M., Banerjee S., Chakrabarty S., Bagchi S. (2016). Correlating Nitrile IR Frequencies to Local Electrostatics Quantifies Noncovalent Interactions of Peptides and Proteins. J. Phys. Chem. B.

[28] Zoi I., Antoniou D., Schwartz S.D. (2017). Electric Fields and Fast Protein Dynamics in Enzymes. J. Phys. Chem. Lett..

[29] Voller J.S., Biava H., Hildebrandt P., Budisa N. (2017). An Expanded Genetic Code for Probing the Role of Electrostatics in Enzyme Catalysis by Vibrational Stark Spectroscopy. Biochim. Biophys. Acta.

[30] Xu R.J., Blasiak B., Cho M., Layfield J.P., Londergan C.H. (2018). A Direct, Quantitative Connection between Molecular Dynamics Simulations and Vibrational Probe Line Shapes. J. Phys. Chem. Lett..

[31] Welborn V.V., Ruiz Pestana L., Head-Gordon T. (2018). Computational Optimization of Electric Fields for Better Catalysis Design. Nat. Catal..

[32] Biava H., Schreiber T., Katz S., Voeller J.S., Stolarski M., Schulz C., Michael N., Budisa N., Kozuch J., Utesch T. (2018). Long-Range Modulations of Electric Fields in Proteins. J. Phys. Chem. B.

[33] Saggu M., Fried S.D., Boxer S.G. (2019). Local and Global Electric Field Asymmetry in Photosynthetic Reaction Centers. J. Phys. Chem. B.

[34] Silverman L.N., Pitzer M.E., Ankomah P.O., Boxer S.G., Fenlon E.E. (2007). Vibrational Stark Effect Probes for Nucleic Acids. J. Phys. Chem. B.

[35] Watson M.D., Gai X.S., Gillies A.T., Brewer S.H., Fenlon E.E. (2008). A Vibrational Probe for Local Nucleic Acid Environments: 5-Cyano-2′-deoxyuridine. J. Phys. Chem. B.

[36] Zhang S., Shi R., Ma X., Lu L., He Y., Zhang X., Wang Y., Deng Y. (2012). Intrinsic Electric Fields in Ionic Liquids Determined by Vibrational Stark Effect Spectroscopy and Molecular Dynamics Simulation. Chem. Eur. J..

[37] Zhang S., Zhang Y., Deng Y. (2013). Investigation of the Intrinsic Electric Field of Nonhydroxyl and Hydroxyl Ionic Liquids by Vibrational Stark Effect Spectroscopy. RSC Adv..

[38] Hu W., Webb L.J. (2011). Direct Measurement of the Membrane Dipole Field in Bicelles Using Vibrational Stark Effect Spectroscopy. J. Phys. Chem. Lett..

[39] Lambert D.K. (1988). Vibrational Stark Effect of CO on Ni(100), and CO in the Aqueous Double Layer: Experiment, Theory, and Models. J. Chem. Phys..

[40] Lambert D.K. (1996). Vibrational Stark Effect of Adsorbates at Electrochemical Interfaces. Electrochim. Acta.

[41] Schkolnik G., Salewski J., Millo D., Zebger I., Franzen S., Hildebrandt P. (2012). Vibrational Stark Effect of the Electric-Field Reporter 4-Mercaptobenzonitrile as a Tool for Investigating Electrostatics at Electrode/SAM/Solution Interfaces. Int. J. Mol. Sci..

[42] Ge A., Videla P.E., Lee G.L., Rudshteyn B., Song J., Kubiak C.P., Batista V.S., Lian T. (2017). Interfacial Structure and Electric Field Probed by in Situ Electrochemical Vibrational Stark Effect Spectroscopy and Computational Modeling. J. Phys. Chem. C.

[43] Gieseking R.L.M., Lee J., Tallarida N., Apkarian V.A., Schatz G.C., Apkarian A., Schatz G.C. (2018). Bias-Dependent Chemical Enhancement and Nonclassical Stark Effect in Tip-Enhanced Raman Spectromicroscopy of CO-Terminated Ag Tips. J. Phys. Chem. Lett..

[44] Takashima K., Furukawa Y. (2015). Vibrational Stark Effect of 9-Cyanoanthracene Dispersed in a Poly(methyl methacrylate) Film. Chem. Phys. Lett..

[45] Oshiroa M., Takashim K., Furukawa Y. (2019). Infrared Stark Spectra for a Nylon 6 Film. Chem. Phys. Lett..

[46] Lim J.H., Cho D., Kang H., Lee J.Y. (2018). Electronic and Nuclear Contributions to Vibrational Stark Shifts of Hydroxyl Stretching Frequencies of Water Clusters. J. Phys. Chem. C.

[47] Park Y., Lim J.H., Lee J.Y., Kang H. (2019). Electric Field Effect on Condensed-Phase Molecular Systems. VII. Vibrational Stark Sensitivity of Spatially Oriented Water Molecules in an Argon Matrix. J. Phys. Chem. C.

[48] Kang H., Maurais J., Park Y., Ayotte P., Kang H. (2019). Electric Field Effect on Condensed-Phase Molecular Systems. VIII. Vibrational Stark Effect and Dipolar Inversion in a Carbon Monoxide Crystal. J. Phys. Chem. C.

[49] Bishop D.M. (1993). The Vibrational Stark Effect. J. Chem. Phys..

[50] Andrews S.S., Boxer S.G. (2000). Vibrational Stark Effects of Nitriles I. Methods and Experimental Results. J. Phys. Chem. A.

[51] Andrews S.S., Boxer S.G. (2002). Vibrational Stark Effects of Nitriles II. Physical Origins of Stark Effects from Experiment and Perturbation Models. J. Phys. Chem. A.

[52] Wilson E.B., Decius J.C., Cross P.C. (2012). Molecular Vibrations: The Theory of Infrared and Raman Vibrational Spectra.

[53] Woodward L.A. (1972). Introduction to the Theory of Molecular Vibrations and Vibrational Spectroscopy.

[54] Herzberg G. (1991). Molecular Spectra and Molecular Structure. Volume II: Infrared and Raman Spectra of Polyatomic Molecules.

[55] Herzberg G. (2008). Molecular Spectra and Molecular Structure. Volume I: 2nd Edition.

[56] Califano S. (1976). Vibrational States.

[57] Konkoli Z., Cremer D. (1998). A New Way of Analyzing Vibrational Spectra. I. Derivation of Adiabatic Internal Modes. Int. J. Quantum Chem..

[58] Konkoli Z., Larsson J.A., Cremer D. (1998). A New Way of Analyzing Vibrational Spectra. II. Comparison of Internal Mode Frequencies. Int. J. Quantum Chem..

[59] Konkoli Z., Cremer D. (1998). A New Way of Analyzing Vibrational Spectra. III. Characterization of Normal Vibrational Modes in terms of Internal Vibrational Modes. Int. J. Quantum Chem..

[60] Konkoli Z., Larsson J.A., Cremer D. (1998). A New Way of Analyzing Vibrational Spectra. IV. Application and Testing of Adiabatic Modes Within the Concept of the Characterization of Normal Modes. Int. J. Quantum Chem..

[61] Zou W., Kalescky R., Kraka E., Cremer D. (2012). Relating Normal Vibrational Modes to Local Vibrational Modes with the Help of an Adiabatic Connection Scheme. J. Chem. Phys..

[62] Tolman C.A. (1970). Electron Donor-Acceptor Properties of Phosphorus Ligands. Substituent Additivity. J. Am. Chem. Soc..

[63] Tolman C.A. (1972). The 16 In addition, 18 Electron Rule in Organometallic Chemistry and Homogeneous Catalysis. Chem. Soc. Rev..

[64] Tolman C.A. (1977). Steric Effects of Phosphorus Ligands in Organometallic Chemistry and Homogeneous Catalysis. Chem. Rev..

[65] Kalescky R., Kraka E., Cremer D. (2013). New Approach to Tolman’s Electronic Parameter Based on Local Vibrational Modes. Inorg. Chem..

[66] Setiawan D., Kalescky R., Kraka E., Cremer D. (2016). Direct Measure of Metal-Ligand Bonding Replacing the Tolman Electronic Parameter. Inorg. Chem..

[67] Cremer D., Kraka E. (2017). Generalization of the Tolman Electronic Parameter: the Metal-Ligand Electronic Parameter and the Intrinsic Strength of the Metal-Ligand Bond. Dalton Trans..

[68] Kraka E., Cremer D. (2019). Dieter Cremer’s Contribution to the Field of Theoretical Chemistry. Int. J. Quantum Chem..

[69] Kraka E. (2019). Preface: Dieter Cremer’s Scientific Journey. Mol. Phys..

[70] Wilson E.B. (1939). A Method of Obtaining the Expanded Secular Equation for the Vibration Frequencies of A Molecule. J. Chem. Phys..

[71] Tao Y., Tian C., Verma N., Zou W., Wang C., Cremer D., Kraka E. (2018). Recovering Intrinsic Fragmental Vibrations Using the Generalized Subsystem Vibrational Analysis. J. Chem. Theory Comput..

[72] Tao Y. (2018). Advances in Local Vibrational Mode Theory and Unified Reaction Valley Approach (URVA). Ph.D. Thesis.

[73] Tao Y., Zou W., Sethio D., Verma N., Qiu Y., Tian C., Cremer D., Kraka E. (2019). In Situ Measure of Intrinsic Bond Strength in Crystalline Structures: Local Vibrational Mode Theory for Periodic Systems. J. Chem. Theory Comput..

[74] Kalescky R., Kraka E., Cremer D. (2013). Local Vibrational Modes of the Formic Acid Dimer - The Strength of the Double Hydrogen Bond. Mol. Phys..

[75] Cremer D., Larsson J.A., Kraka E. (1998). New Developments in the Analysis of Vibrational Spectra on the use of Adiabatic Internal Vibrational Modes. Theoretical and Computational Chemistry.

[76] Nakanaga T., Kondo S., Saëki S. (1982). Infrared Band Intensities of Formaldehyde and Formaldehyde-d2. J. Chem. Phys..

[77] Morino Y., Kuchitsu K. (1952). A Note on the Classification of Normal Vibrations of Molecules. J. Chem. Phys..

[78] Taylor W.J. (1954). Distribution of Kinetic and Potential Energy in Vibrating Molecules. J. Chem. Phys..

[79] Miyazawa T., Shimanouchi T., Ichiro Mizushima S. (1958). Normal Vibrations of N-Methylacetamide. J. Chem. Phys..

[80] Zerbi G., Overend J., Crawford B. (1963). Urey-Bradley Force Constants of Methanol. J. Chem. Phys..

[81] Keresztury G., Jalsovszky G. (1971). An Alternative Calculation of the Vibrational Potential Energy Distribution. J. Mol. Struct..

[82] Jamróz M.H. (2013). Vibrational Energy Distribution Analysis (VEDA): Scopes and Limitations. Spectrochim. Acta. A.

[83] Brewer S.H., Franzen S. (2003). A Quantitative Theory and Computational Approach for the Vibrational Stark Effect. J. Chem. Phys..

[84] Kraka E., Larsson J.A., Cremer D. (2010). Generalization of the Badger Rule Based on the Use of Adiabatic Vibrational Modes. Computational Spectroscopy.

[85] Frisch M.J., Trucks G.W., Schlegel H.B., Scuseria G.E., Robb M.A., Cheeseman J.R., Scalmani G., Barone V., Petersson A., Nakatsuji H. (2016). Gaussian16 Revision A. 03.

[86] Chai J.D., Head-Gordon M. (2008). Long-Range Corrected Hybrid Density Functionals with Damped Atom-atom Dispersion Corrections. Phys. Chem. Chem. Phys..

[87] Dunning T.H. (1989). Gaussian Basis Sets for use in Correlated Molecular Calculations. I. The Atoms Boron through Neon and Hydrogen. J. Phys. Chem..

[88] Woon D.E., Dunning T.H. (1993). Gaussian Basis Sets for use in Correlated Molecular Calculations. III. The Atoms Aluminum through Argon. J. Phys. Chem..

[89] Wilson A.K., Woon D.E., Peterson K.A., Dunning T.H. (1999). Gaussian Basis Sets for use in Correlated Molecular Calculations. IX. The Atoms Gallium through Krypton. J. Phys. Chem..

[90] Wang Y., Jin X., Yu H.S., Truhlar D.G., He X. (2017). Revised M06-L Functional for Improved Accuracy on Chemical Reaction Barrier Heights, Noncovalent Interactions, and Solid-state Physics. Proc. Natl. Acad. Sci. USA.

[91] Zhao Y., Truhlar D.G. (2006). A New Local Density Functional for Main-Group Thermochemistry, Transition Metal Bonding, Thermochemical Kinetics, and Noncovalent Interactions. J. Chem. Phys..

[92] Weigend F., Ahlrichs R. (2005). Balanced Basis Sets of Split Valence, Triple Zeta Valence and Quadruple Zeta Valence Quality for H to Rn: Design and Assessment of Accuracy. Phys. Chem. Chem. Phys..

[93] Kraka E., Zou W., Filatov M., Tao Y., Grafenstein J., Izotov D., Gauss J., He Y., Wu A., Konkoli Z. (2019). COLOGNE2019. http://www.smu.edu/catco.

[94] Zhao Y., Truhlar D.G. (2008). The M06 Suite of Density Functionals for Main Group Thermochemistry, Thermochemical Kinetics, Noncovalent Interactions, Excited States, and Transition Elements: Two New Functionals and Systematic Testing of four M06-class Functionals and 12 other Functionals. Theor. Chem. Acc..

[95] Park Y., Kang H., Kang H. (2016). Brute Force Orientation of Matrix-Isolated Molecules: Reversible Reorientation of Formaldehyde in An Argon Matrix toward Perfect Alignment. Angew. Chem. Int. Ed. Engl..

[96] Park E.S., Boxer S.G. (2002). Origins of the Sensitivity of Molecular Vibrations to Electric Fields: Carbonyl and Nitrosyl Stretches in Model Compounds and Proteins. J. Phys. Chem. B.

[97] Fried S.D., Wang L.P., Boxer S.G., Ren P., Pande V.S. (2013). Calculations of the Electric Fields in Liquid Solutions. J. Phys. Chem. B.

[98] Dalosto S.D., Vanderkooi J.M., Sharp K.A. (2004). Vibrational Stark Effects on Carbonyl, Nitrile, and Nitrosyl Compounds Including Heme Ligands, CO, CN, and NO, Studied with Density Functional Theory. J. Phys. Chem. B.

[99] McKean D.C. (1978). Individual CH Bond Strengths in Simple Organic Compounds: Effects of Conformation and Substitution. Chem. Soc. Rev..

[100] Lehle H., Kriegl J.M., Nienhaus K., Deng P., Fengler S., Nienhaus G.U. (2005). Probing Electric Fields in Protein Cavities by Using the Vibrational Stark Effect of Carbon Monoxide. Biophys. J..

[101] Reimers J.R., Hush N.S. (1999). Vibrational Stark Spectroscopy 3. Accurate Benchmark ab Initio and Density Functional Calculations for CO and CN^-^. J. Phys. Chem. A.

[102] Okuda M., Higashi M., Ohta K., Saito S., Tominaga K. (2018). Theoretical Investigation on Vibrational Frequency Fluctuations of SCN-Derivatized Vibrational Probe Molecule in Water. Chem. Phys..

[103] El-Azhary A.A., Suter H.U. (1996). Comparison Between Optimized Geometries and Vibrational Frequencies Calculated by the DFT Methods. J. Phys. Chem..

[104] Kim S., Chen J., Cheng T., Gindulyte A., He J., He S., Li Q., Shoemaker B.A., Thiessen P.A., Yu B. (2018). PubChem 2019 Update: Improved Access to Chemical Data. Nucleic Acids Res..

[105] Lide D.R. (1998). Handbook of Chemistry and Physics (87 ed.).

[106] Kashinski D.O., Chase G.M., Nelson R.G., Di Nallo O.E., Scales A.N., VanderLey D.L., Byrd E.F.C. (2017). Harmonic Vibrational Frequencies: Approximate Global Scaling Factors for TPSS, M06, and M11 Functional Families Using Several Common Basis Sets. J. Phys. Chem. A.

