# Supplementary material for: A Critical Evaluation of Vibrational Stark Effect (VSE) Probes with the Local Vibrational Mode Theory
Source: Sensors (Basel). 2020 Apr 21;20(8):2358. doi: 10.3390/s20082358 (PMC7219233; doi:10.3390/s20082358)
Supplement: Supplementary file 1 [file sensors-20-02358-s001.pdf]

A Quantitative Evaluation of Vibrational Stark Effect (VSE) Probes with Local Vibrational Mode Theory

Niraj Verma,[†,§] Yunwen Tao,[†,§] Wenli Zou,[†] Xia Chen,[‡] Xin Chen,[¶] Marek Freindorf,[†] and Elfi Kraka[*,†]

†*Department of Chemistry, Southern Methodist University, 3215 Daniel Avenue, Dallas, Texas 75275-0314, United States*

‡*Hubei Key Laboratory of Natural Medicinal Chemistry and Resource Evaluation, School of Pharmacy, Tongji Medical College, Huazhong University of Science and Technology, Wuhan, Hubei Province 430030, P.R. China*

¶*Laboratory of Theoretical and Computational Chemistry, Institute of Theoretical Chemistry, Jilin University, Changchun, Jilin Province 130023, P. R. China.*

§*Contributed equally to this work*

E-mail: ekraka@gmail.com

Figure 1 to 6 shows all the probes investigated in this work. Followed by, we show the decomposition of all the normal modes into corresponding local modes. The target bond is highlighted with yellow color. For molecules with large number of local modes, we show only the decomposition of target normal mode (all local modes contributing more than 1 % to the target normal mode).

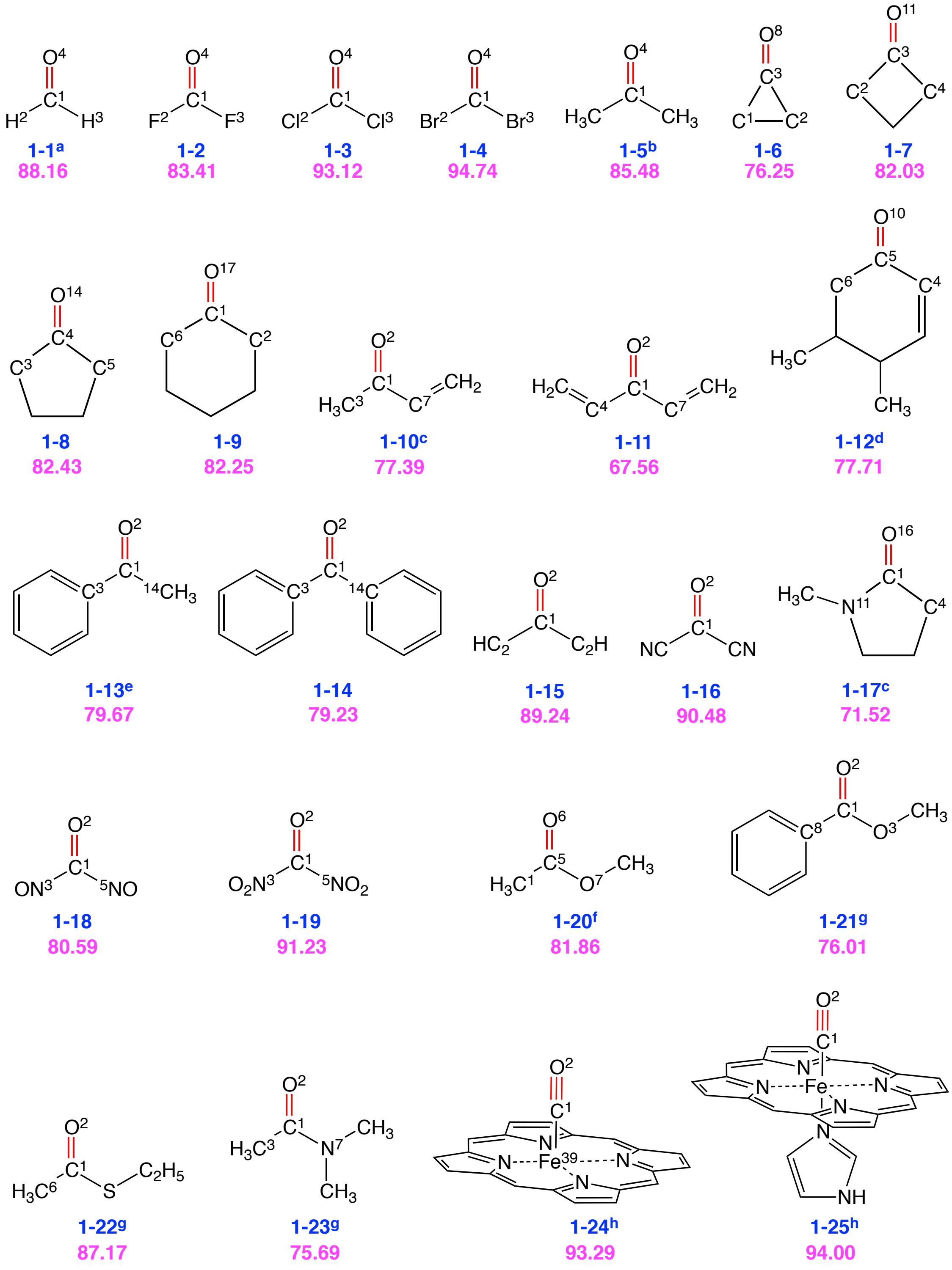

Figure 1: List of different vibrational probes containing the C=O/C≡O bond. Below the 2D structure of each molecule is the label of current compound and its performance score as a vibrational Stark effect probe. Vibrational probes with superscripts on their labels are taken from literature (a,[1] b,[2,3] c,[2] d,[3] e,[3–5] f,[4] g,[4,6] h[7]). The number in brown refers to atom index in the molecule (Note : only those atom indexes are shown which participate in the C=O or C≡O normal mode with more than 5% contribution).

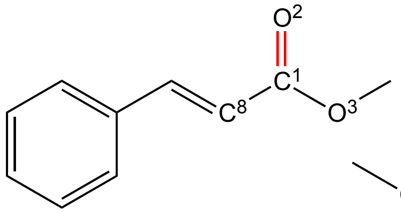

1-26[a]
81.13

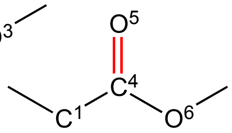

1-27[a]
81.96

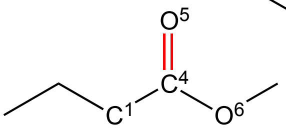

1-28[a]
82.01

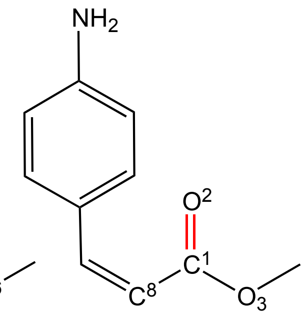

1-29[a]
81.14

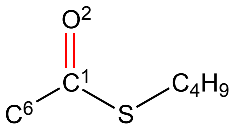

1-30[a]
87.28

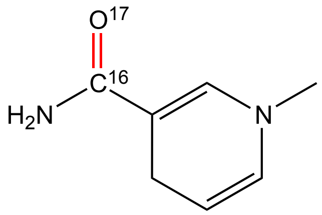

1-31[b]
67.77

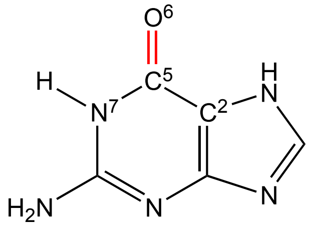

1-32[b]
51.42

Figure 2: List of different vibrational probes containing the C=O/C≡O bond. Below the 2D structure of each molecule is the label of current compound and its performance score as a vibrational Stark effect probe. Vibrational probes with superscripts on their labels are taken from literature (a,[6] b[8]). The number in brown refers to atom index in the molecule (Note : only those atom indexes are shown which participate in the C=O or C≡O normal mode with more than 5% contribution).

Table 1: Decomposition of target normal mode

C=O and C≡O probes	
1-1	88.2% C1-O4, 11.8% H2-C1-H3
1-2	83.4% C1-O2, 10.4% (C1-F3, C1-F4), 6.2% F4-C1-F3
1-3	93.1% C1-O2
1-4	94.7% C1-O2
1-5	85.5% O2-C1
1-6	76.2% O8-C3, 18.7% (C3-C1, C3-C2)
1-7	82.0% O11-C3, 6.9% C2-C3-C4
1-8	82.4% C4-O14, 8.3% C3-C4-C5
1-9	82.2% C1-O17, 5.2% C6-C1-C2
1-10	77.4% O2-C1, 5.3% C3-C1-C7
1-11	67.6% C1-O2, 7.0% C4-C1-C3, 5.6% C4-C6, 5.6% C3-C9
1-12	77.7% C5-O10, 7.3% C4-C5-C6
1-13	79.7% C1-O2, 6.2% C14-C1-C3
1-14	79.2% O2-C1, 6.2% C3-C1-C14
1-15	89.2% C1-O2
1-16	90.5% C1-O2
1-17	71.5% C1-O16, 10.7% N11-C1-C4, 7.5% N11-C1
1-18	80.6% C1-O2, 6.4% N3-C1, 6.4% N5-C1

1-19	91.2% C1-O2
1-20	81.9% O6-C5, 6.1% C5-C1, 5.5% O7-C5-C1
1-21	76.0% O2-C1, 7.1% C8-C1-O3, 5.7% C8-C1
1-22	87.2% C1-O2, 5.3% C1-C6
1-23	75.7% O2-C1, 6.9% N7-C1, 6.8% N7-C1-C3
1-24	93.3% C1-O2, 6.7% C1-Fe39
1-25	94.0% C1-O2
1-26	81.1% O2-C1, 5.6% C8-C1-O3, 5.5% C8-C1
1-27	82.0% O5-C4, 5.7% C4-C1, 5.6% O6-C4-C1
1-28	82.0% O5-C4, 5.7% C4-C1, 5.6% O6-C4-C1
1-29	81.1% O2-C1, 5.6% C8-C1-O3, 5.6% C8-C1
1-30	87.3% O2-C1, 5.3% C6-C1
1-31	67.8% O17-C16, 7.2% C3-C2
1-32	51.4% C5-O6, 16.1% C2-C5, 8.6% C2-C5-N7
C≡N probes	
2-1	98.0% C1-N2
2-2	92.5% C1-N2, 7.5% H3-C1
2-3	81.3% C1-N2, 18.7% F3-C1
2-4	90.8% C1-N2, 9.2% Cl3-C1
2-5	92.9% C1-N2, 7.1% Br3-C1
2-6	92.6% N7-C6, 7.4% S5-C6
2-7	92.5% C9-N10, 7.5% C9-S1
2-8	89.9% C1-N2, 10.1% C3-C1
2-9	45.1% N9-C7, 45.1% C8-N10
2-10	44.1% C1-N2, 44.1% C3-N4, 11.6% (C3-C7, C5-C1)
2-11	88.3% C12-N13, 11.3% C12-C3
2-12	88.4% N2-C1, 11.3% C3-C1
2-13	88.3% C1-N2, 11.5% C1-C3
2-14	92.8% C13-N14, 7.2% C13-S12
2-15	87.1% N7-C6, 12.5% C4-C6
2-16	94.2% N2-C1, 5.8% Se3-C1
2-17	93.7% C1-N2, 6.3% C1-Se3
2-18	90.4% C21-N22, 9.6% C21-C18
2-19	88.5% C23-N24, 11.4% C23-C11
2-20	90.4% N2-C1, 9.5% C3-C1
2-21	90.4% N2-C1, 9.6% C3-C1
2-22	90.4% N2-C1, 9.6% C3-C1
2-23	90.4% N2-C1, 9.6% C3-C1
2-24	88.2% N2-C1, 11.6% C3-C1
2-25	87.2% N7-C6, 12.4% C4-C6
2-26	88.3% N2-C1, 11.4% C3-C1
2-27	88.1% N2-C1, 11.6% C3-C1
2-28	88.4% C1-N2, 11.4% C1-C3
2-29	88.2% N2-C1, 11.6% C3-C1
2-30	45.2% C15-N16, 45.1% C7-N8
2-31	46.1% C25-N26, 42.2% C1-N2, 6.0% C22-C25, 5.4% C1-C3
2-32	44.3% C10-N11, 43.7% C9-N12, 6.0% C10-C3, 5.9% C7-C9
2-33	90.3% C15-N16, 9.6% C12-C15
2-34	86.3% C11-N12, 13.3% C2-C11
S=O probes	
3-1	98.6% S1-O4
3-2	90.1% S1-O2
3-3	98.9% S1-O2
3-4	95.4% S1-O4

3-5	82.1% O1-S10, 5.2% pyra (S10-C6-O1-C2)*
3-6	89.5% S1-O2, 5.5% pyra (S1-O4-O2-O3)*
Other probes	
4-1	91.9% Si1-N2, 6.8% C3-Si1
4-2	86.9% Si12-N13, 9.2% Si12-C3
4-3	74.6% Li14-C1
4-4	36.8% O14-N12, 36.7% O13-N12, 17.5% N12-C6
4-5	60.6% Si1-P2, 39.1% Si1-C3
4-6	69.9% C1-H14
4-7	74.6% C1-Na14, 11.5% (C2-C1-C10, C6-C1-C10), 5.8% C2-C1-C6
4-8	99.9% H35-Si34
4-9	97.9% H2-S1
4-10	99.9% H2-S1
4-11	85.8% C1-S3, 10.6% S3-O4-H5
4-12	94.0% N1-O2
4-13	96.3% N1-O2
4-14	76.6% N3-N2, 23.2% N2-N1
4-15	80.7% N3-N2, 19.2% N2-N1
4-16	89.5% P1-O2
4-17	95.4% P1-O2
4-18	92.1% P1-O5
4-19	70.2% C6-Li16
4-20	99.1% C1-H2
4-21	58.5% C1-Cl14, 6.1% C2-C1-Cl14, 6.1% C6-C1-Cl14
4-22	54.4% N1-Li2, 9.5% N1-C3, 9.5% N1-C13
4-23	55.6% C1-Br14, 7.3% C2-C1-Br14, 7.1% C6-C1-Br14, 5.9% C2-C1-C6, 5.9% C2-C1-C10, 5.7% C6-C1-C10
4-24	56.2% C1-F14, 6.5% C1-C10, 6.3% C1-C2, 6.2% C1-C6
4-25	47.7% F12-C3, 16.8% (H10-C5-C4, H7-C1-C2), 8.5% C2-C3-C4, 6.8% C2-C1, 6.8% C5-C4
4-26	43.4% F9-C3, 10.7% C2-C1, 8.4% C4-C3-C2, 8.3% C4-C3, 7.0% H7-C2-C1
4-27	52.2% O3-C2, 46.1% C2-N1
4-28	42.8% C1-F2, 8.6% C1-C7-H10, 8.6% C1-C3-H4, 7.3% C1-C7, 7.3% C1-C3, 6.9% H8-C7-H10, 6.9% H4-C3-H5, 5.9% C3-C1-C7
4-29	51.2% O3-C2, 46.9% C2-N1
4-30	43.1% C3-F11, 7.0% C1-C2, 7.0% C4-C5, 6.7% C4-C3-C2, 6.2% C4-C5-H10, 6.2% C2-C1-H7
4-31	47.1% F11-C3, 7.9% C2-C1, 7.9% C5-C4, 7.2% C4-C3-C2, 5.9% H10-C5-C4, 5.9% H7-C1-C2
4-32	47.9% F11-C3, 7.5% C2-C1, 7.4% C5-C4, 7.1% C4-C3-C2
4-33	80.4% N3-N2, 19.4% N2-N1
4-34	67.1% C13-D14, 31.1% C13-C12
4-35	49.2% Li12-C4, 26.8% C5-C4-C3, 14.3% (C4-C5-C6, C4-C3-C2)

*pyra refers to pyramidilization angle. The first atom in the paranthesis moves orthogonal to the plane formed by the other three atoms.

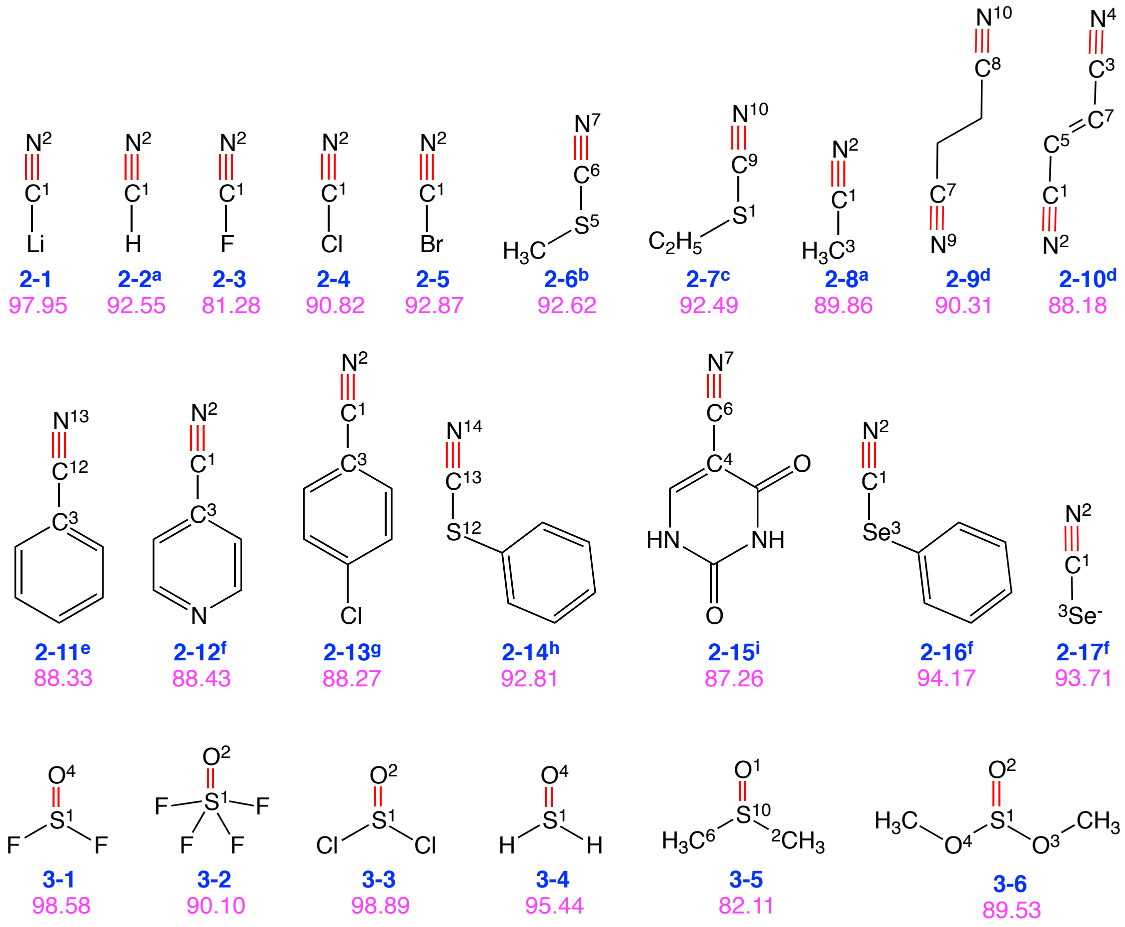

Figure 3: List of different vibrational probes containing the C≡N and S=O bond. Below the 2D structure of each molecule is the label of current compound and its performance score as a vibrational Stark effect probe. Vibrational probes with superscripts on their labels are taken from literature (a,[9] b,[10,11] c,[10] d,[9,11] e,[3,6,9,11] f,[11] g,[6,9] h,[12] i[13]). The number in brown refers to atom index in the molecule (Note : only those atom indexes are shown which participate in the S=O or C≡N normal mode with more than 5% contribution).

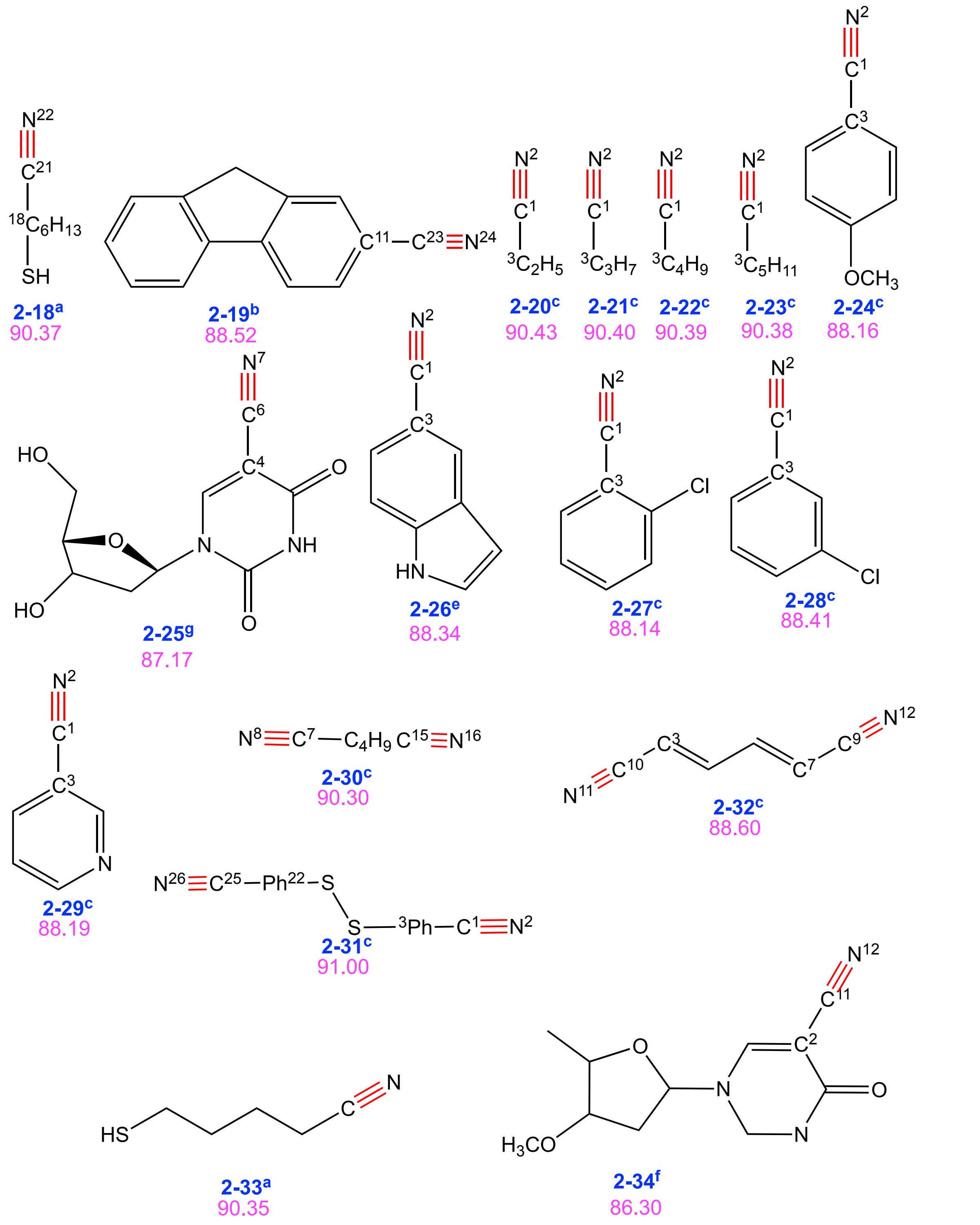

Figure 4: List of different vibrational probes containing the C≡N and S=O bond. Below the 2D structure of each molecule is the label of current compound and its performance score as a vibrational Stark effect probe. Vibrational probes with superscripts on their labels are taken from literature (a,[14] b,[15] c,[16] d,[17] e,[11] f,[18] g[13]). The number in brown refers to atom index in the molecule (Note : only those atom indexes are shown which participate in the C≡N normal mode with more than 5% contribution).

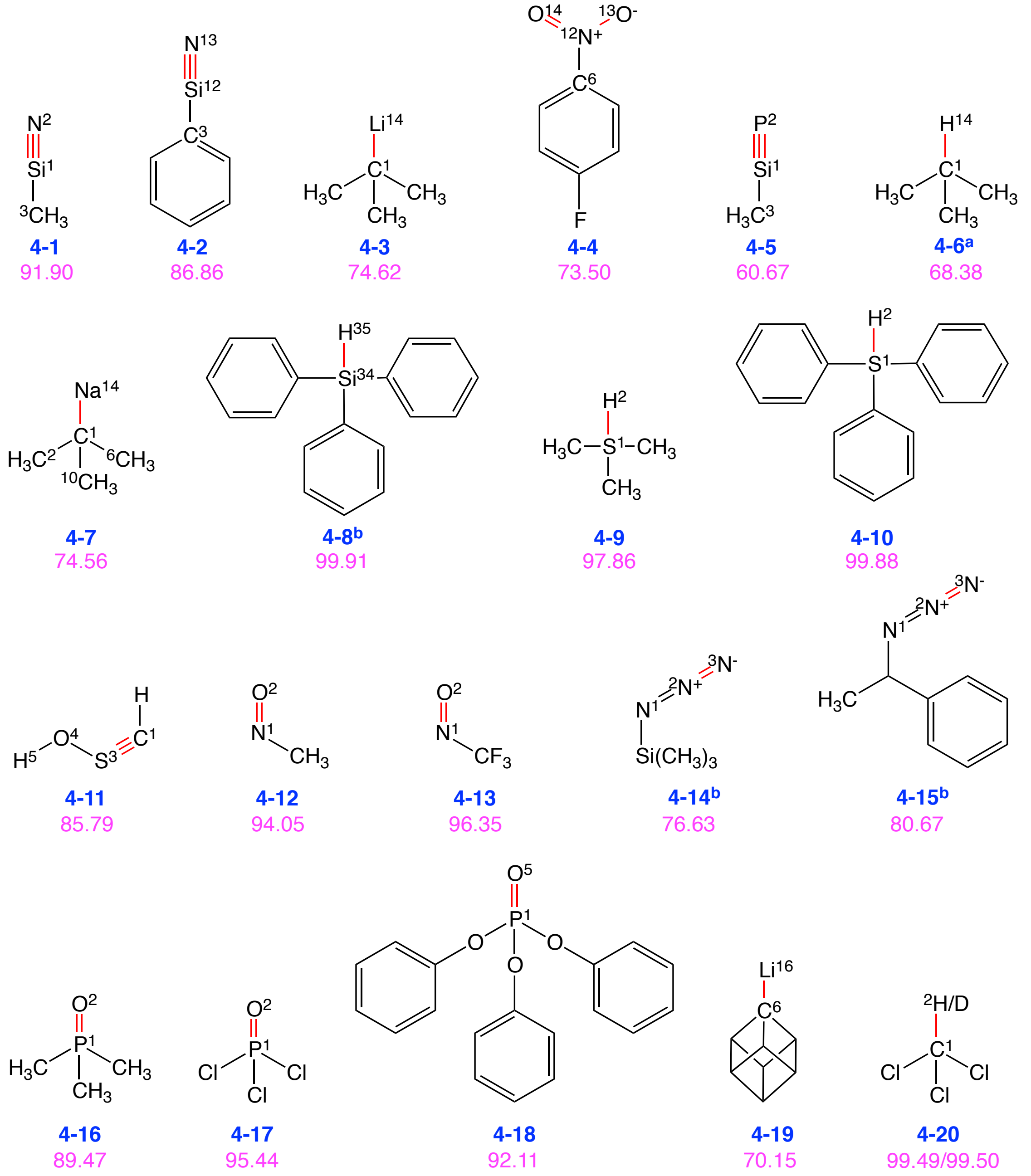

Figure 5: List of different vibrational probes with miscellaneous probe bonds. Below the 2D structure of each molecule is the label of current compound and its performance score as a vibrational Stark effect probe. Vibrational probes with superscripts on their labels are taken from literature (a,[19] b[11]). The number in brown refers to atom index in the molecule (Note : only those atom indexes are shown which participate in the target normal mode with more than 5% contribution).

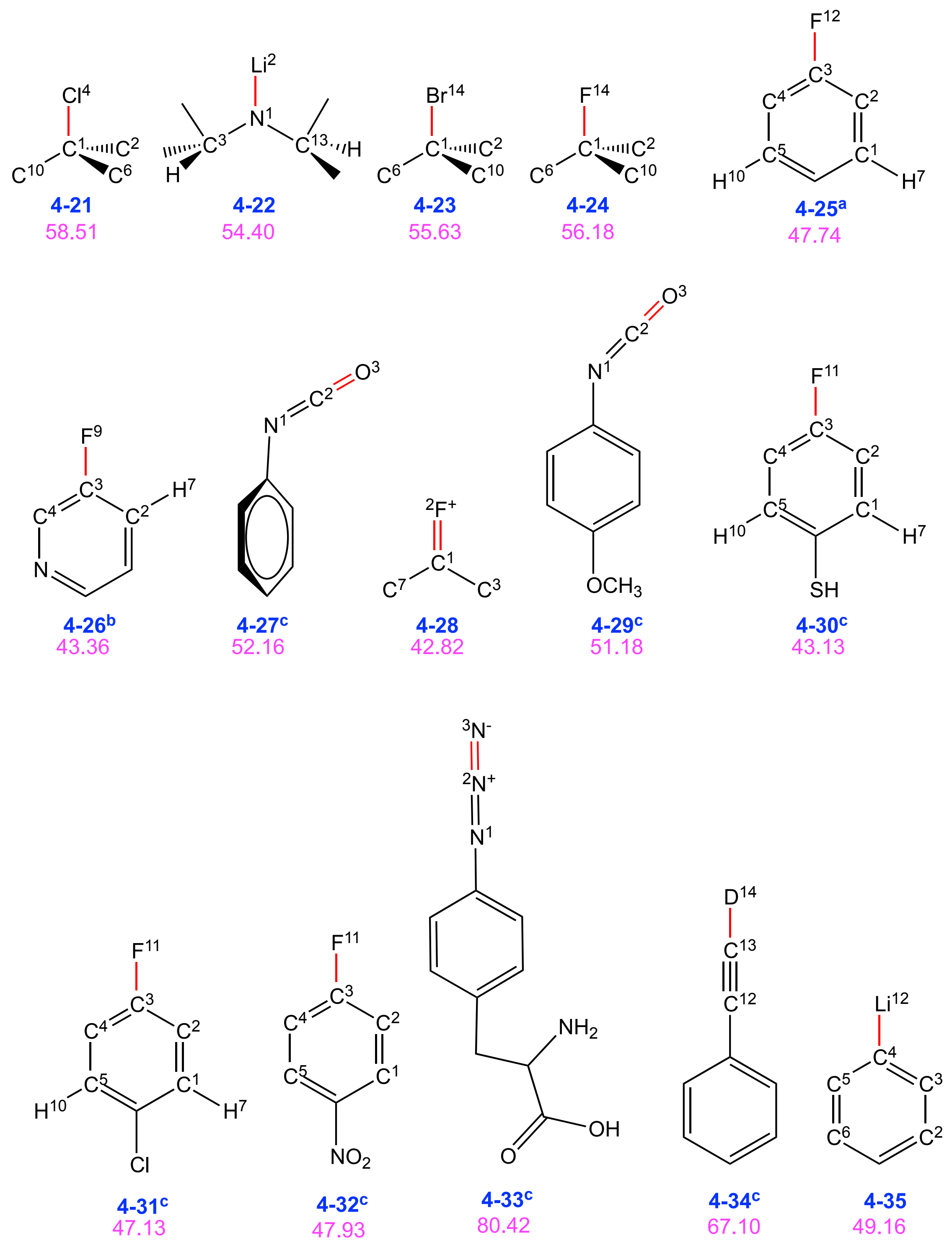

Figure 6: List of 19 different vibrational probes with miscellaneous probe bonds. Below the 2D structure of each molecule is the label of current compound and its performance score as a vibrational Stark effect probe. Vibrational probes with superscripts on their labels are taken from literature (a,[11,19] b,[19] c[11]). The number in brown refers to atom index in the molecule (Note : only those atom indexes are shown which participate in the target normal mode with more than 5% contribution).

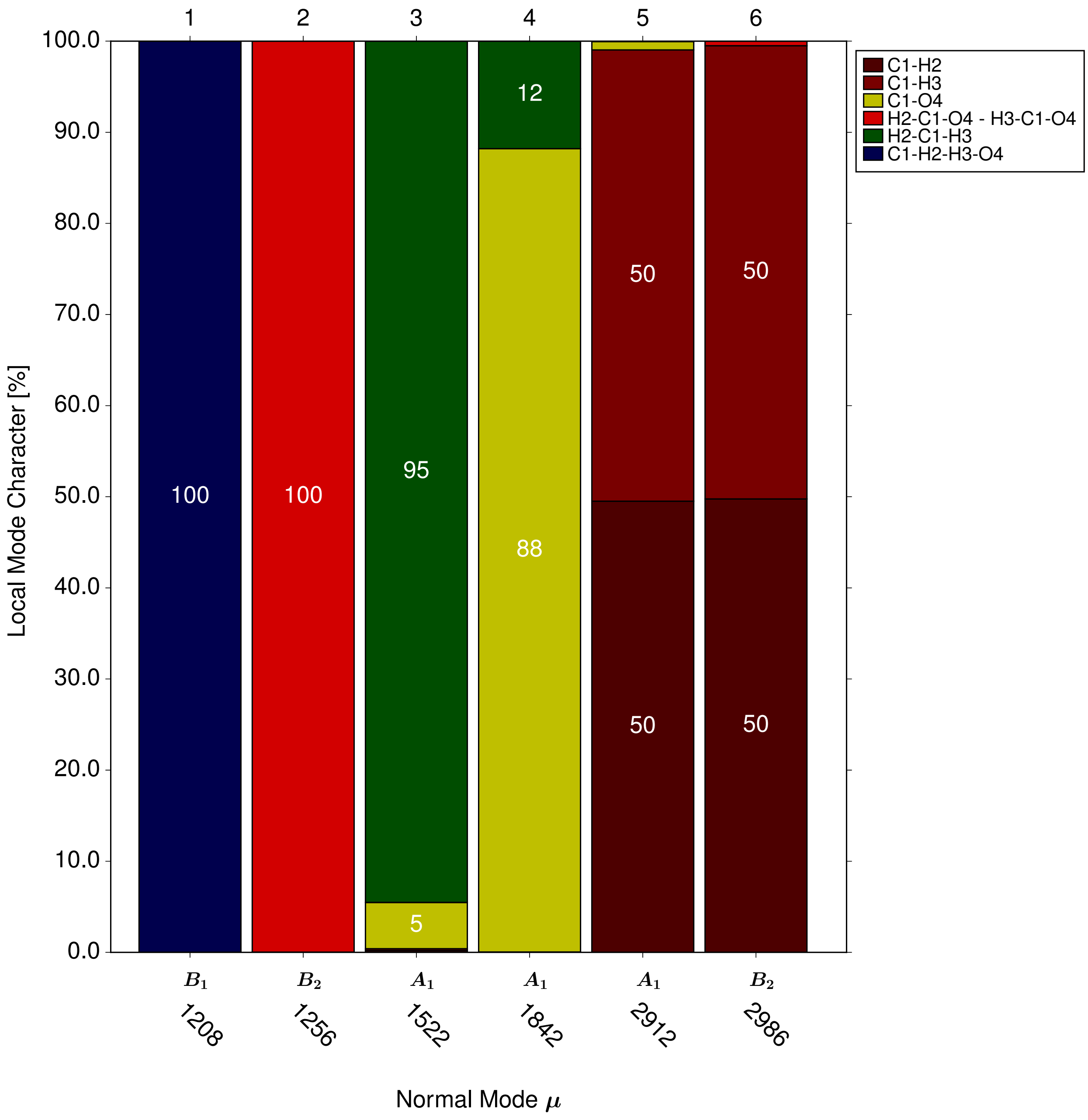

Figure 7: Decomposition of normal mode frequencies for 1-1

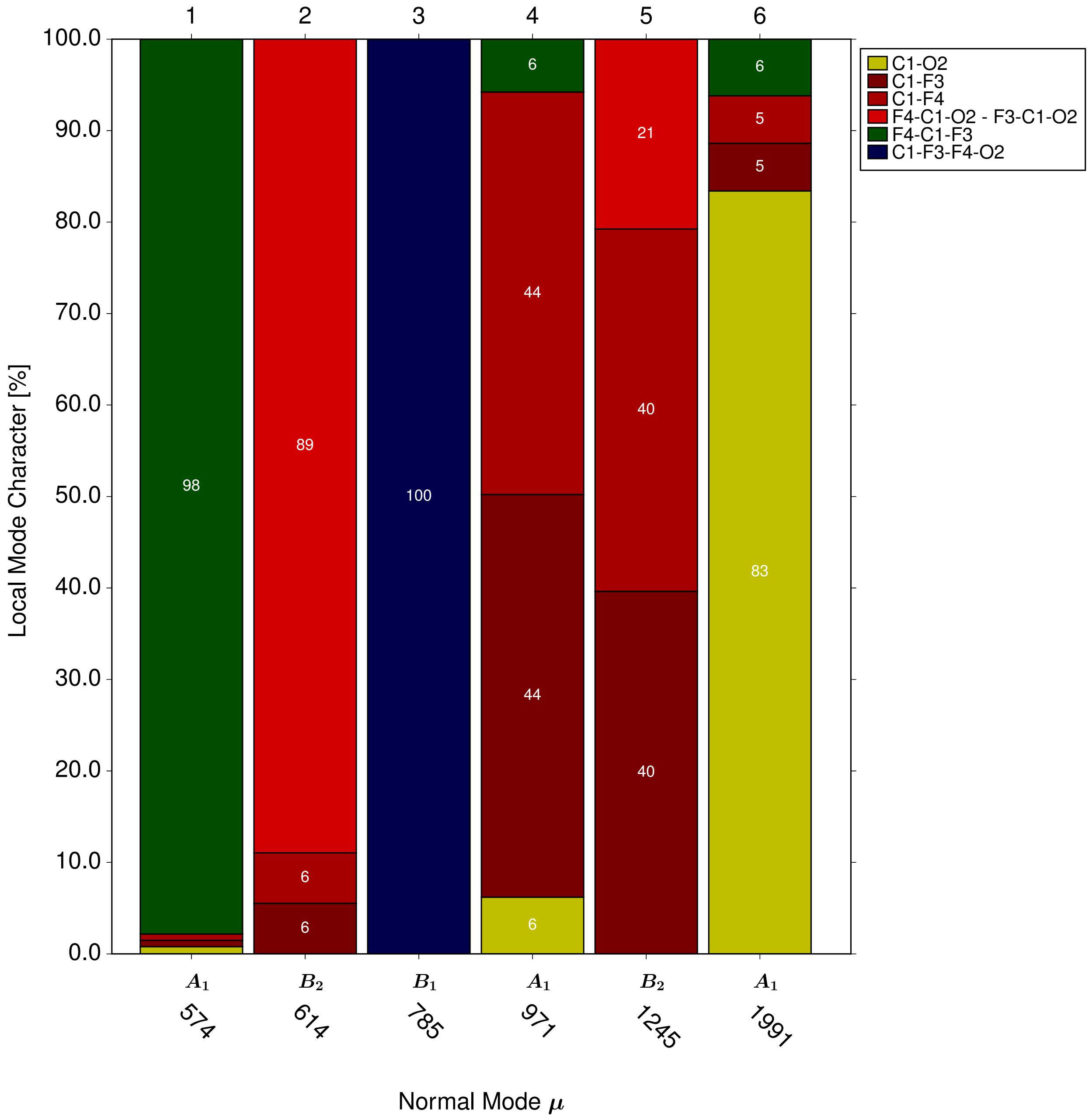

Figure 8: Decomposition of normal mode frequencies for 1-2

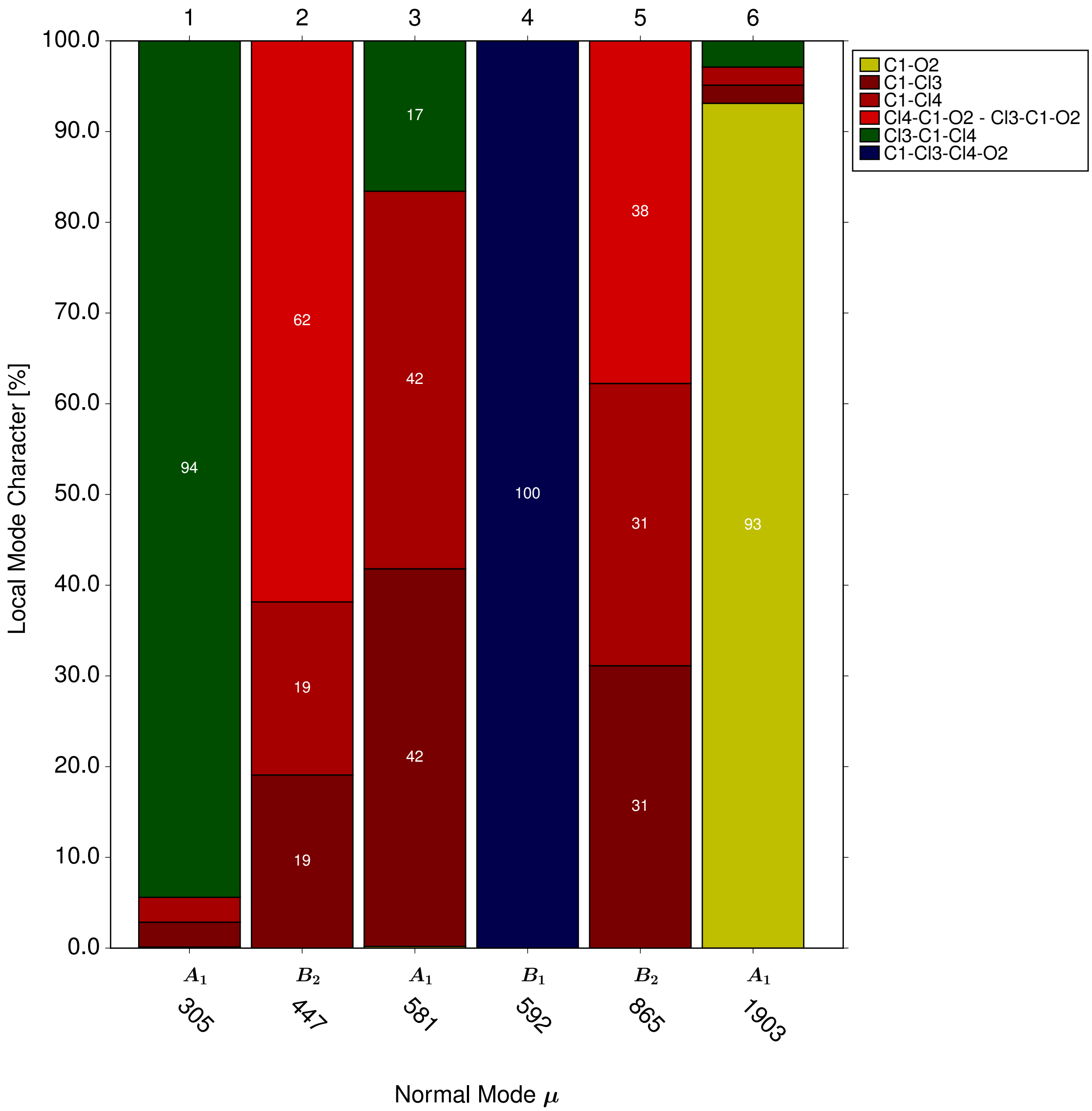

Figure 9: Decomposition of normal mode frequencies for 1-3

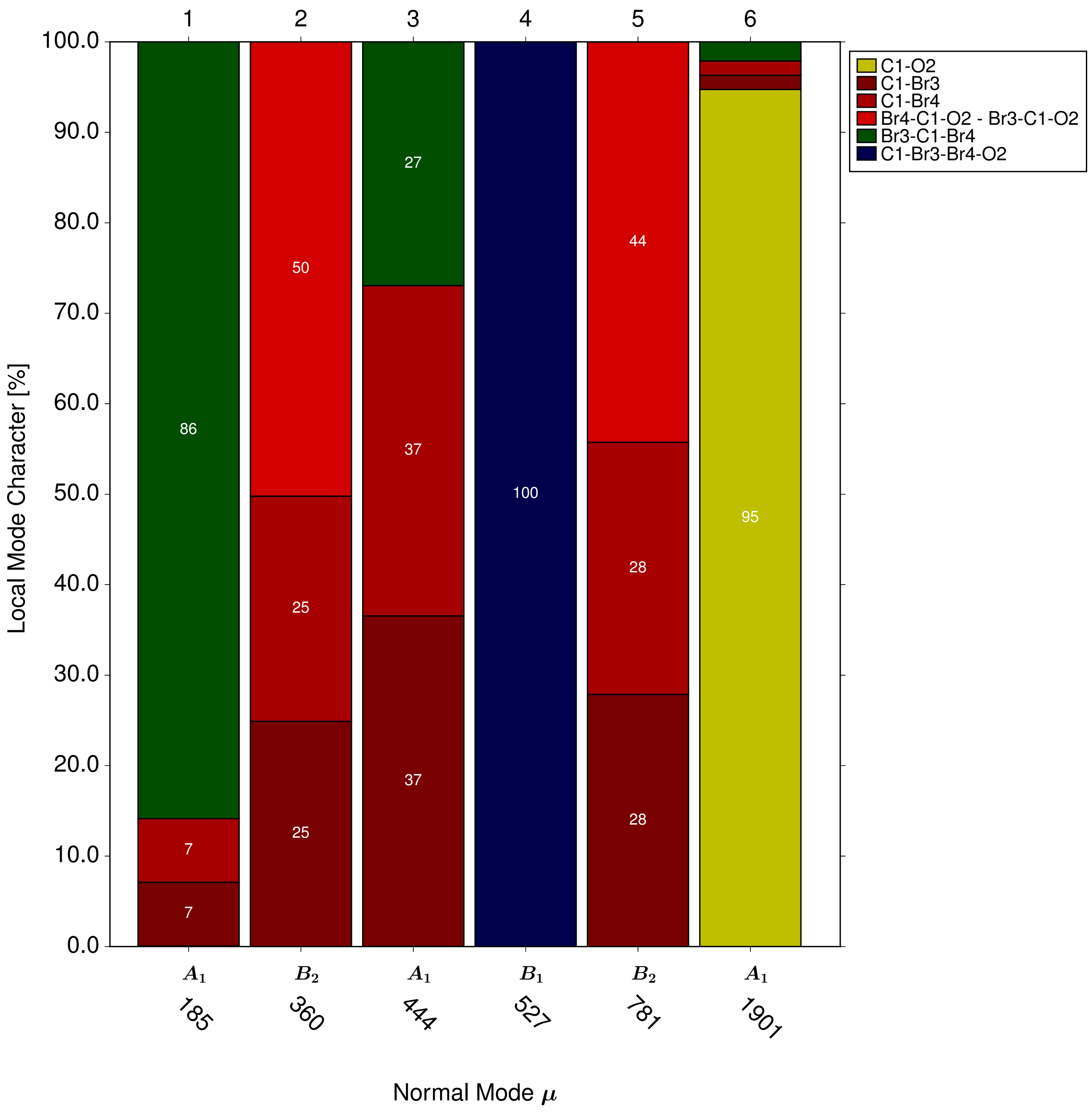

Figure 10: Decomposition of normal mode frequencies for 1-4

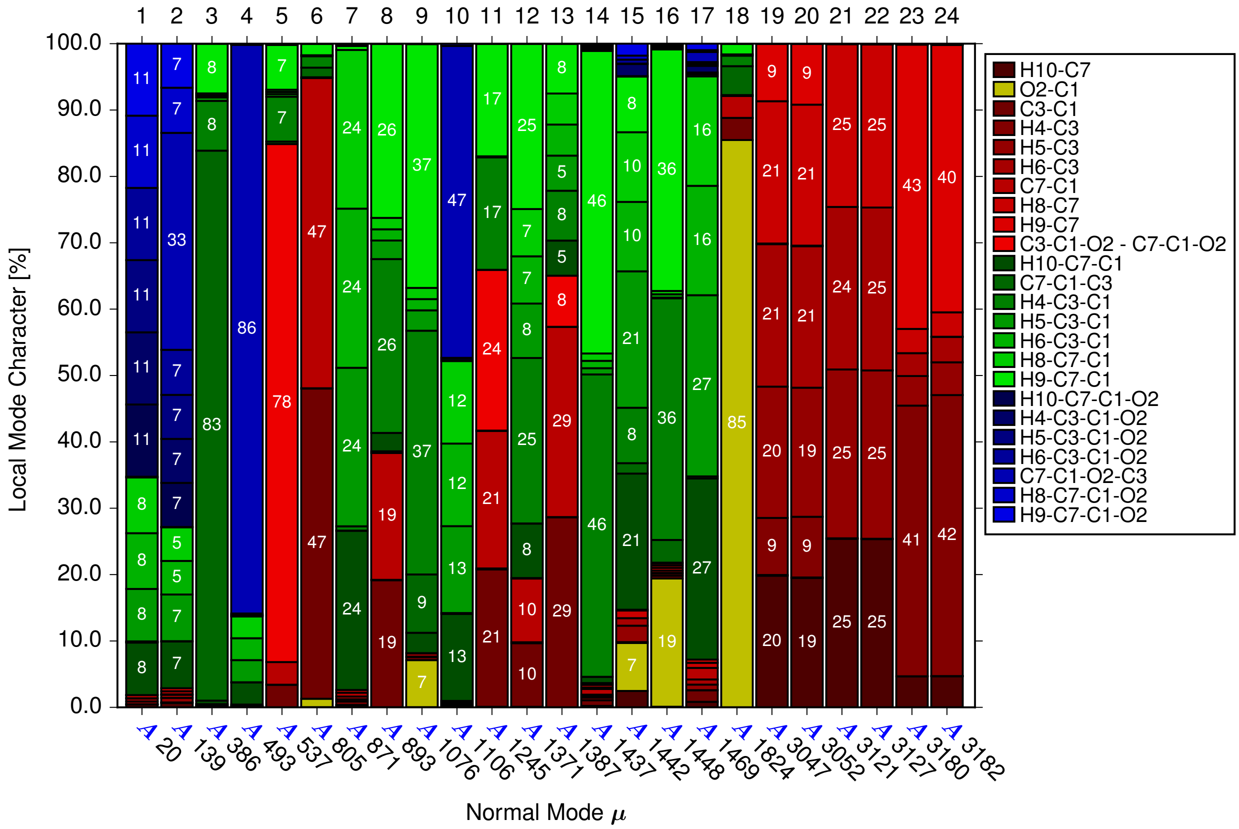

Figure 11: Decomposition of normal mode frequencies for 1-5

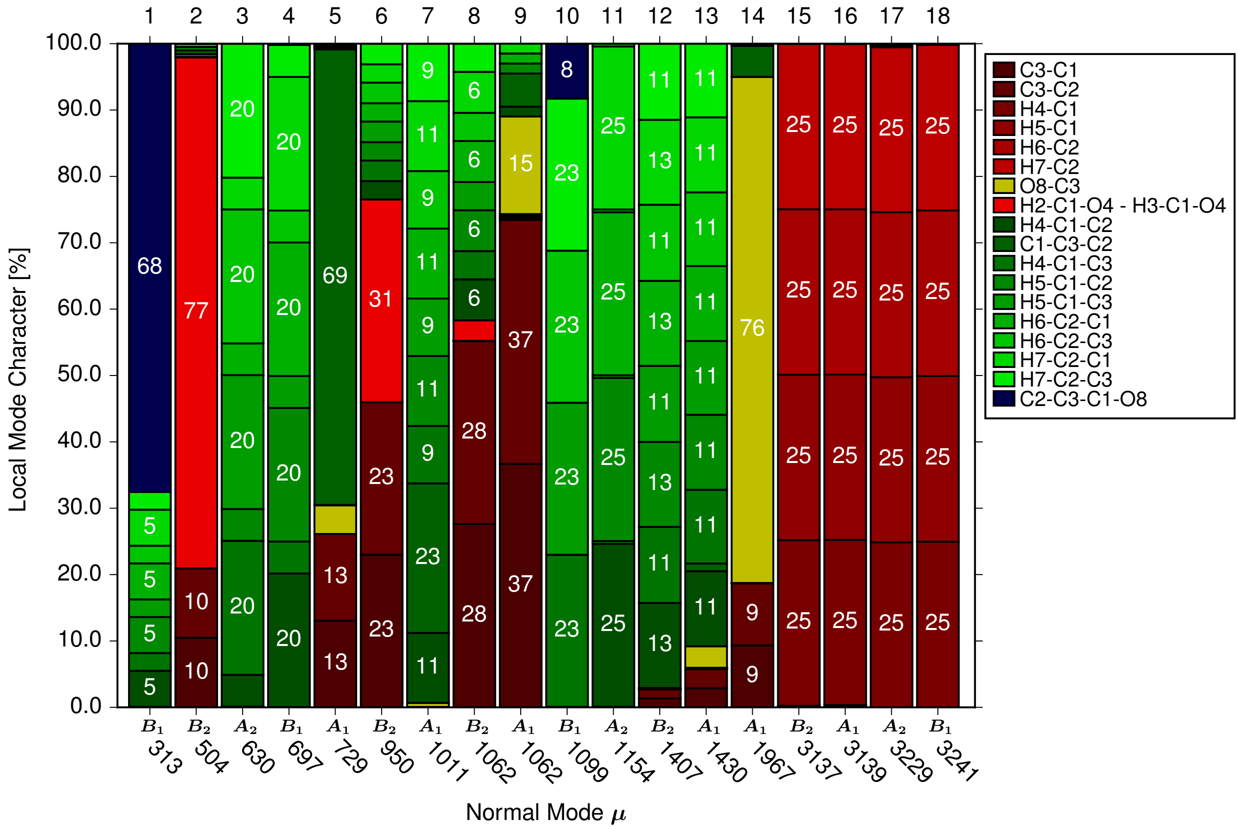

Figure 12: Decomposition of normal mode frequencies for 1-6

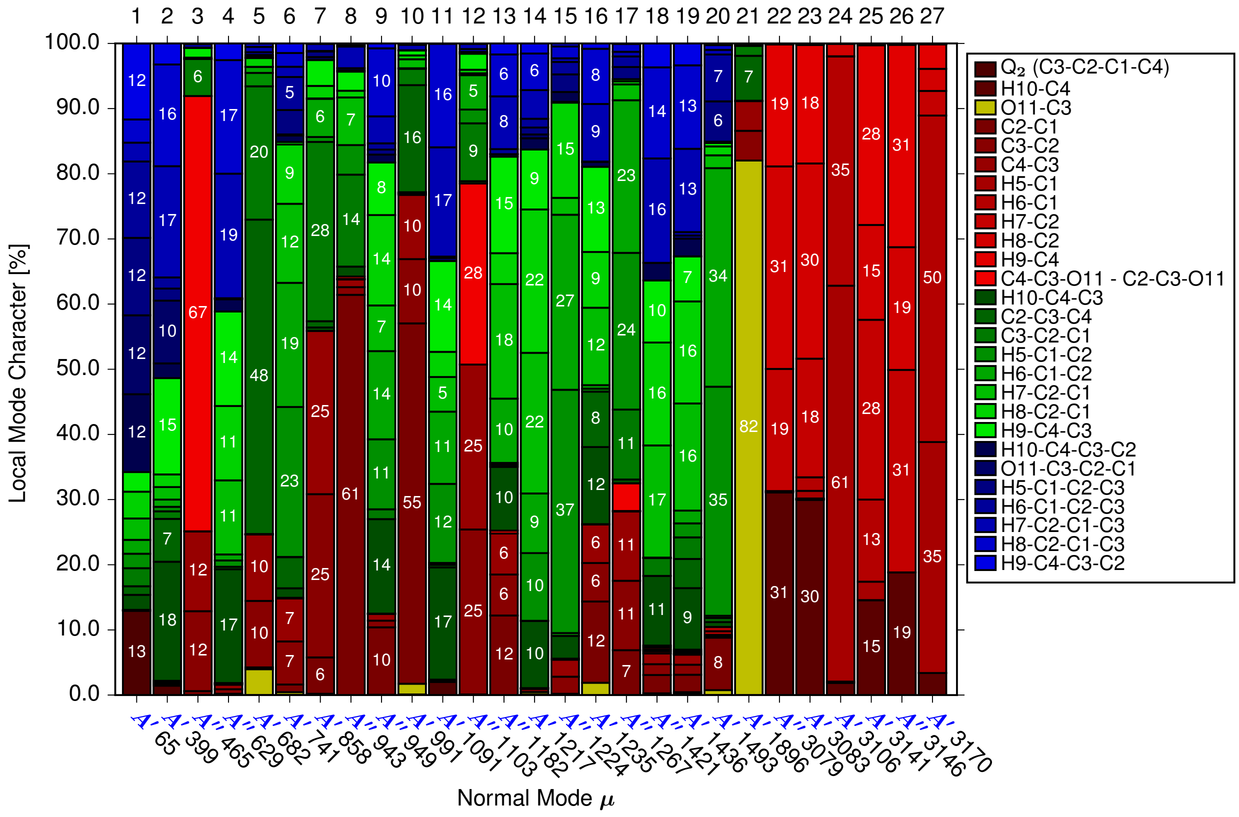

Figure 13: Decomposition of normal mode frequencies for 1-7

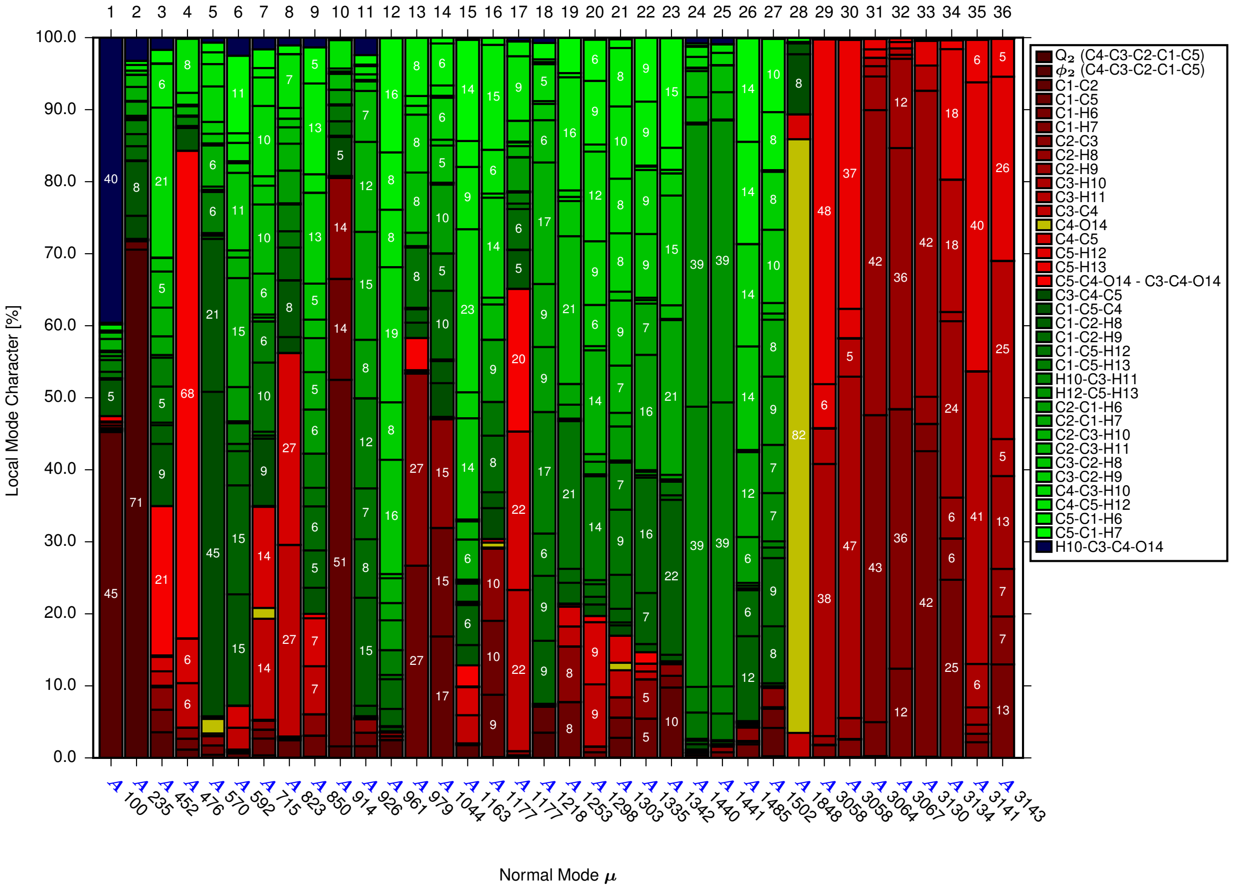

Figure 14: Decomposition of normal mode frequencies for 1-8

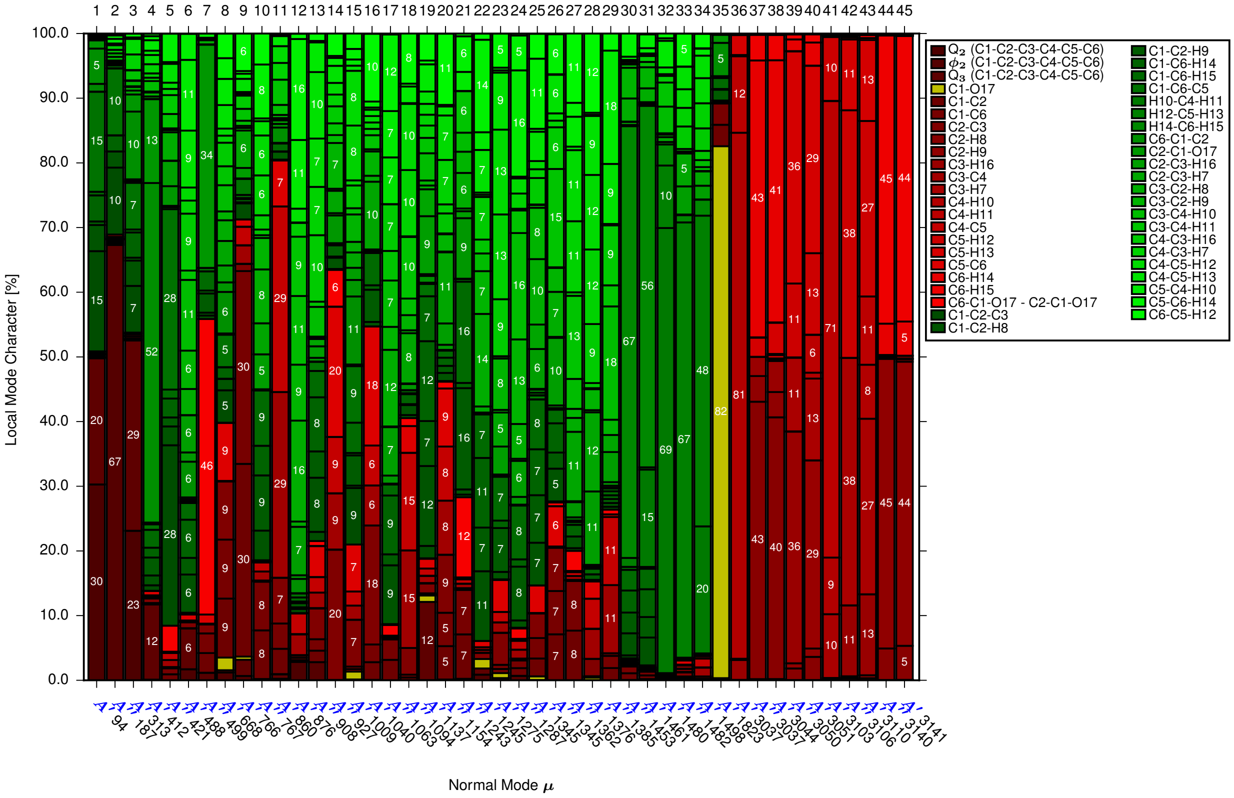

Figure 15: Decomposition of normal mode frequencies for 1-9

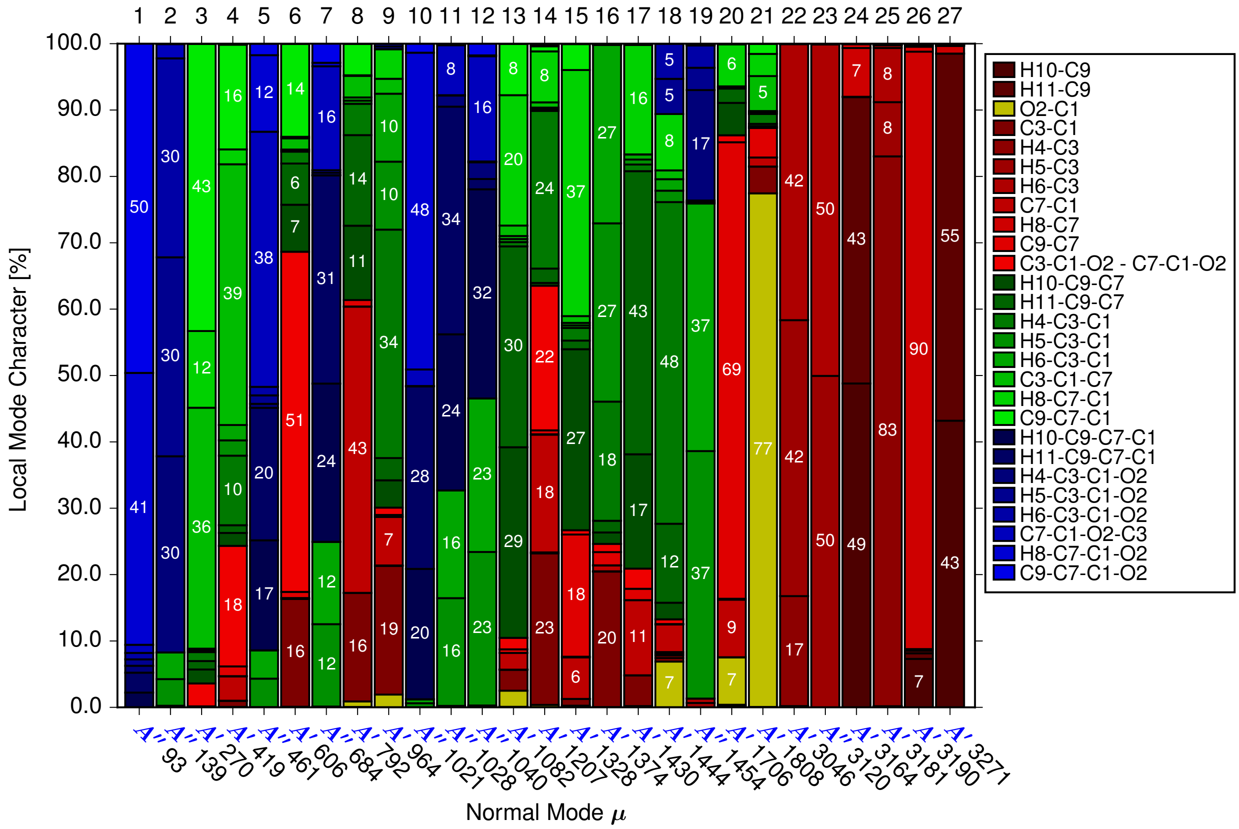

Figure 16: Decomposition of normal mode frequencies for 1-10

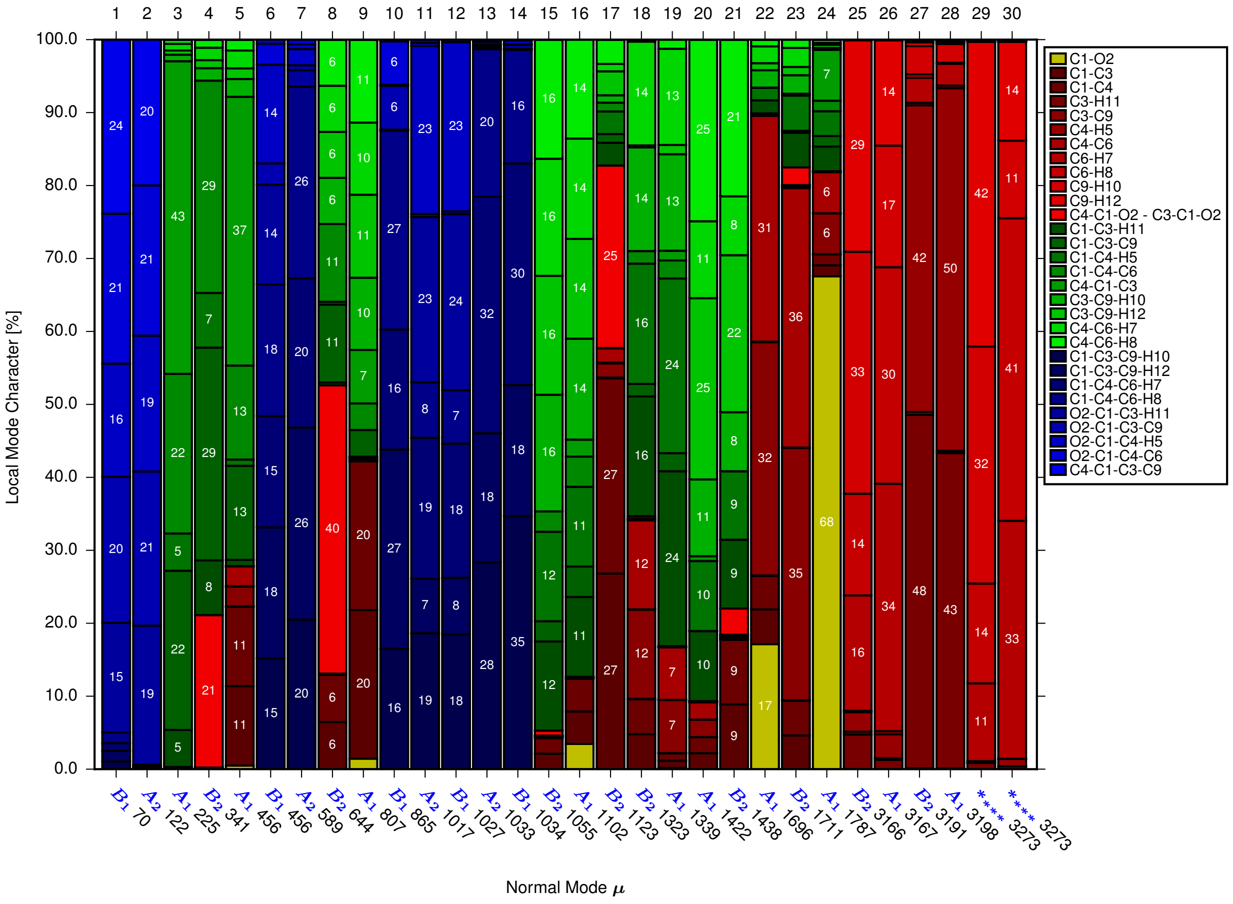

Figure 17: Decomposition of normal mode frequencies for 1-11

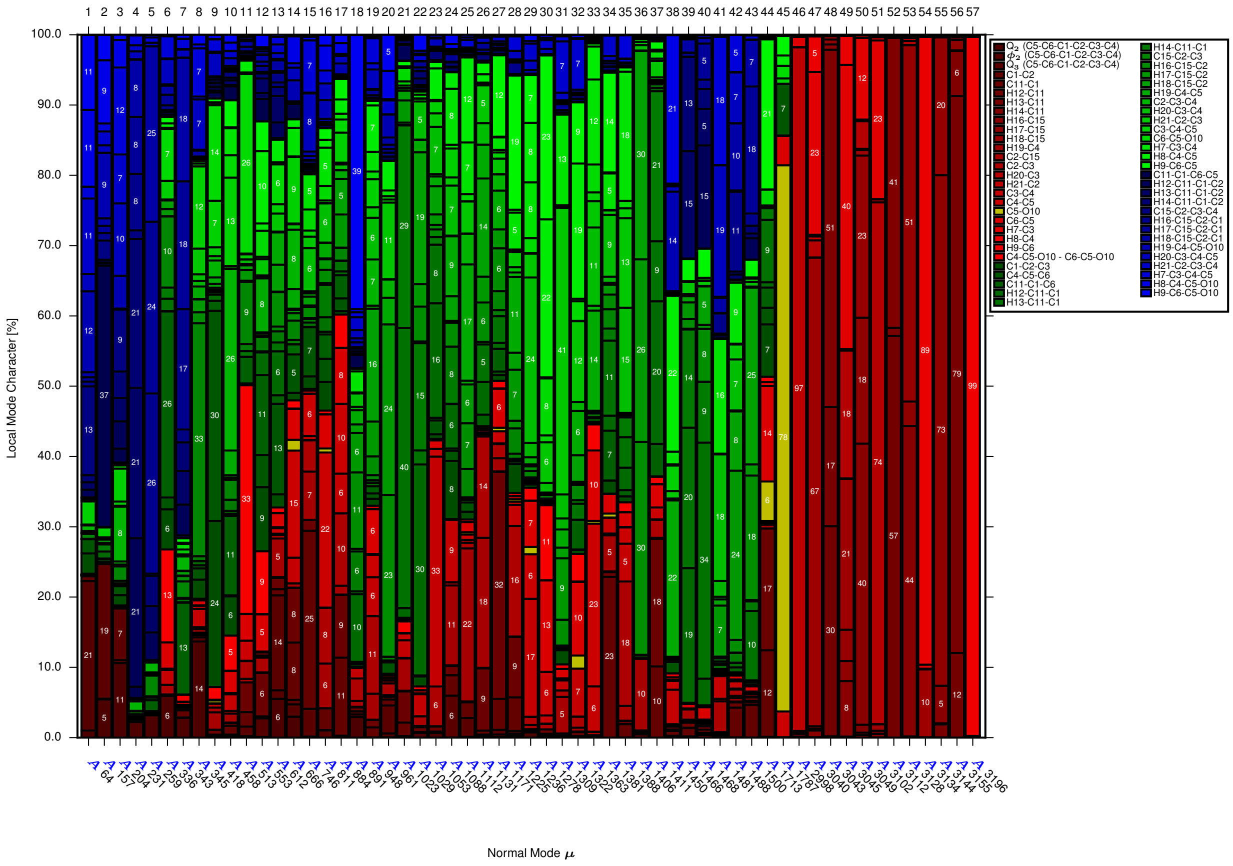

Figure 18: Decomposition of normal mode frequencies for 1-12

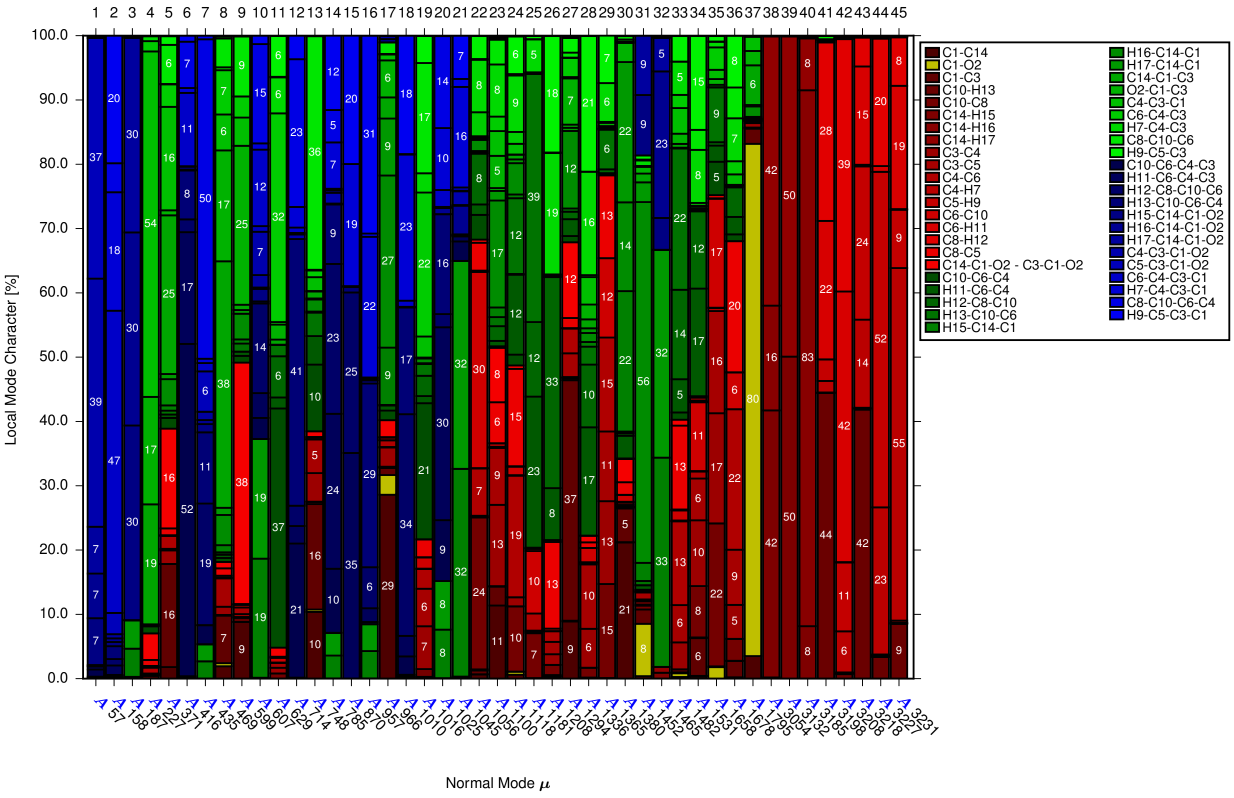

Figure 19: Decomposition of normal mode frequencies for 1-13

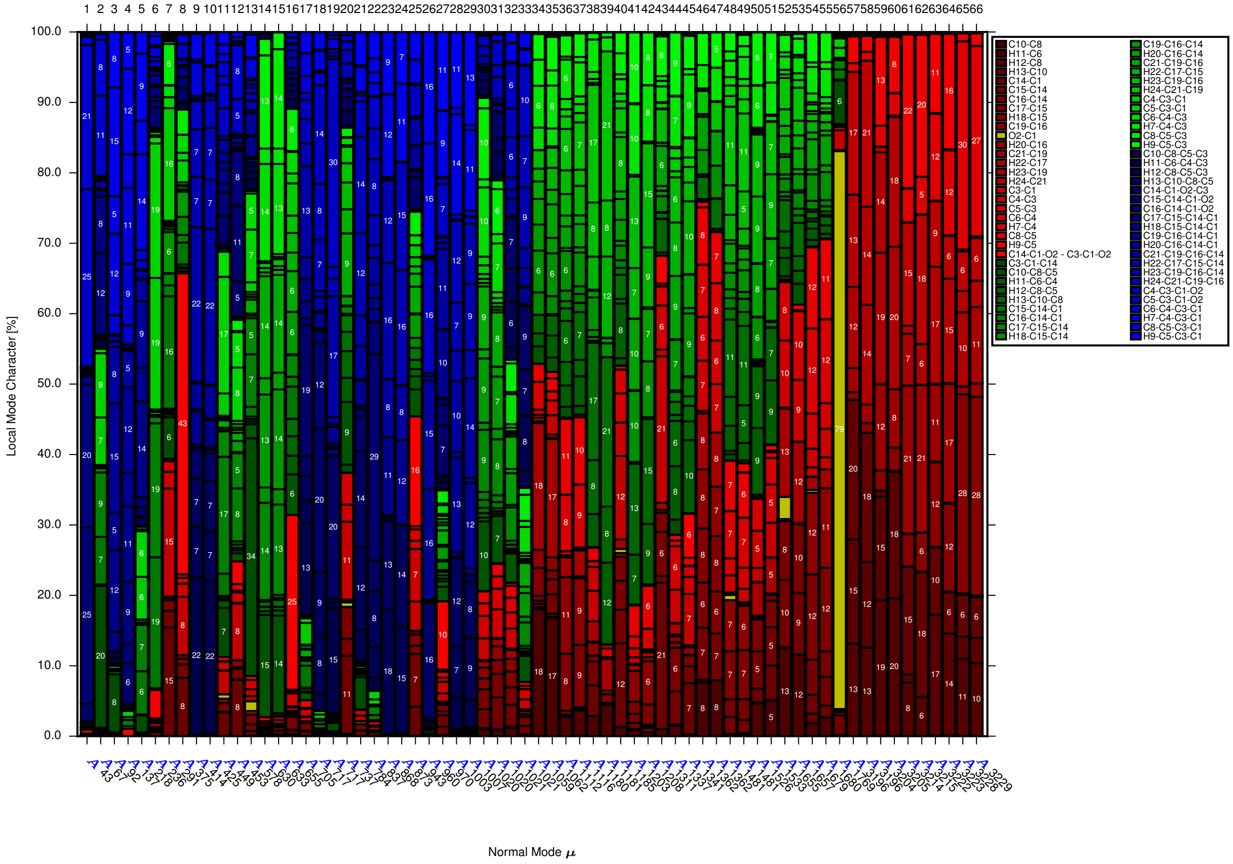

Figure 20: Decomposition of normal mode frequencies for 1-14

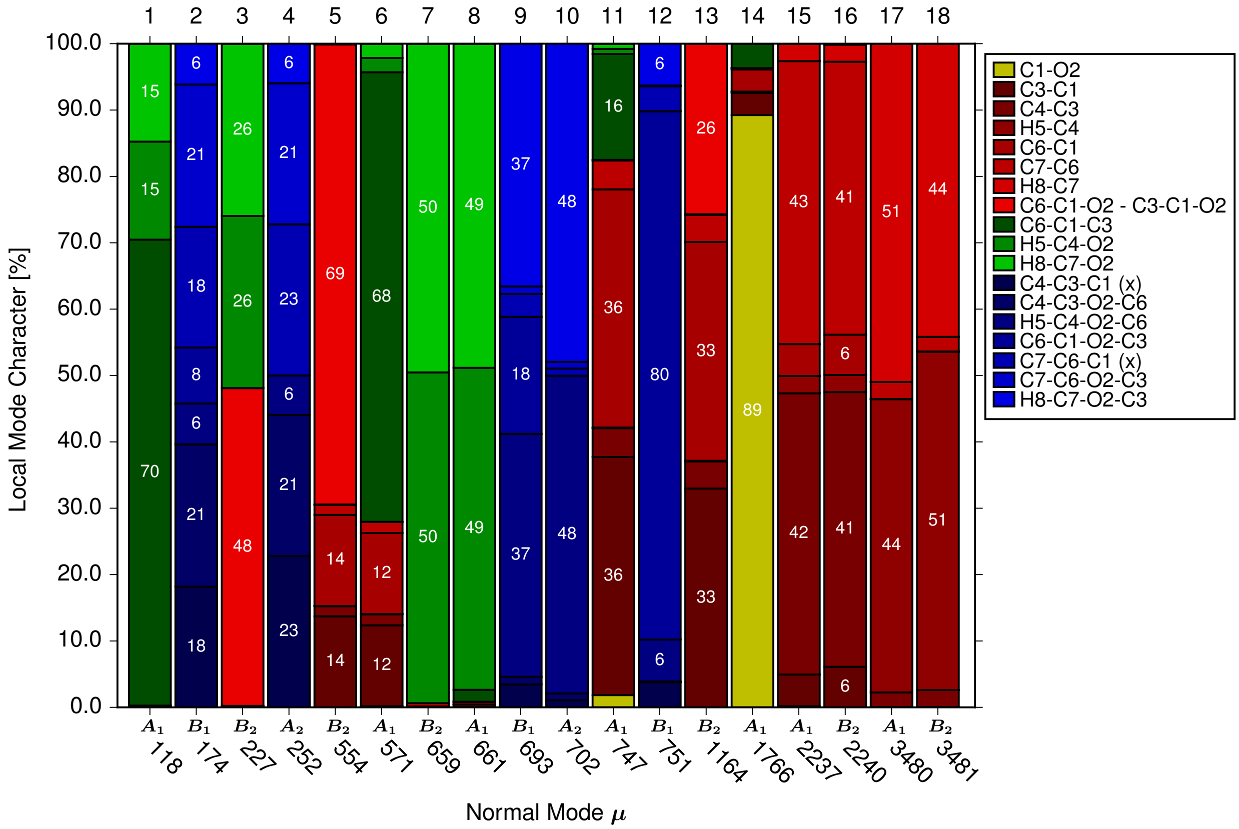

Figure 21: Decomposition of normal mode frequencies for 1-15

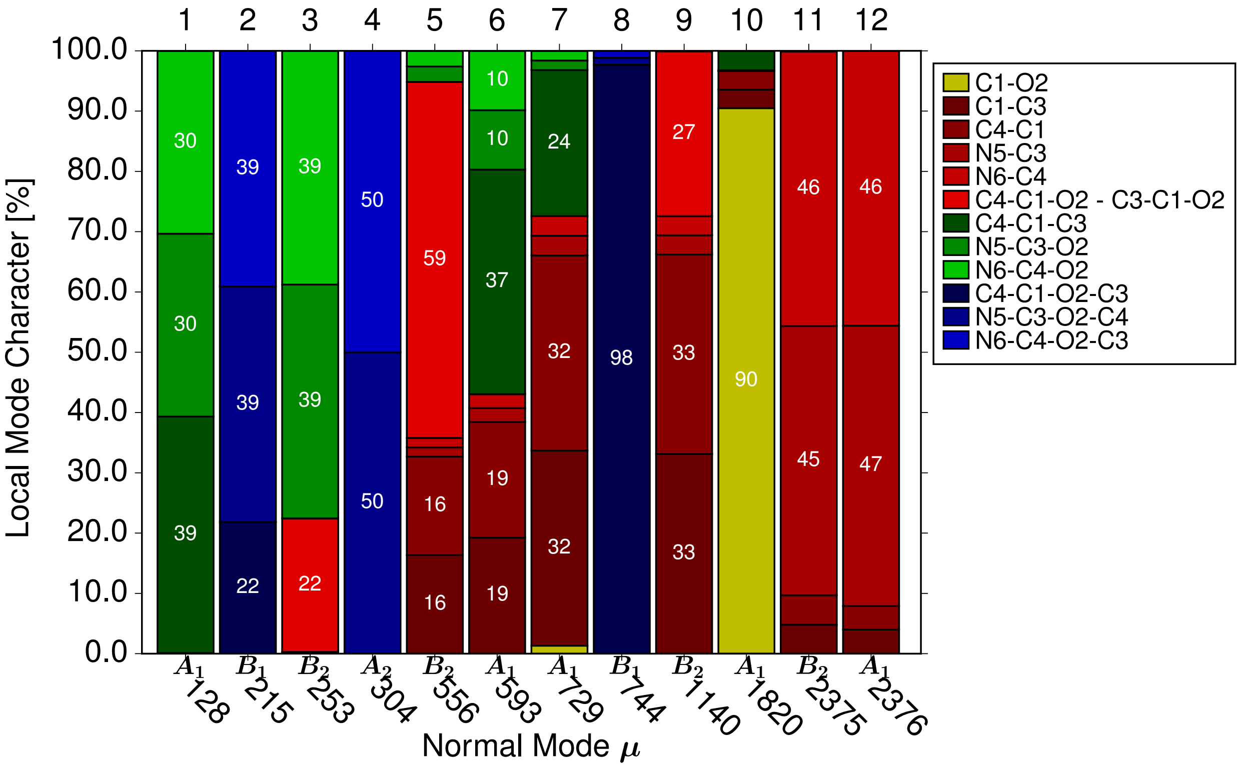

Figure 22: Decomposition of normal mode frequencies for 1-16

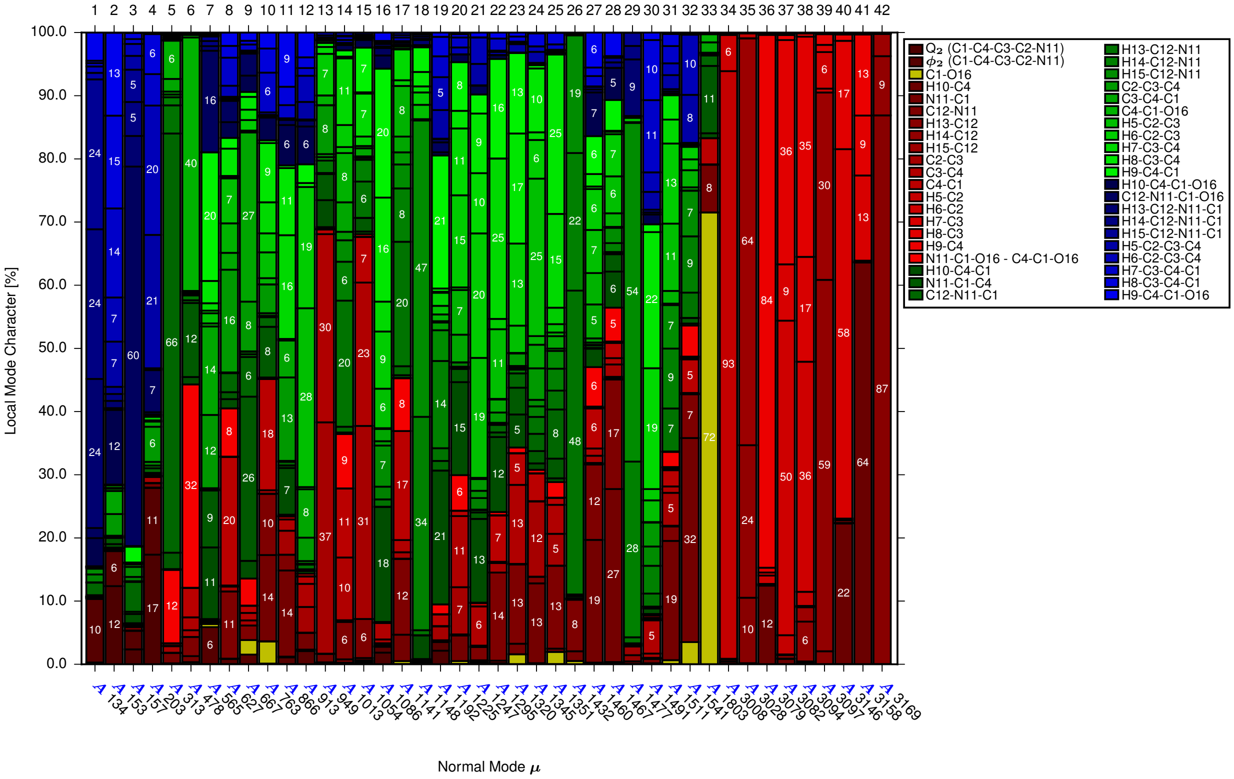

Figure 23: Decomposition of normal mode frequencies for 1-17

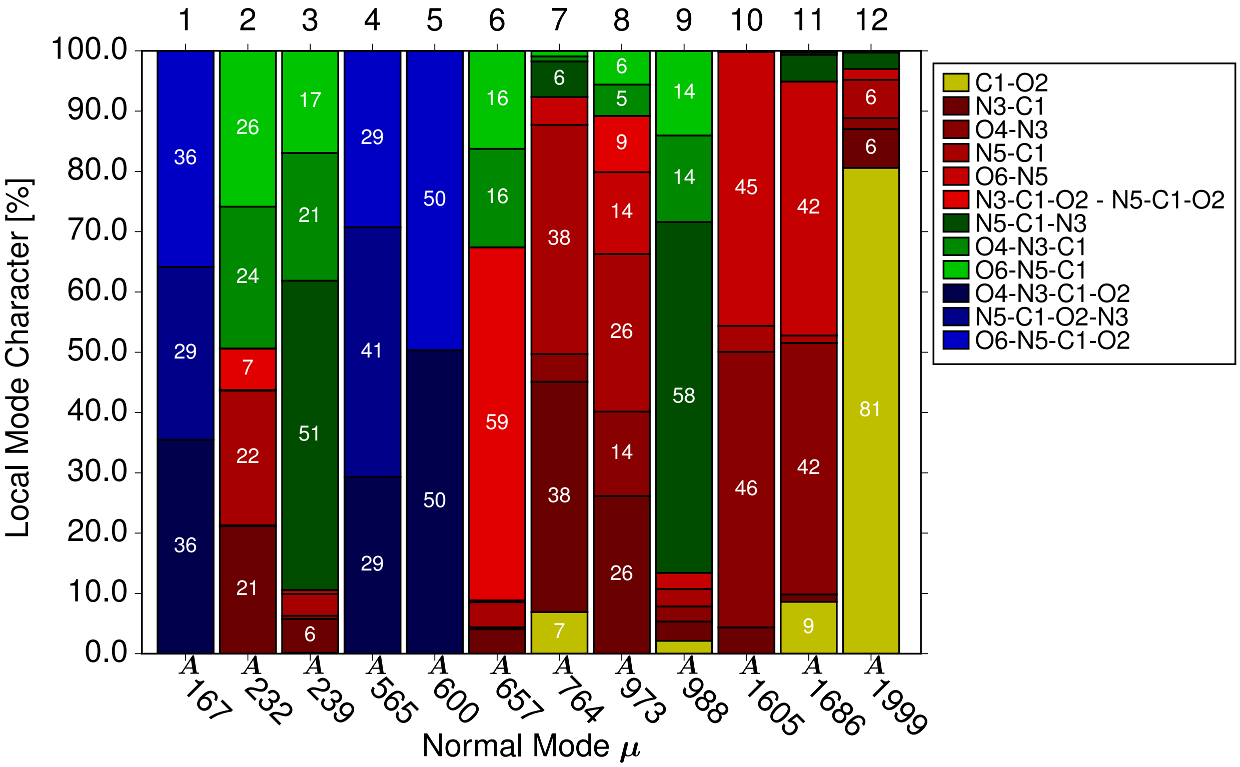

Figure 24: Decomposition of normal mode frequencies for 1-18

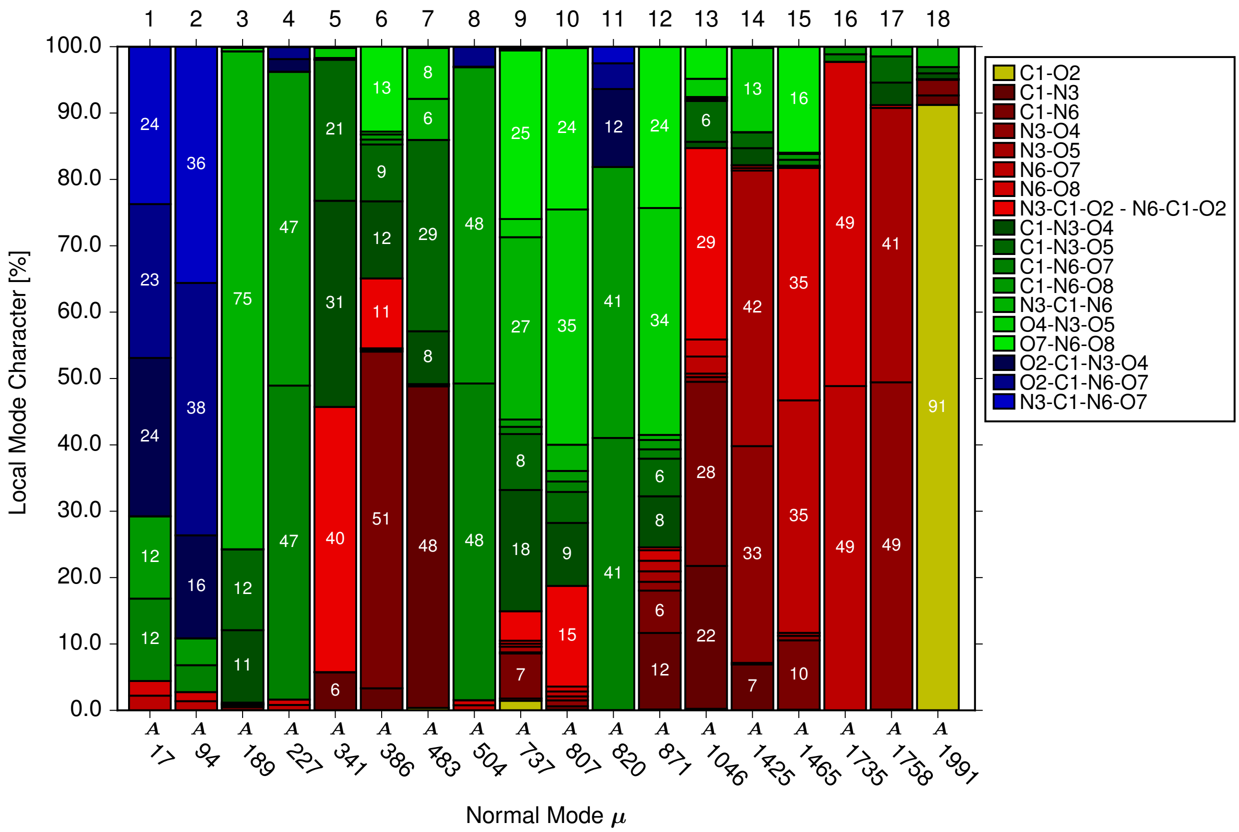

Figure 25: Decomposition of normal mode frequencies for 1-19

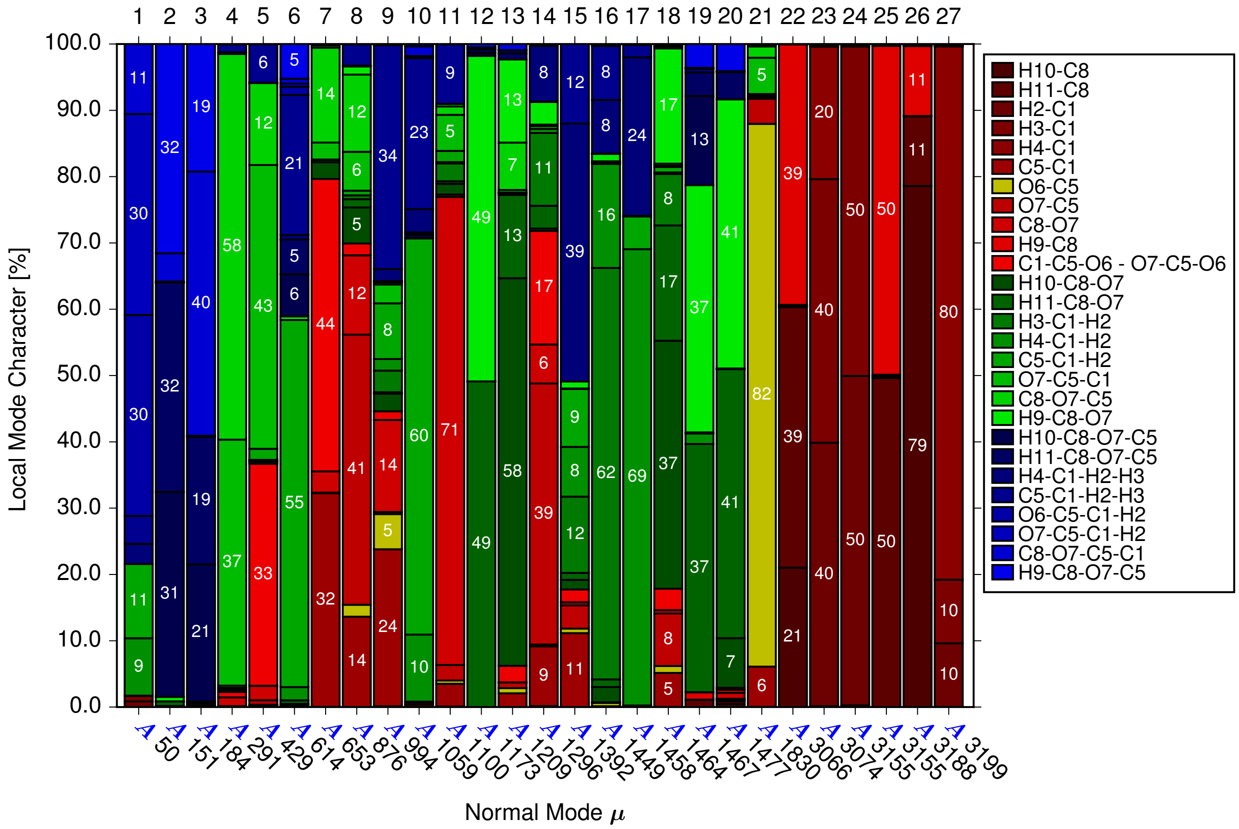

Figure 26: Decomposition of normal mode frequencies for 1-20

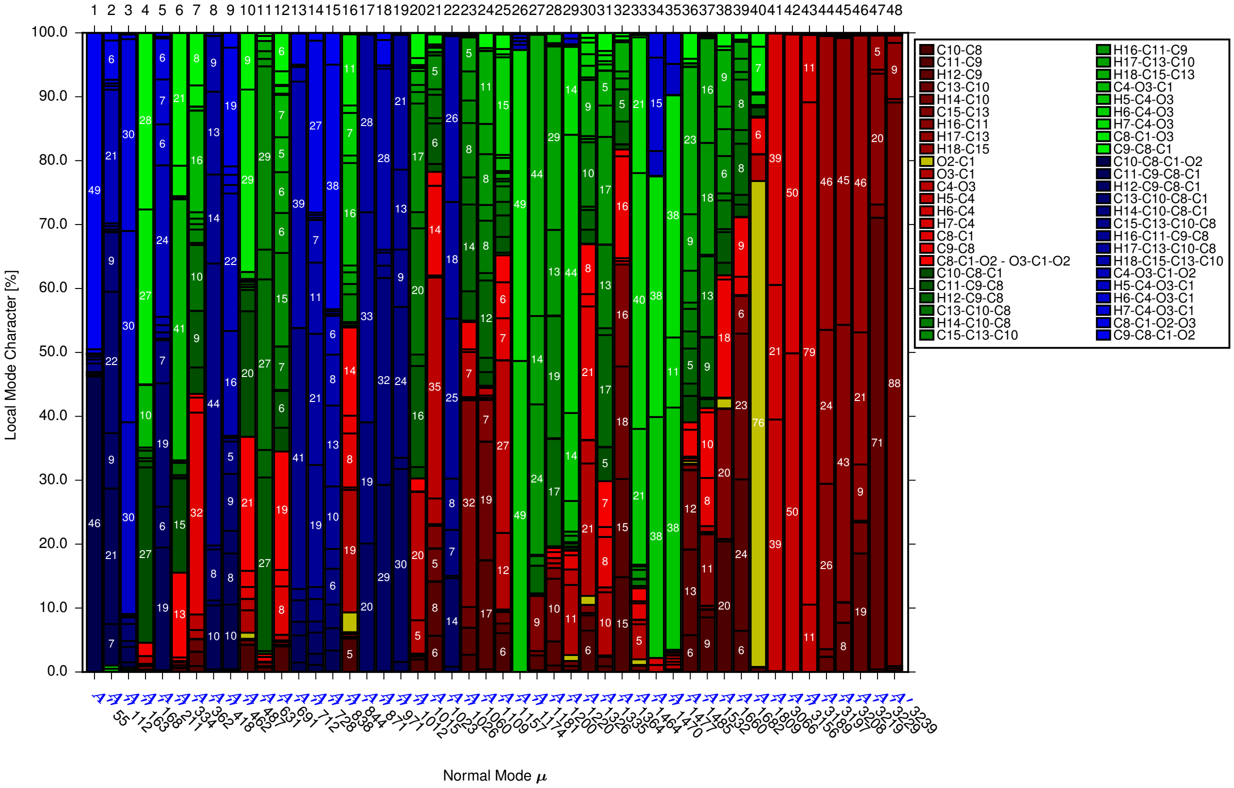

Figure 27: Decomposition of normal mode frequencies for 1-21

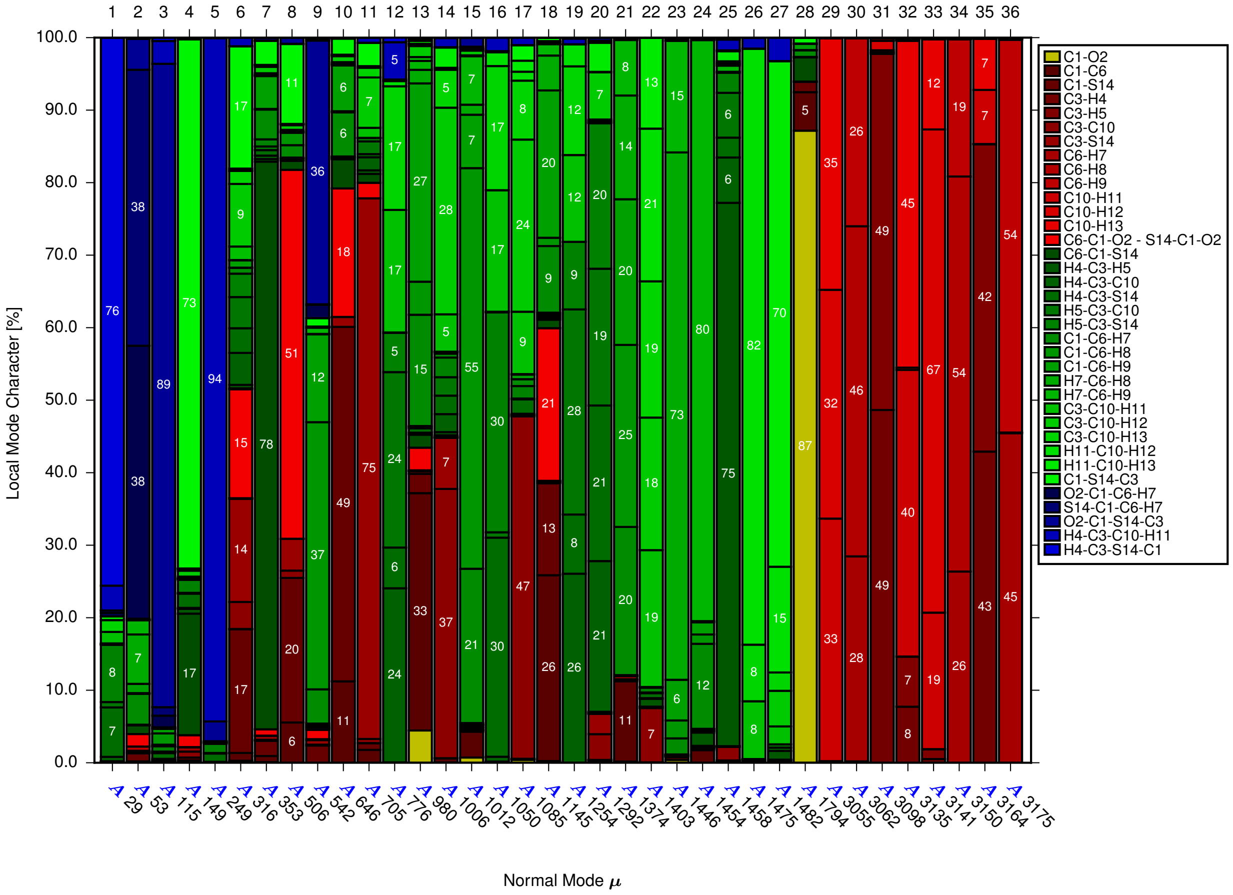

Figure 28: Decomposition of normal mode frequencies for 1-22

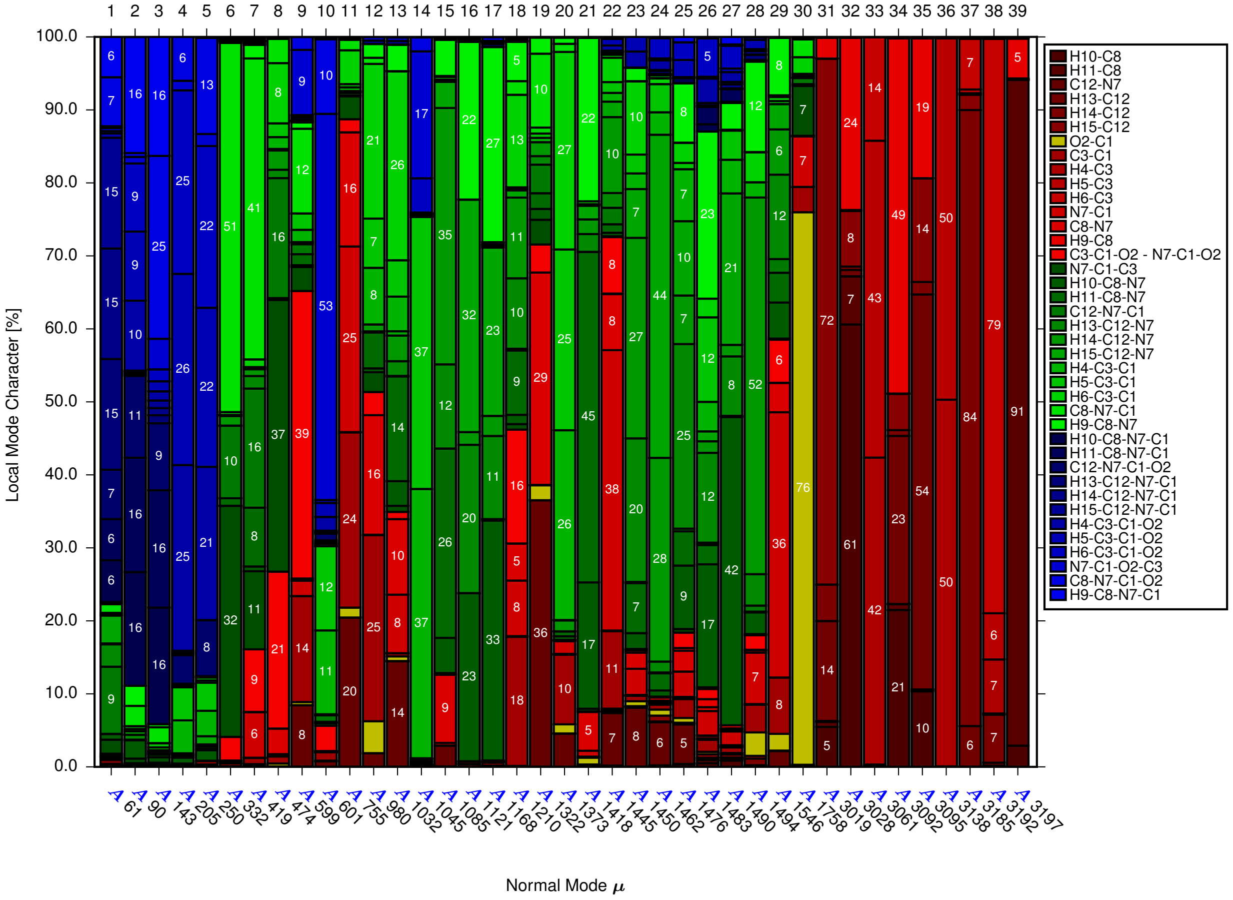

Figure 29: Decomposition of normal mode frequencies for 1-23

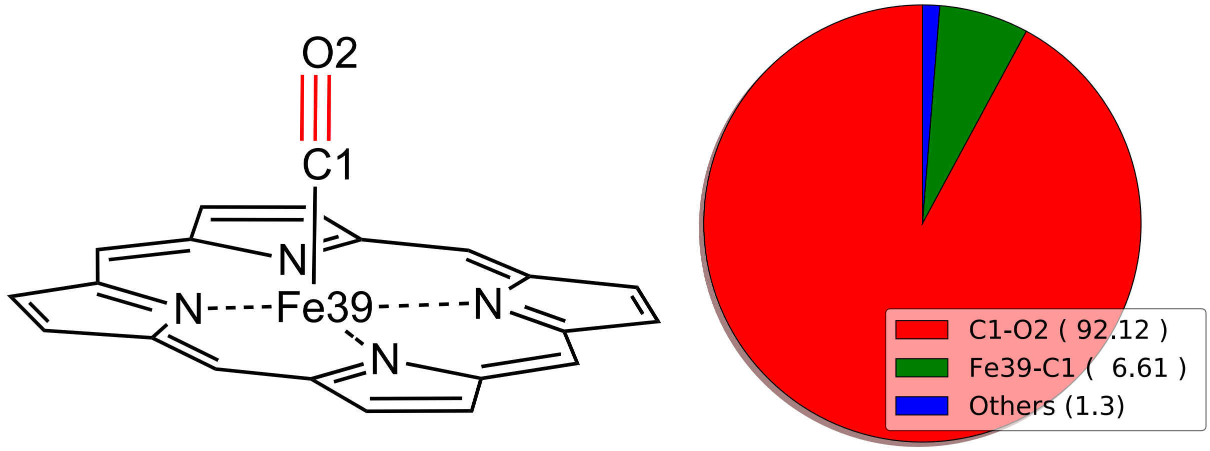

Figure 30: Decomposition of target (C=O bond) normal mode frequency for 1-24

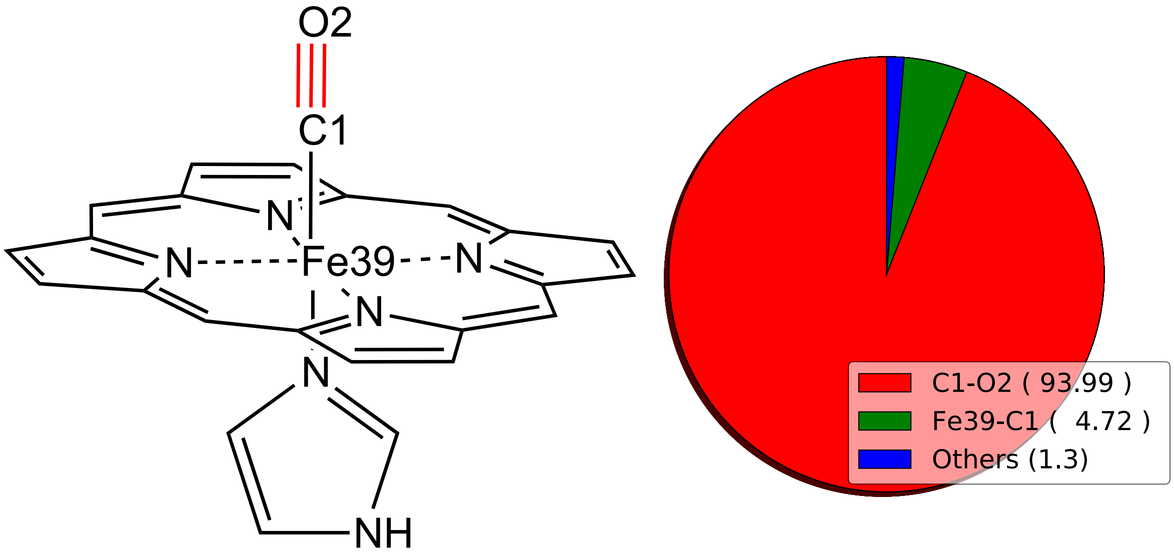

Figure 31: Decomposition of target (C=O bond) normal mode frequency for 1-25

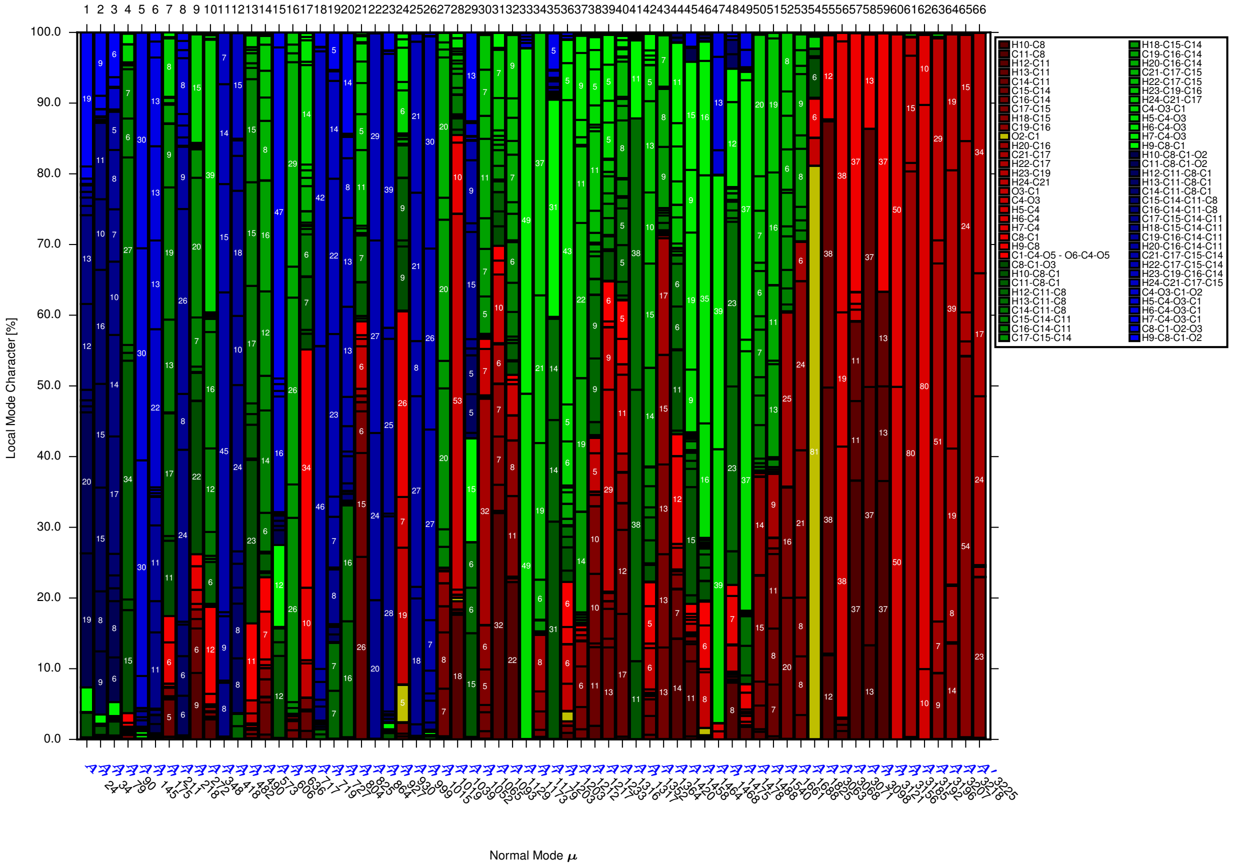

Figure 32: Decomposition of normal mode frequencies for 1-26

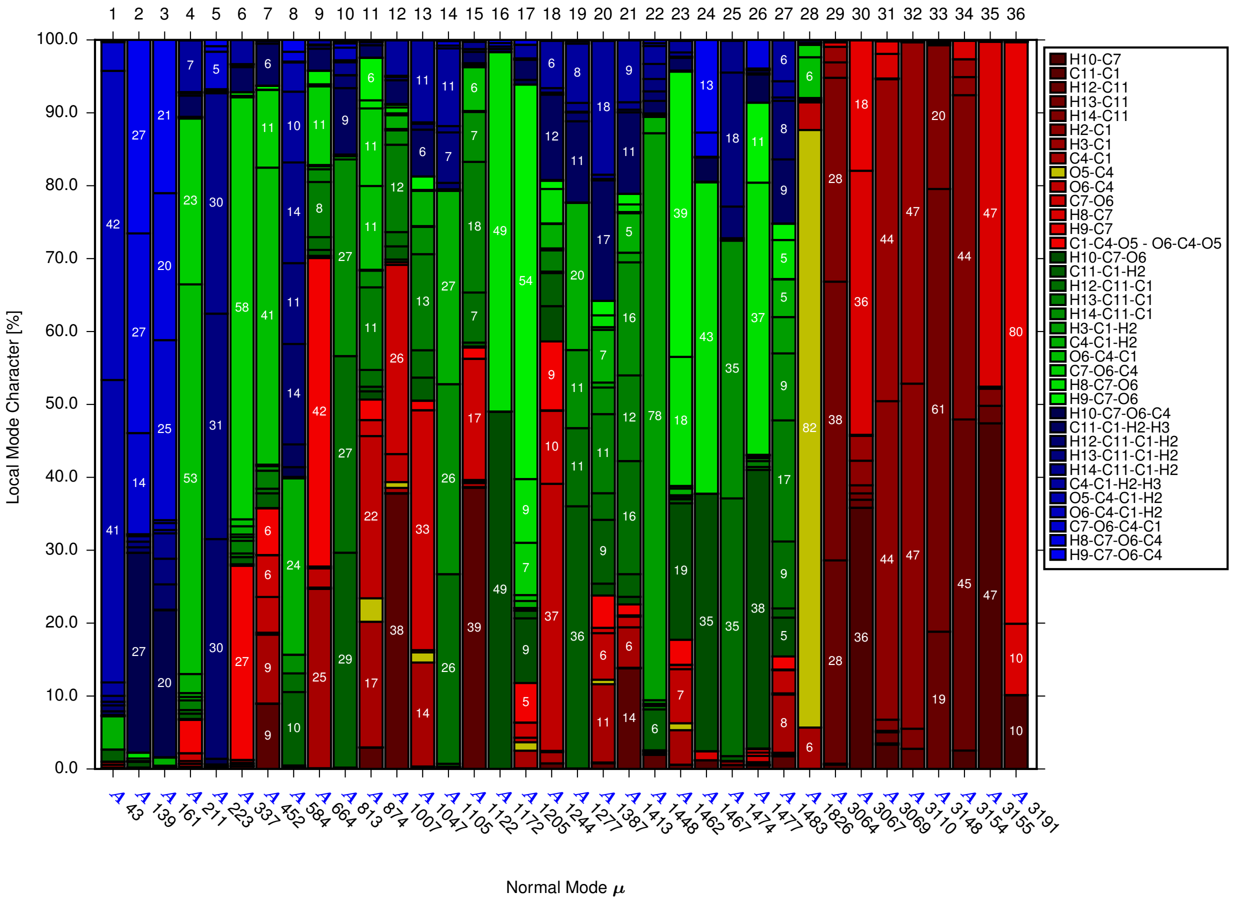

Figure 33: Decomposition of normal mode frequencies for 1-27

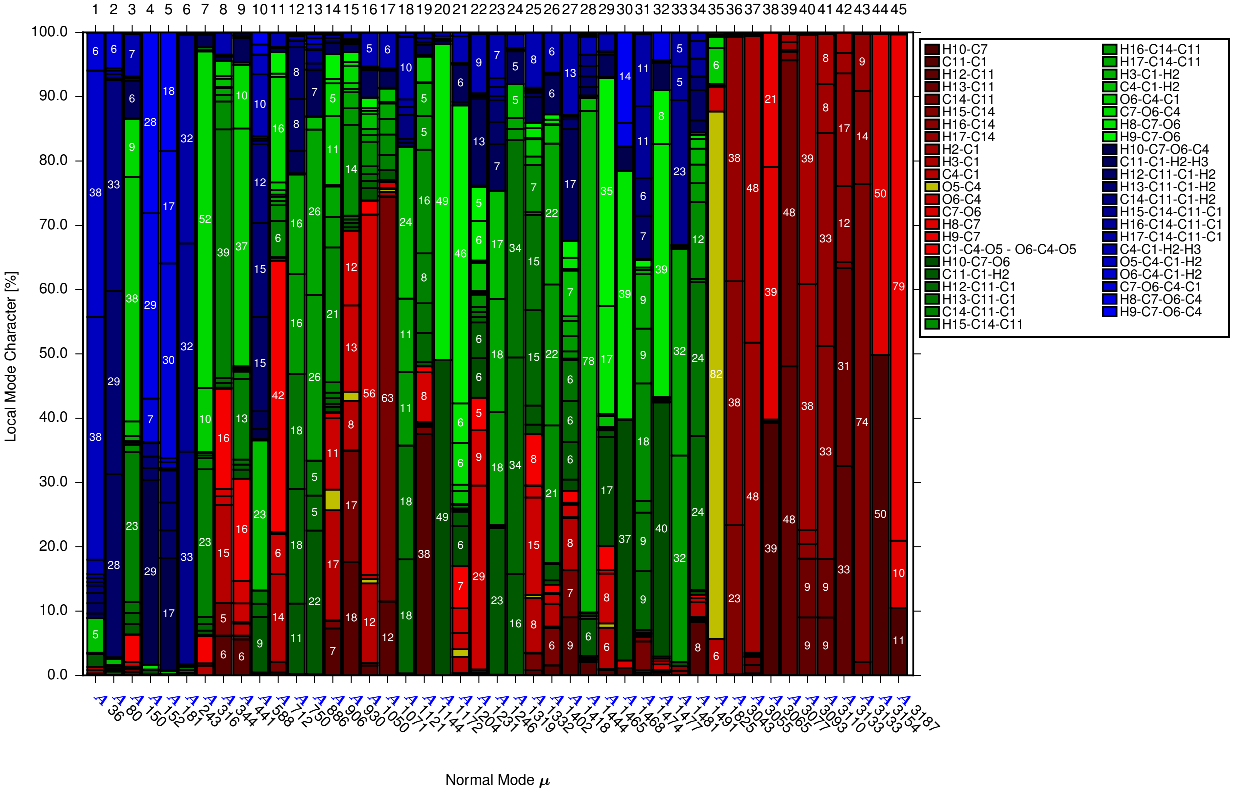

Figure 34: Decomposition of normal mode frequencies for 1-28

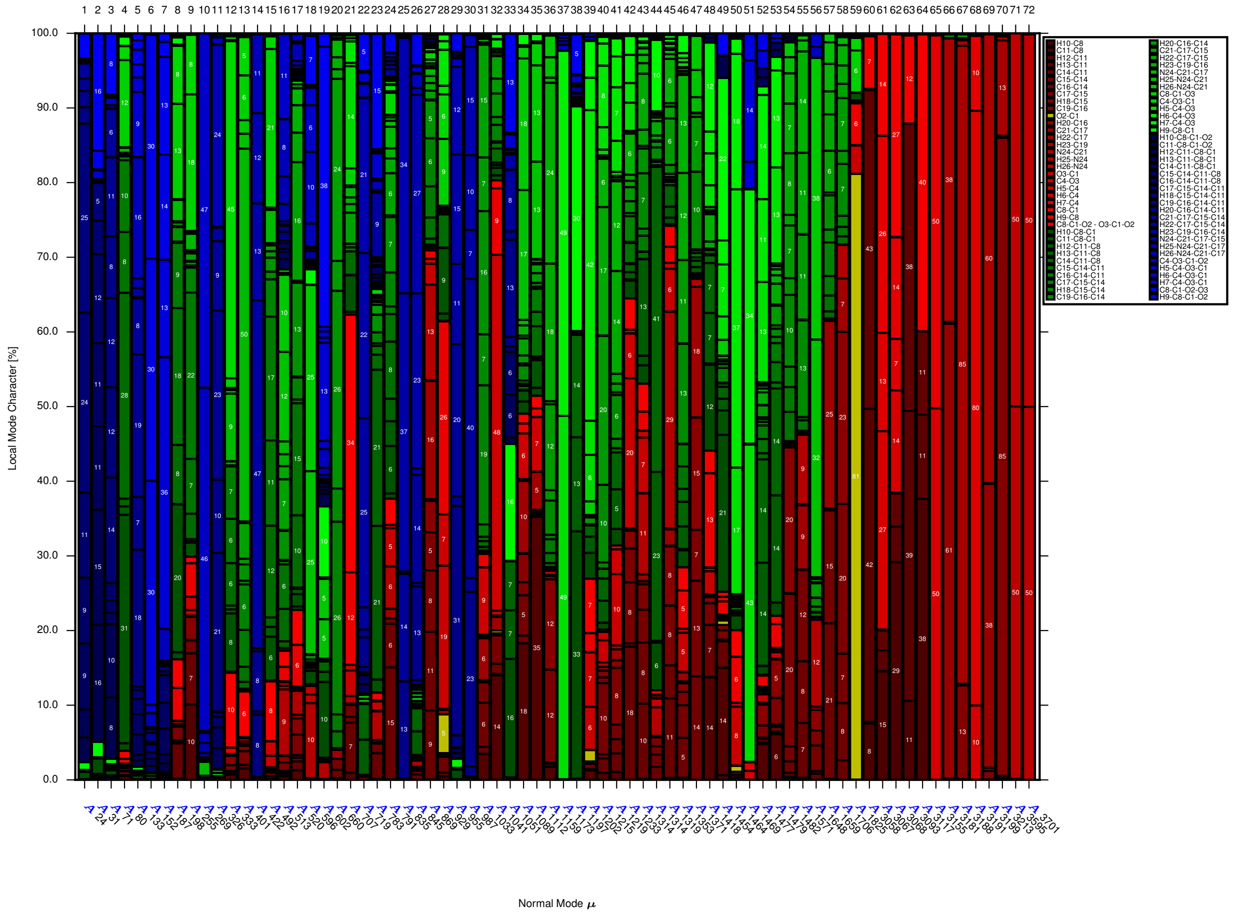

Figure 35: Decomposition of normal mode frequencies for 1-29

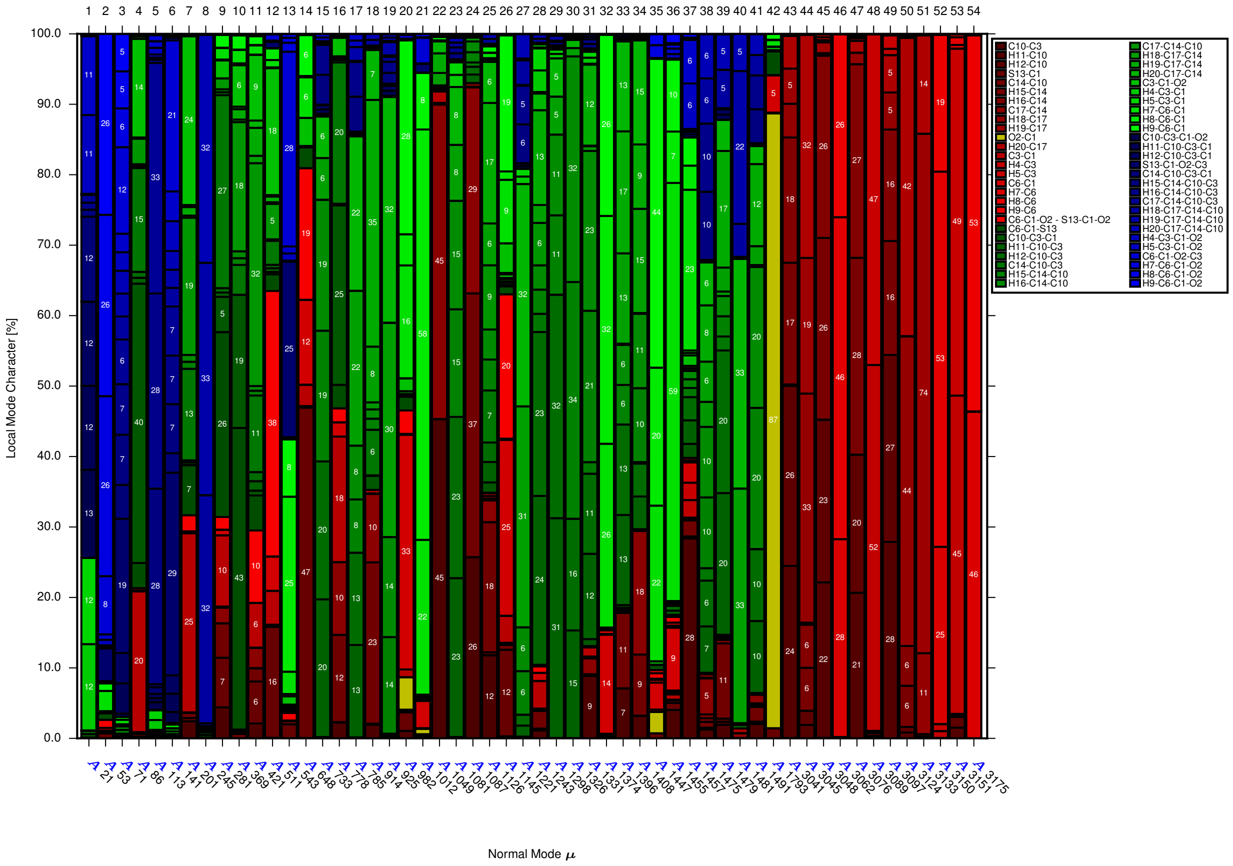

Figure 36: Decomposition of normal mode frequencies for 1-30

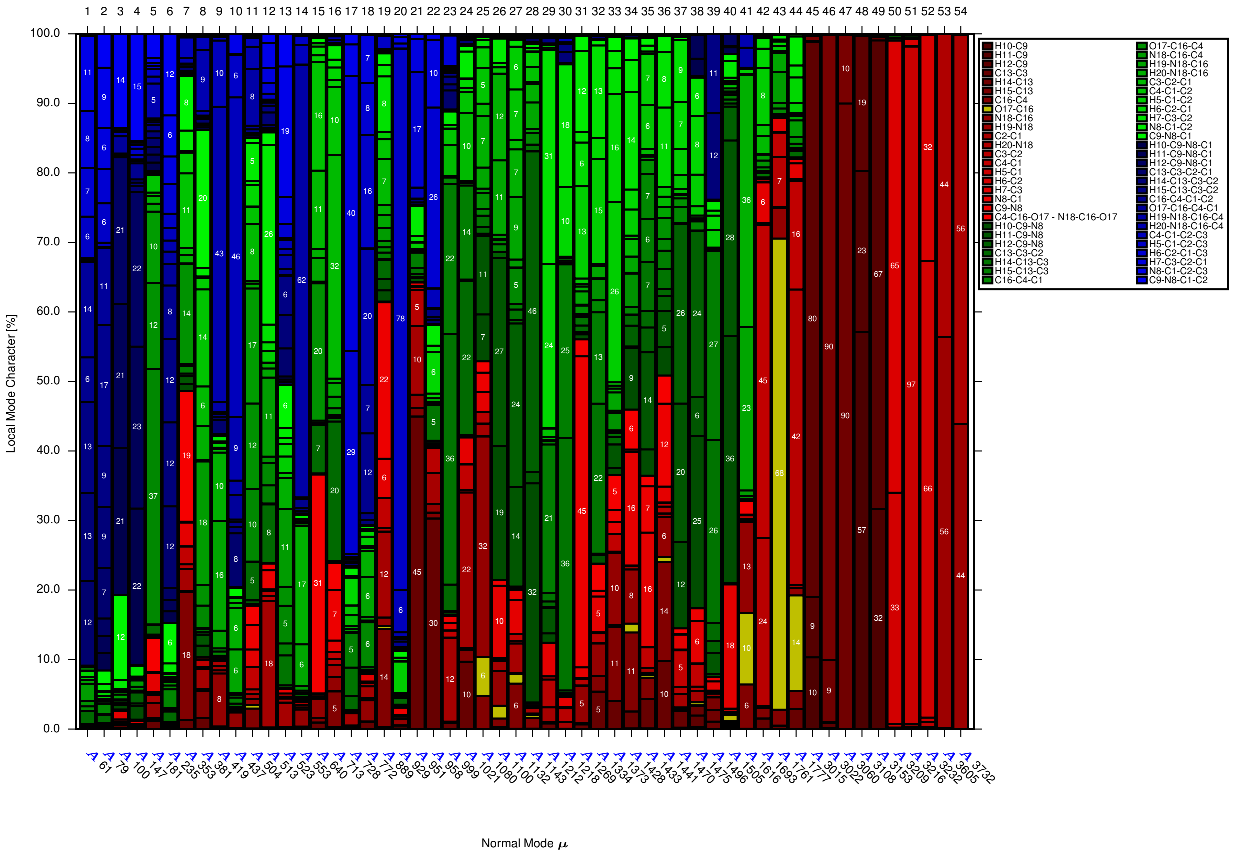

Figure 37: Decomposition of normal mode frequencies for 1-31

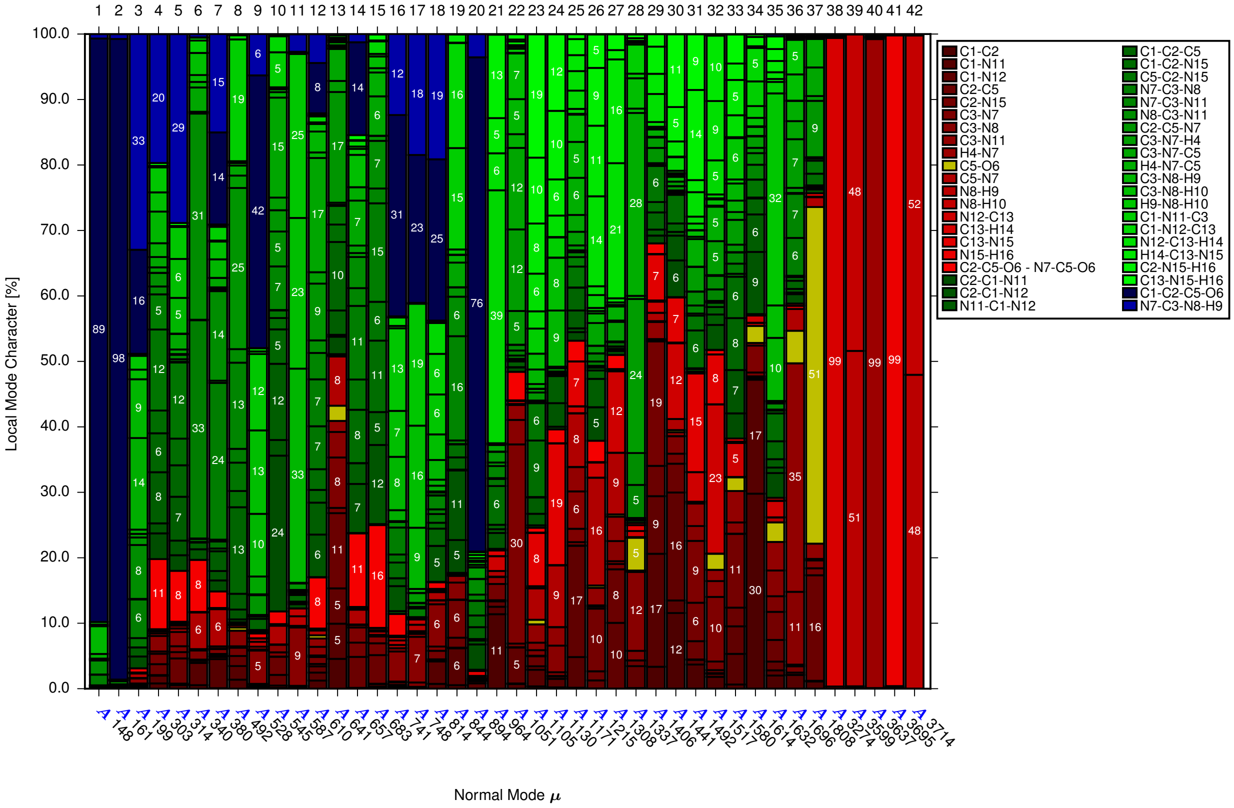

Figure 38: Decomposition of normal mode frequencies for 1-32

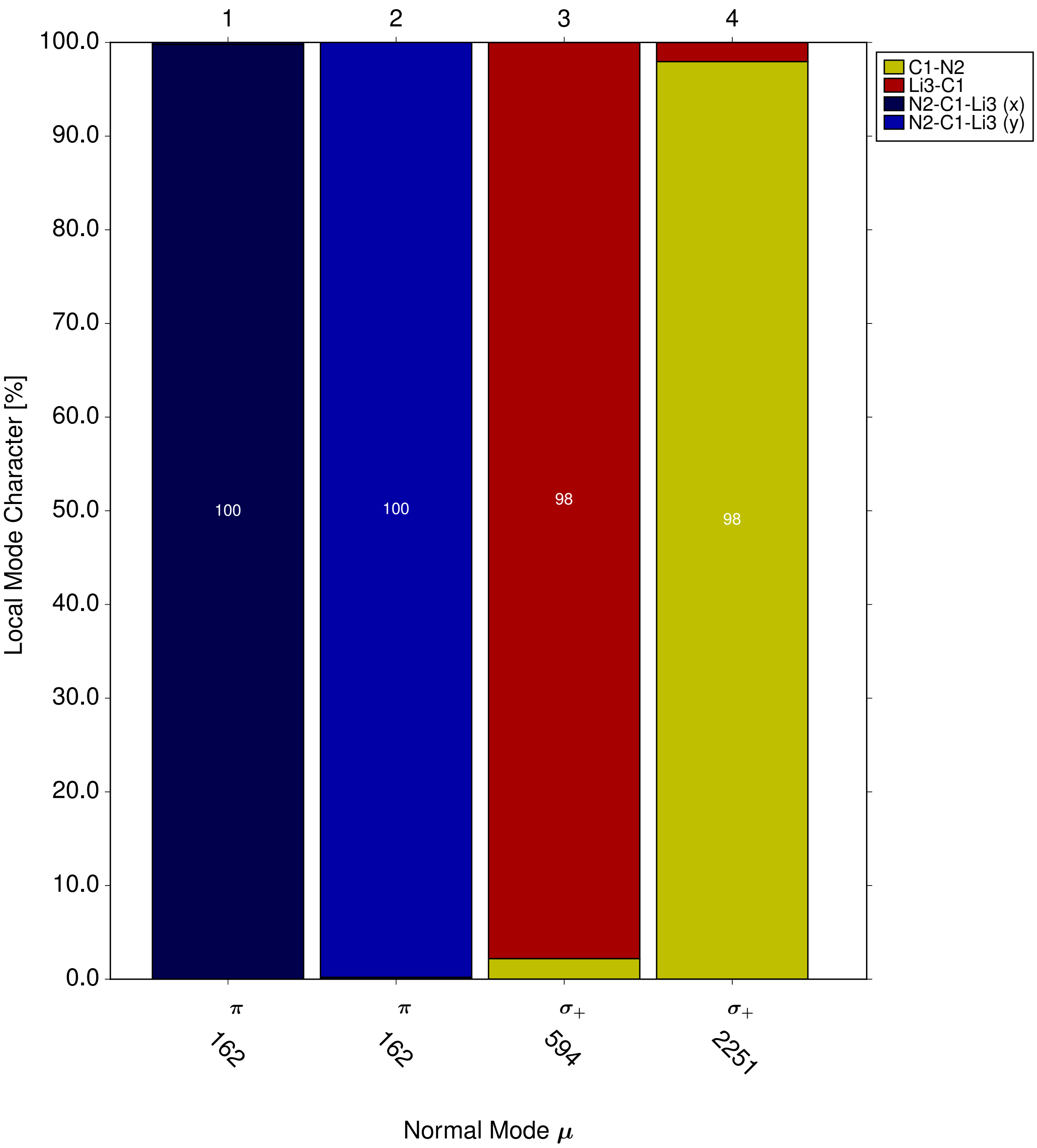

Figure 39: Decomposition of normal mode frequencies for 2-1

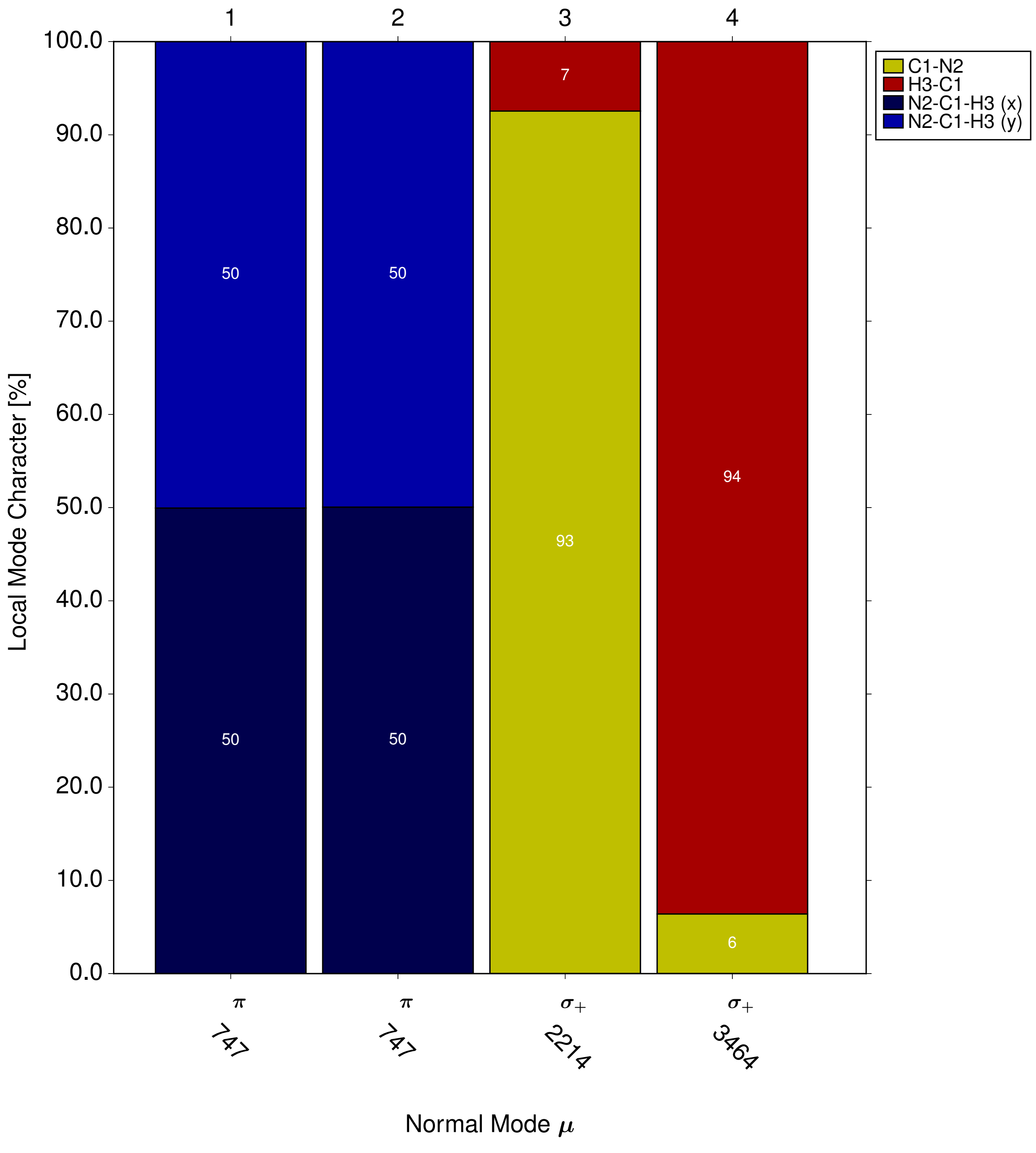

Figure 40: Decomposition of normal mode frequencies for 2-2

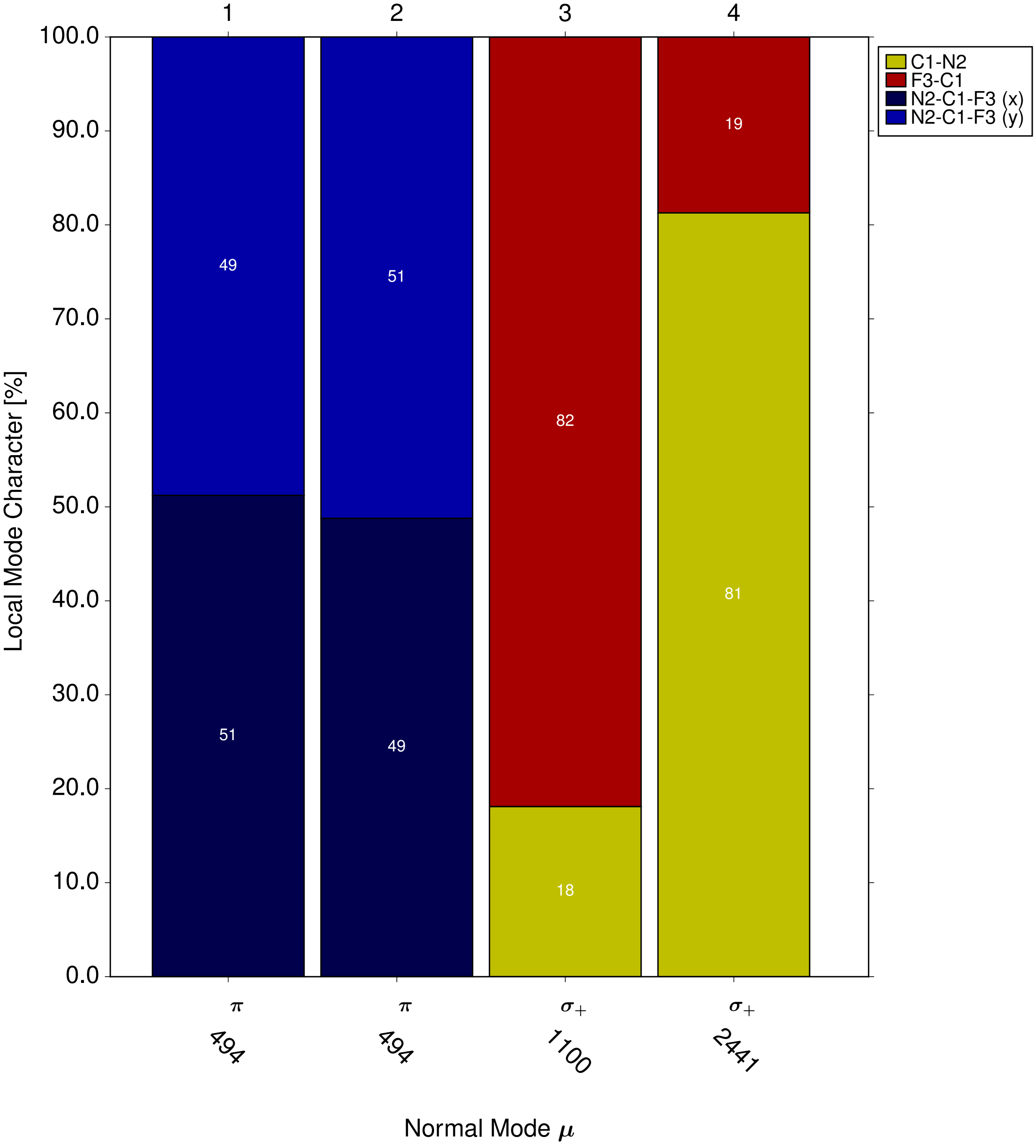

Figure 41: Decomposition of normal mode frequencies for 2-3

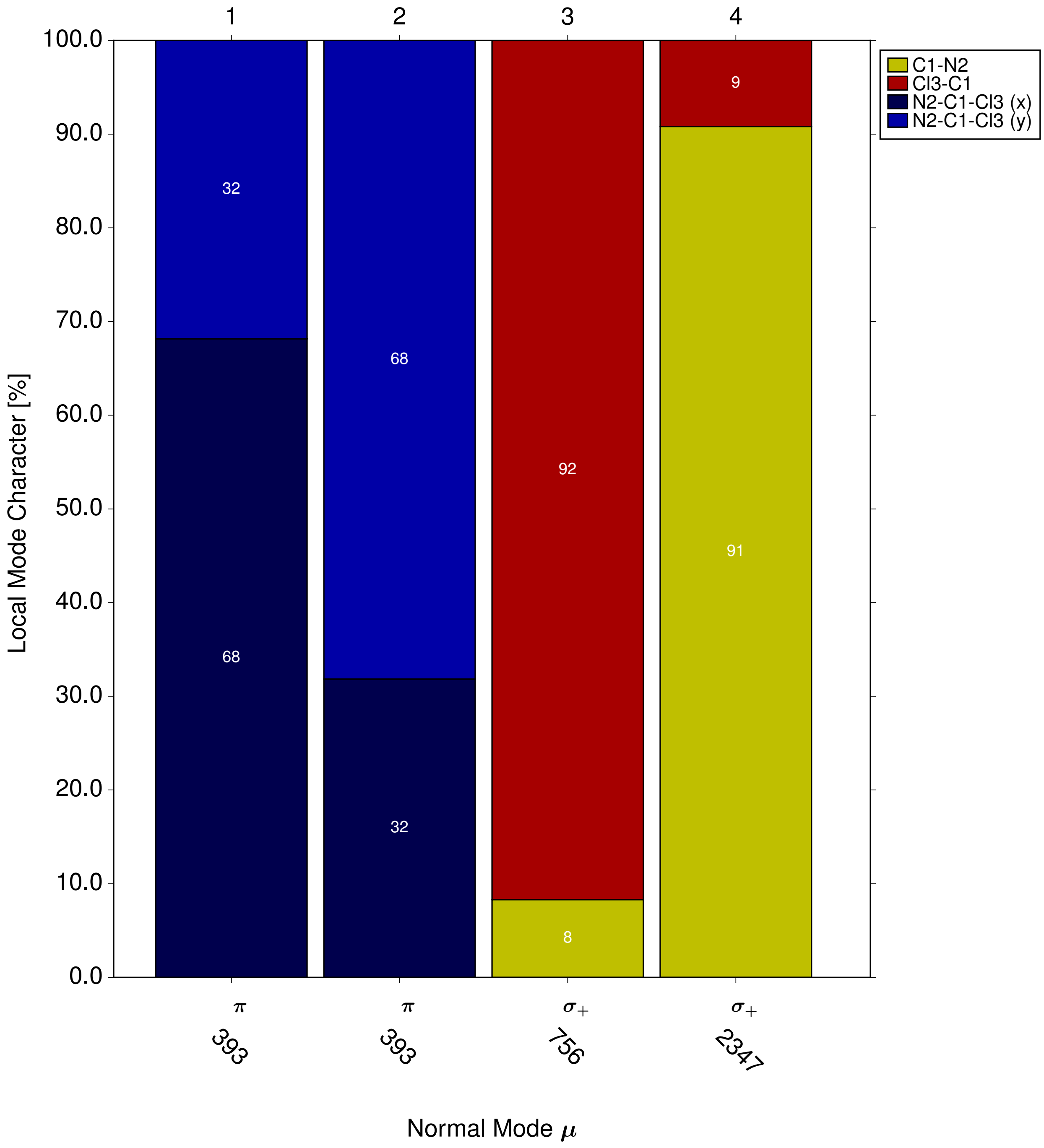

Figure 42: Decomposition of normal mode frequencies for 2-4

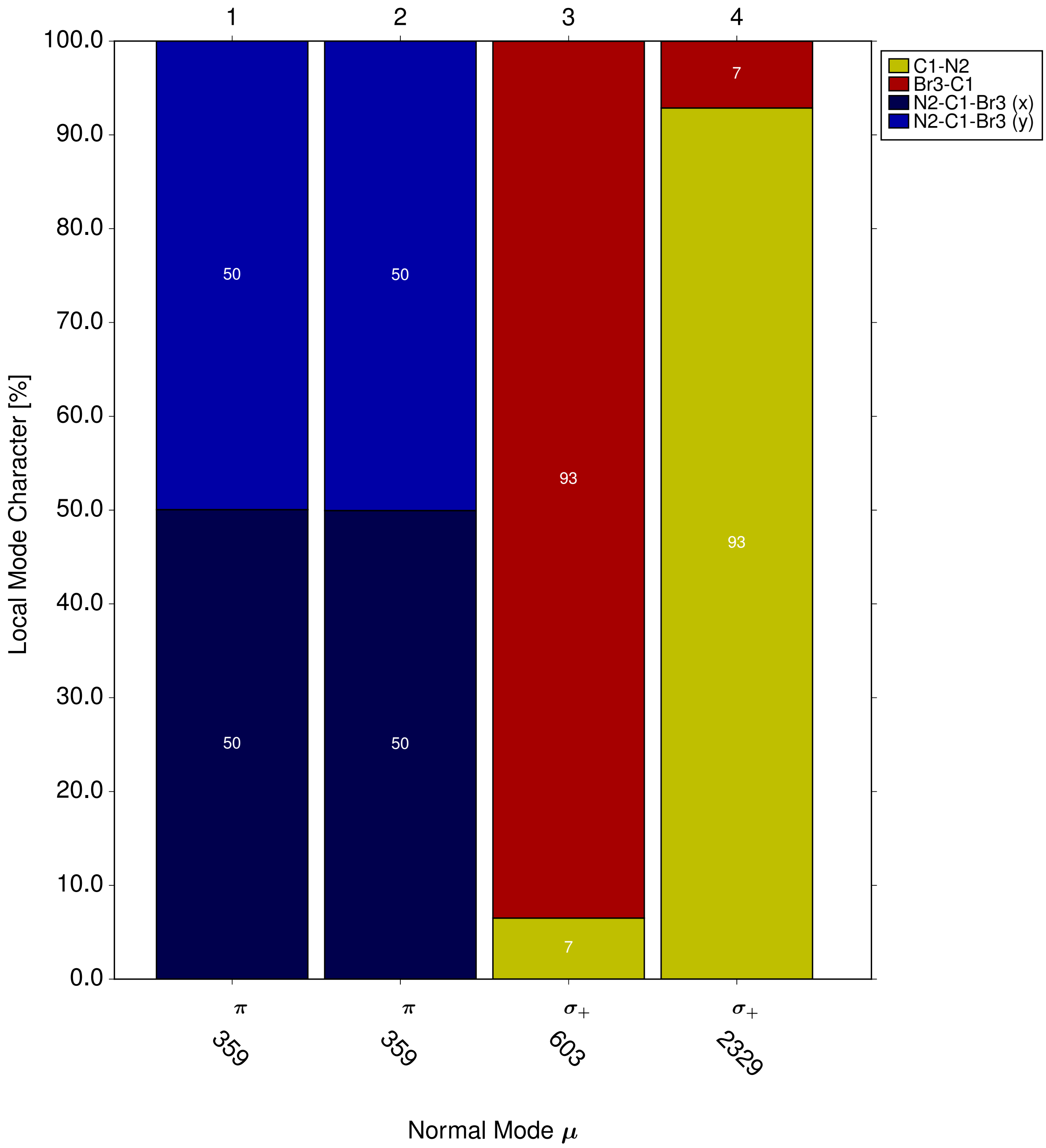

Figure 43: Decomposition of normal mode frequencies for 2-5

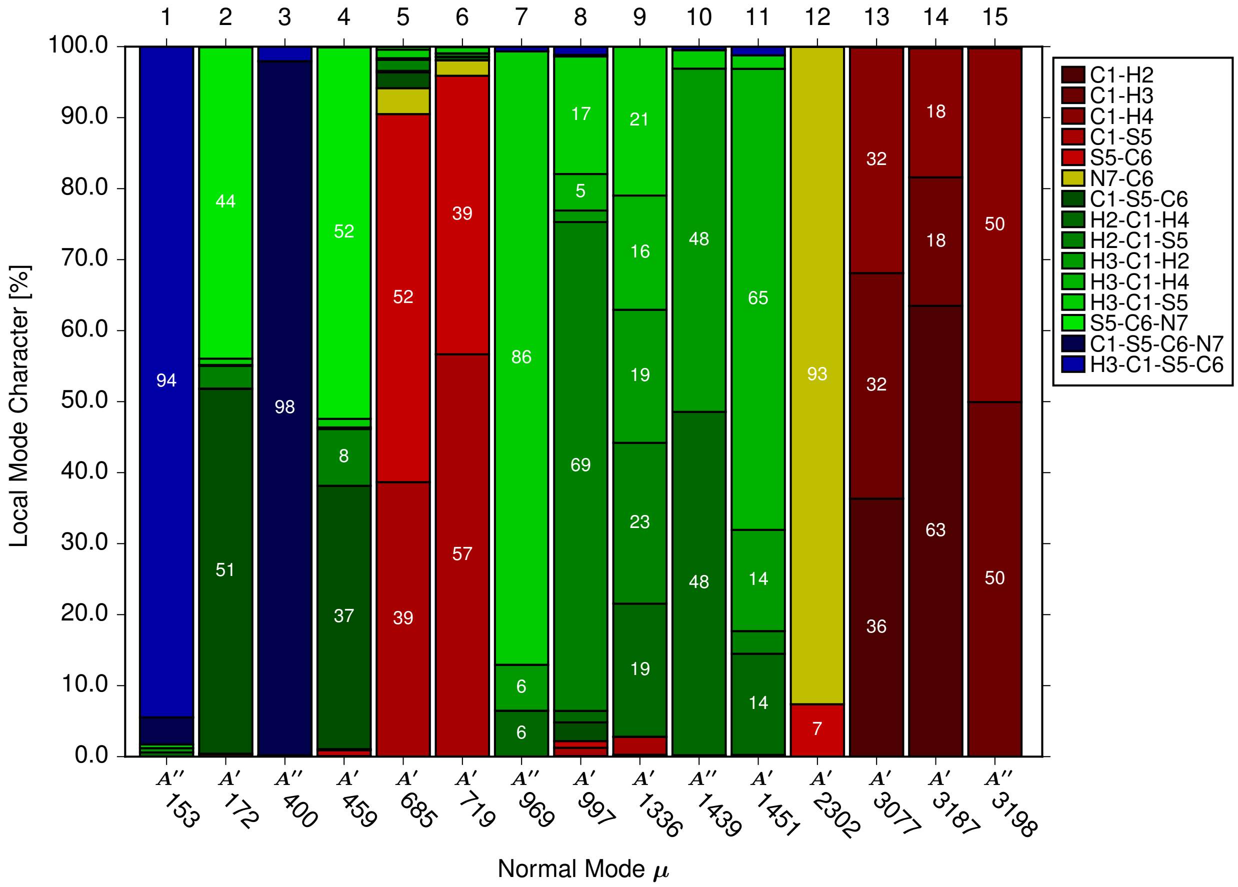

Figure 44: Decomposition of normal mode frequencies for 2-6

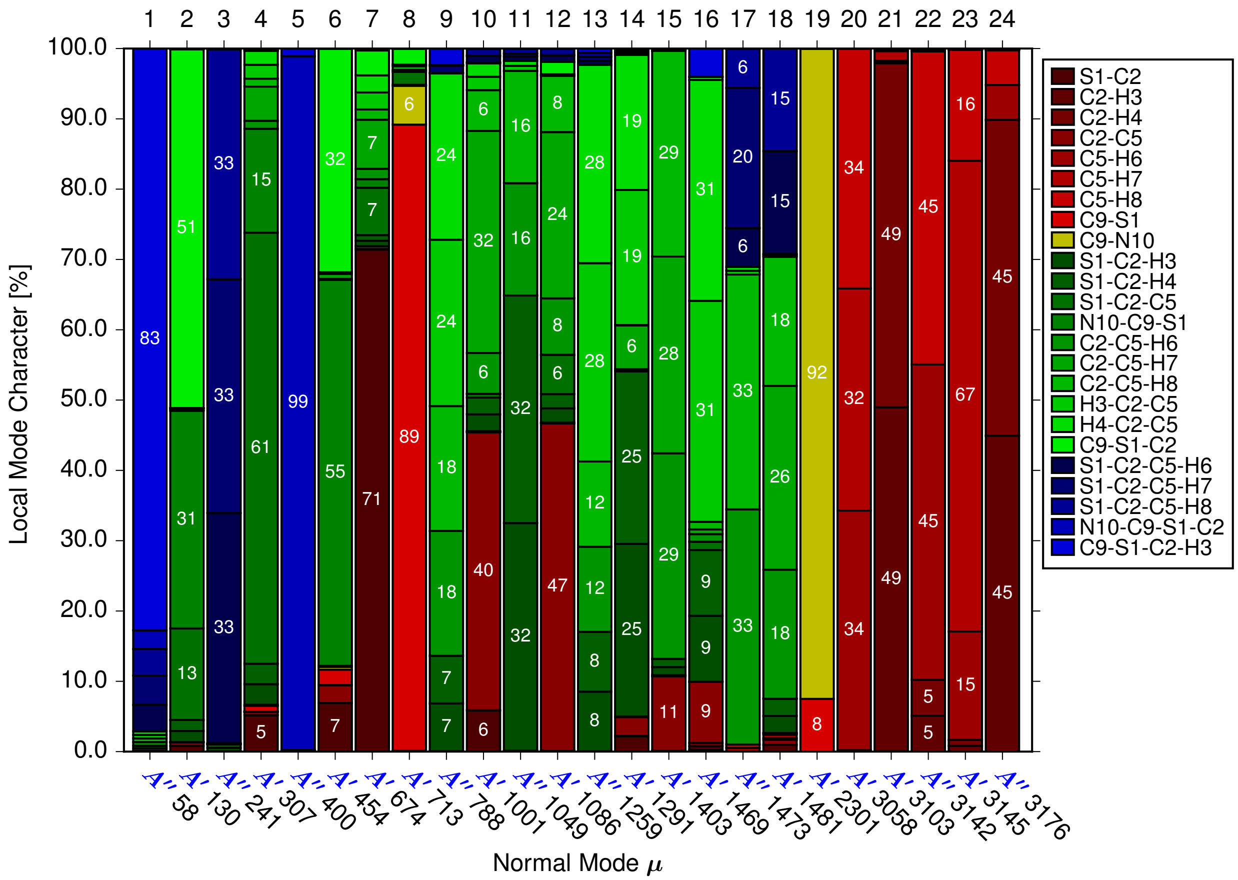

Figure 45: Decomposition of normal mode frequencies for 2-7

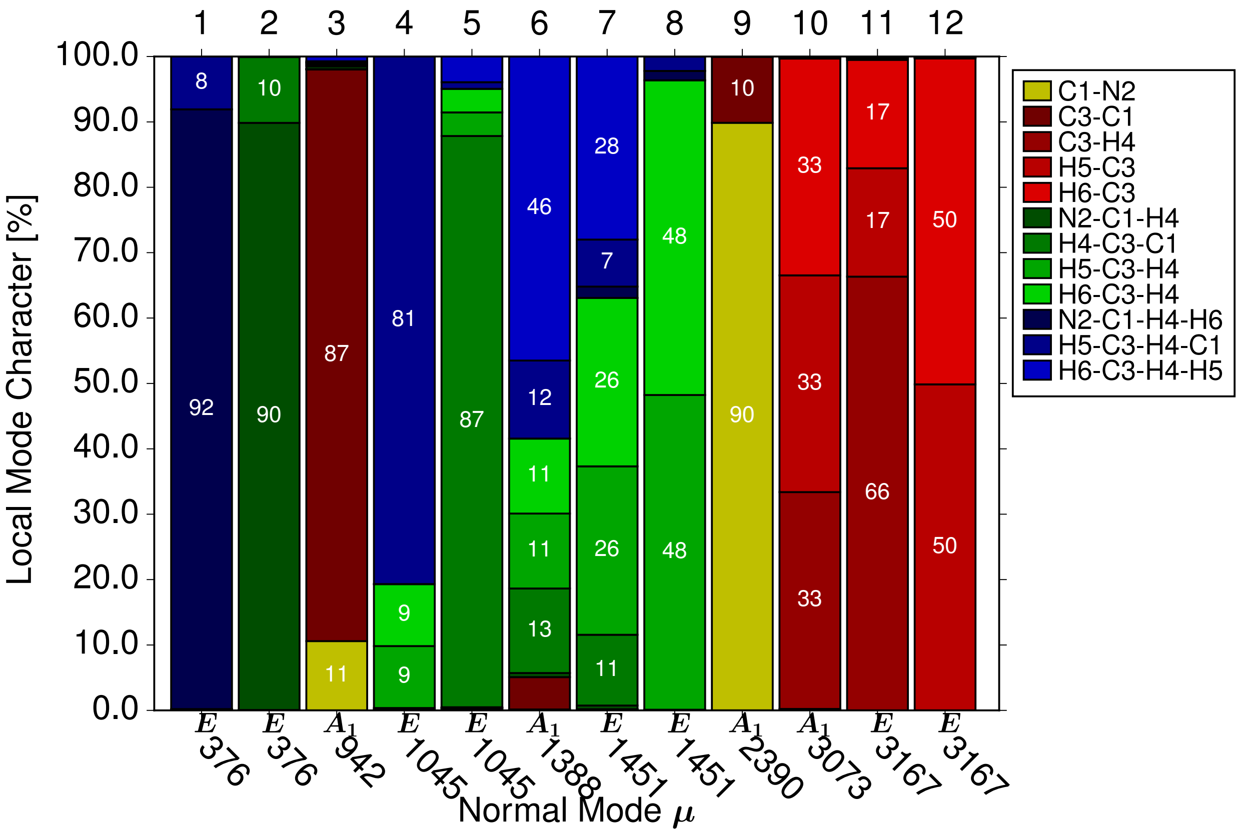

Figure 46: Decomposition of normal mode frequencies for 2-8

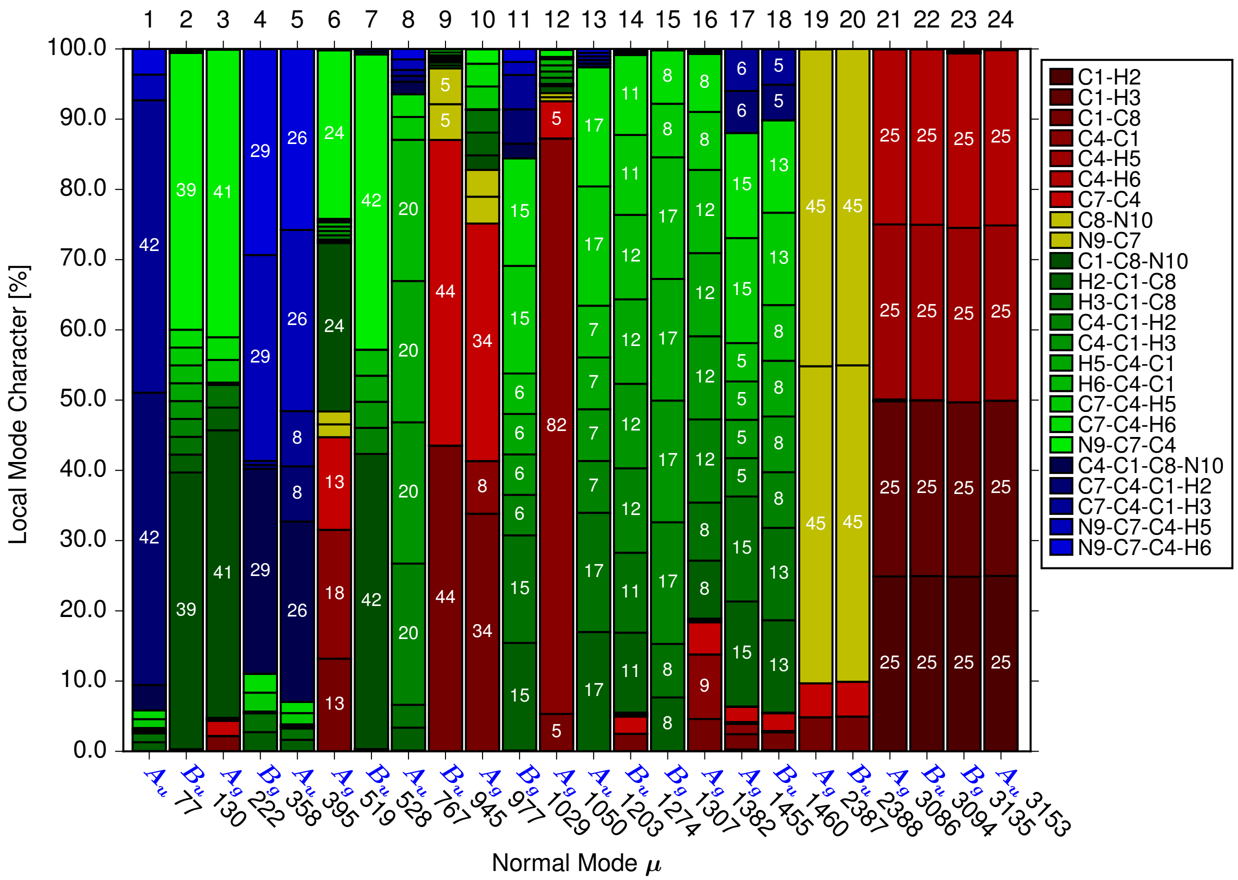

Figure 47: Decomposition of normal mode frequencies for 2-9

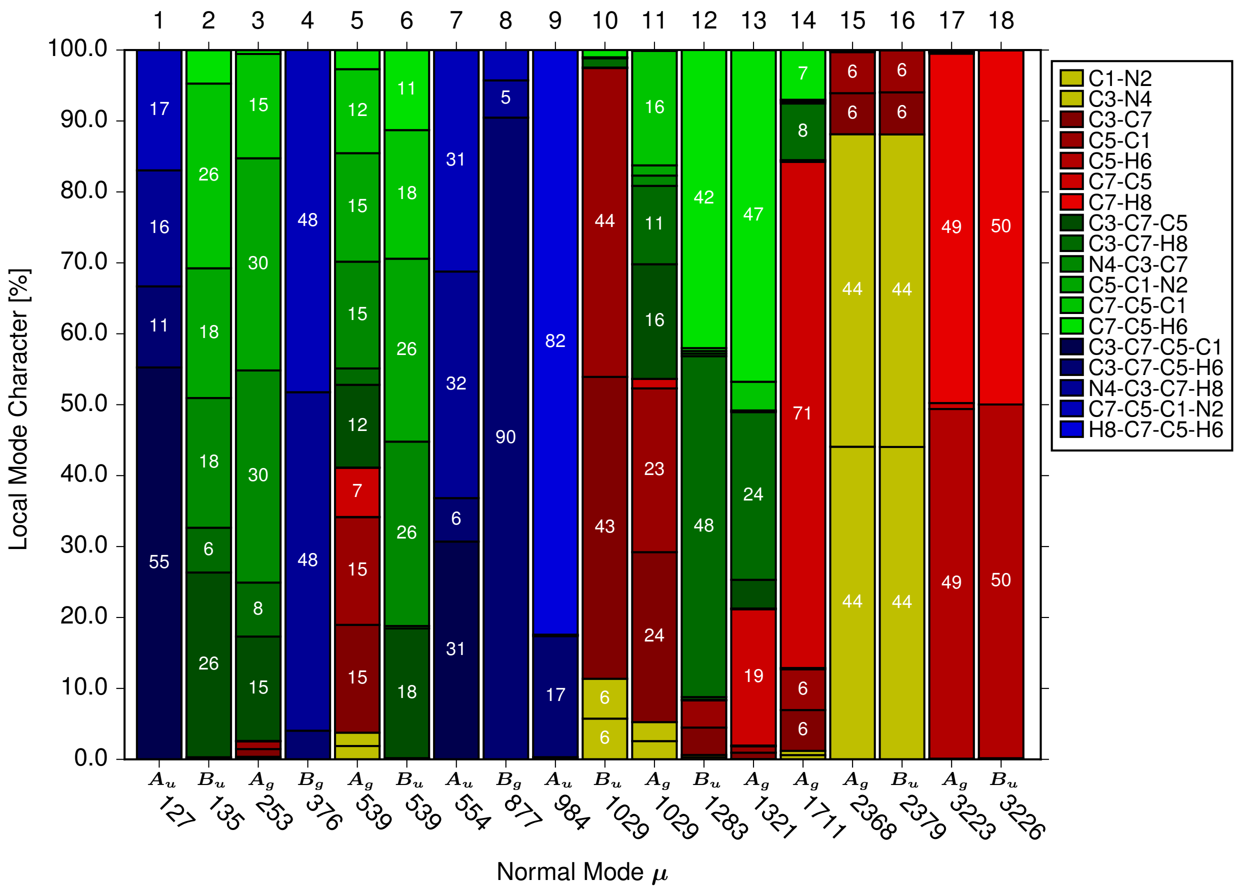

Figure 48: Decomposition of normal mode frequencies for 2-10

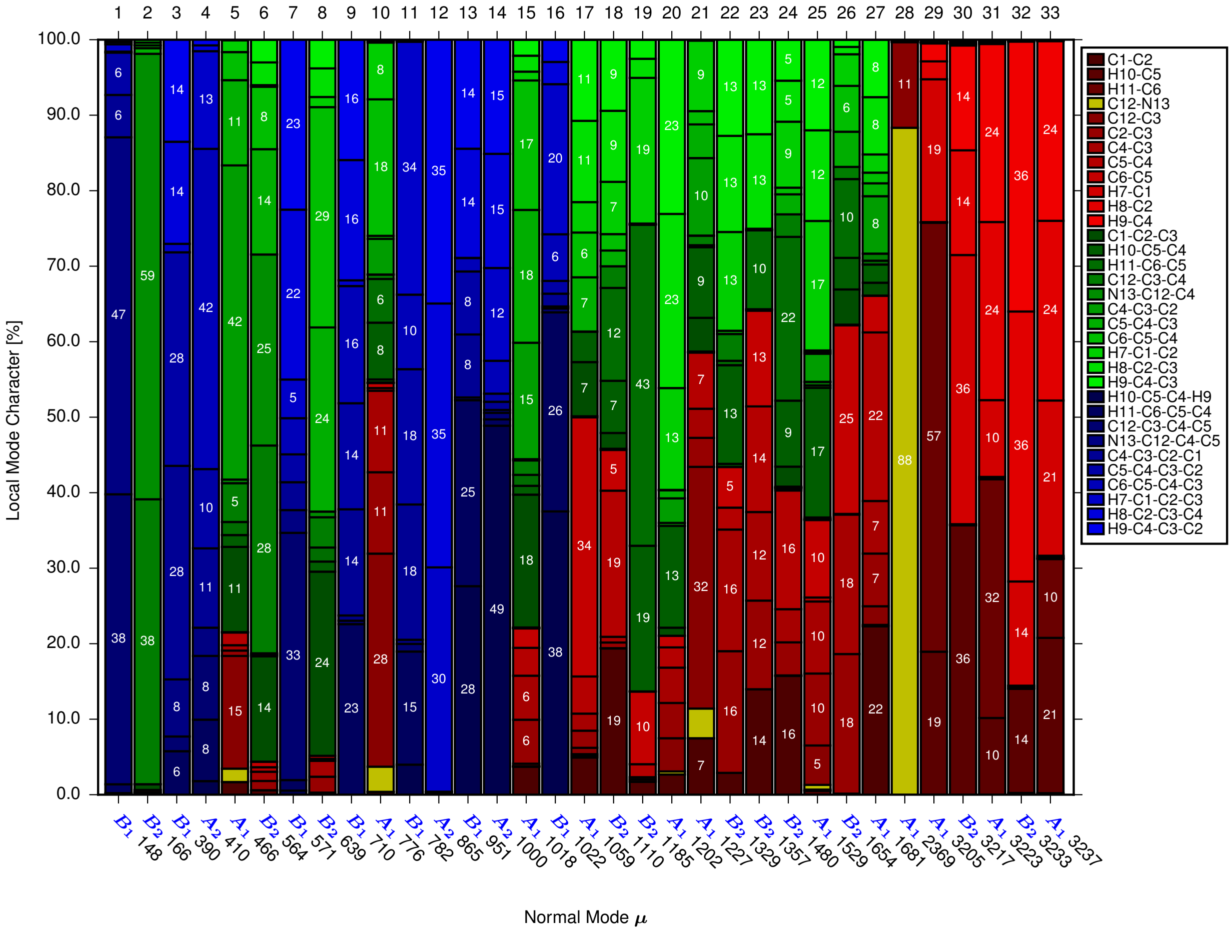

Figure 49: Decomposition of normal mode frequencies for 2-11

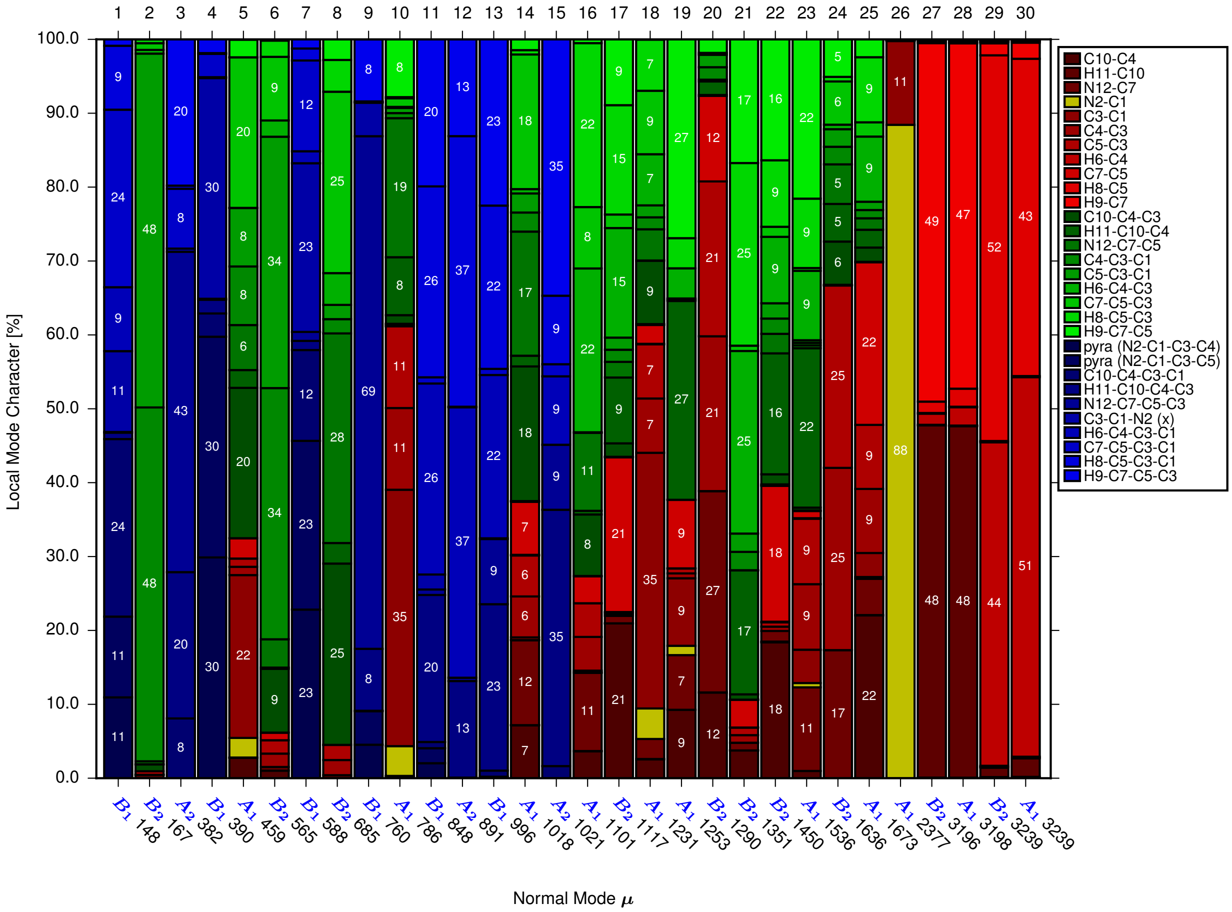

Figure 50: Decomposition of normal mode frequencies for 2-12

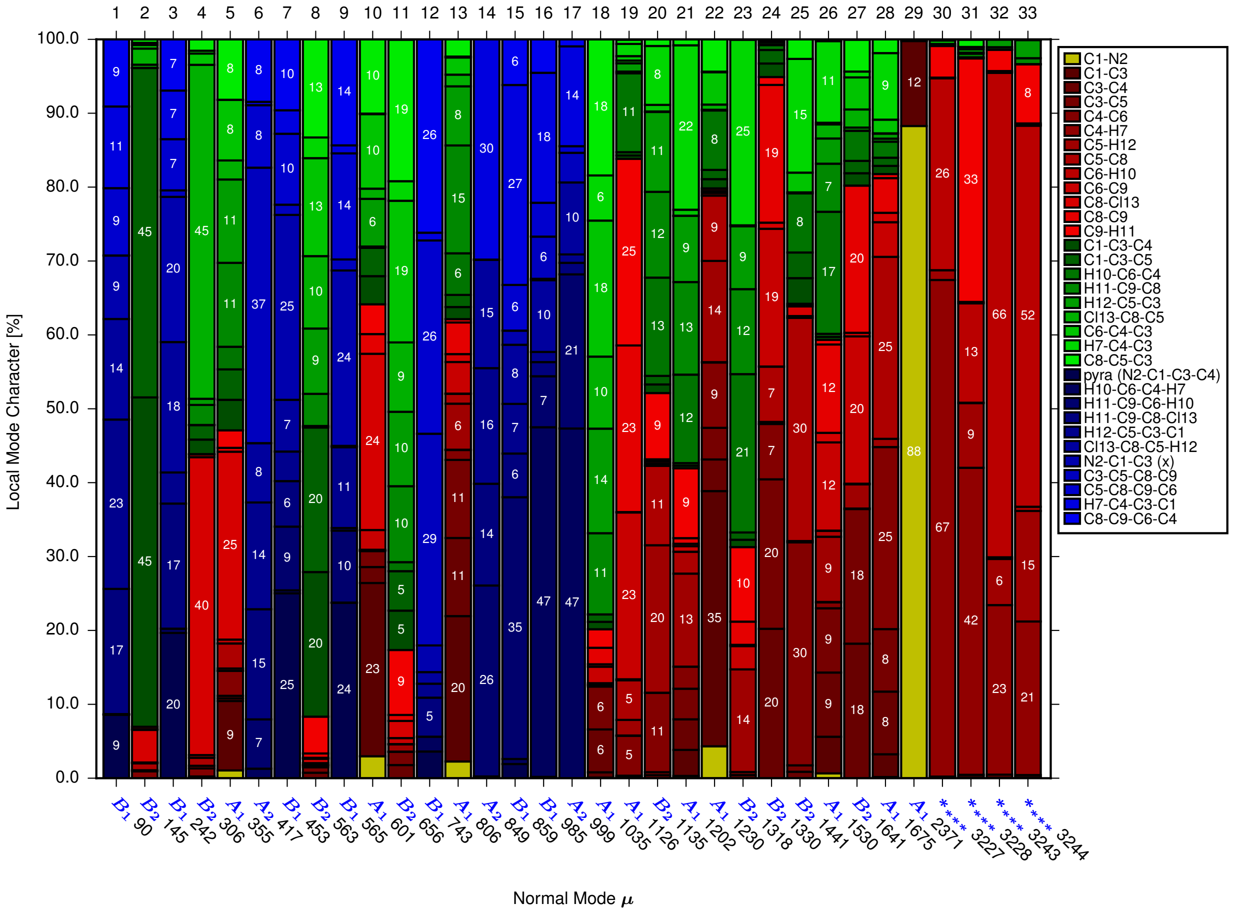

Figure 51: Decomposition of normal mode frequencies for 2-13

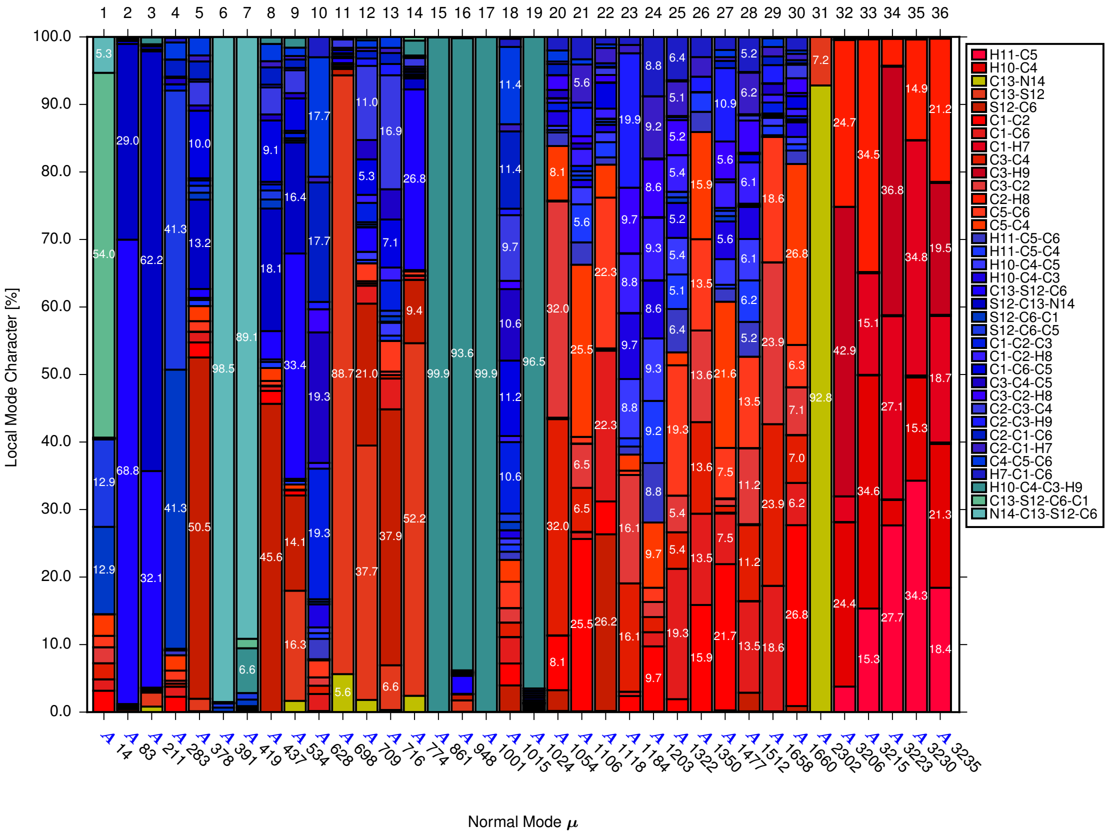

Figure 52: Decomposition of normal mode frequencies for 2-14

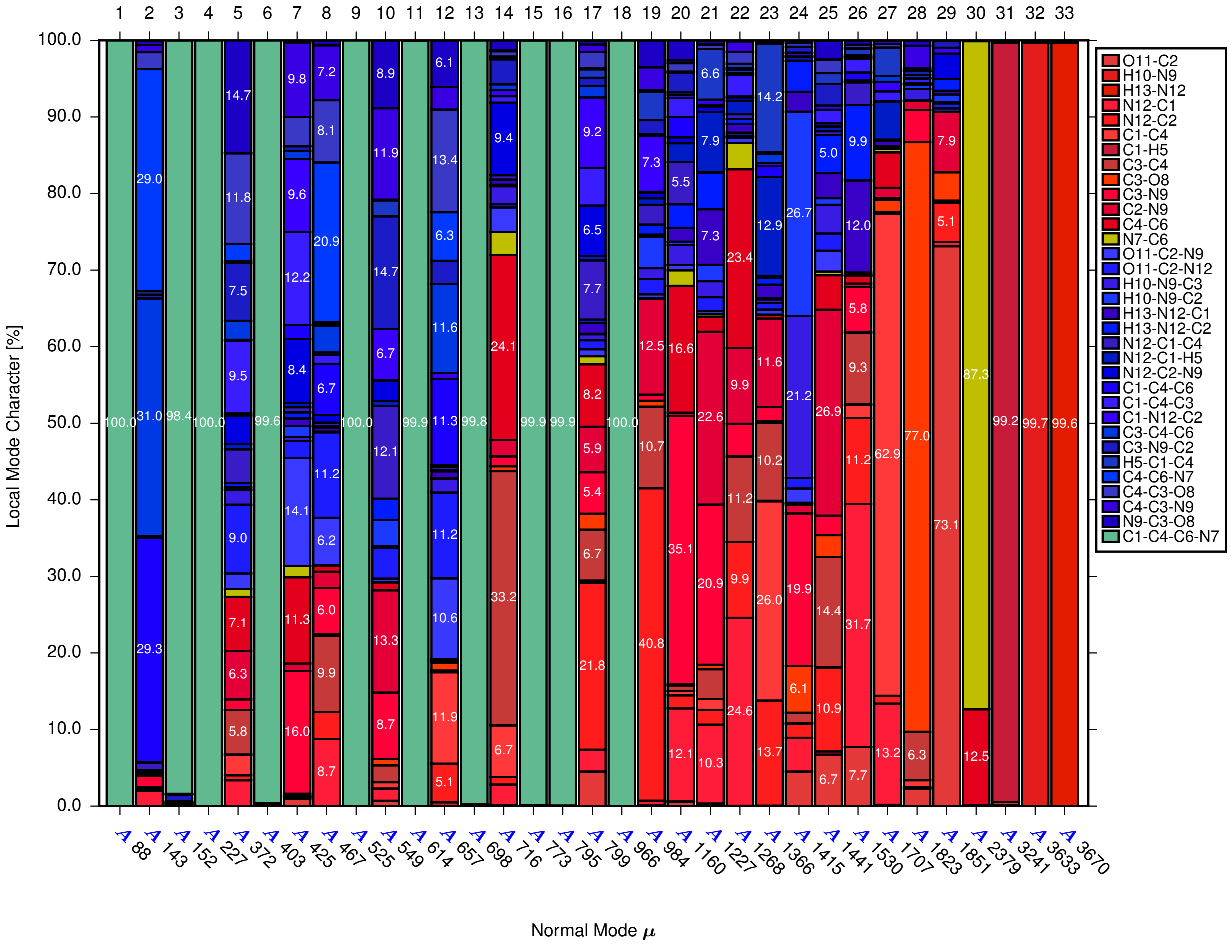

Figure 53: Decomposition of normal mode frequencies for 2-15

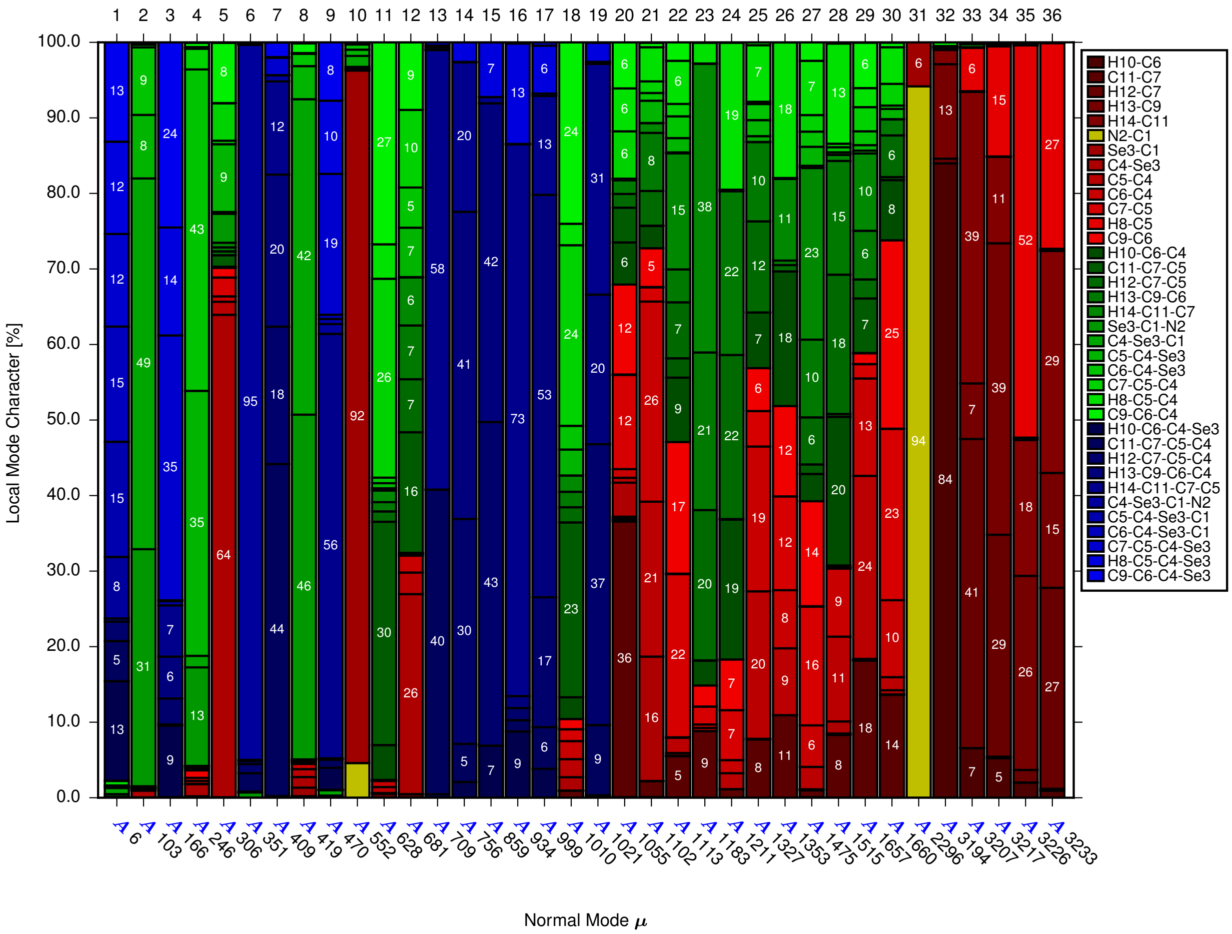

Figure 54: Decomposition of normal mode frequencies for 2-16

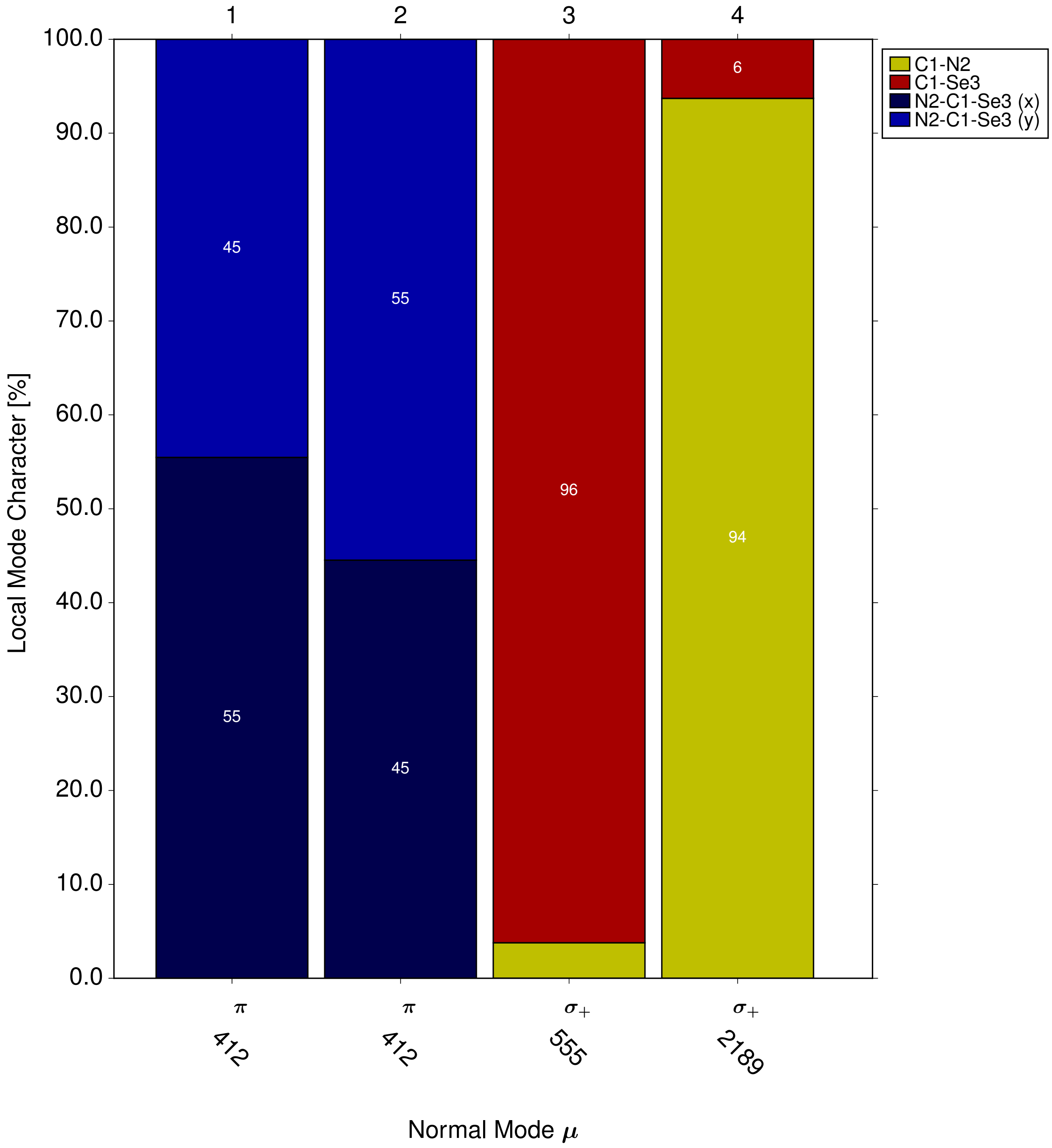

Figure 55: Decomposition of normal mode frequencies for 2-17

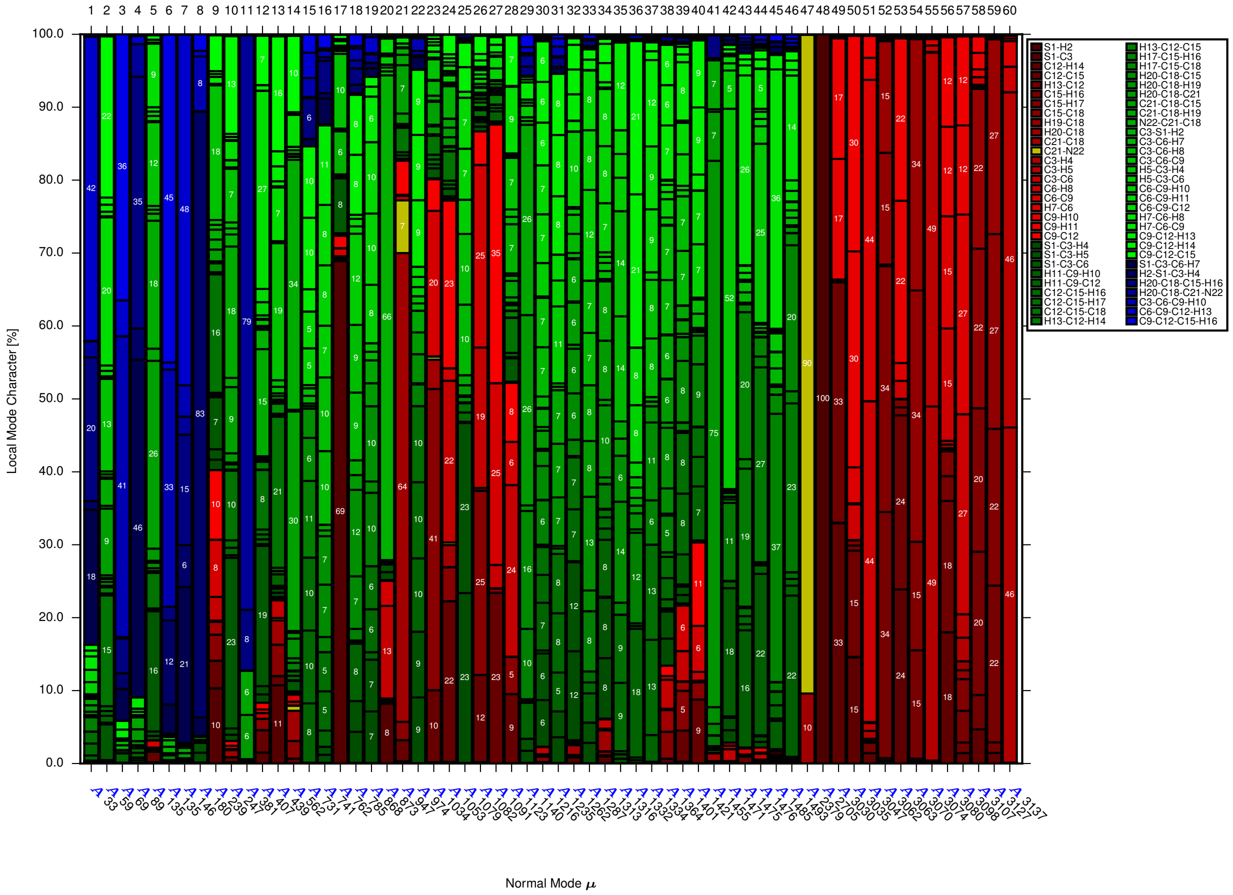

Figure 56: Decomposition of normal mode frequencies for 2-18

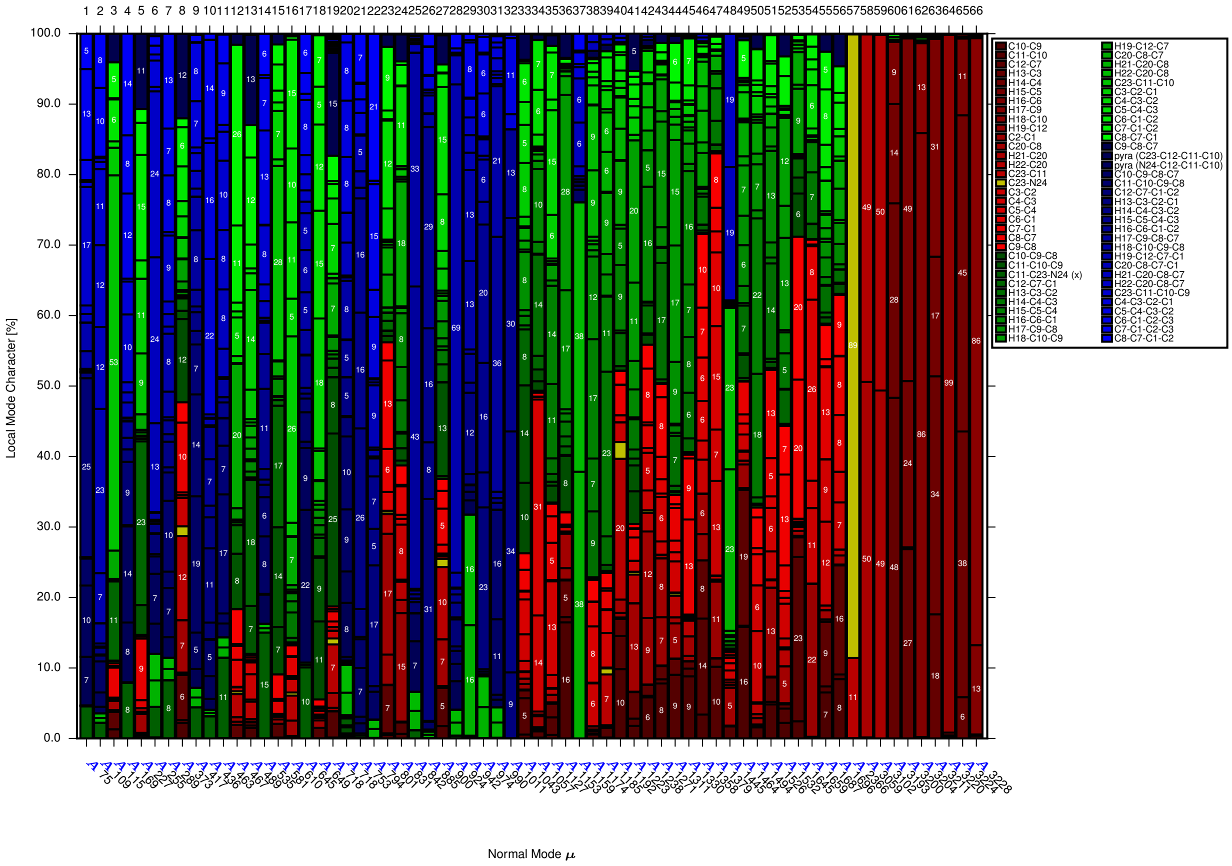

Figure 57: Decomposition of target normal mode frequency (C≡N bond) for 2-19

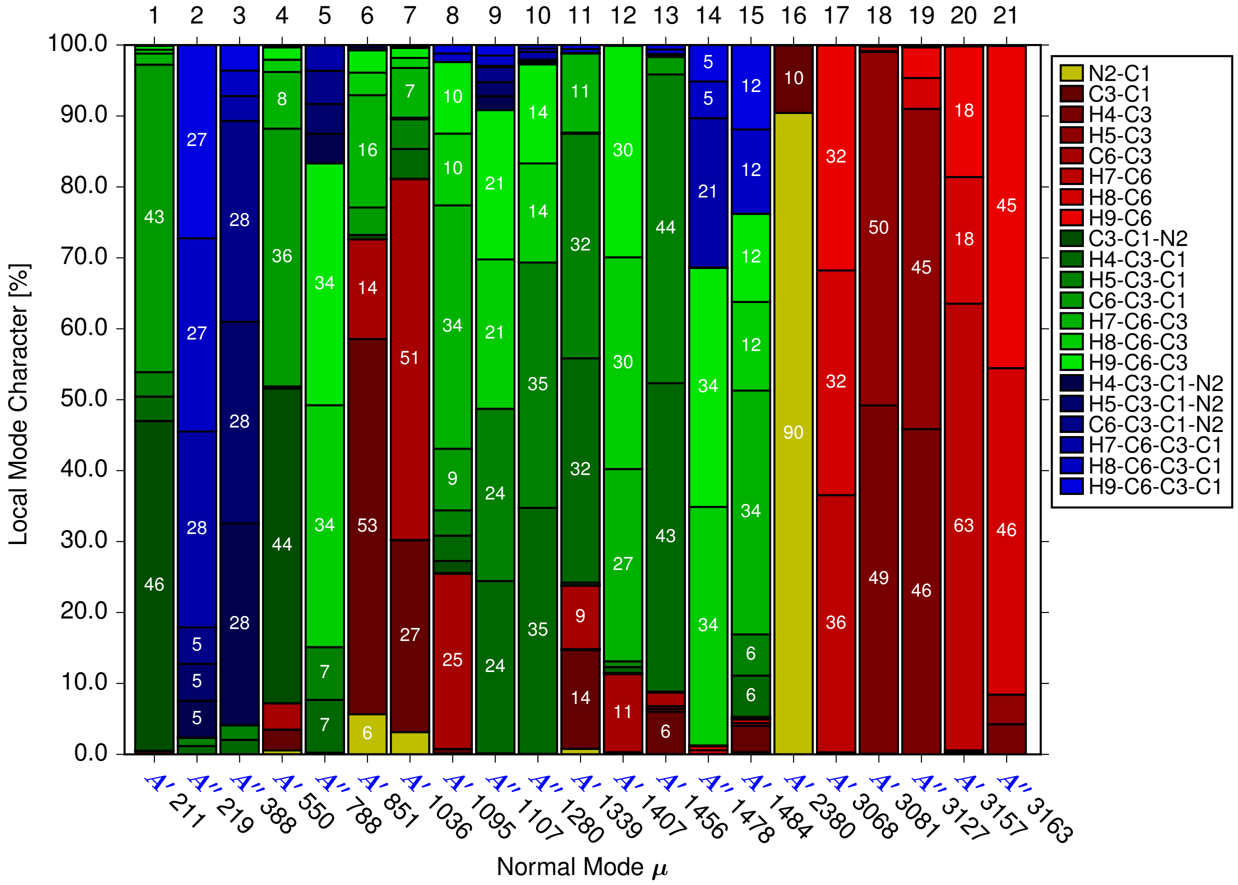

Figure 58: Decomposition of normal mode frequencies for 2-20

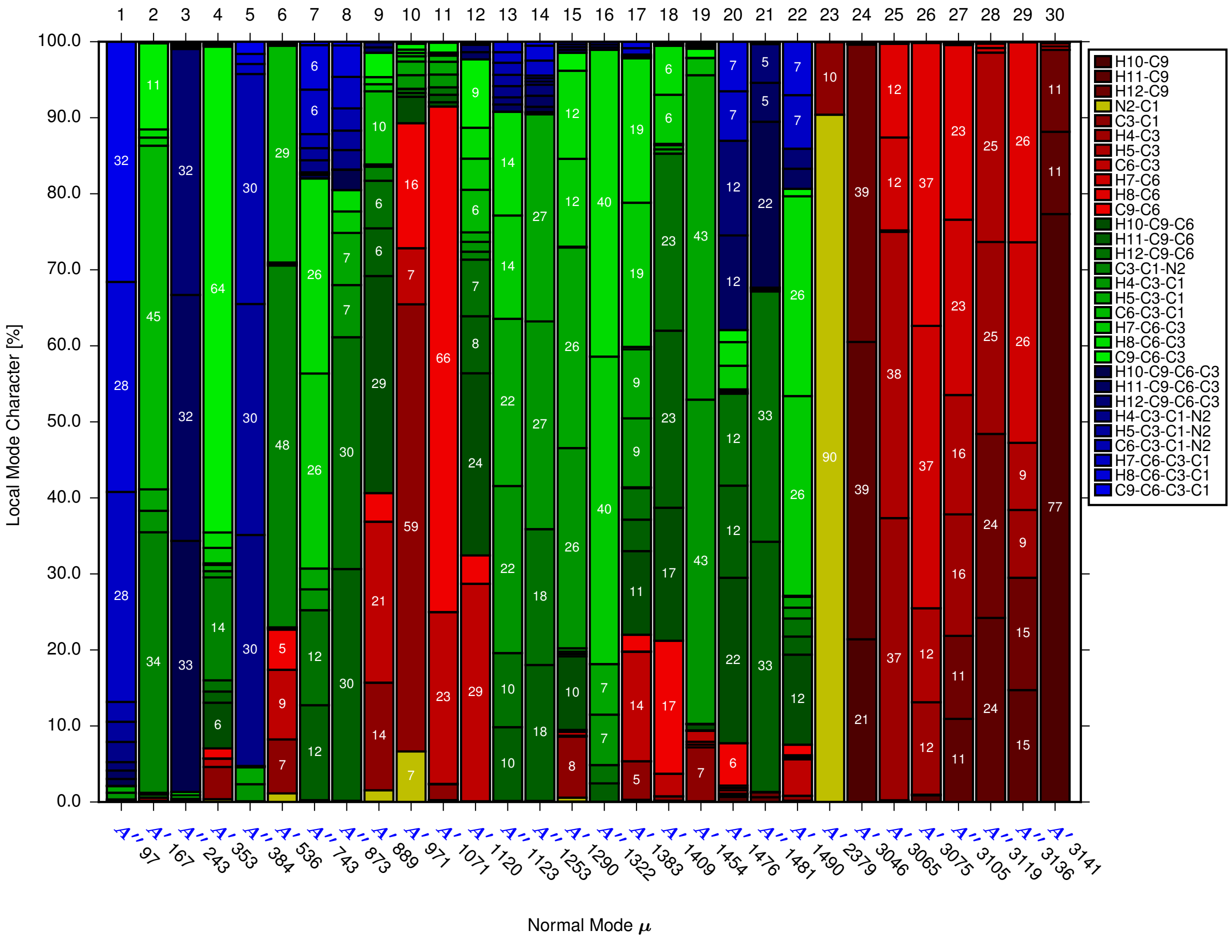

Figure 59: Decomposition of normal mode frequencies for 2-21

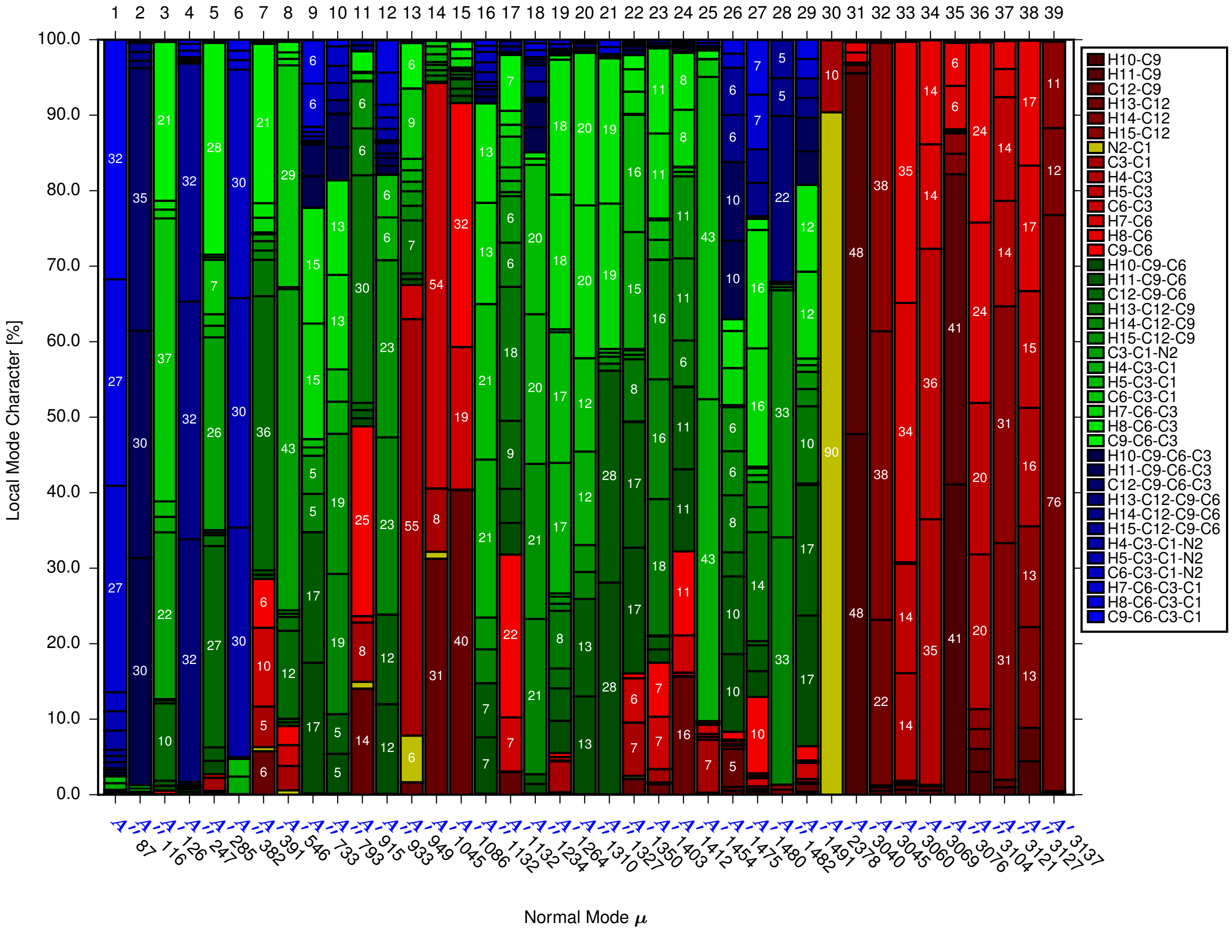

Figure 60: Decomposition of normal mode frequencies for 2-22

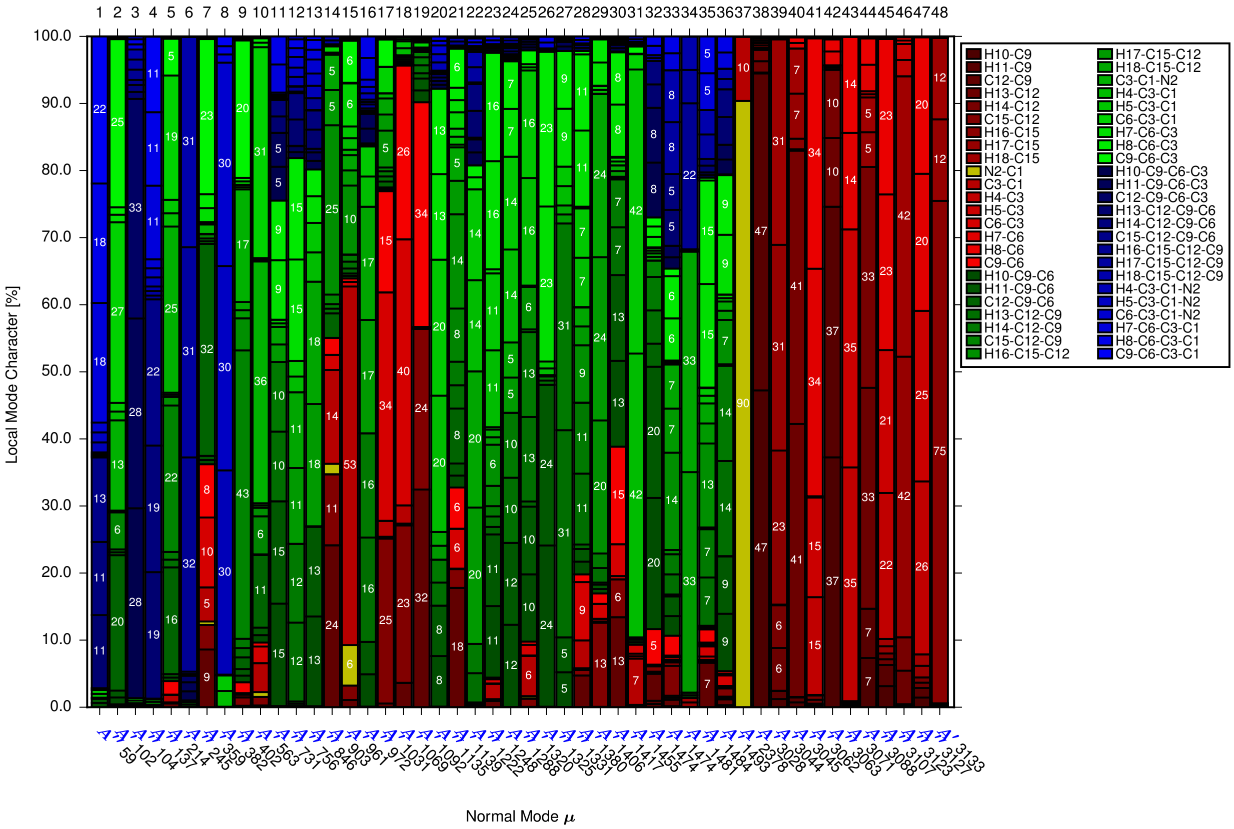

Figure 61: Decomposition of normal mode frequencies for 2-23

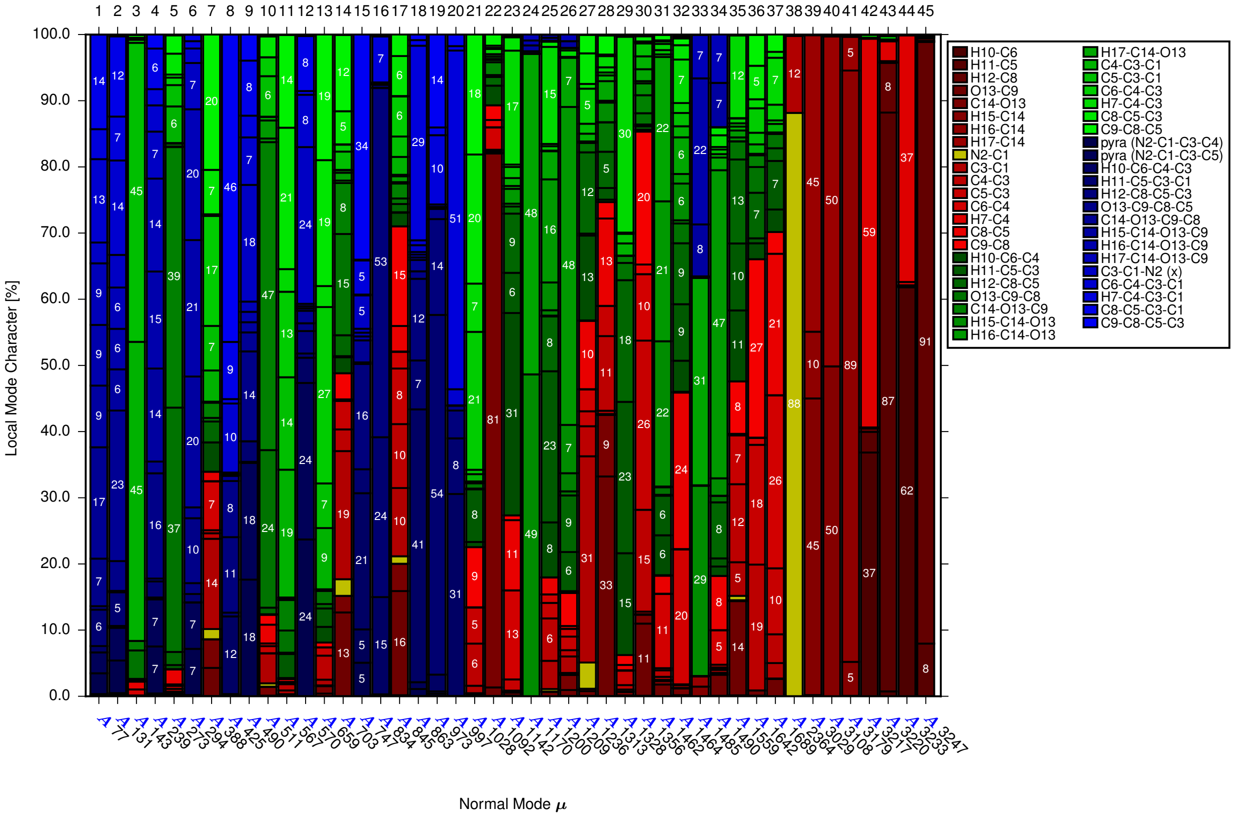

Figure 62: Decomposition of normal mode frequencies for 2-24

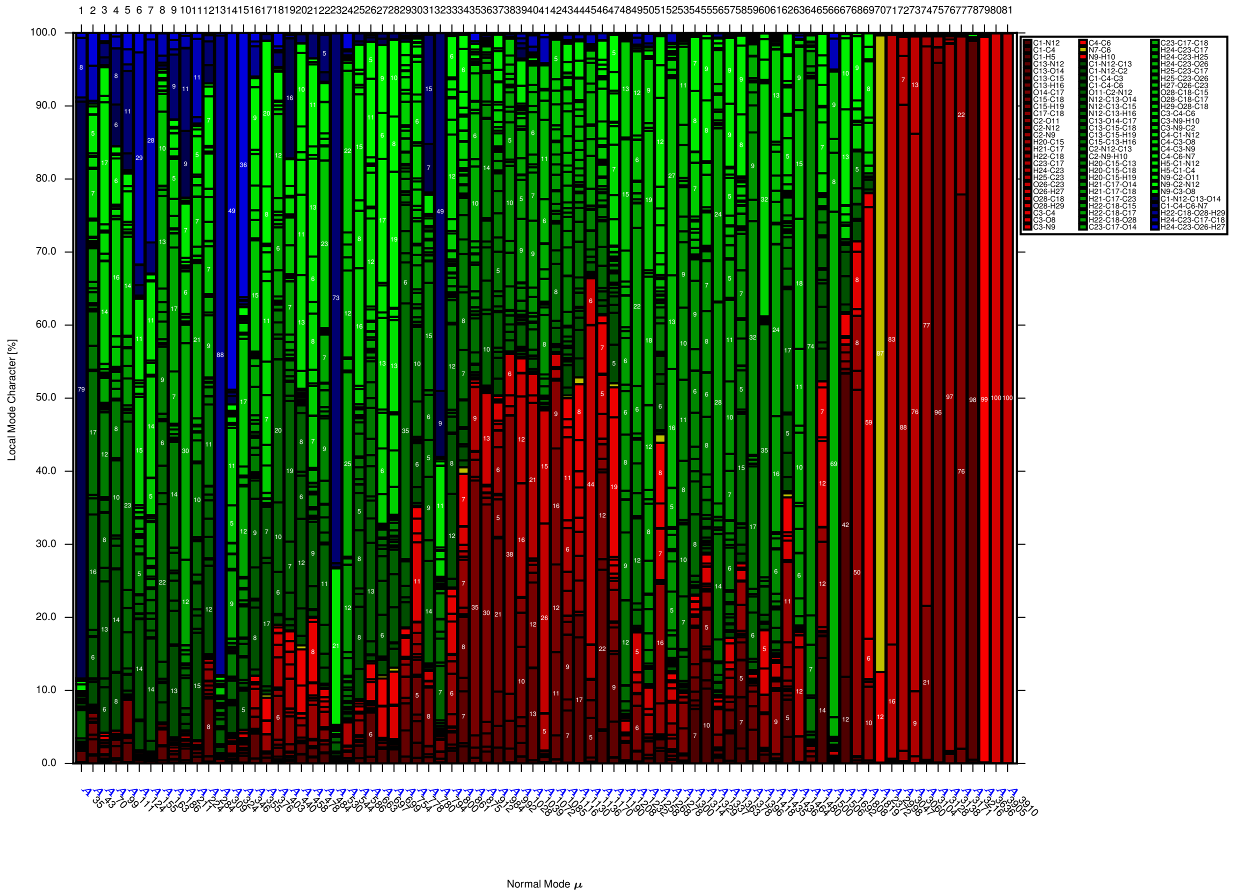

Figure 63: Decomposition of target normal mode frequency (C≡N bond) for 2-25

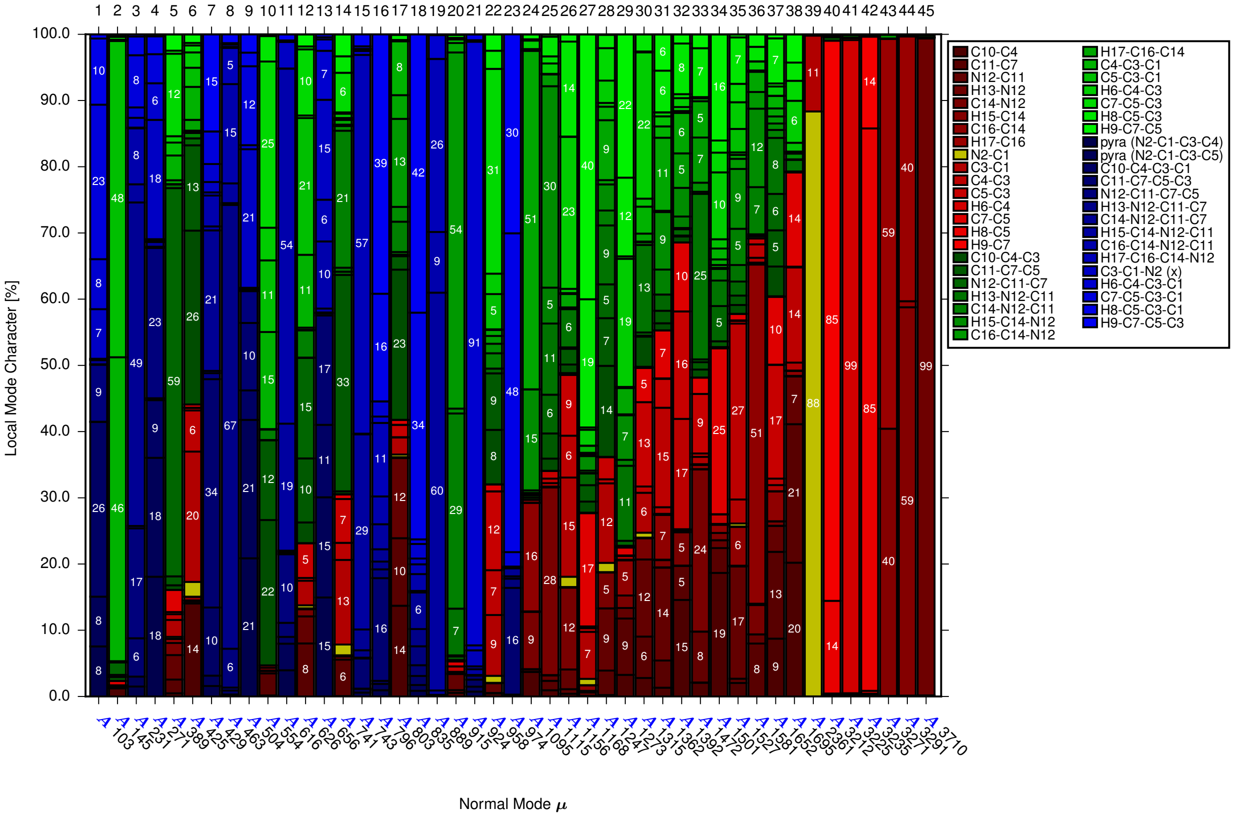

Figure 64: Decomposition of normal mode frequencies for 2-26

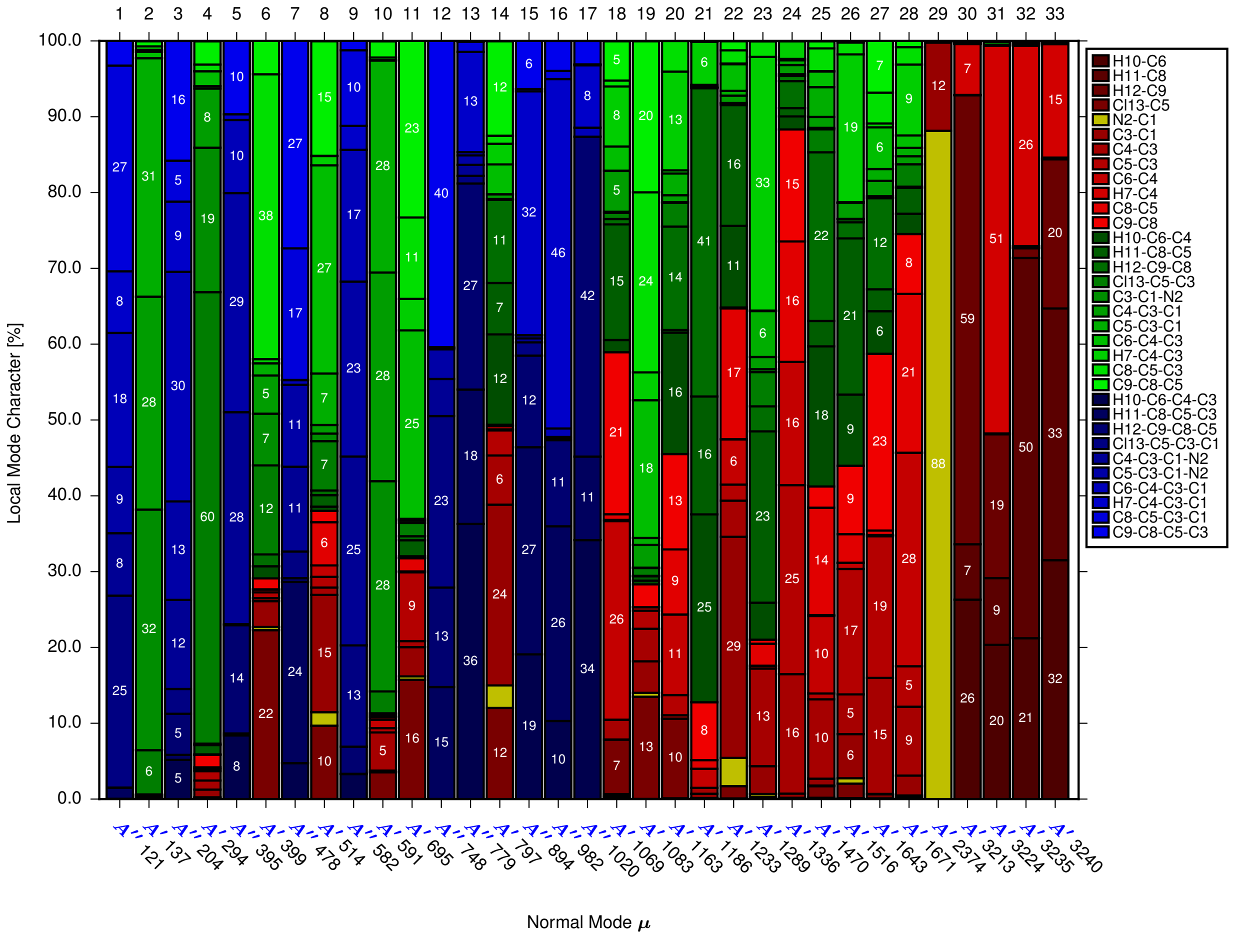

Figure 65: Decomposition of normal mode frequencies for 2-27

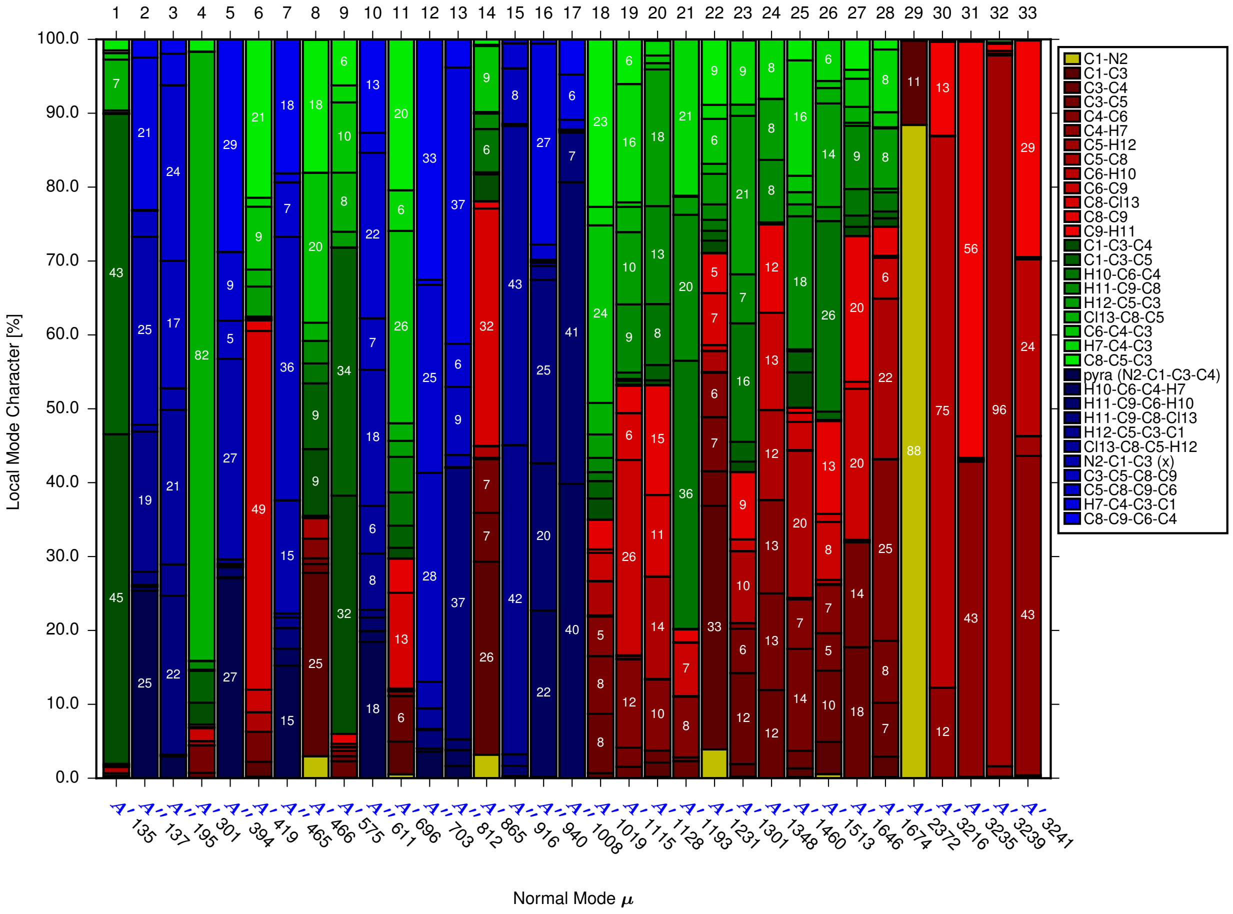

Figure 66: Decomposition of normal mode frequencies for 2-28

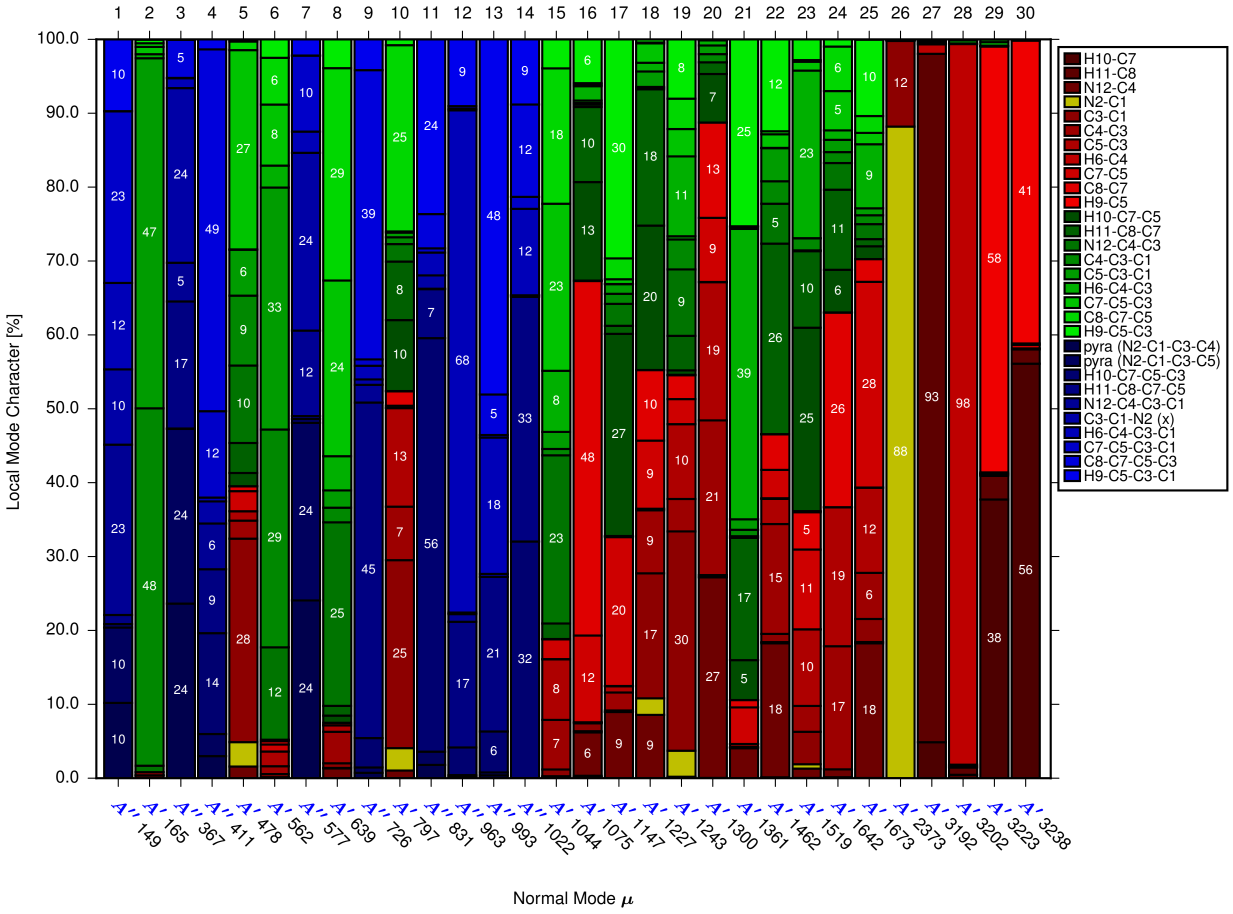

Figure 67: Decomposition of normal mode frequencies for 2-29

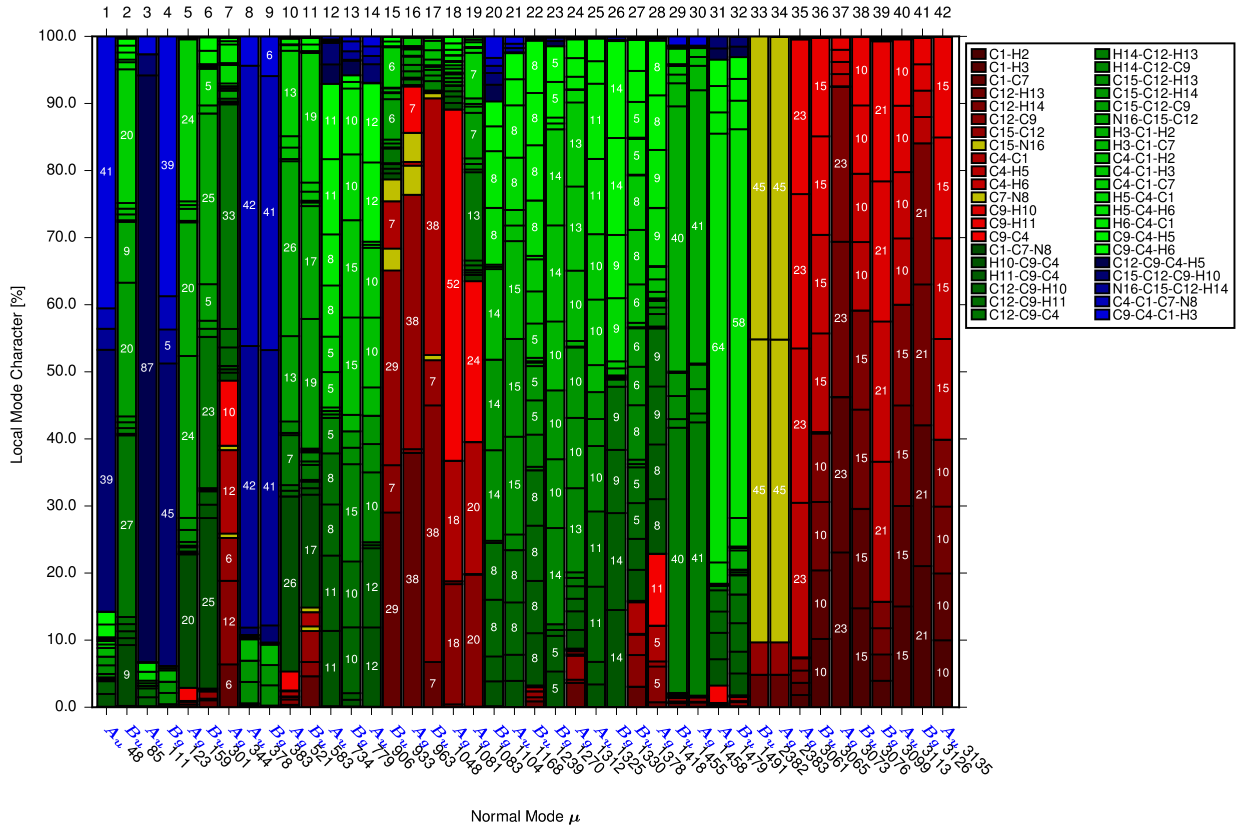

Figure 68: Decomposition of normal mode frequencies for 2-30

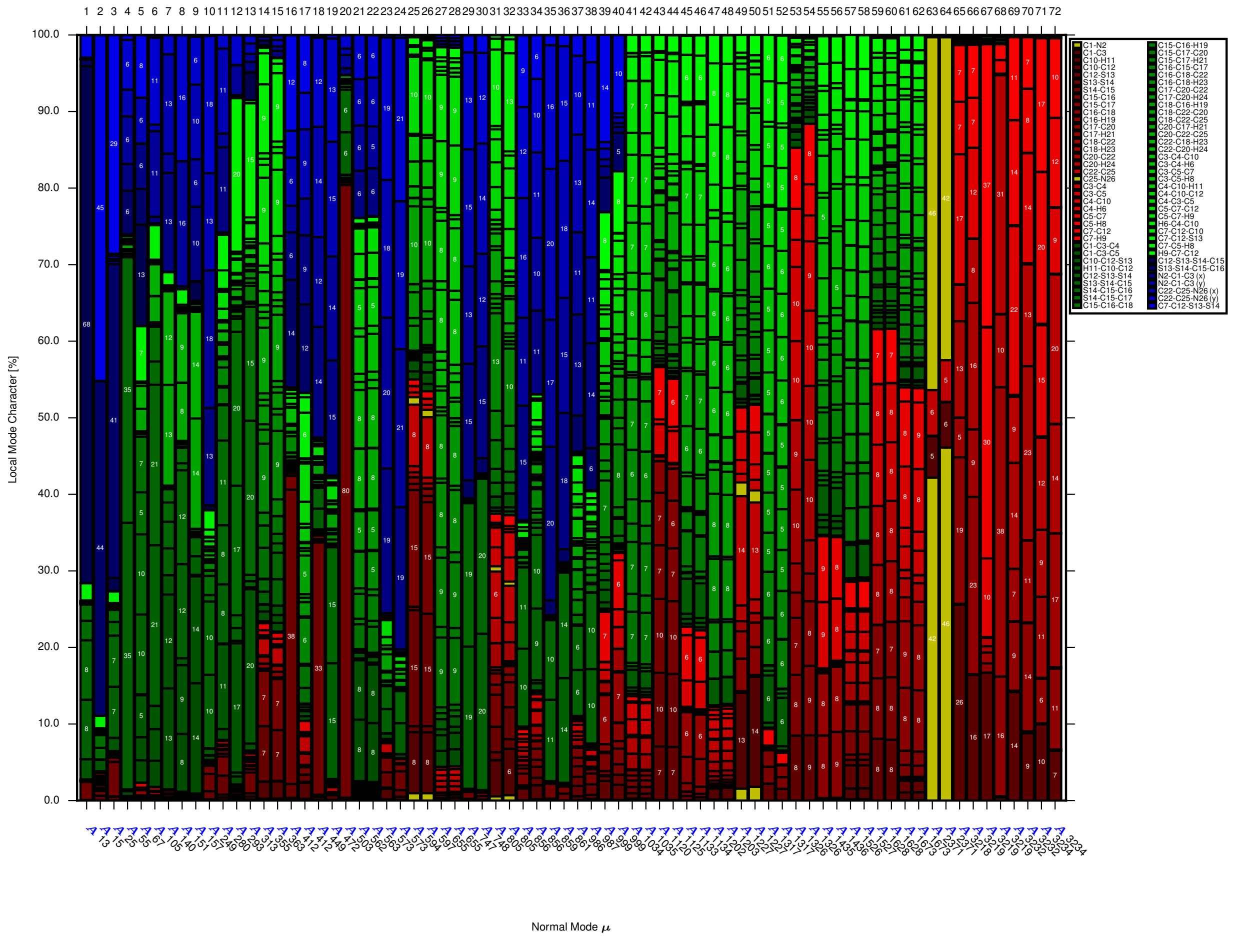

Figure 69: Decomposition of normal mode frequencies for 2-31

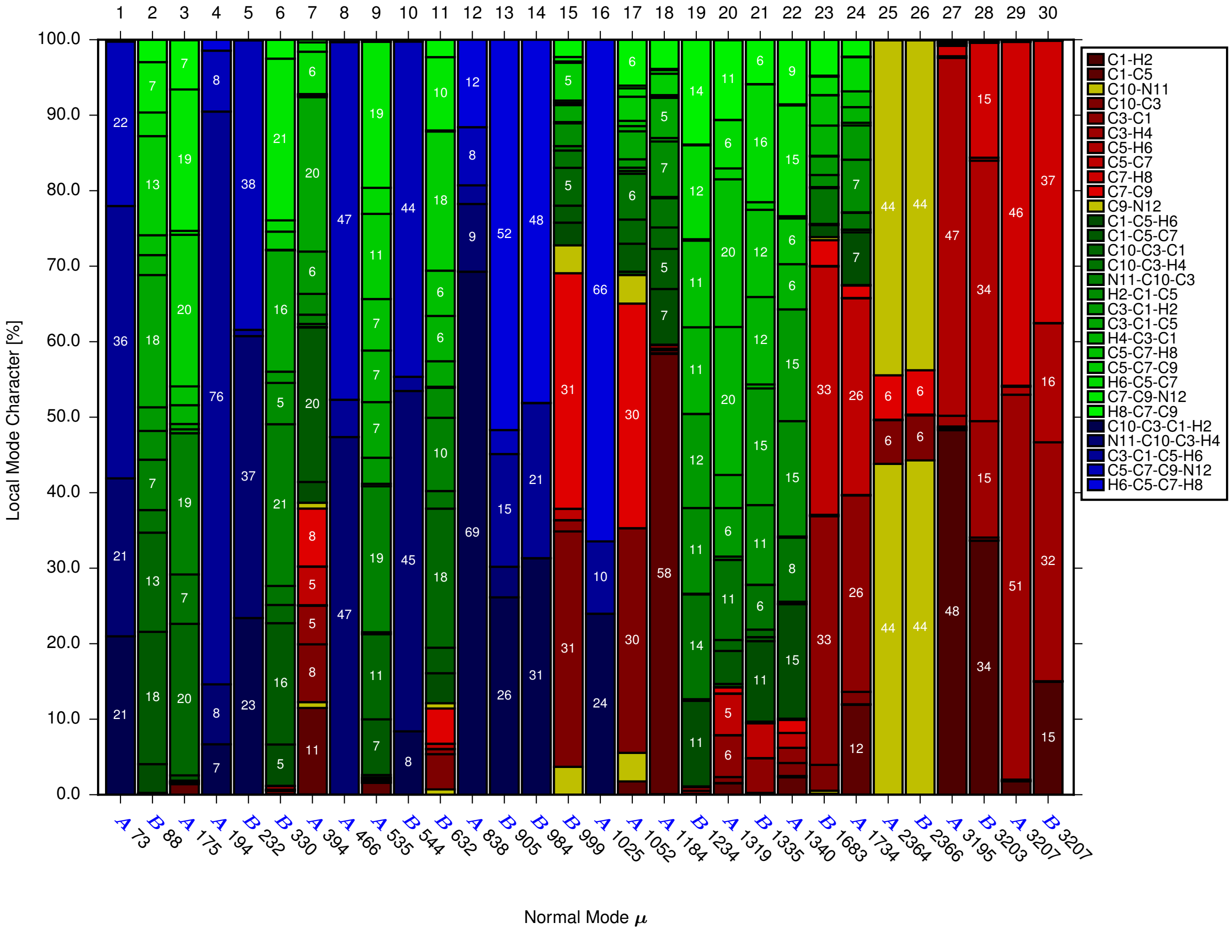

Figure 70: Decomposition of normal mode frequencies for 2-32

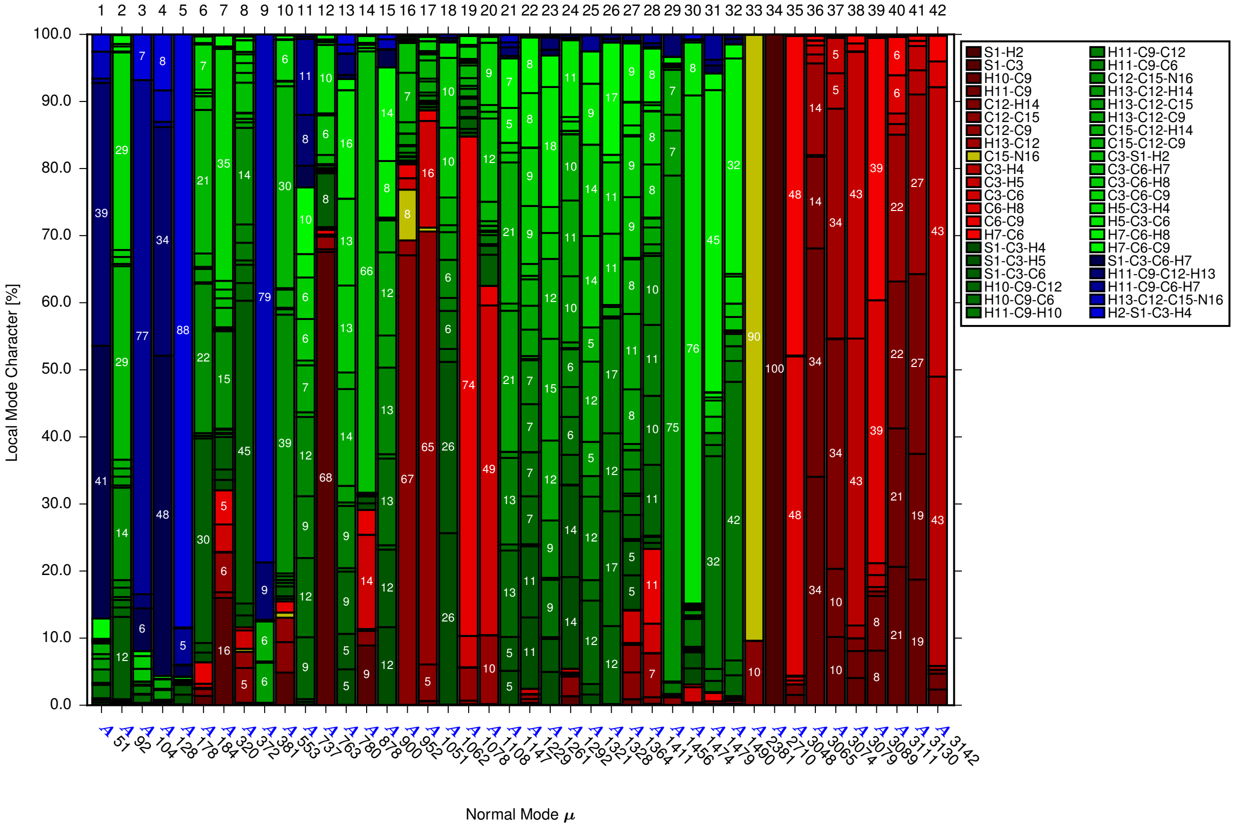

Figure 71: Decomposition of normal mode frequencies for 2-33

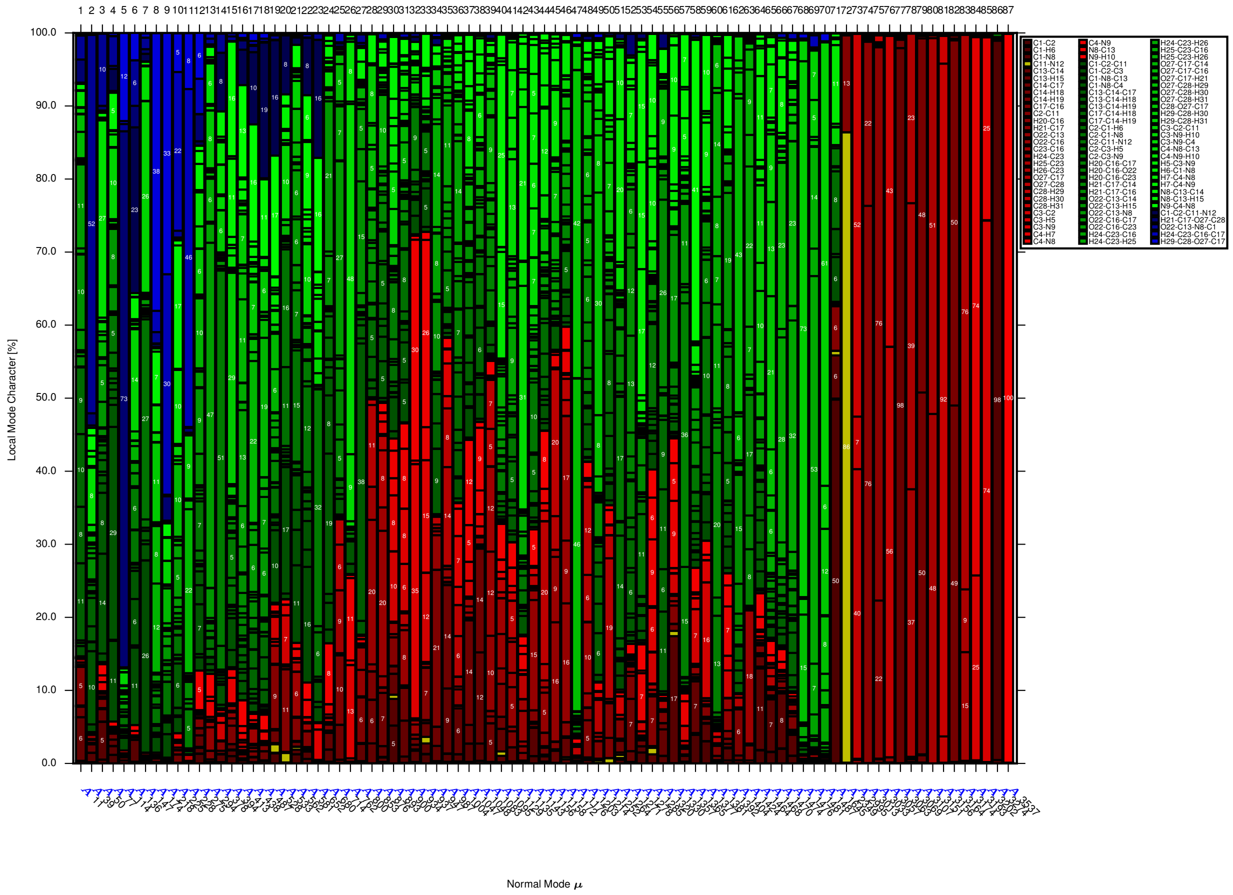

Figure 72: Decomposition of target normal mode frequency (C≡N bond) for 2-34

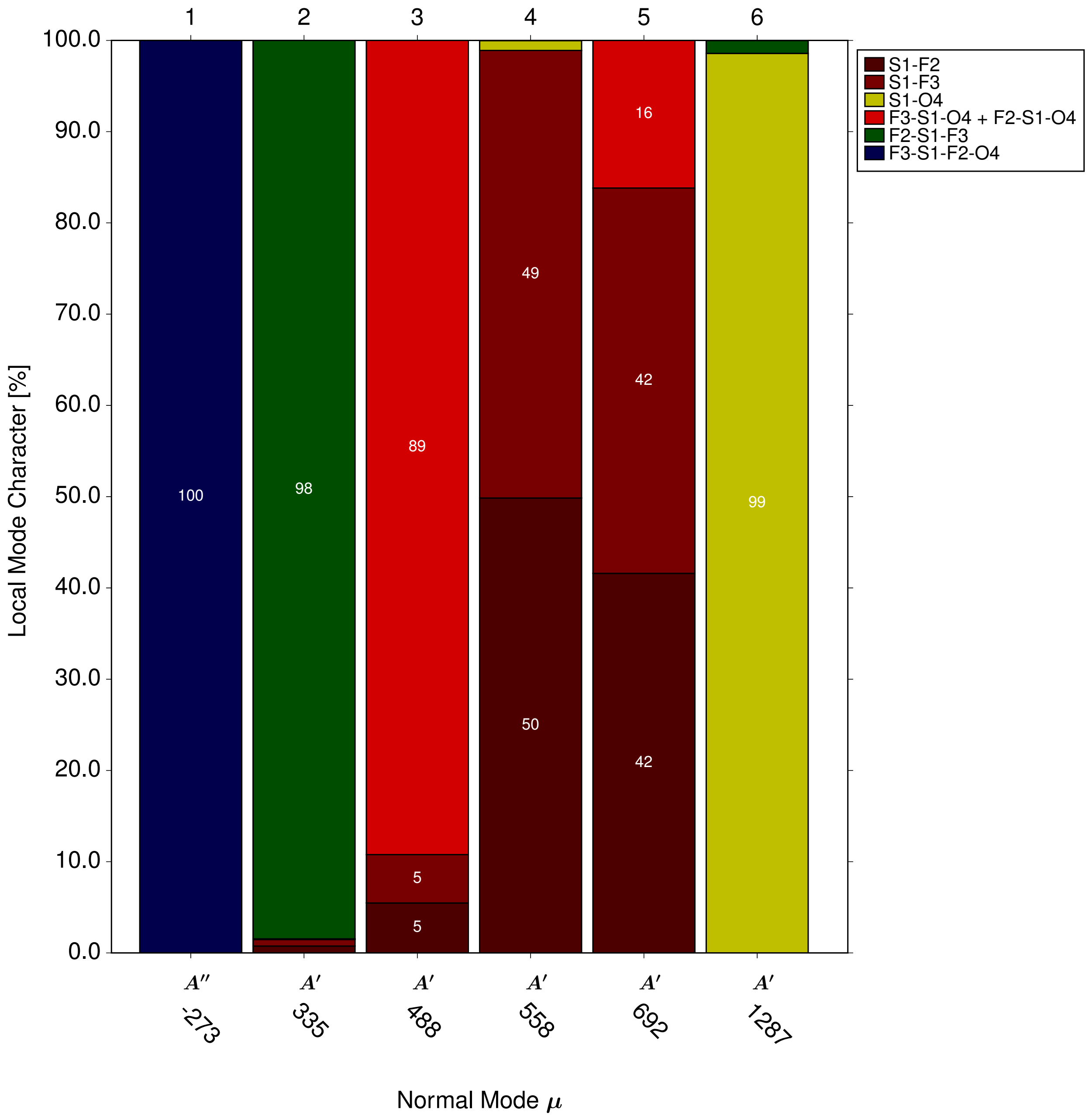

Figure 73: Decomposition of normal mode frequencies for 3-1

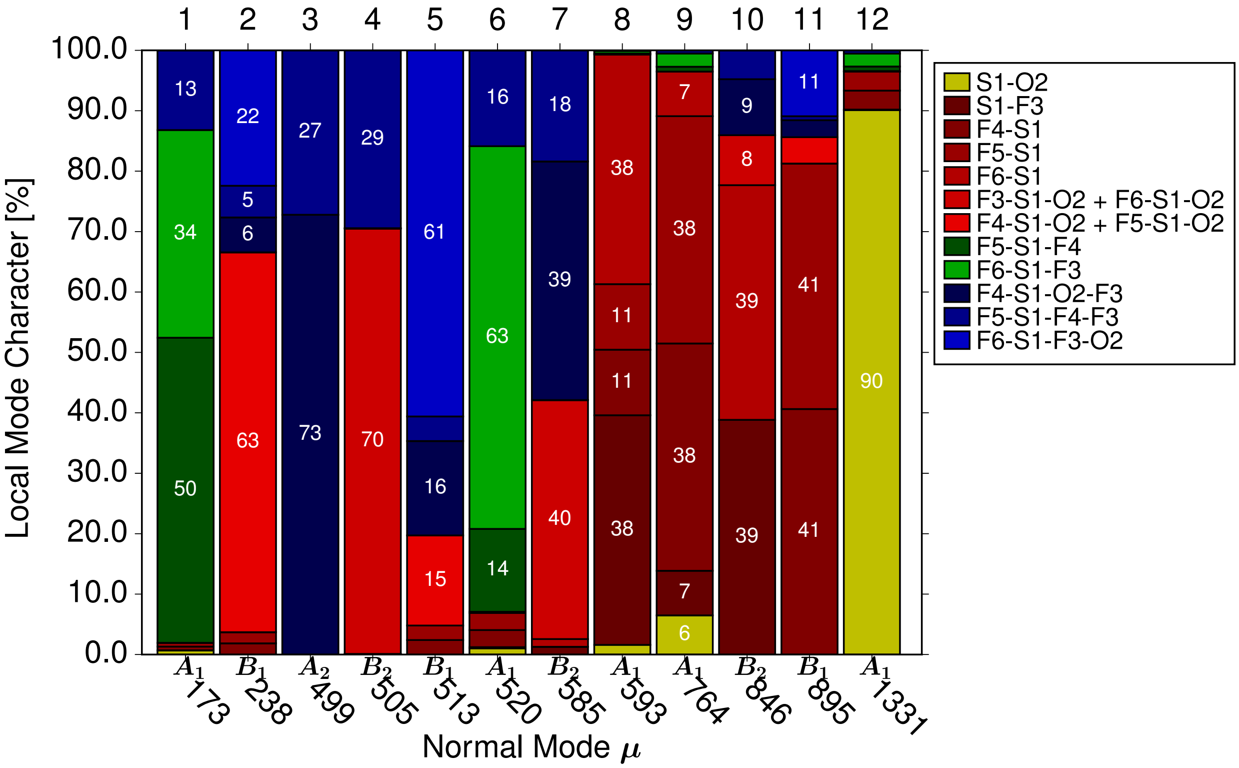

Figure 74: Decomposition of normal mode frequencies for 3-2

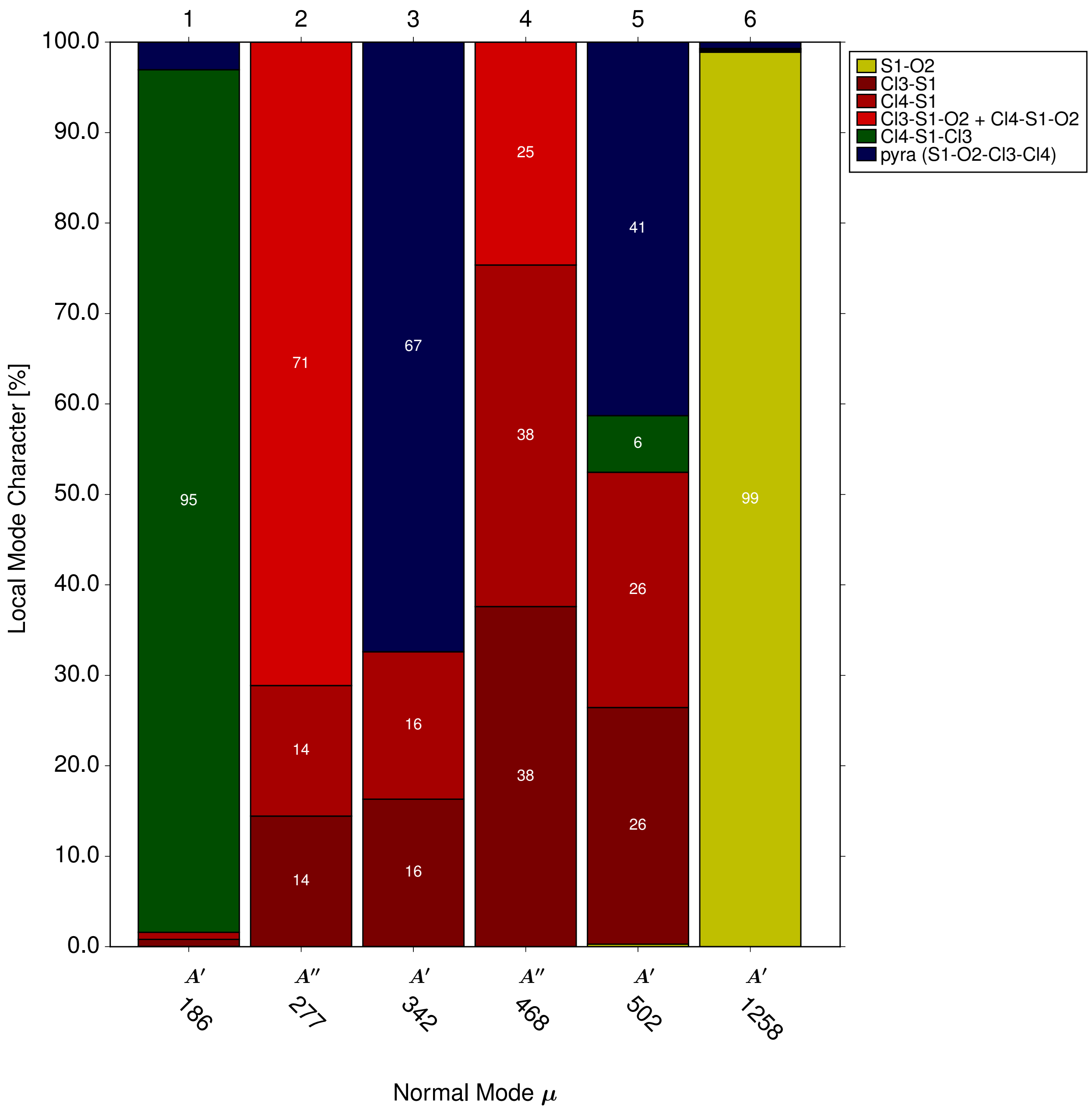

Figure 75: Decomposition of normal mode frequencies for 3-3

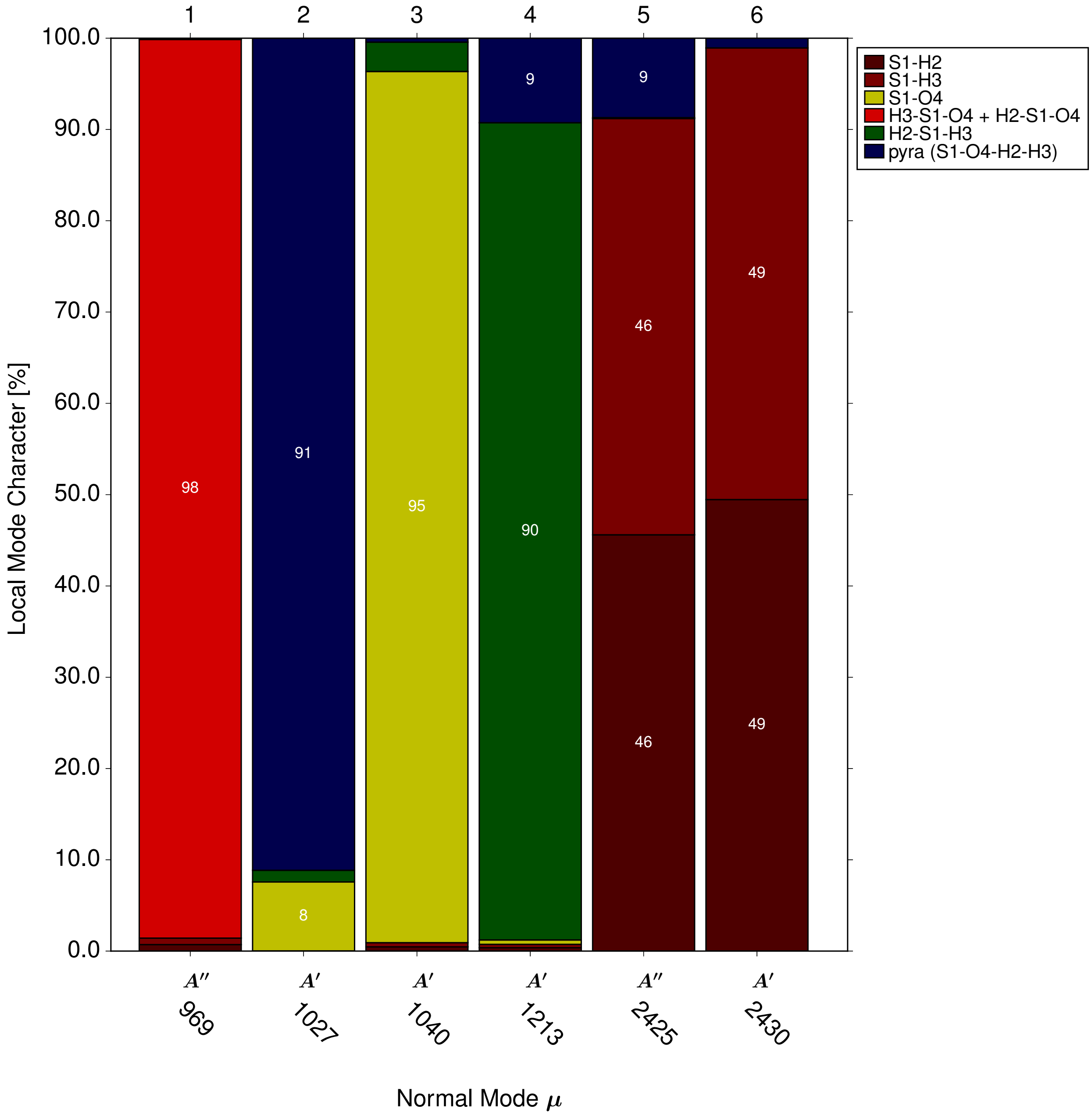

Figure 76: Decomposition of normal mode frequencies for 3-4

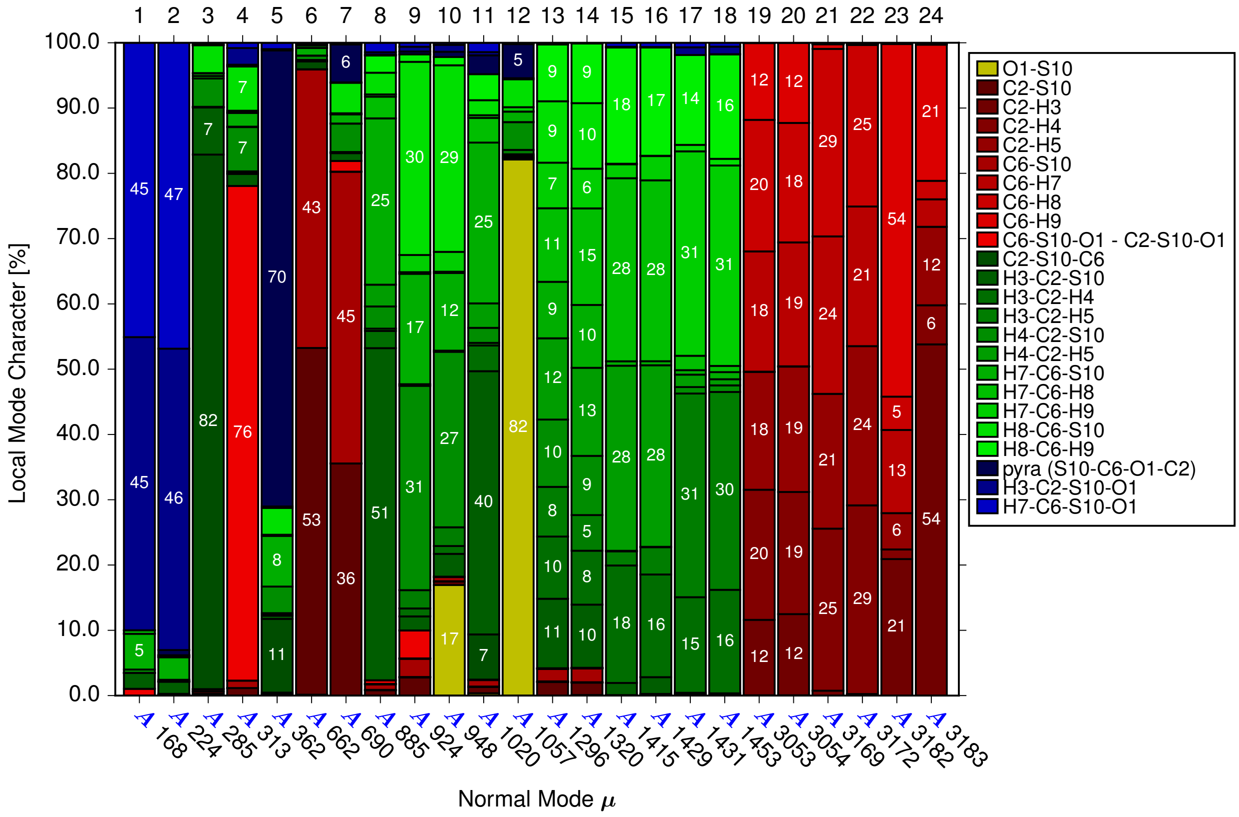

Figure 77: Decomposition of normal mode frequencies for 3-5

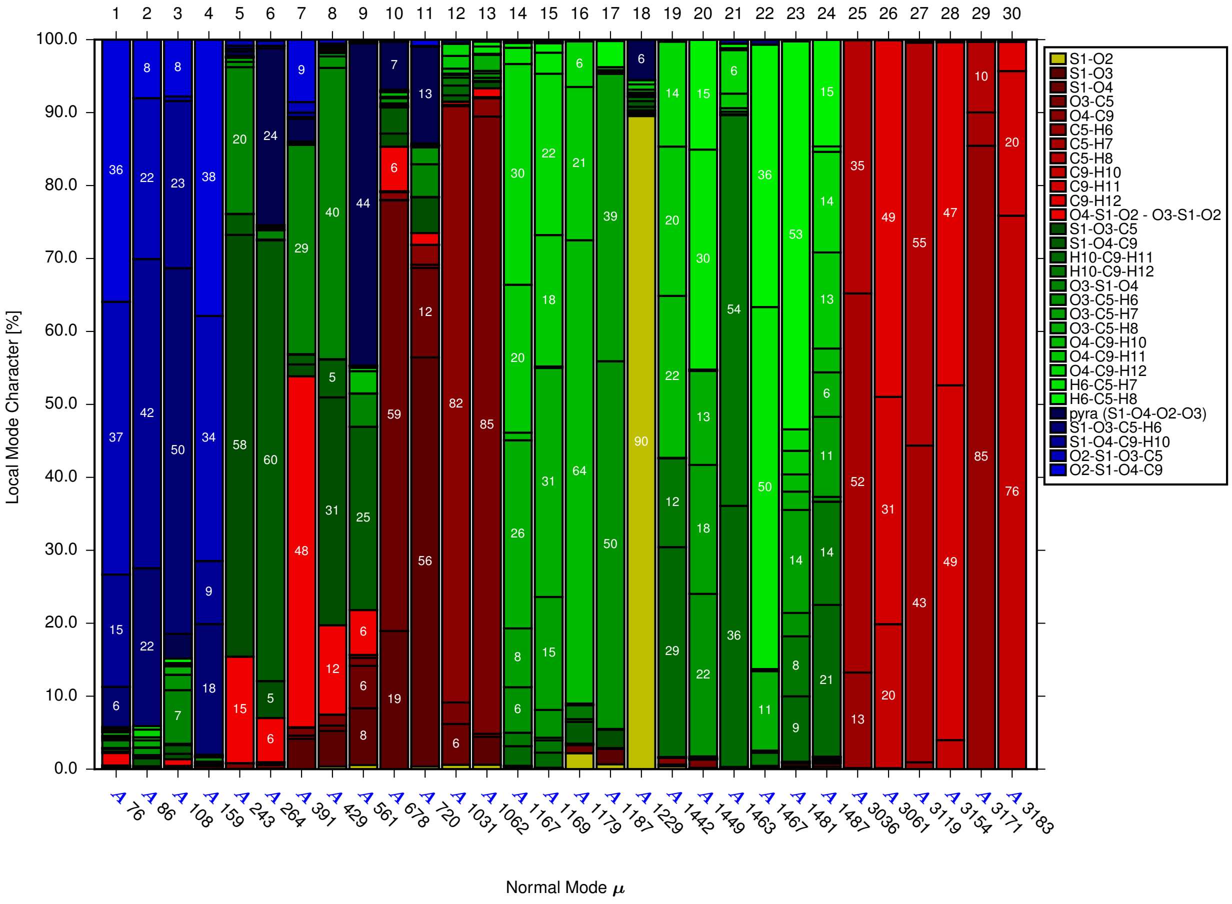

Figure 78: Decomposition of normal mode frequencies for 3-6

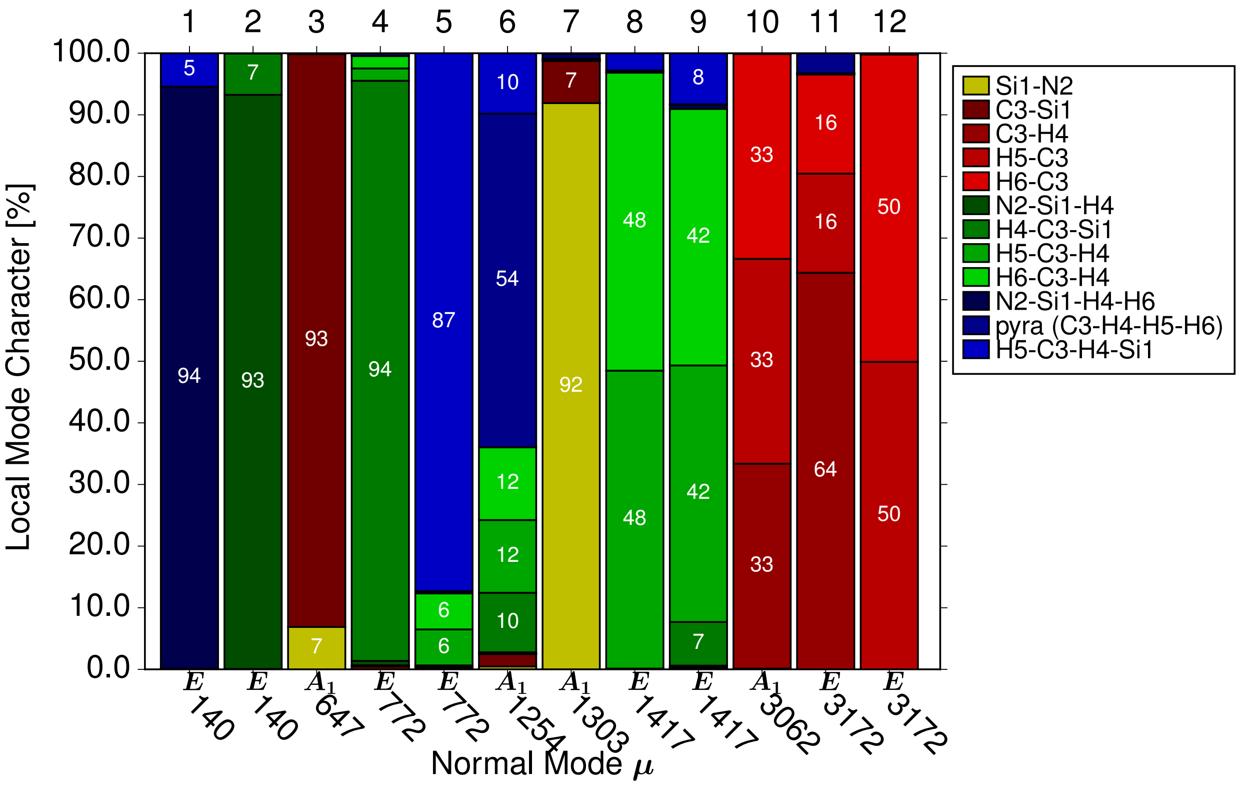

Figure 79: Decomposition of normal mode frequencies for 4-1

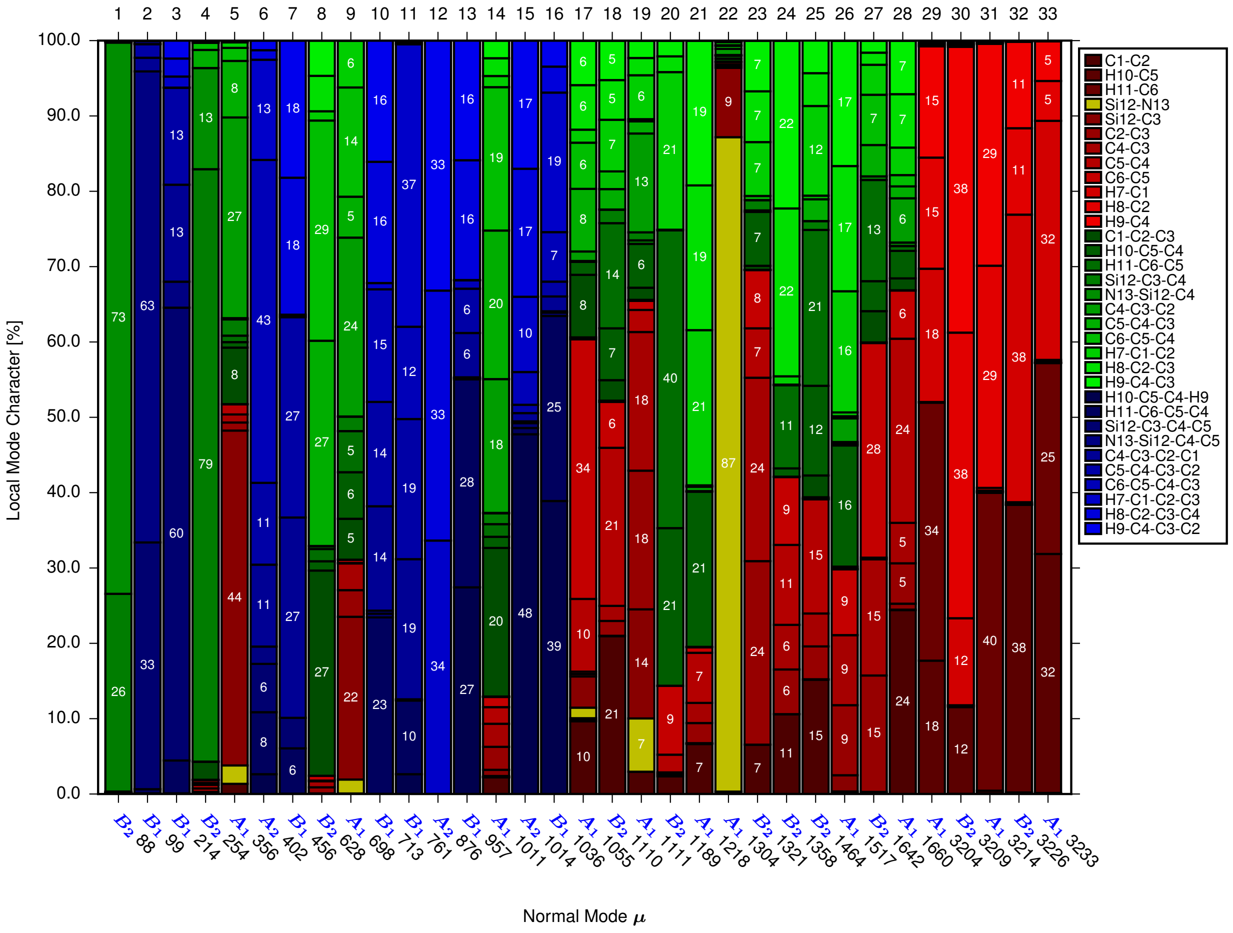

Figure 80: Decomposition of normal mode frequencies for 4-2

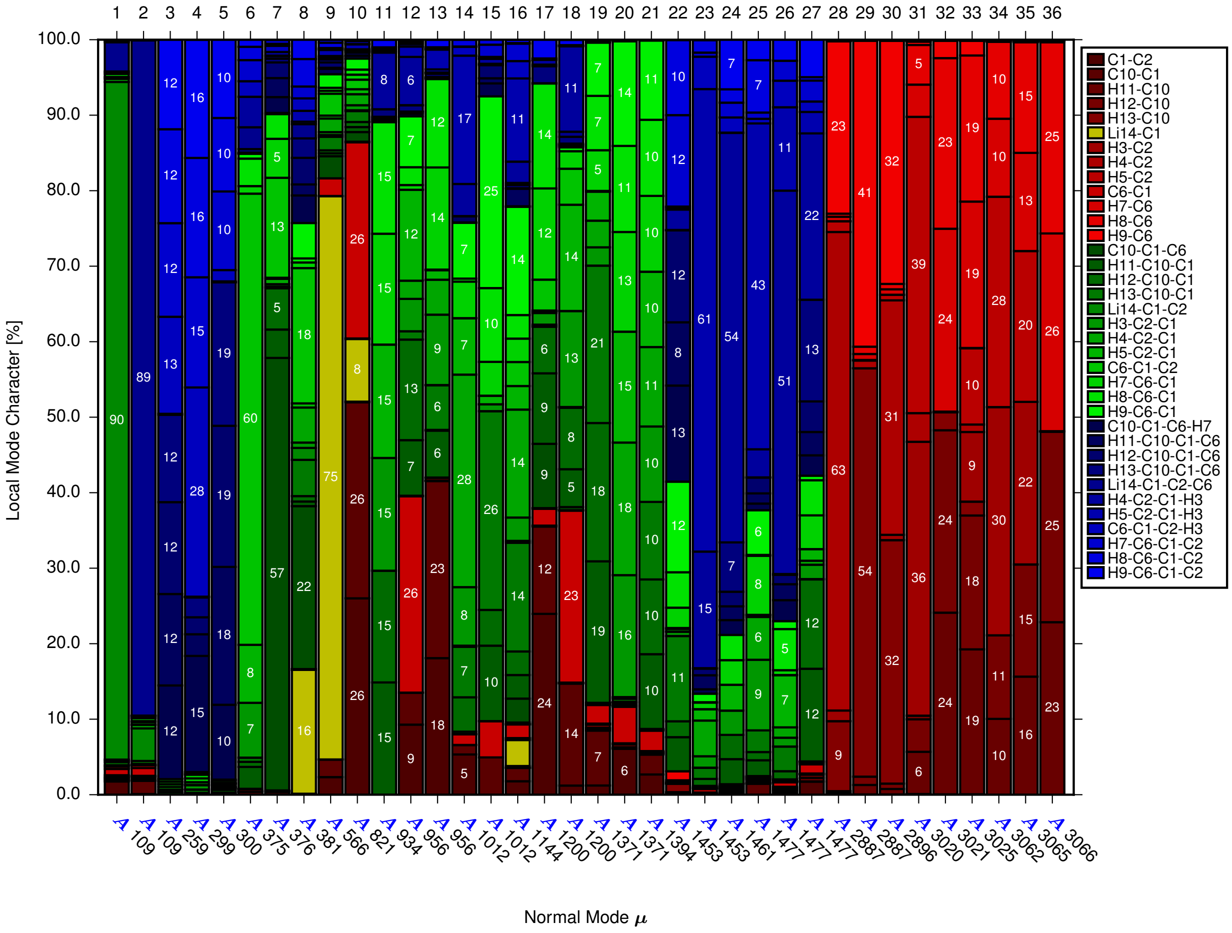

Figure 81: Decomposition of normal mode frequencies for 4-3

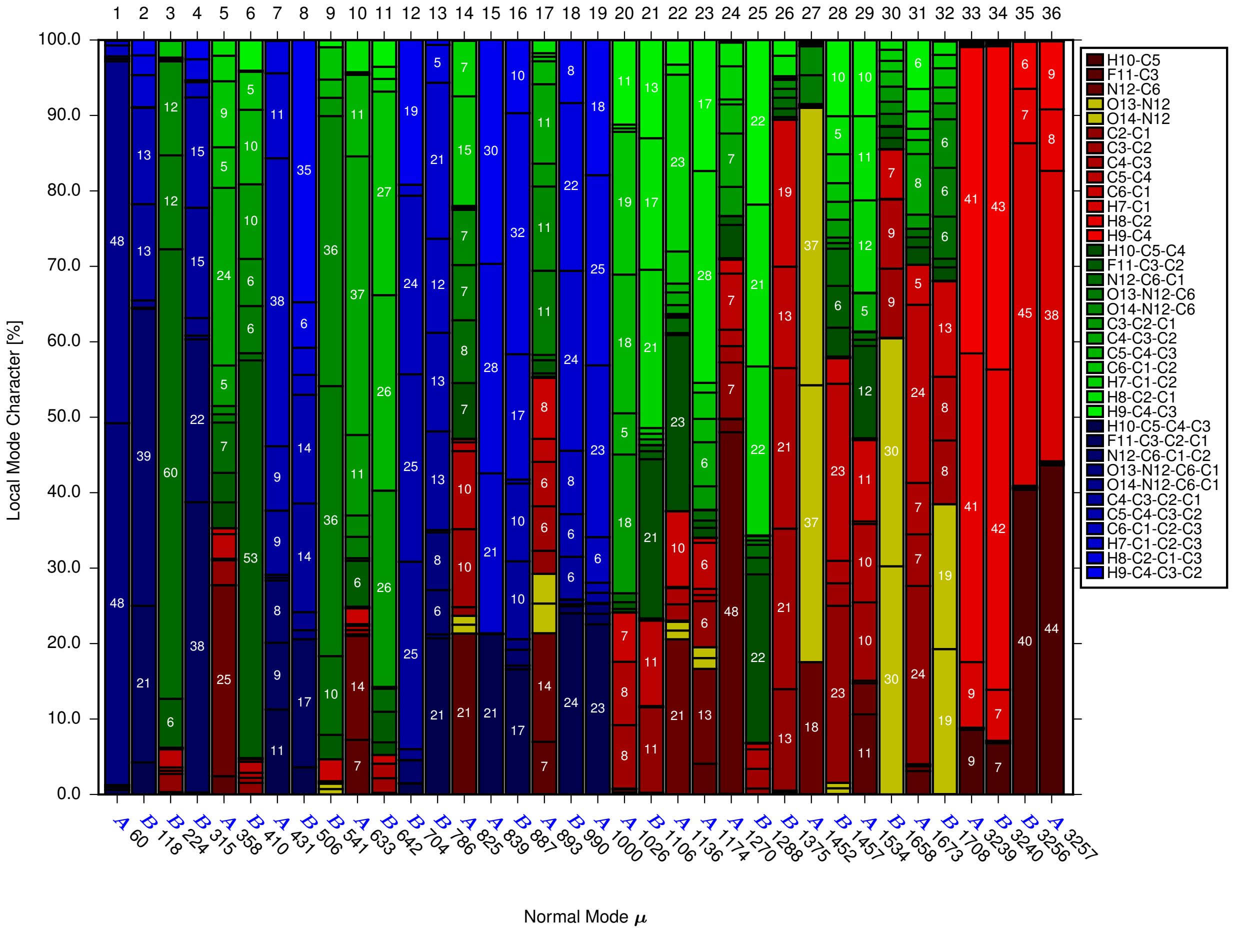

Figure 82: Decomposition of normal mode frequencies for 4-4

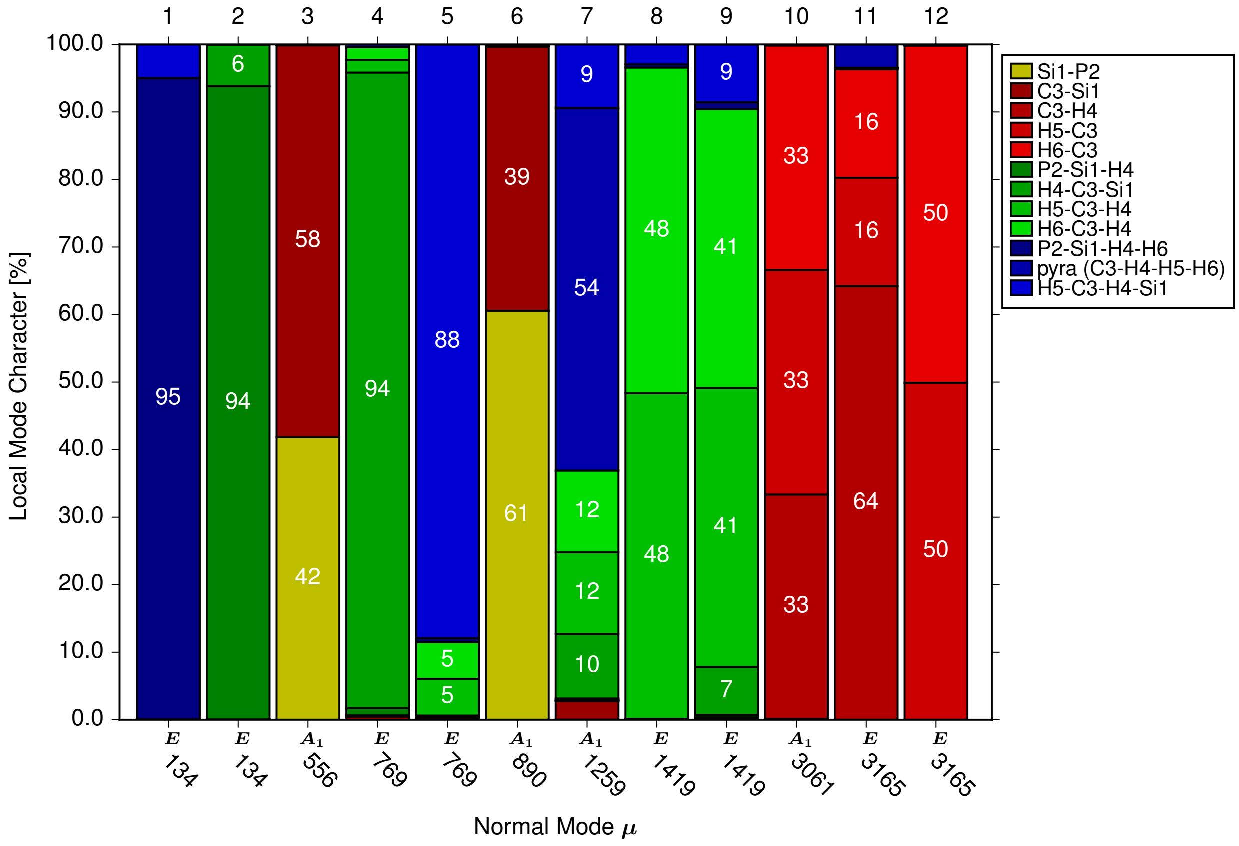

Figure 83: Decomposition of normal mode frequencies for 4-5

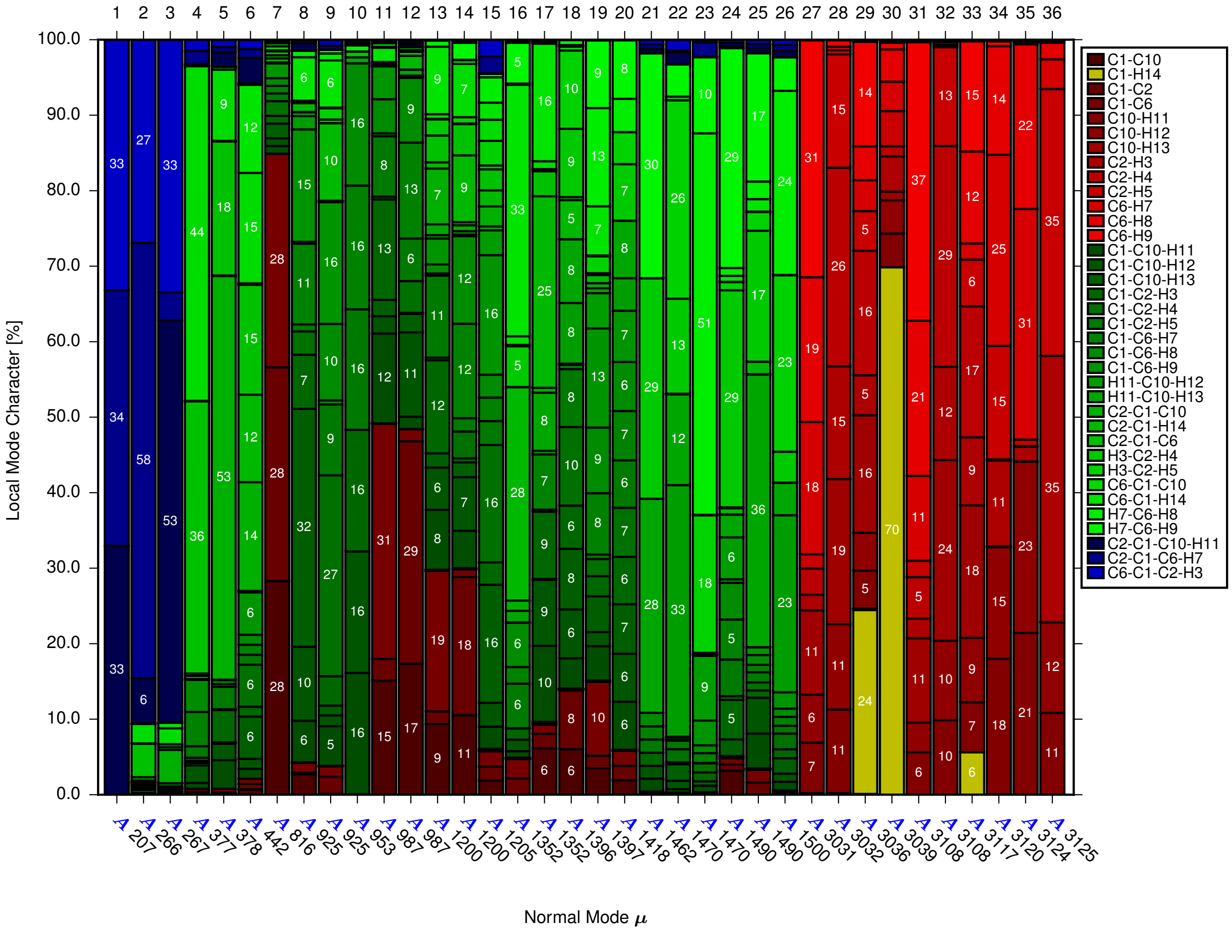

Figure 84: Decomposition of normal mode frequencies for 4-6

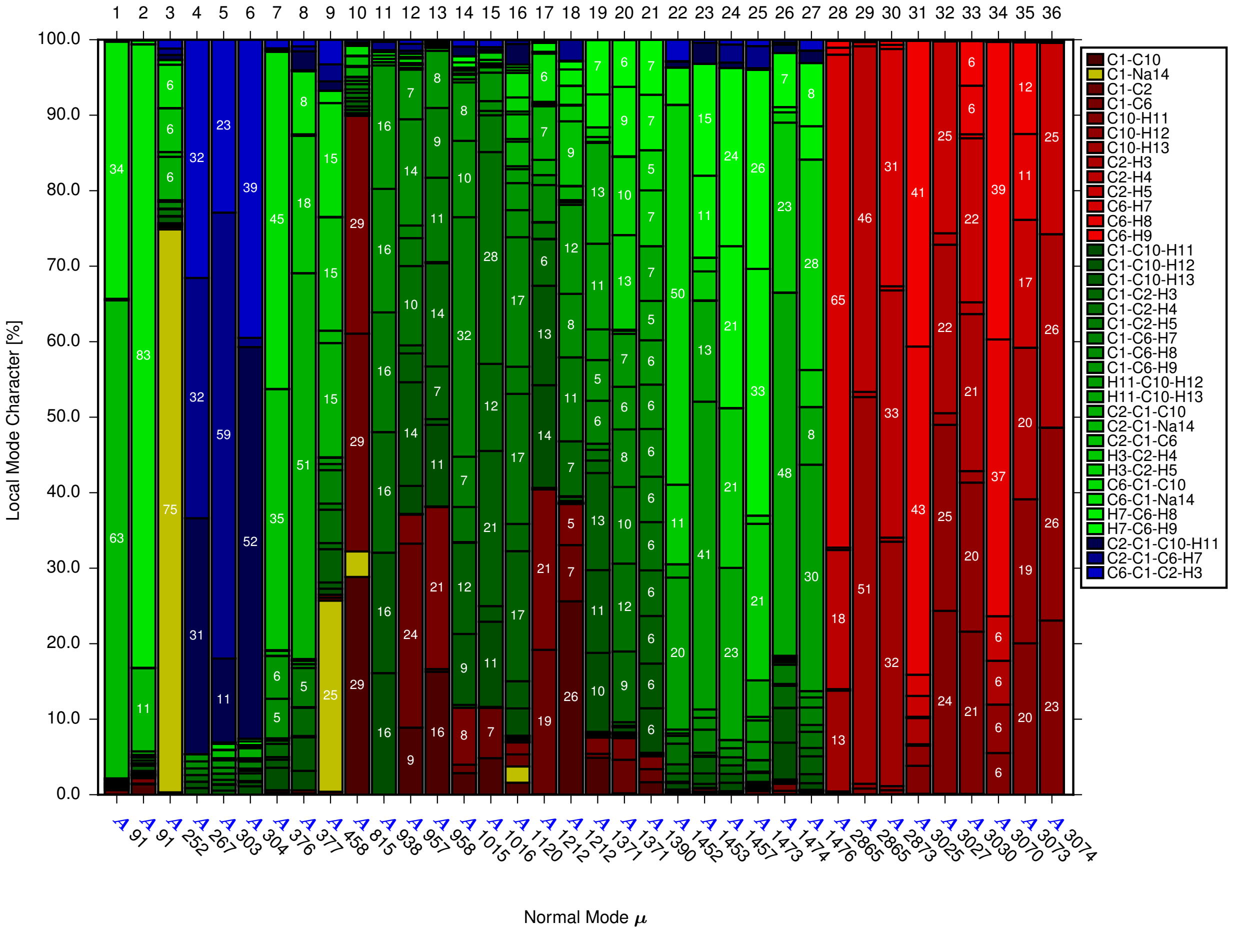

Figure 85: Decomposition of normal mode frequencies for 4-7

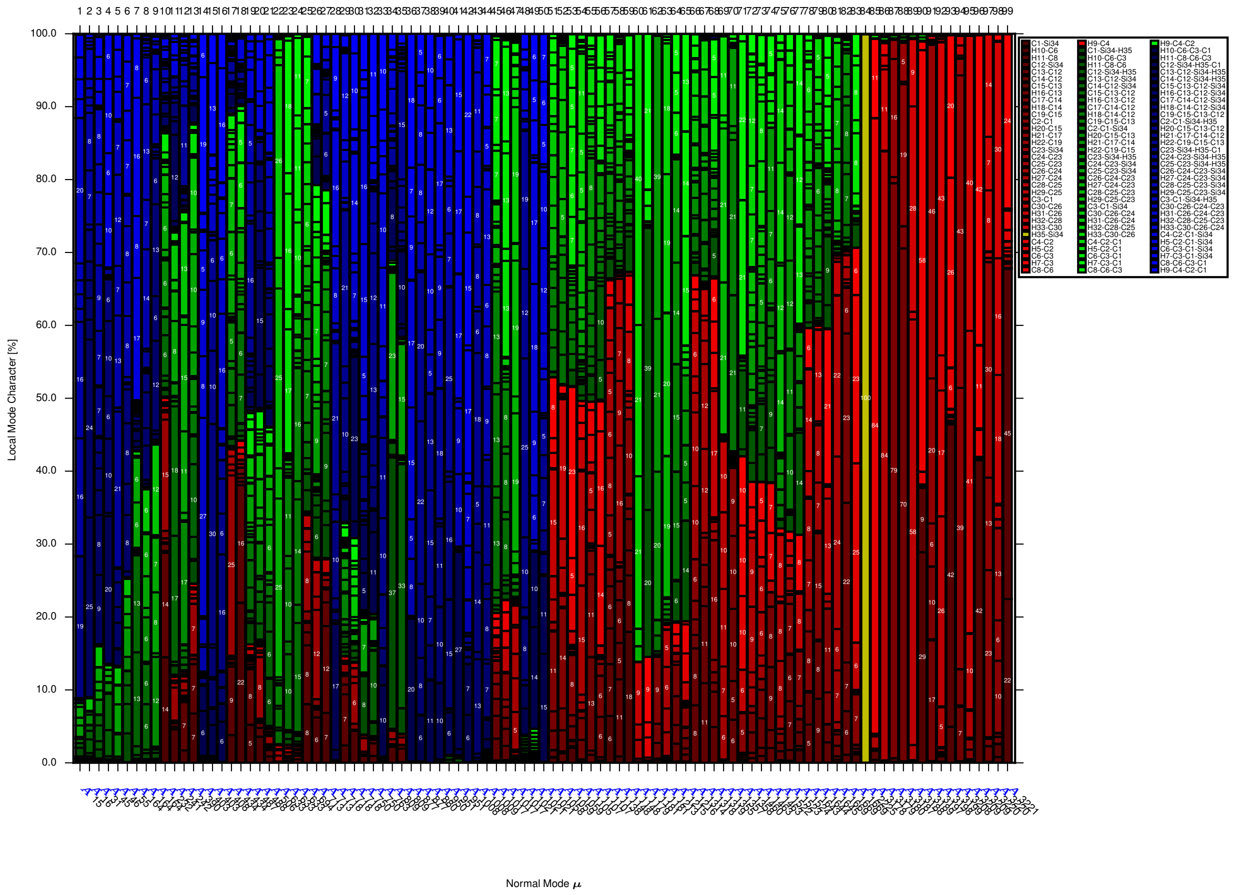

Figure 86: Decomposition of target normal mode frequency (Si-H bond) for 4-8

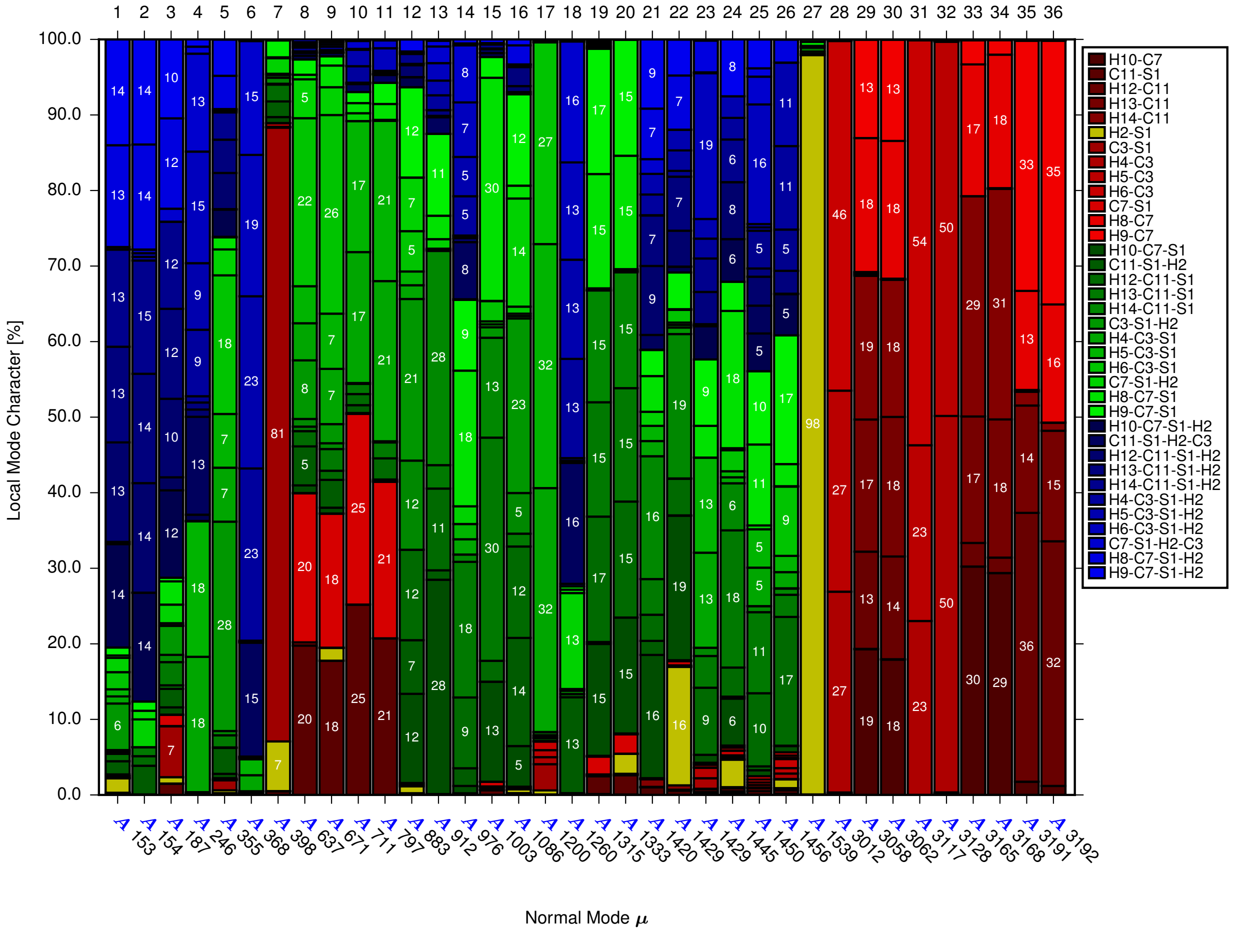

Figure 87: Decomposition of normal mode frequencies for 4-9

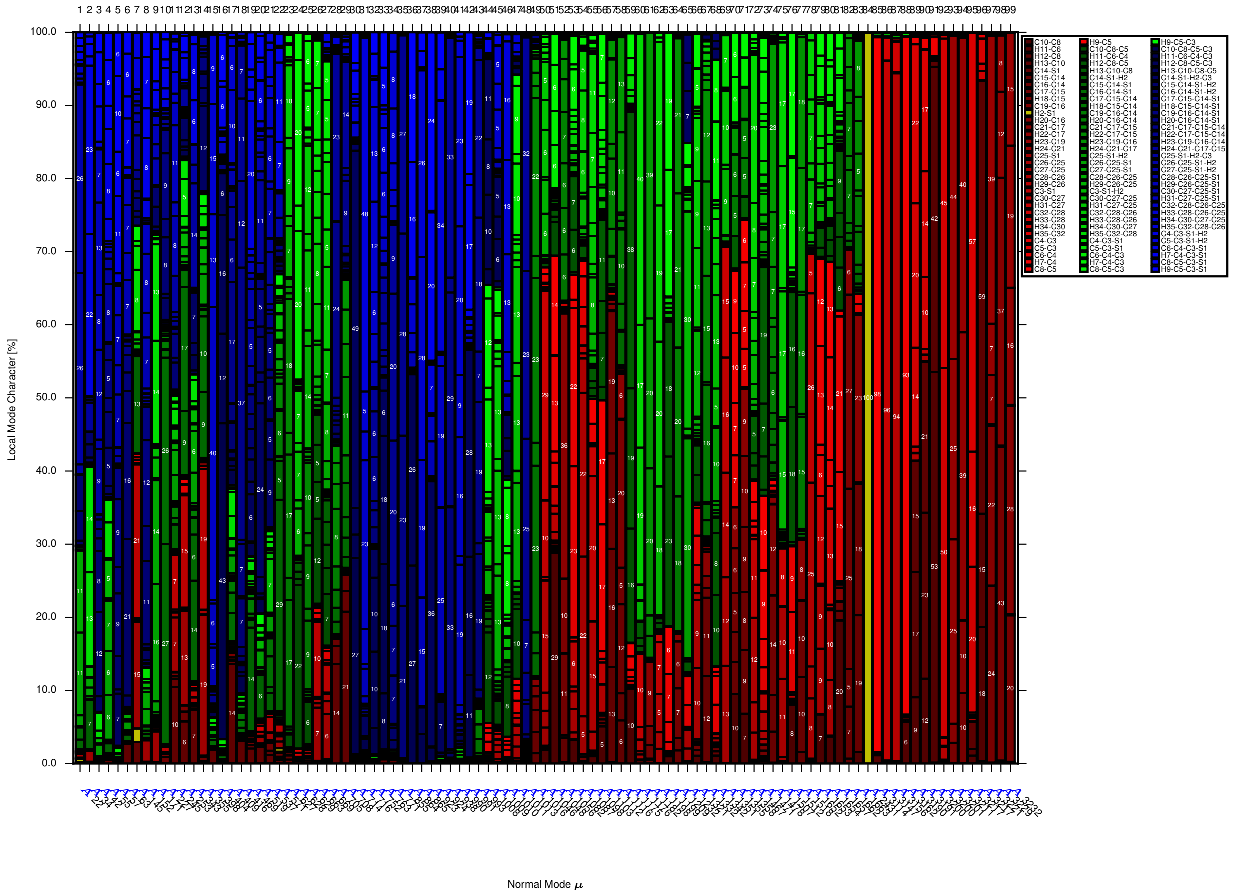

Figure 88: Decomposition of target normal mode frequency (S-H bond) for 4-10

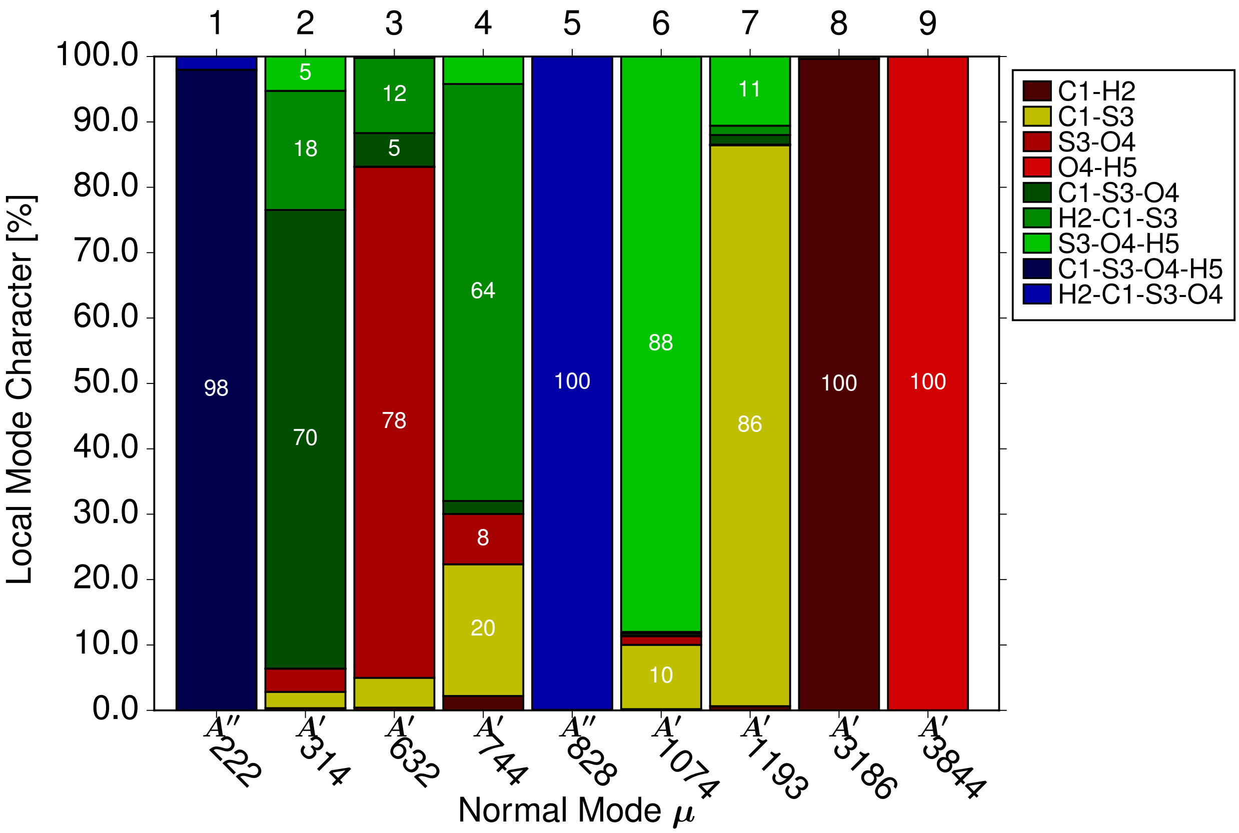

Figure 89: Decomposition of normal mode frequencies for 4-11

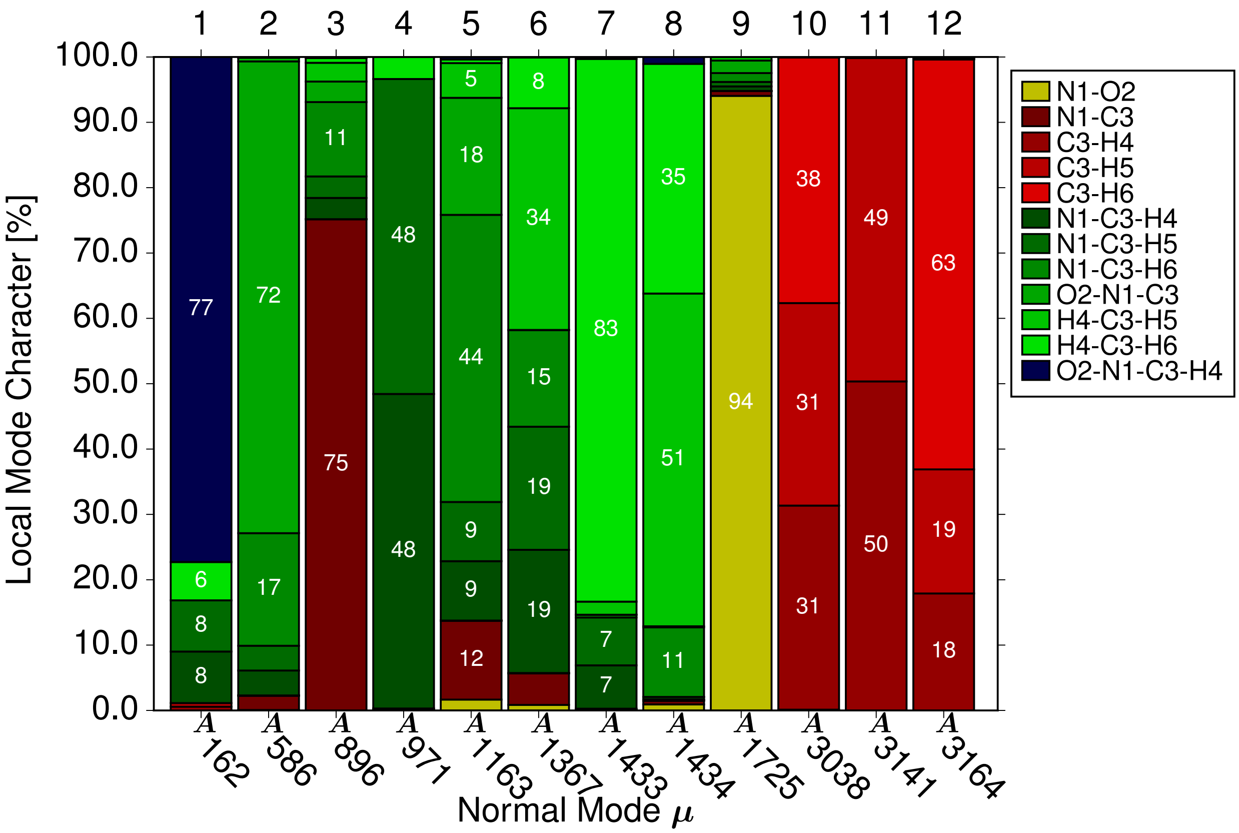

Figure 90: Decomposition of normal mode frequencies for 4-12

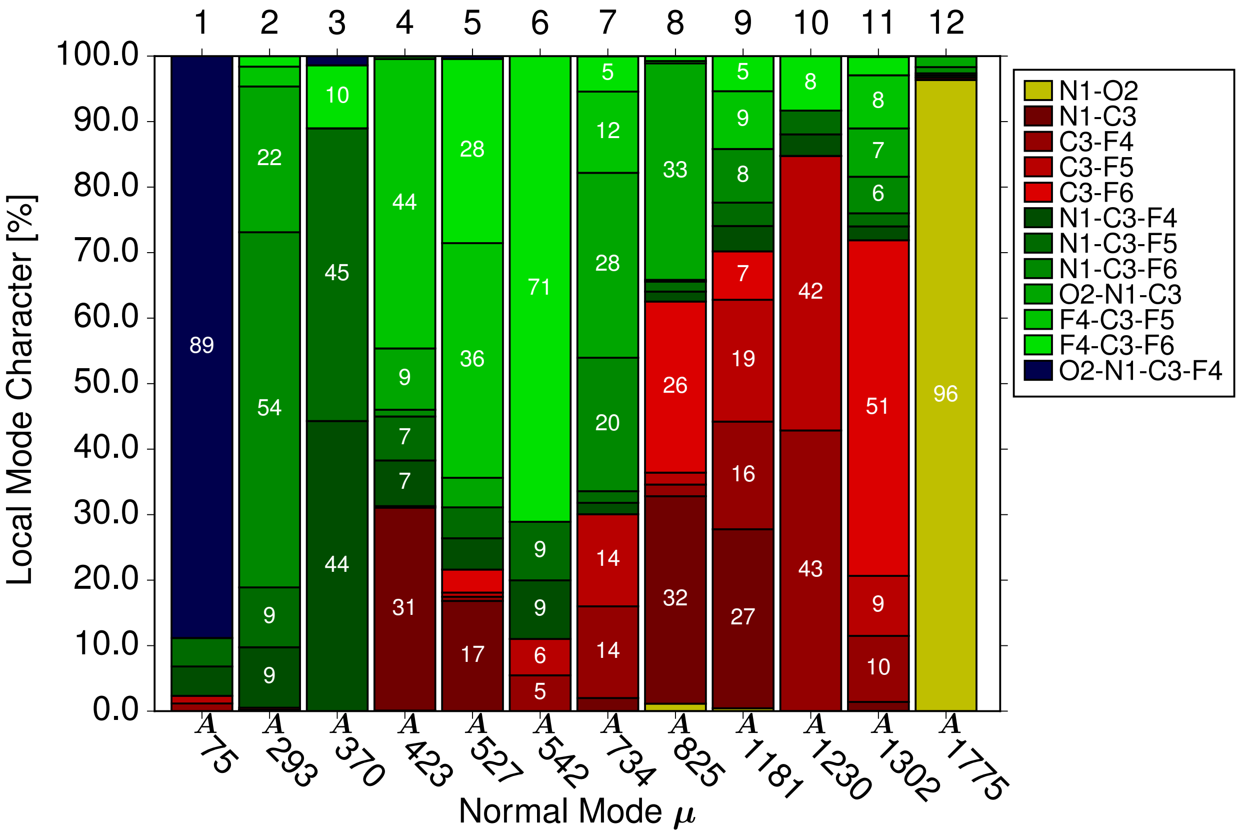

Figure 91: Decomposition of normal mode frequencies for 4-13

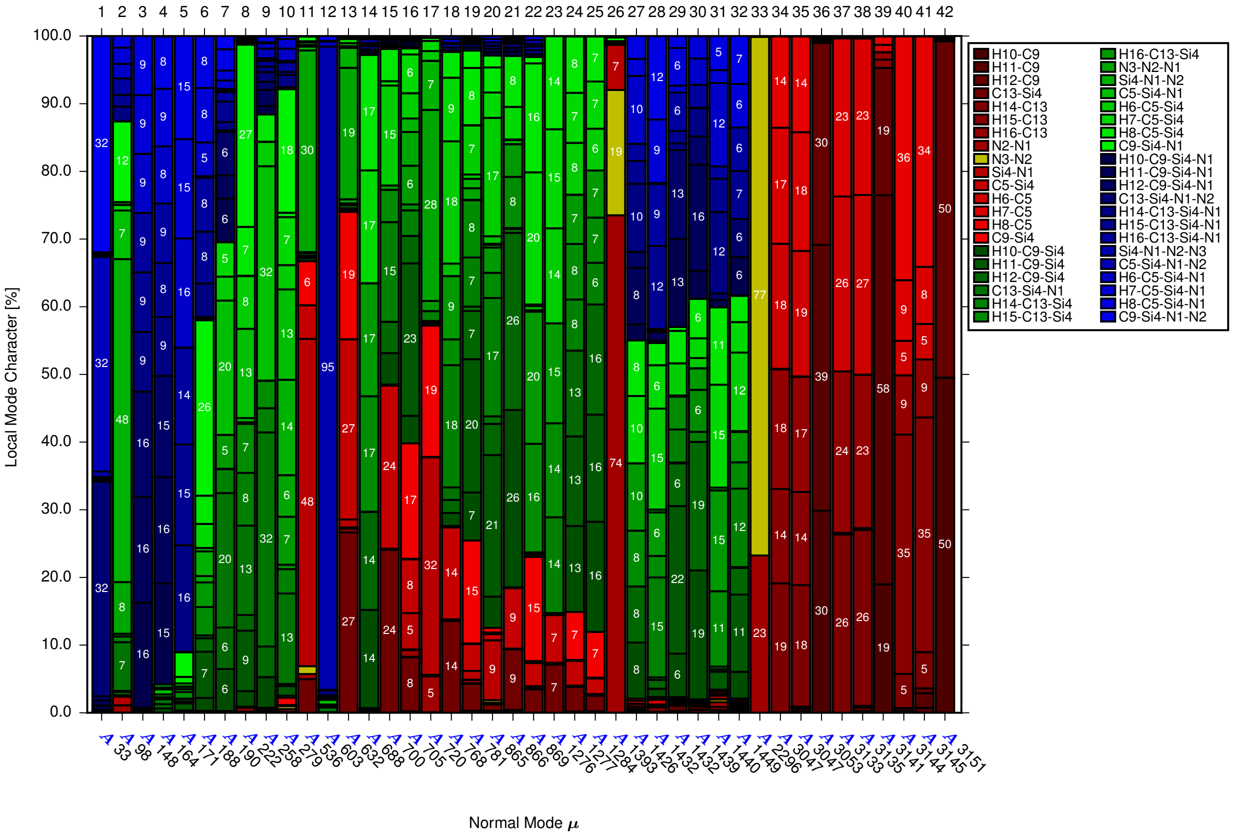

Figure 92: Decomposition of normal mode frequencies for 4-14

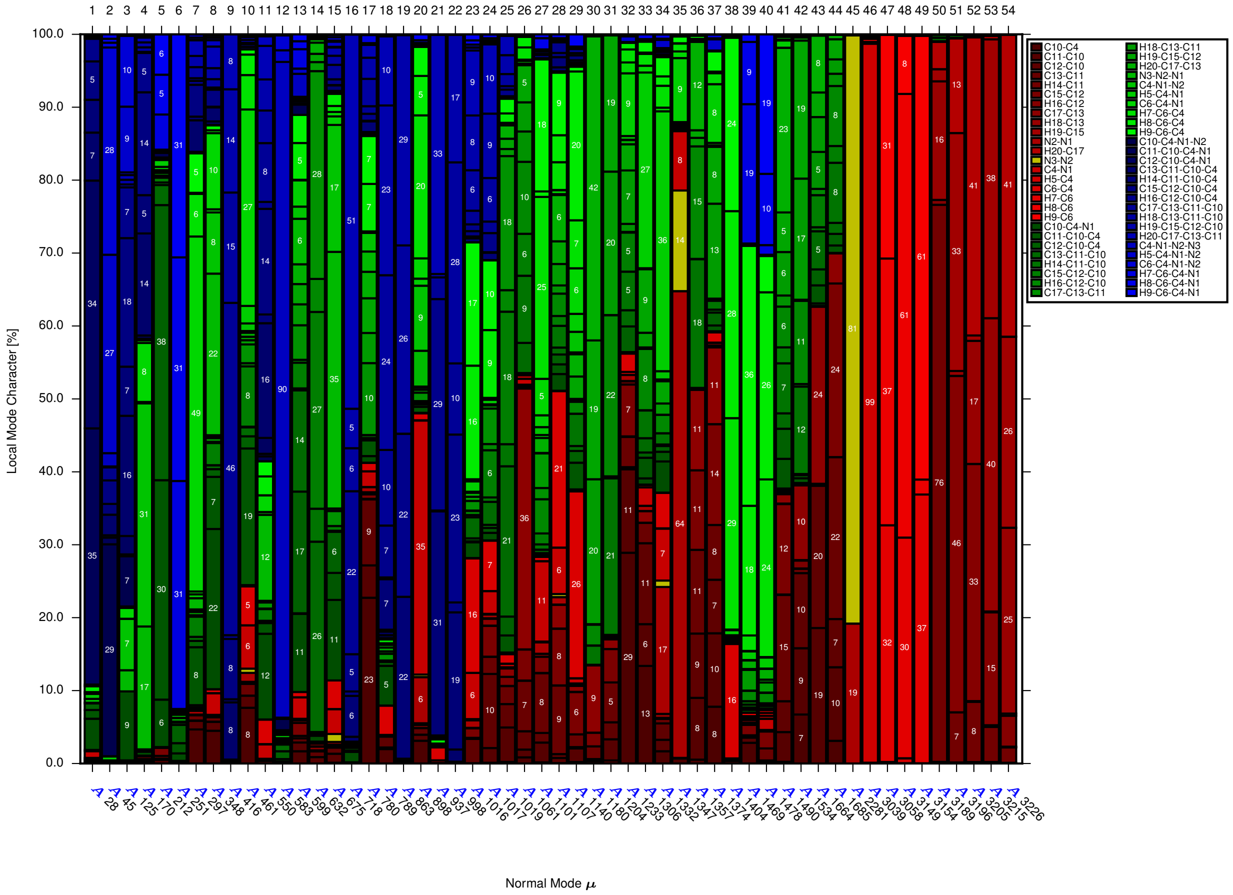

Figure 93: Decomposition of normal mode frequencies for 4-15

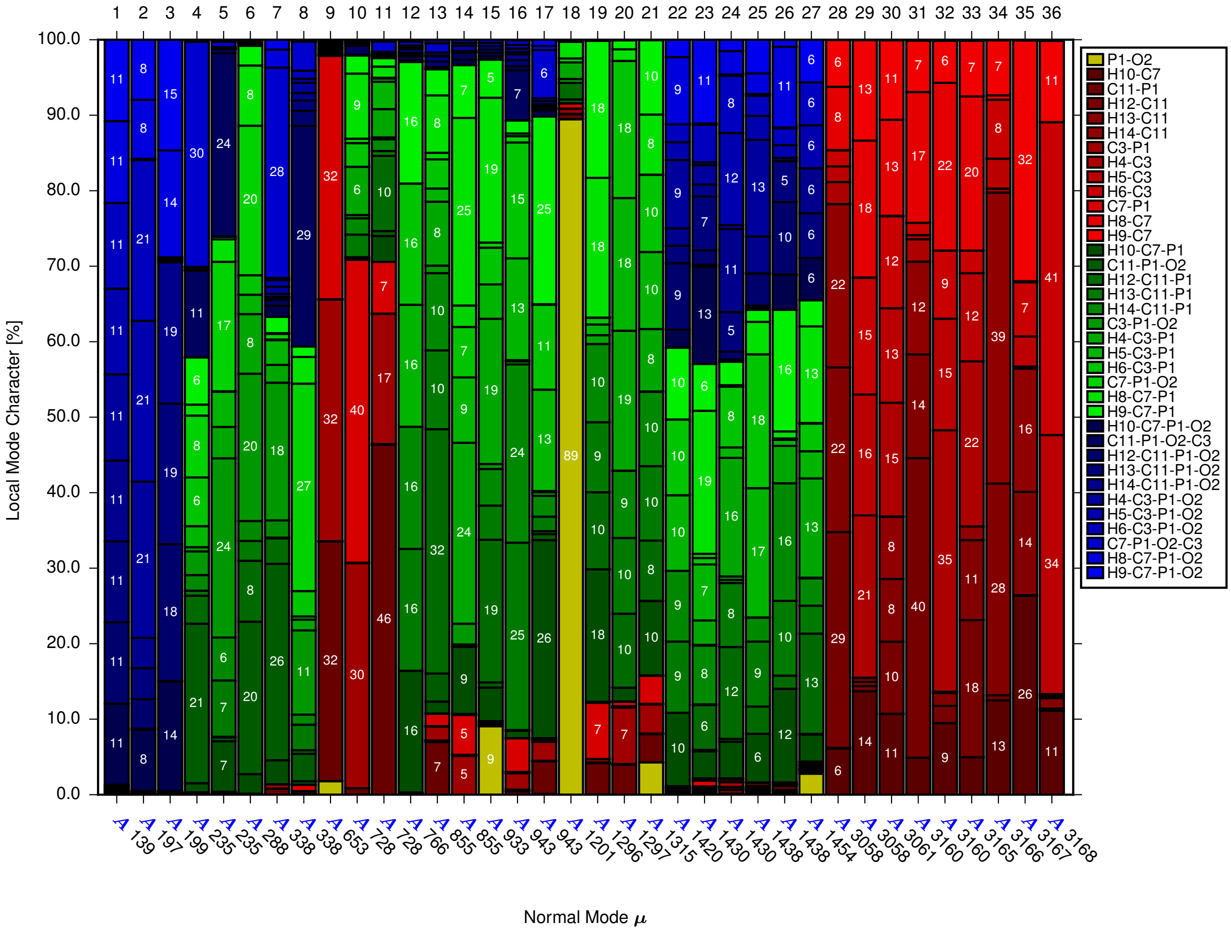

Figure 94: Decomposition of normal mode frequencies for 4-16

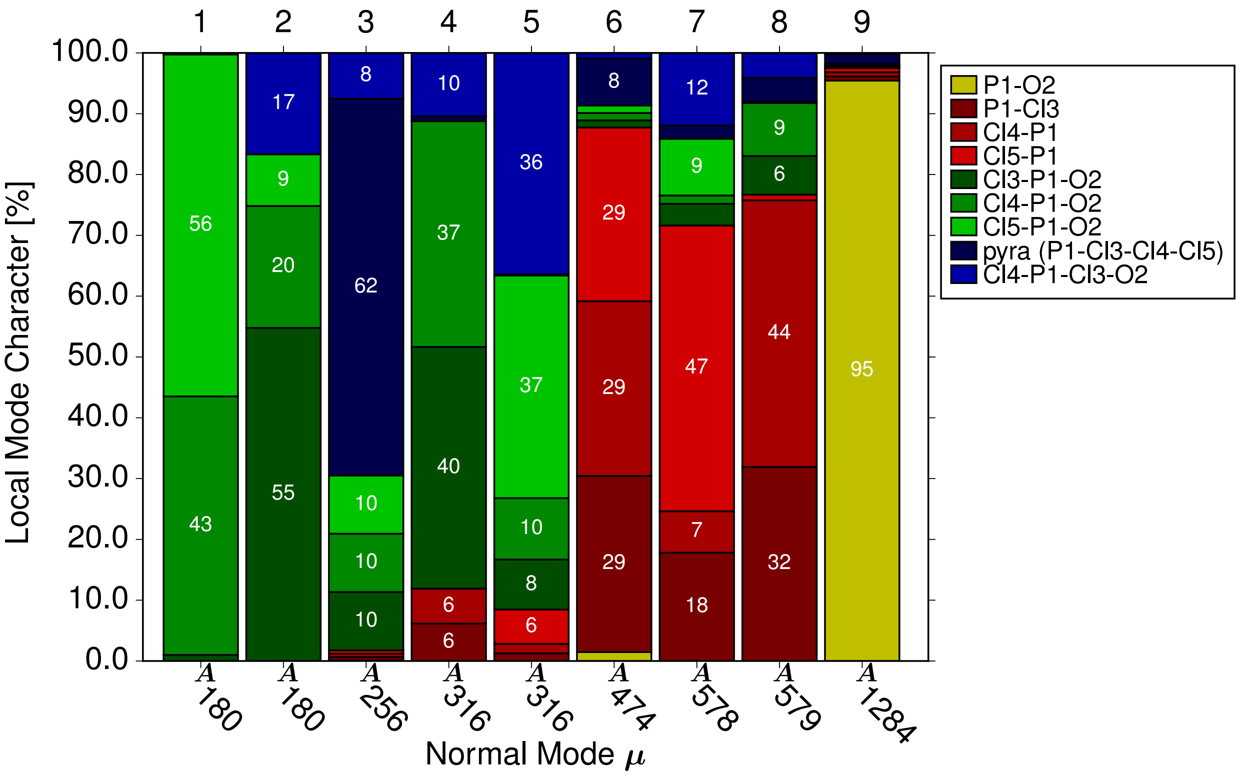

Figure 95: Decomposition of normal mode frequencies for 4-17

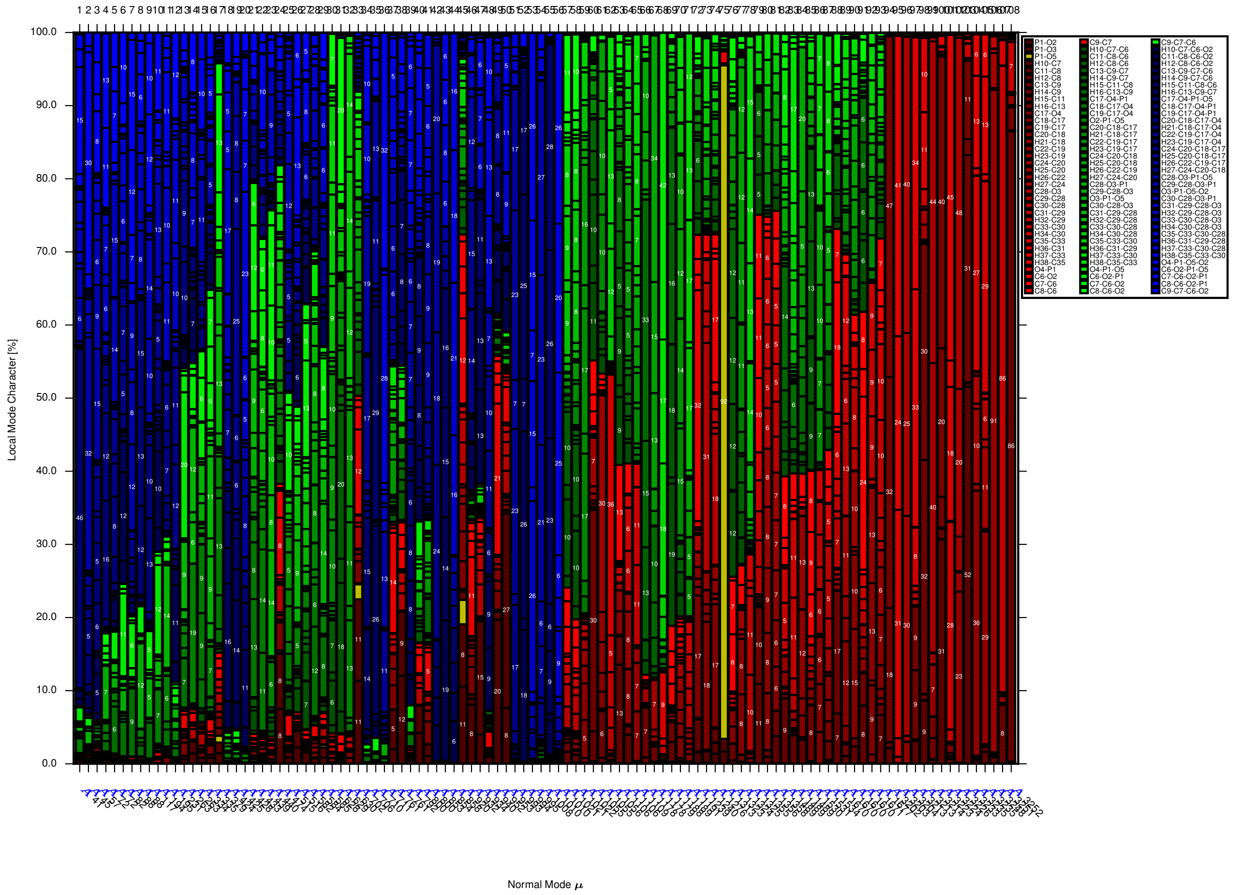

Figure 96: Decomposition of target normal mode frequency (P=O bond) for 4-18

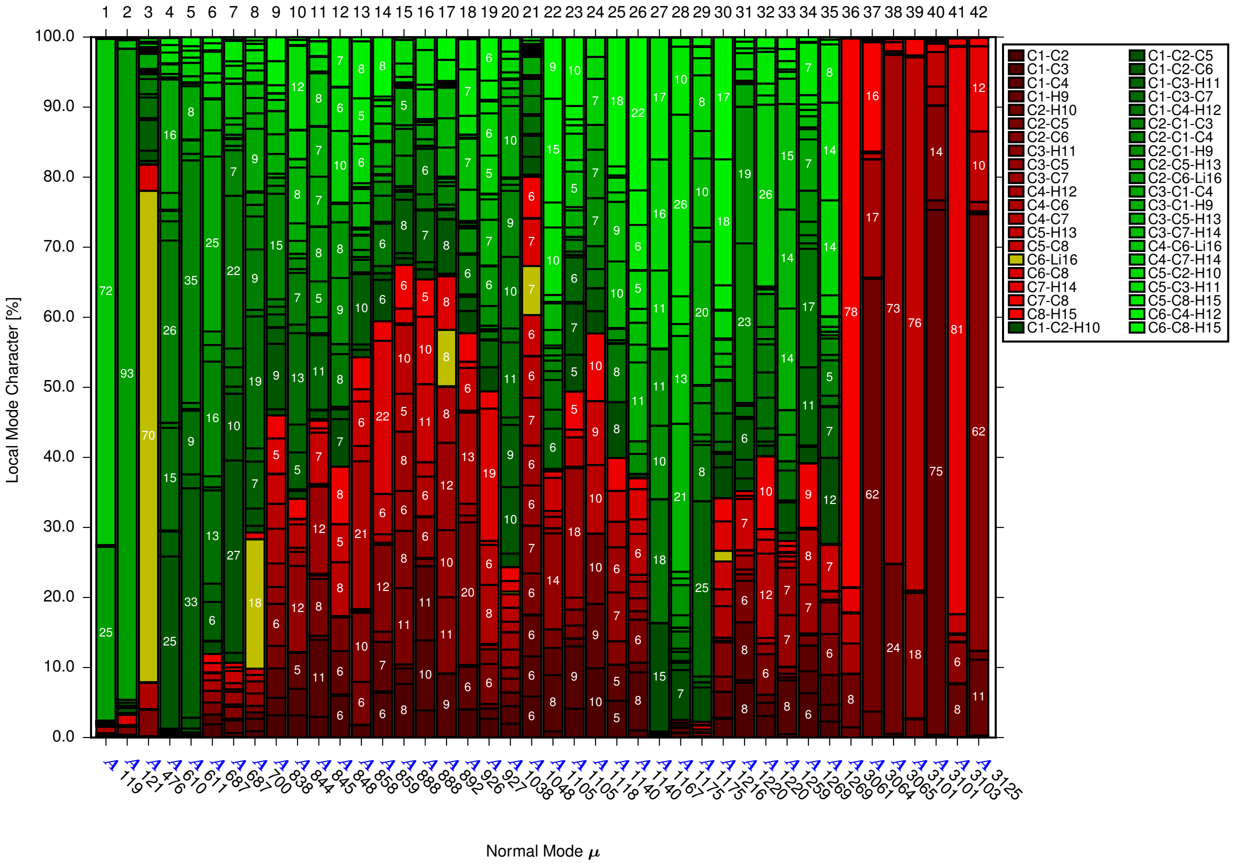

Figure 97: Decomposition of normal mode frequencies for 4-19

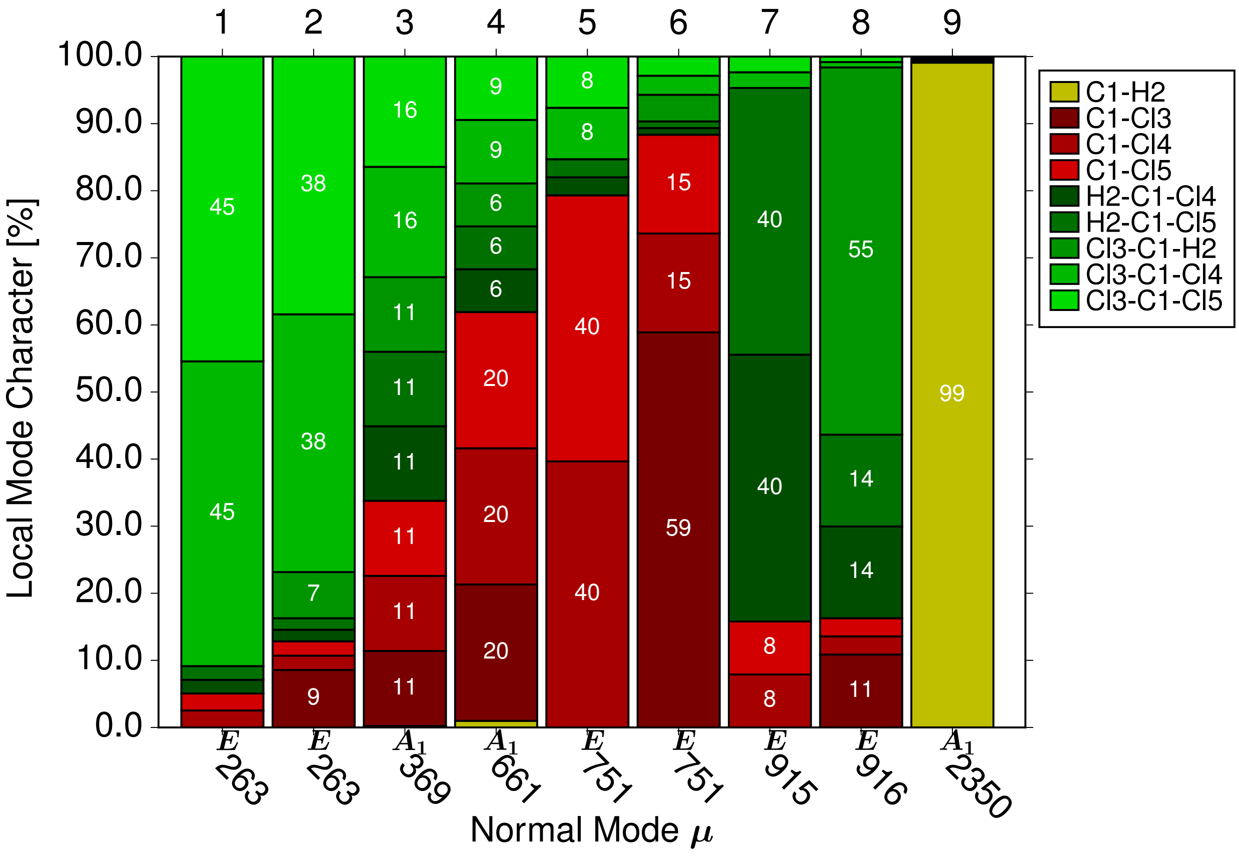

Figure 98: Decomposition of normal mode frequencies for 4-20

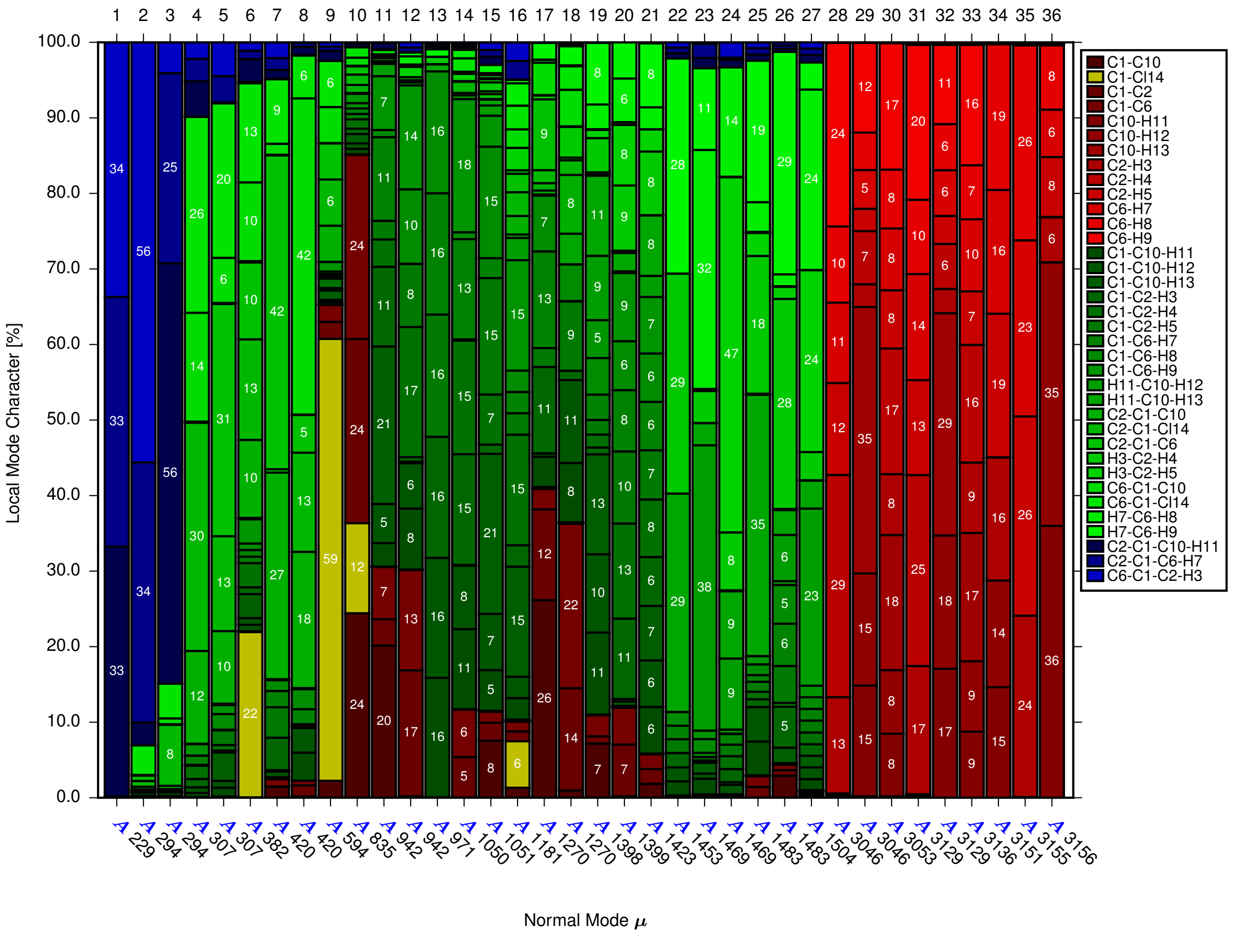

Figure 99: Decomposition of normal mode frequencies for 4-21

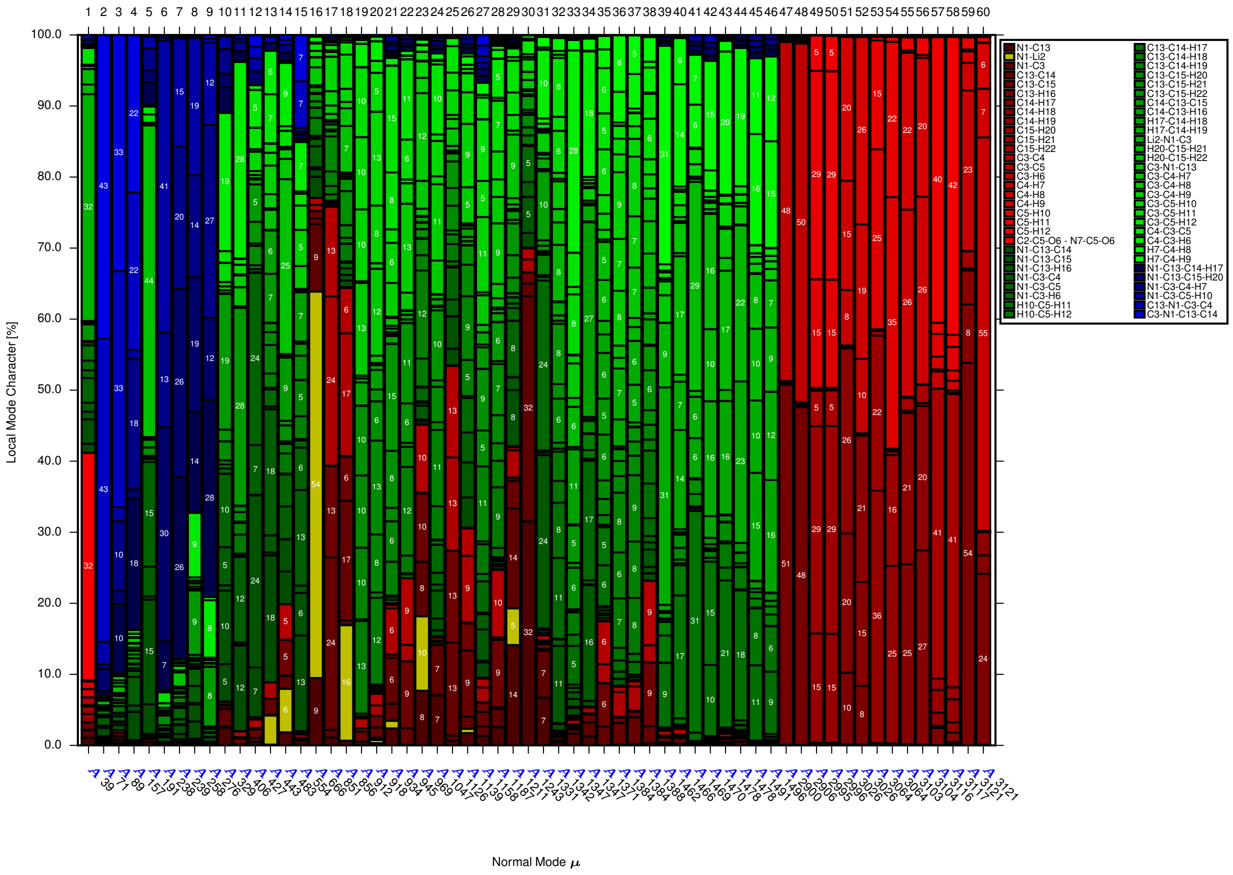

Figure 100: Decomposition of normal mode frequencies for 4-22

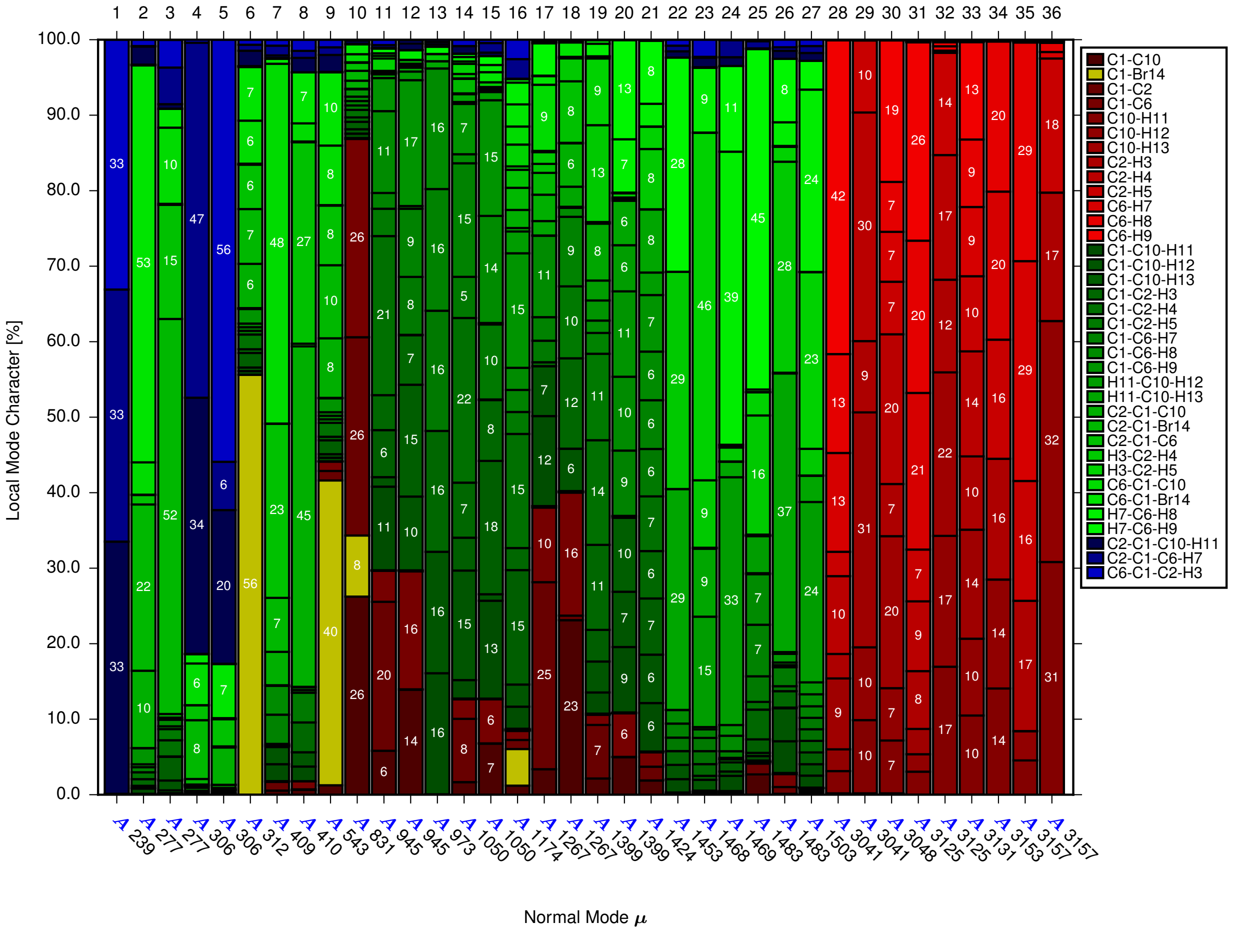

Figure 101: Decomposition of normal mode frequencies for 4-23

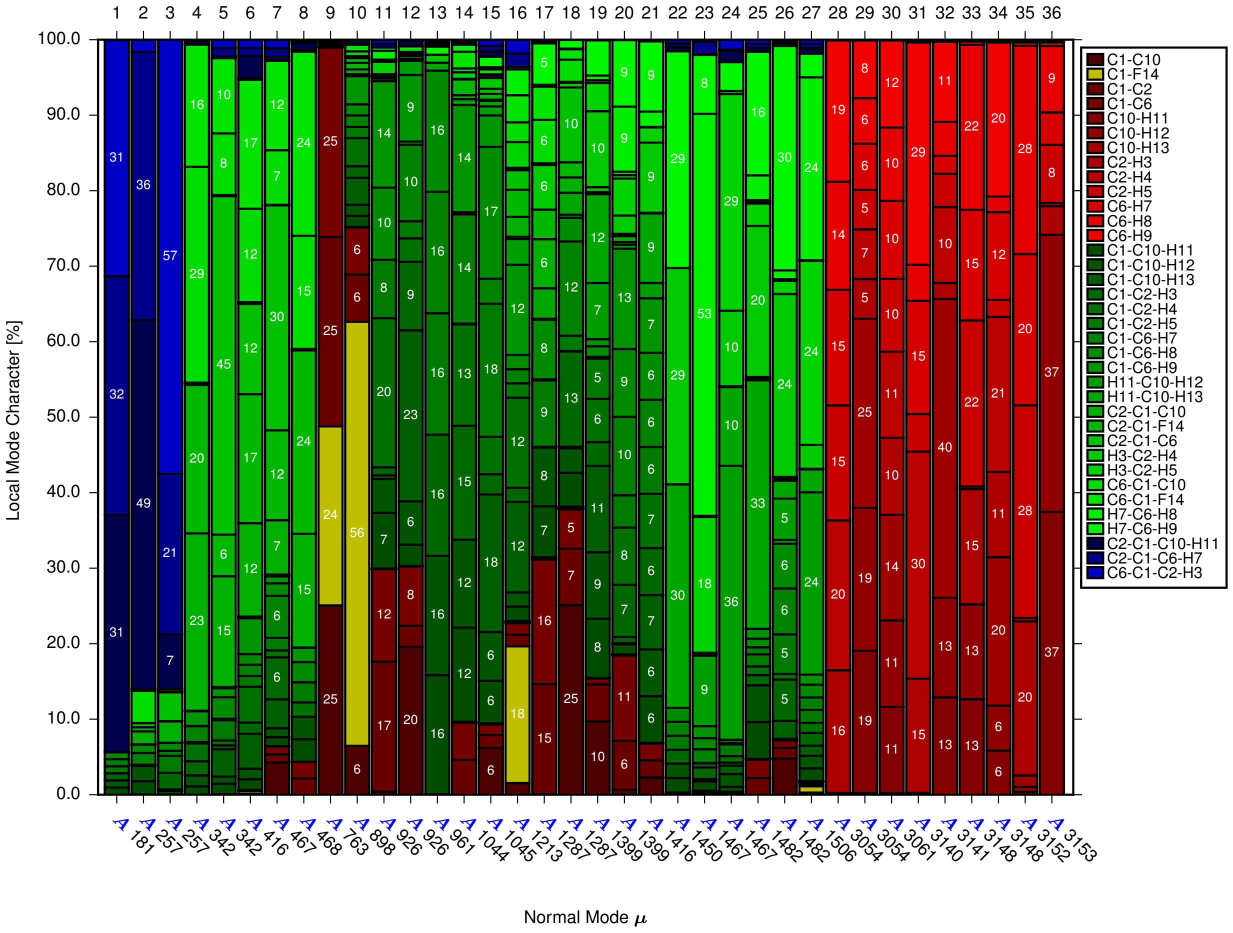

Figure 102: Decomposition of normal mode frequencies for 4-24

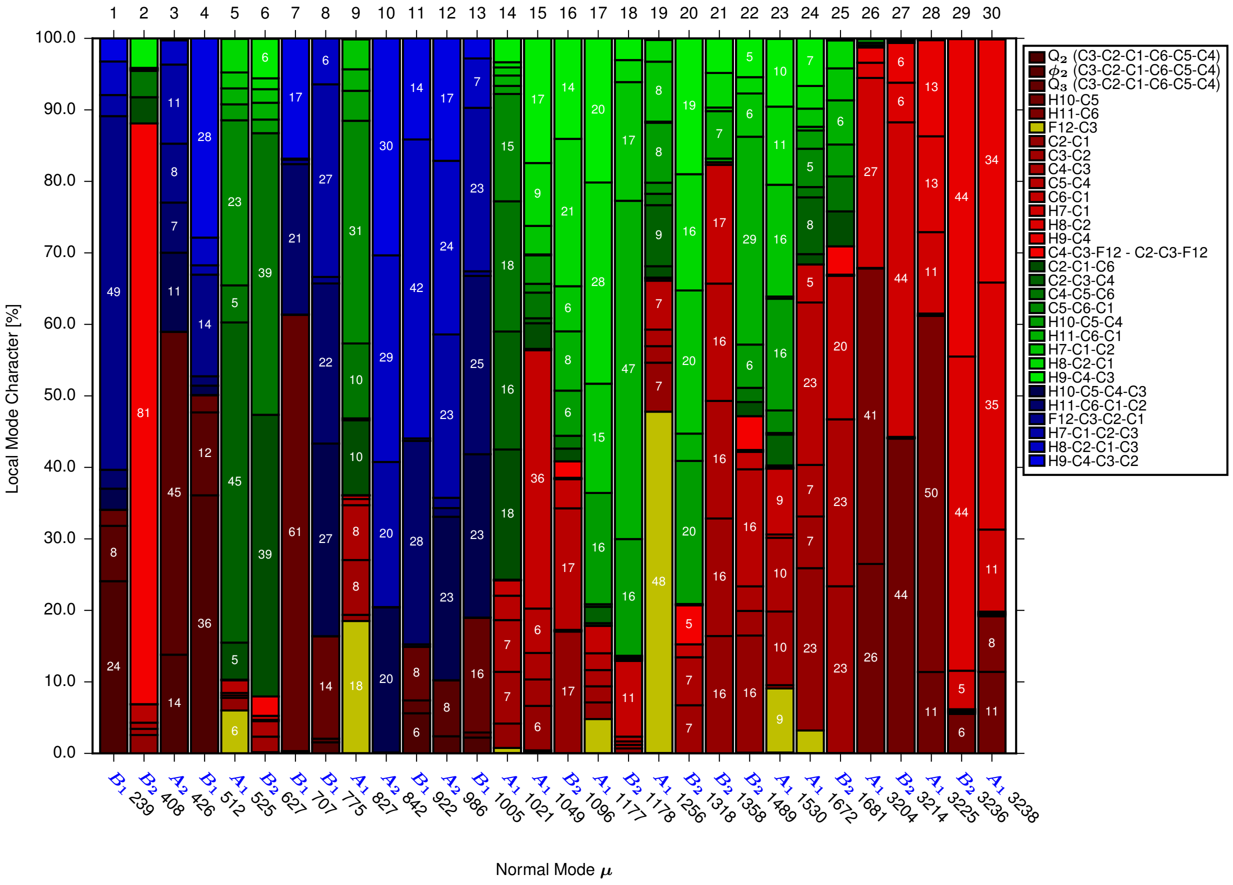

Figure 103: Decomposition of normal mode frequencies for 4-25

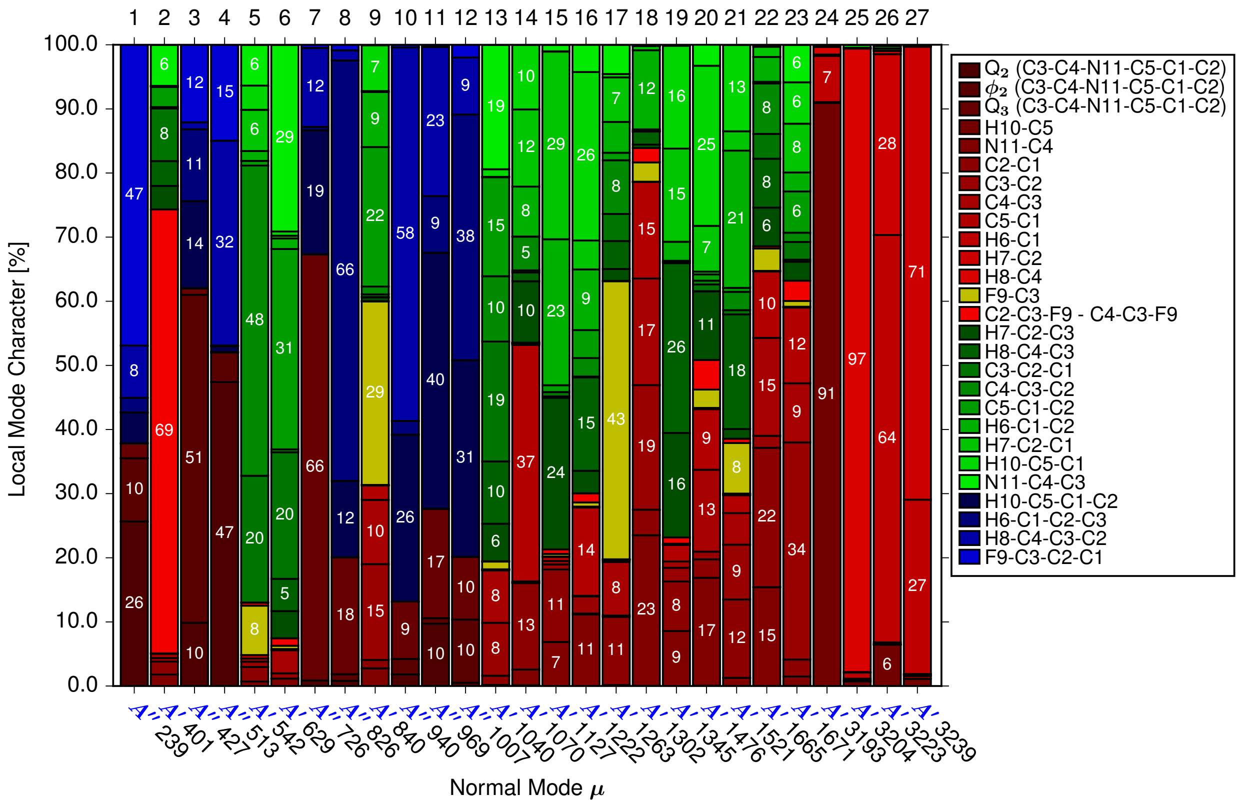

Figure 104: Decomposition of normal mode frequencies for 4-26

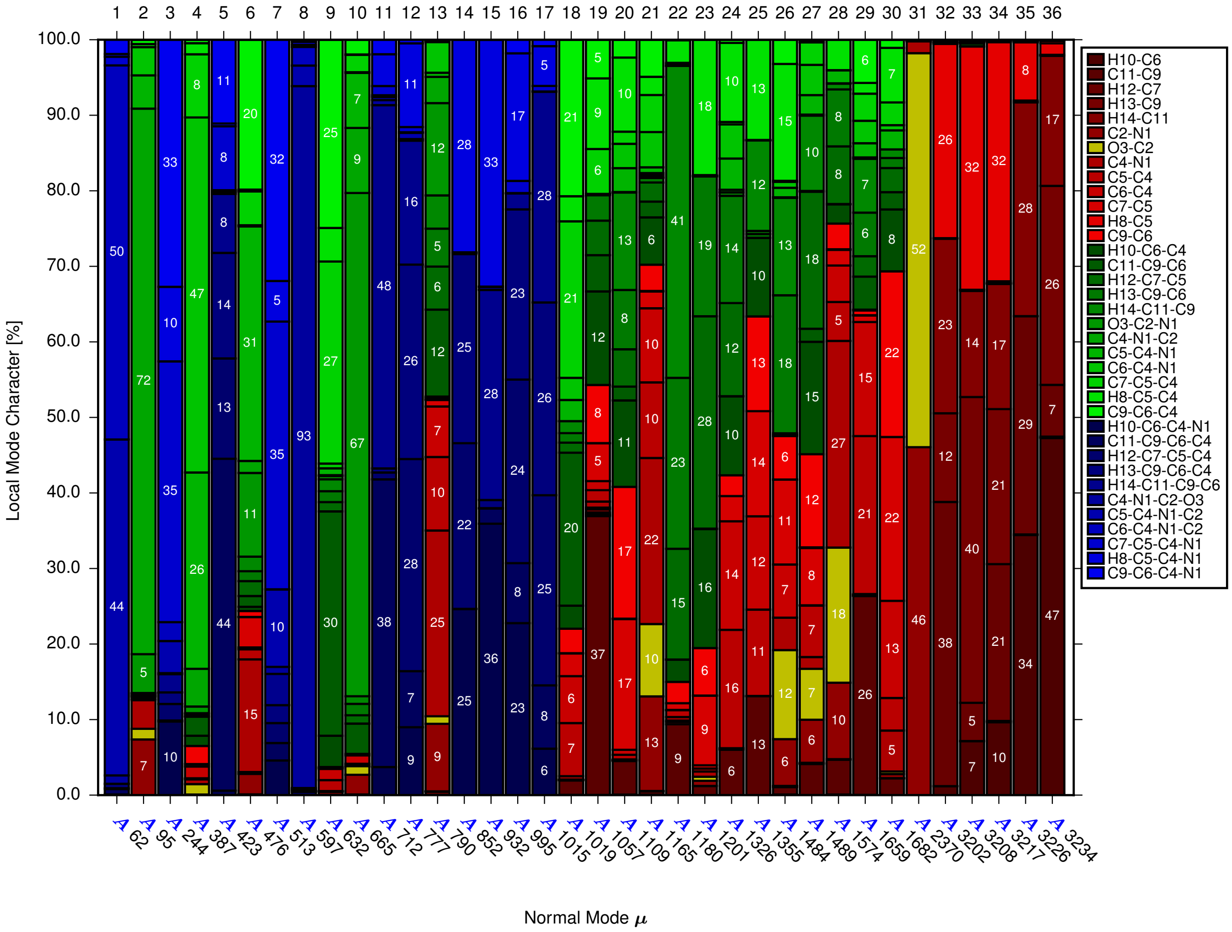

Figure 105: Decomposition of normal mode frequencies for 4-27

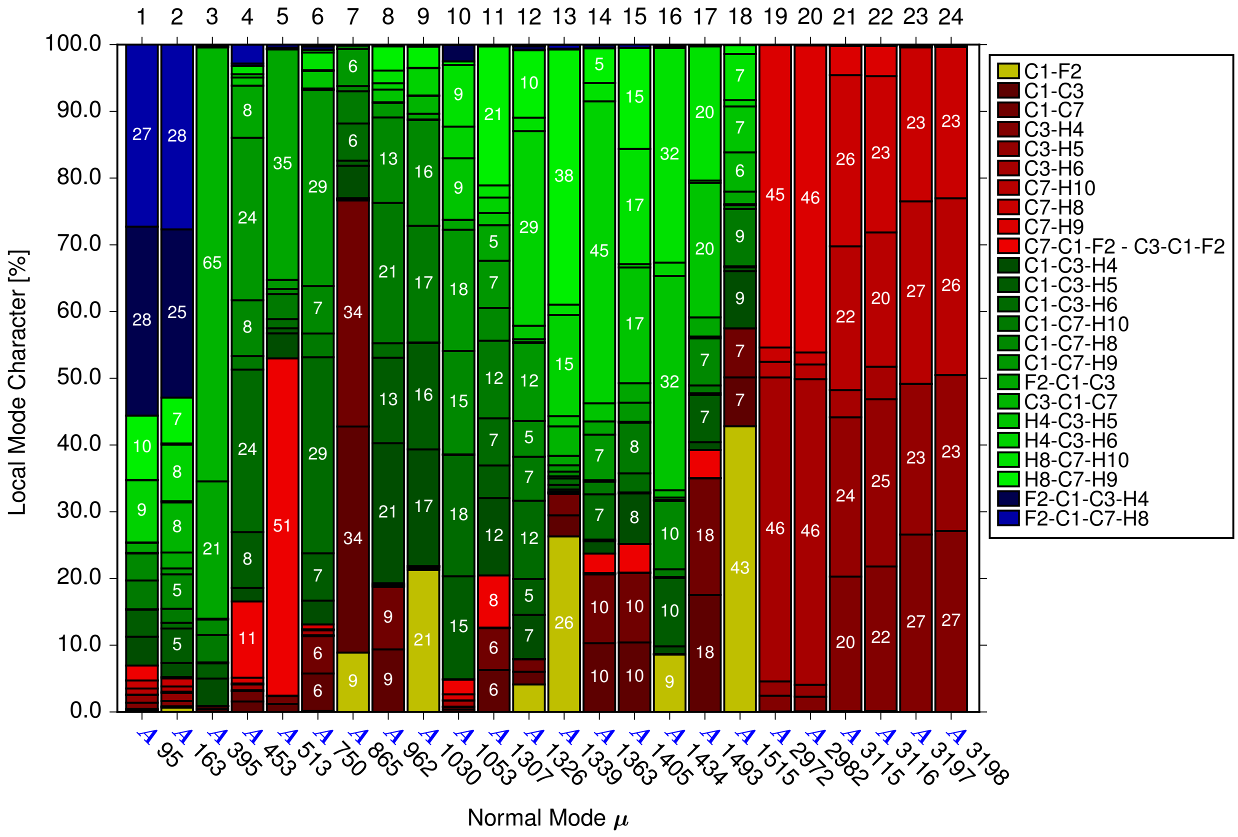

Figure 106: Decomposition of normal mode frequencies for 4-28

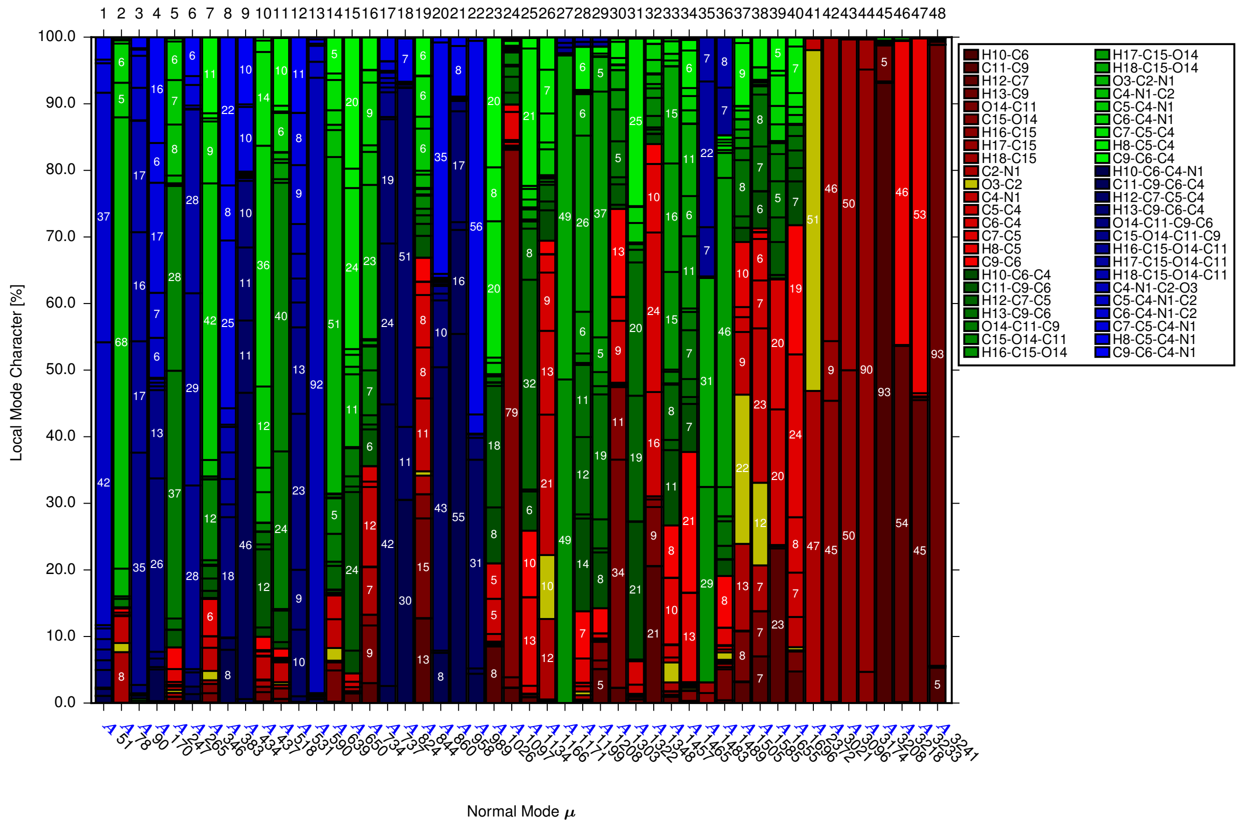

Figure 107: Decomposition of normal mode frequencies for 4-29

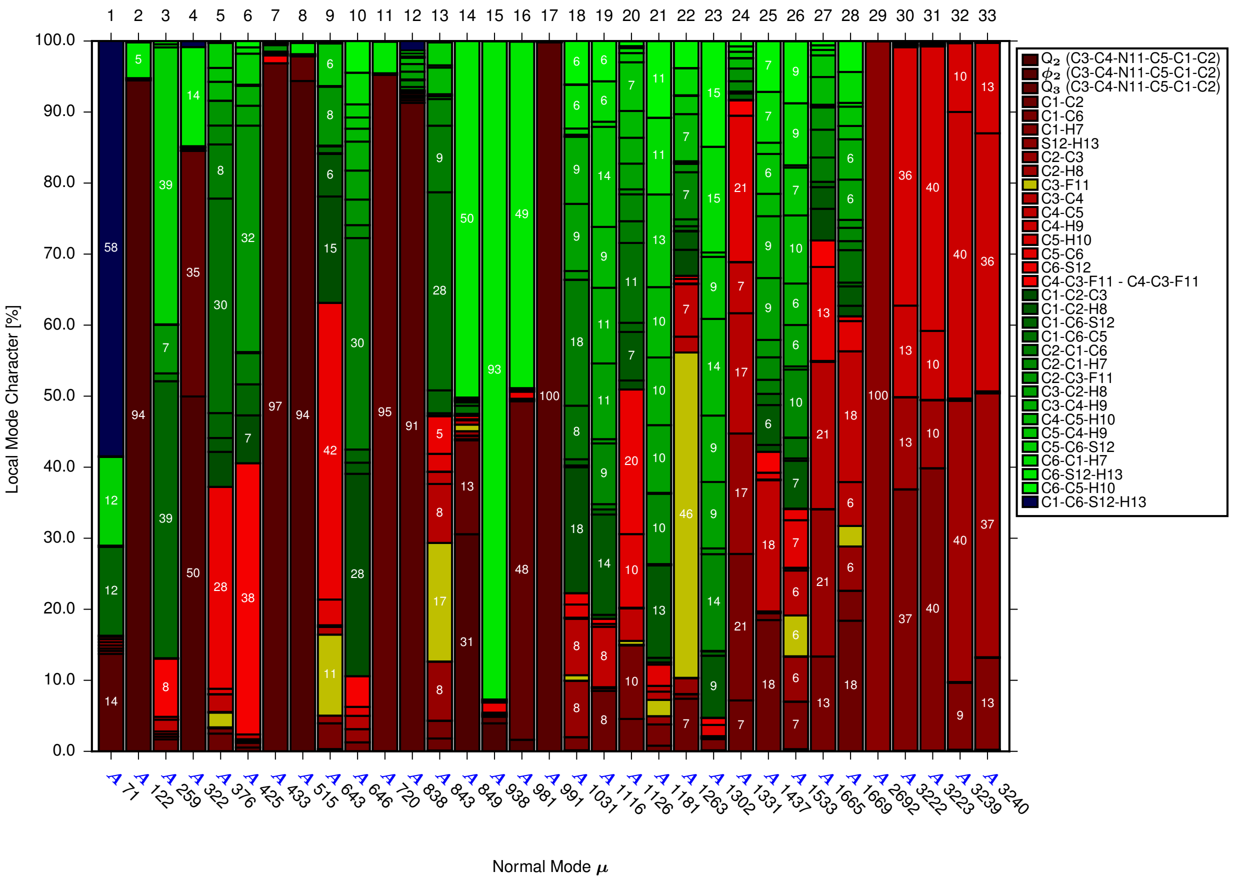

Figure 108: Decomposition of normal mode frequencies for 4-30

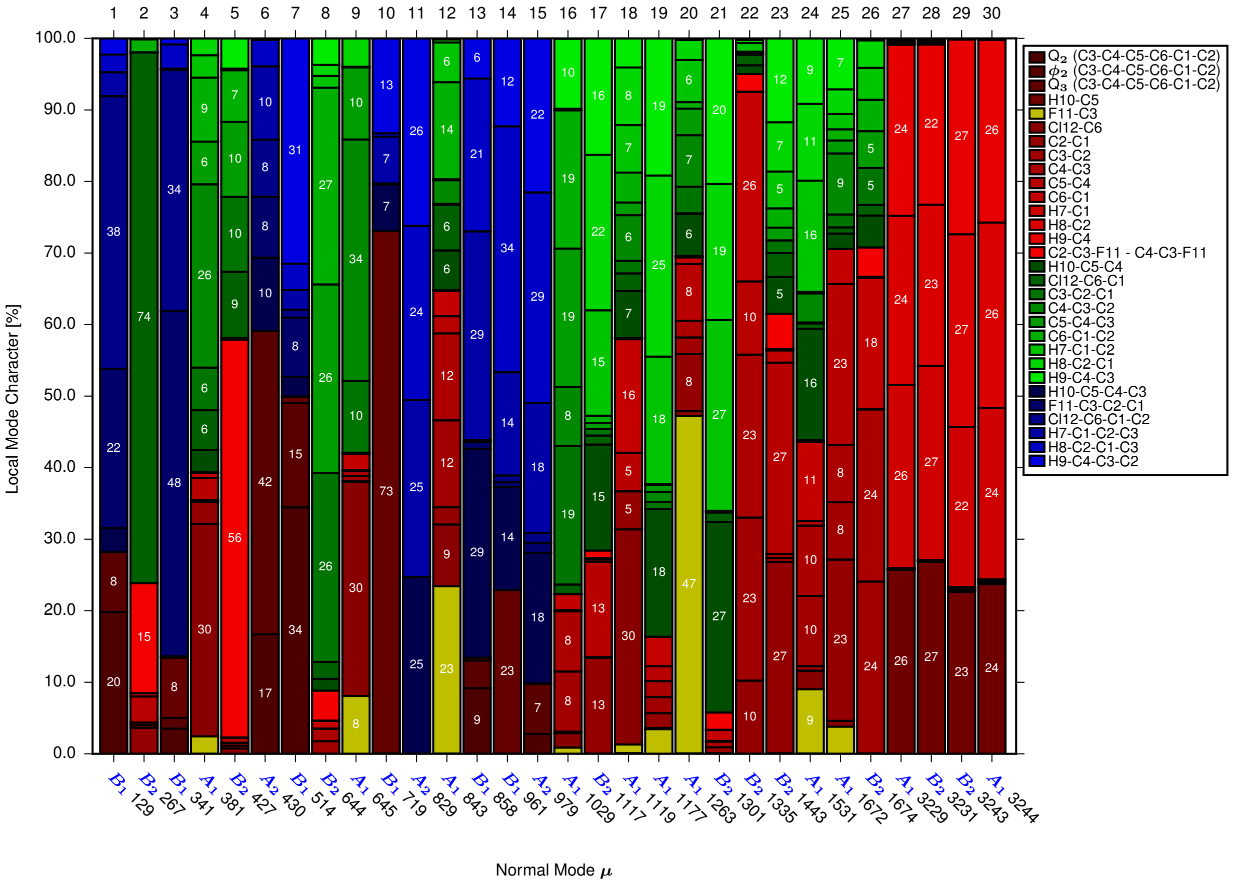

Figure 109: Decomposition of normal mode frequencies for 4-31

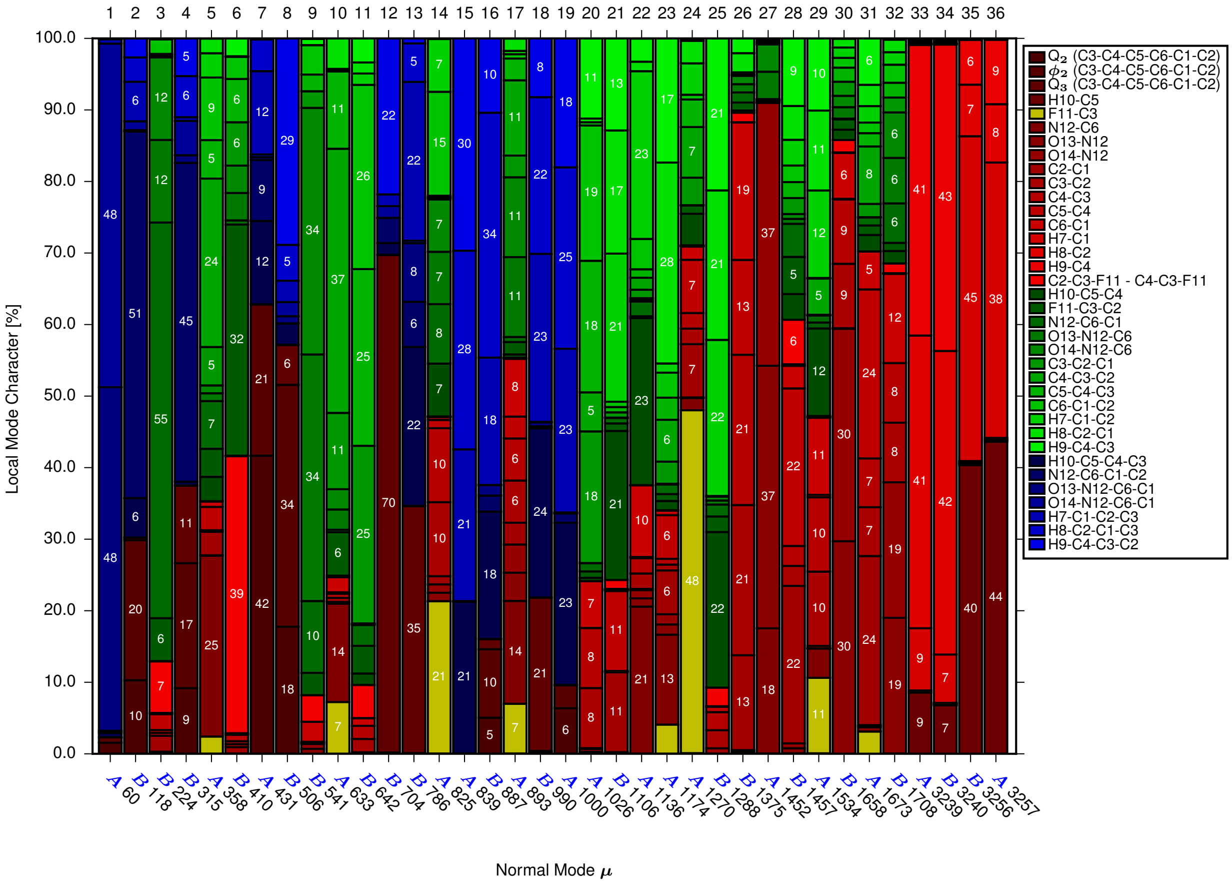

Figure 110: Decomposition of normal mode frequencies for 4-32

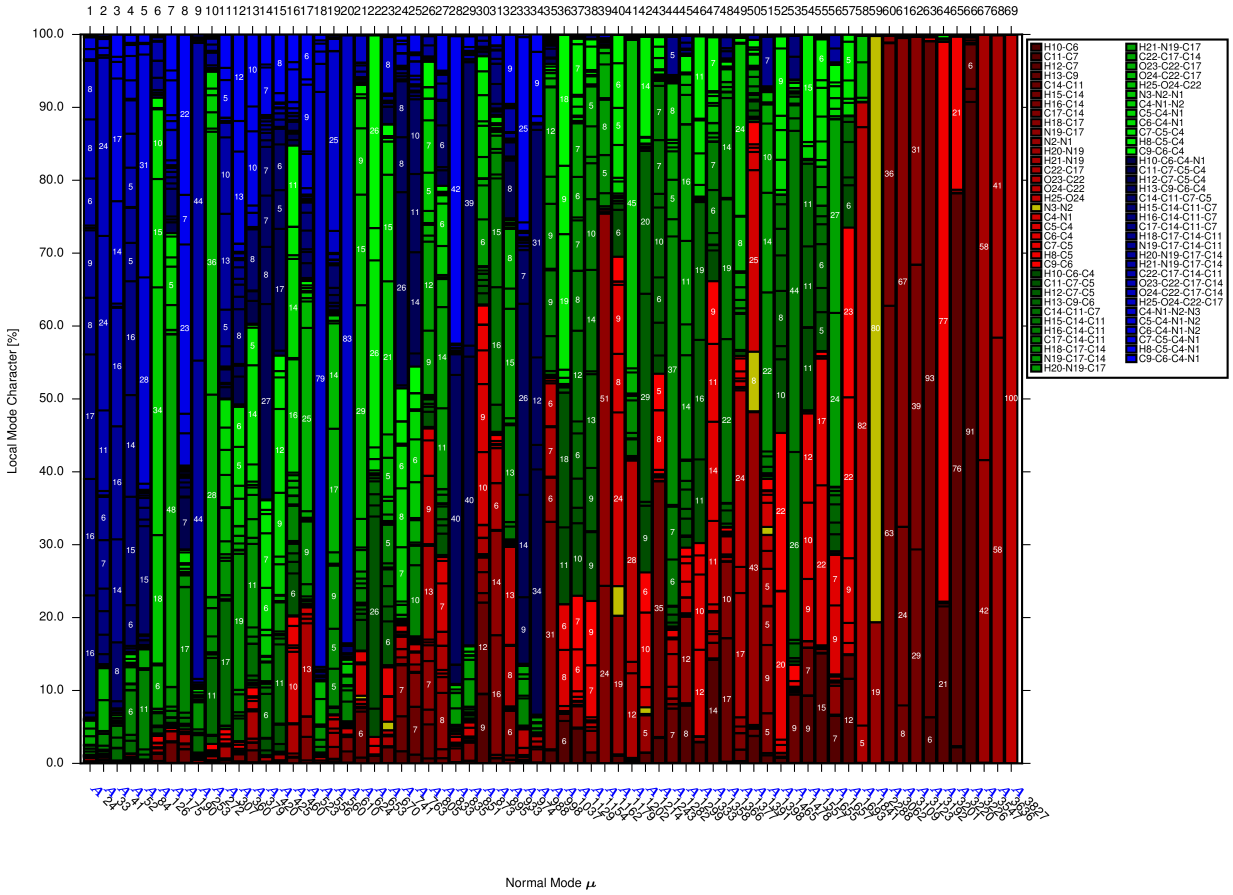

Figure 111: Decomposition of normal mode frequencies for 4-33

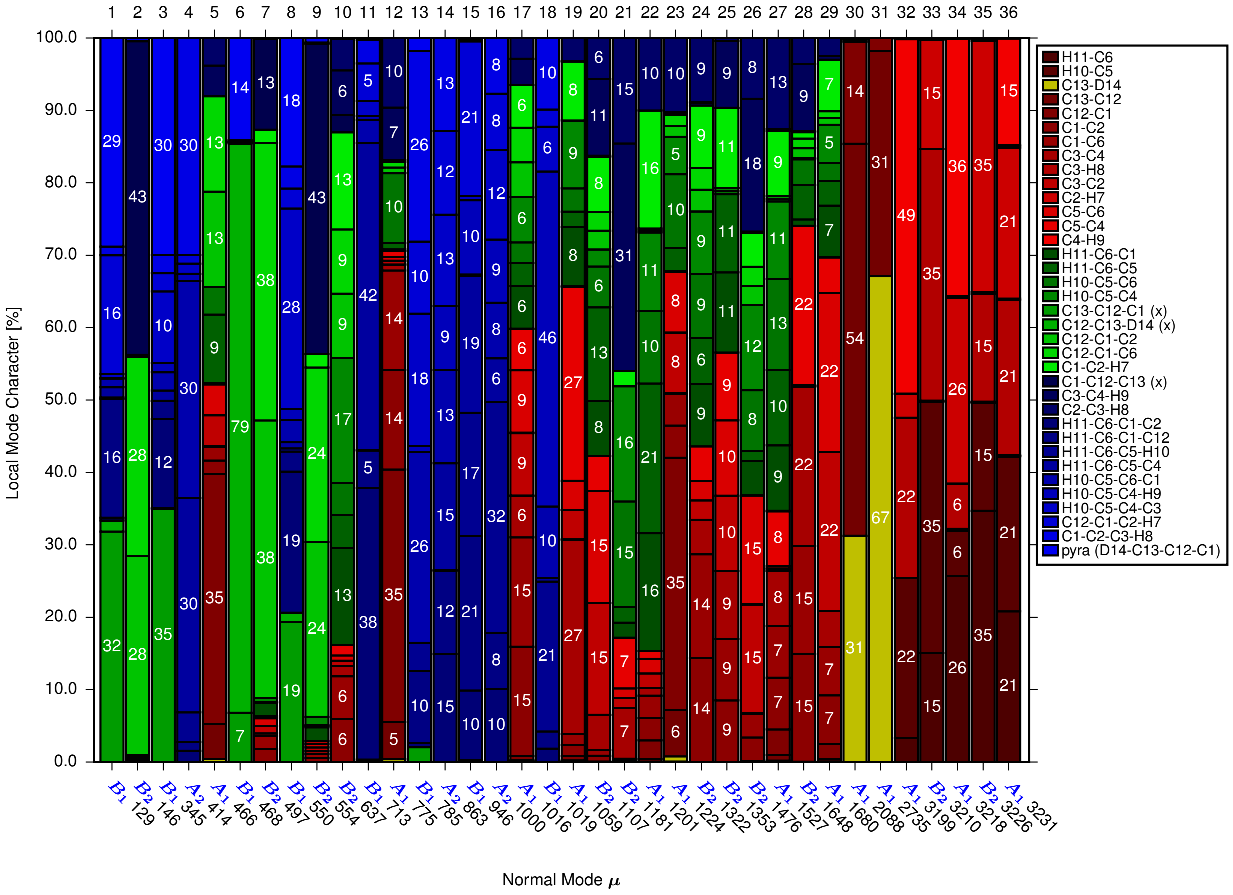

Figure 112: Decomposition of target normal mode frequency (C-D bond) for 4-34

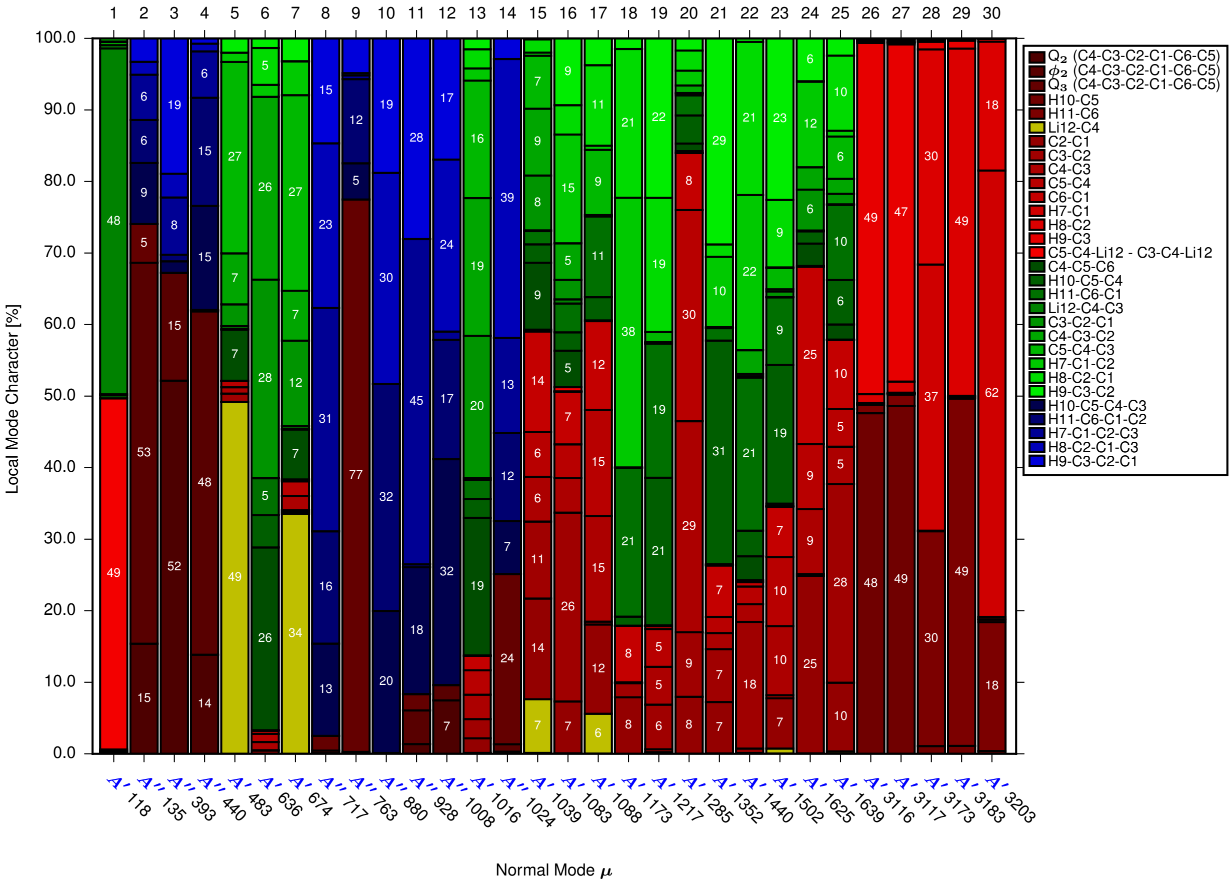

Figure 113: Decomposition of normal mode frequencies for 4-35

References

(1) Park, Y.; Kang, H.; Kang, H. Brute Force Orientation of Matrix-Isolated Molecules: Reversible Reorientation of Formaldehyde in an Argon Matrix toward Perfect Alignment. *Angew. Chem. Int. Ed. Engl.* **2016**, *56*, 1046–1049.

(2) Park, E. S.; Boxer, S. G. Origins of the Sensitivity of Molecular Vibrations to Electric Fields: Carbonyl and Nitrosyl Stretches in Model Compounds and Proteins. *J. Phys. Chem. B* **2002**, *106*, 5800–5806.

(3) Fried, S. D.; Boxer, S. G. Measuring Electric Fields and Noncovalent Interactions Using the Vibrational Stark Effect. *Acc. Chem. Res.* **2015**, *48*, 998–1006.

(4) Fried, S. D.; Bagchi, S.; Boxer, S. G. Measuring Electrostatic Fields in Both Hydrogen-Bonding and Non-Hydrogen-Bonding Environments Using Carbonyl Vibrational Probes. *J. Am. Chem. Soc.* **2013**, *135*, 11181–11192.

(5) Fried, S. D.; Wang, L.-P.; Boxer, S. G.; Ren, P.; Pande, V. S. Calculations of the Electric Fields in Liquid Solutions. *J. Phys. Chem. B* **2013**, *117*, 16236–16248.

(6) Schneider, S. H.; Boxer, S. G. Vibrational Stark Effects of Carbonyl Probes Applied to Reinterpret IR and Raman Data for Enzyme Inhibitors in Terms of Electric Fields at the Active Site. *J. Phys. Chem. B* **2016**, *120*, 9672–9684.

(7) Dalosto, S. D.; Vanderkooi, J. M.; Sharp, K. A. Vibrational Stark Effects on Carbonyl, Nitrile, and Nitrosyl Compounds Including Heme Ligands, CO, CN, and NO, Studied with Density Functional Theory. *J. Phys. Chem. B* **2004**, *108*, 6450–6457.

(8) Zoi, I.; Antoniou, D.; Schwartz, S. D. Electric Fields and Fast Protein Dynamics in Enzymes. *J. Phys. Chem. Lett.* **2017**, *8*, 6165–6170.

(9) Andrews, S. S.; Boxer, S. G. Vibrational Stark Effects of Nitriles I. Methods and Experimental Results. *J. Phys. Chem. A* **2000**, *104*, 11853–11863.

(10) Mohrmann, H.; Kube, I.; Lórenz-Fonfría, V. A.; Engelhard, M.; Heberle, J. Transient Conformational Changes of Sensory Rhodopsin II Investigated by Vibrational Stark Effect Probes. *J. Phys. Chem. B* **2016**, *120*, 4383–4387.

(11) Suydam, I. T.; Boxer, S. G. Vibrational Stark Effects Calibrate the Sensitivity of Vibrational Probes for Electric Fields in Proteins. *Biochemistry* **2003**, *42*, 12050–12055.

(12) Okuda, M.; Higashi, M.; Ohta, K.; Saito, S.; Tominaga, K. Theoretical Investigation on Vibrational Frequency Fluctuations of SCN-Derivatized Vibrational Probe Molecule in Water. *Chem. Phys.* **2018**, *512*, 82 – 87.

(13) Lindquist, B. A.; Furse, K. E.; Corcelli, S. A. Nitrile Groups as Vibrational Probes of Biomolecular Structure and Dynamics: An Overview. *Phys. Chem. Chem. Phys.* **2009**, *11*, 8119.

(14) Staffa, J. K.; Lorenz, L.; Stolarski, M.; Murgida, D. H.; Zebger, I.; Utesch, T.; Kozuch, J.; Hildebrandt, P. Determination of The Local Electric Field at Au/SAM Interfaces Using the Vibrational Stark Effect. *J. Phys. Chem. C* **2017**, *121*, 22274–22285.

(15) Mani, T.; Grills, D. C.; Miller, J. R. Vibrational Stark Effects To Identify Ion Pairing and Determine Reduction Potentials in Electrolyte-Free Environments. *J. Am. Chem. Soc.* **2015**, *137*, 1136–1140.

(16) Andrews, S. S.; Boxer, S. G. Vibrational Stark Effects of Nitriles II. Physical Origins of Stark Effects from Experiment and Perturbation Models. *J. Phys. Chem. A* **2002**, *106*, 469–477.

(17) Mani, T.; Grills, D. C. Probing Intermolecular Electron Delocalization in Dimer Radical Anions by Vibrational Spectroscopy. *J. Phys. Chem. B* **2017**, *121*, 7327–7335.

(18) Silverman, L. N.; Pitzer, M. E.; Ankomah, P. O.; Boxer, S. G.; Fenlon, E. E. Vibrational Stark Effect Probes for Nucleic Acids. *J. Phys. Chem. B* **2007**, *111*, 11611–11613.

(19) Voller, J.-S.; Biava, H.; Hildebrandt, P.; Budisa, N. An Expanded Genetic Code for Probing the Role of Electrostatics in Enzyme Catalysis by vibrational Stark Spectroscopy. *Biochim. Biophys. Acta. Gen. Subj.* **2017**, *1861*, 3053 – 3059.
